# Early indicators of exposure to biological threat agents using host gene profiles in peripheral blood mononuclear cells

**DOI:** 10.1186/1471-2334-8-104

**Published:** 2008-07-30

**Authors:** Rina Das, Rasha Hammamieh, Roger Neill, George V Ludwig, Steven Eker, Patrick Lincoln, Preveen Ramamoorthy, Apsara Dhokalia, Sachin Mani, Chanaka Mendis, Christiano Cummings, Brian Kearney, Atabak Royaee, Xiao-Zhe Huang, Chrysanthi Paranavitana, Leonard Smith, Sheila Peel, Niranjan Kanesa-Thasan, David Hoover, Luther E Lindler, David Yang, Erik Henchal, Marti Jett

**Affiliations:** 1 Division of Pathology, Walter Reed Army Institute of Research, Silver Spring, MD, USA; 2 Diagnostic Systems Division, United States Army Medical Research Institute of Infectious Diseases, USAMRIID, Ft. Detrick, Fredrick, MD, USA; 3 Computer Science Laboratory, SRI International, Menlo Park, CA, USA; 4 Geo-Centers, Inc. Rockville, MD; USA; 5 Department of Chemistry, Georgetown University, Washington, DC, USA; 6 Department of Bacterial & Rickettsial Diseases, Walter Reed Army Institute of Research, Silver Spring, MD 20910, USA; 7 Division of Retrovirology, Walter Reed Army Institute of Research, Rockville, MD, USA

## Abstract

**Background:**

Effective prophylaxis and treatment for infections caused by biological threat agents (BTA) rely upon early diagnosis and rapid initiation of therapy. Most methods for identifying pathogens in body fluids and tissues require that the pathogen proliferate to detectable and dangerous levels, thereby delaying diagnosis and treatment, especially during the prelatent stages when symptoms for most BTA are indistinguishable flu-like signs.

**Methods:**

To detect exposures to the various pathogens more rapidly, especially during these early stages, we evaluated a suite of host responses to biological threat agents using global gene expression profiling on complementary DNA arrays.

**Results:**

We found that certain gene expression patterns were unique to each pathogen and that other gene changes occurred in response to multiple agents, perhaps relating to the eventual course of illness. Nonhuman primates were exposed to some pathogens and the *in vitro* and *in vivo* findings were compared. We found major gene expression changes at the earliest times tested post exposure to aerosolized *B. anthracis *spores and 30 min post exposure to a bacterial toxin.

**Conclusion:**

Host gene expression patterns have the potential to serve as diagnostic markers or predict the course of impending illness and may lead to new stage-appropriate therapeutic strategies to ameliorate the devastating effects of exposure to biothreat agents.

## Background

Recent events have demonstrated that the capability to assess exposure and infection of individuals by biological threat agent (BTA) well in advance of onset of illness or at various stages post-exposure could offer important diagnostic and therapeutic benefits. Direct pathogen identification can be elusive since many pathogens sequester in tissues initially. Direct pathogen methods include the classical culture methods, immunoassay, and gene amplification of the pathogen. Although these methods are being improved for incredibly greater sensitivity [1-3], the efficiency of any diagnostic approach for direct pathogen assessment depends upon the presence of agent in a small specimen matrix. By the time detectable levels of pathogen are reached, it is frequently too late to halt the progression of the intractable illness [4,5]. Host responses that occur rapidly after exposure to specific pathogenic agents could provide the needed information for defense against the biothreat agents at time periods when clinical signs might include general malaise or flu-like symptoms that would not differentiate among diverse pathogenic agents (Additional file 1).

Correlation of the course of the infection and the disease progression with molecular responses provides opportunities to understand pathogenesis resulting from biological agent exposure. Symptoms of exposure to biological toxins such as *Vibrio cholerae *toxin (CT), *Clostridium botulinum *toxin A (BoNT-A), and Staphylococcal enterotoxin B (SEB) include violent reactions within hours of exposure and may lead to death in a few days. In contrast, *Brucella *infection *(B. melitensis *16 M) produces late-onset of mild symptoms that persist over a long period of time. Other bacterial pathogens, such as those causing anthrax (*B. anthracis*) or plague (*Yersinia pestis*), induce systemic, flu-like symptoms initially and progress to lethal shock and death days later. In the case of viral threat agents, Venezuelan equine encephalitis (VEE) initially causes serious illness, associated with severe malaise and an extended recovery time. Dengue (DEN-2) patients usually recover fully within days (< 1% mortality), but some individuals, especially those with prior dengue exposure, may develop dengue hemorrhagic fever (DHF). Therefore, the time when an individual is exposed, the incubation period, and the time of manifestation of the illness are crucial to designing diagnostic and therapeutic strategies.

In the post-genomic period, it is no longer unrealistic to hope that the examination of host responses, by interrogating large numbers of genes, could reveal unique responses to the various BTA. However, there are a number of obstacles yet to be overcome. Various pathogens may affect different tissues or cells that may not be available for diagnostic purposes. For example, botulinum toxins target neuromuscular synapses and cholera toxin aims for intestinal epithelial cells. As a first step toward the goal, we focused on gene expression changes in human peripheral blood mononuclear cells (PBMC) since they could be readily obtained from an exposed individual, thus taking advantage of their "reconnaissance" role. We carried out *in vitro* exposure to each of 8 biological threat or infectious agents. We confirmed the *in vitro* results by using peripheral blood mononuclear cells (PBMC) from nonhuman primates (NHP) exposed to a bacterial pathogen (*Bacillus anthracis*) or, separately, to a toxin (SEB) at various time points post-exposure to compare findings *in vitro* to those seen *in vivo*. We have identified host gene expression patterns that can discriminate exposure to various BTA, even at early time periods when flu-like symptoms occur.

## Methods

For the *in vitro* studies, the bacterial pathogens used were *Bacillus anthracis*, *Yersinia pestis*, *Brucella melitensis*; toxins: SEB, CT, BoNT-A; viruses: VEE and DEN-2.

We isolated RNA from human lymphoid cells after exposure and analyzed the gene expression patterns induced by these agents using cDNA arrays. We have not amplified our message for gene array analysis in order to avoid PCR-mediated bias.

### Bacterial strain and growth conditions

#### Y. pestis

*Y. pestis *KIM5 *Pgm *negative mutant bacteria (Laboratory stock) were grown on Brain Heart Infusion (BHI, Invitrogen, Rockville, MD) agar plates 30°C for 48 h. Pre-culture of *Y. pestis *bacteria were grown in BHI broth at 26°C overnight in shaker incubator at 180 rpm and were diluted 1/25 in fresh BHI medium. The organism was then grown at 26°C for 5 h (OD 600 ~0.5) and used for infection. To determine the MOI we counted serial dilutions of the bacterial culture by plating on (BHI) agar plates followed by incubation at 30°C for 48 h.

#### B. anthracis

Spores were prepared from *B. anthracis *Ames strain (pXO1+, pXO2+). Briefly, 5% sheep blood agar (SBA) plates were inoculated with *B. anthracis *Ames spores and incubated overnight at 35°C. Several isolated colonies were transferred to a sterile screw capped tube containing 5 ml of sterile PBS. NSM plates (New sporulation medium: per liter added Tryptone; 3 grams. Yeast extract; 3 grams. Agar; 2 grams. Lab Lemco agar; 23 grams. 1% MnCl_2_·4H_2_O; 1 ml) (150 × 25 mm Petri plate) was inoculated with 200 μl of prepared cell suspension. These plates were incubated for 48 hrs at 35°C, then checked for sporulation progress by microscopic examination. Continued incubation at room temperature was performed until free refractive spores constituted 90–99% of total suspension. Spores were then harvested from plates using 5 ml of sterile water. Spores were then washed 4 times in sterile water. Spores were checked for purity by plating 10 μl in triplicate onto 5% SBA plates and incubating overnight @ 35°C. Enumerations of spores were calculated via CFU/ml (determination of viable spores) and also for actual spores/ml using Petroff Hauser chamber.

#### B. melitensis

*B. melitensis *16 M strain was grown in shaker flasks overnight in *Brucella *broth at 37°C, plated on *Brucella *agar for 48 h at 37°C to obtain confluent growth. Bacteria were scraped from plates in pyrogen-free, sterile 0.9% NaCl for irrigation (saline), washed twice, concentration adjusted in saline to the appropriate OD_600_.

#### Venezuelan Equine Encephalitis Virus (VEE)

The Trinidad (Trd) strain used in these studies is a virulent virus of the epizootic IA/B variety of VEEV and was originally isolated from the brain of a donkey. Virus was diluted to an appropriate concentration in Hank's buffered saline solution containing 1% fetal bovine serum.

#### Dengue 2 Virus

DV-2 was grown and propagated in mycoplasma-free Vero cell lines. The viral titer was determined by limiting dilution plaque assays on Vero cells. All virus stocks and culture supernatants used in the present study were free from LPS and mycoplasma [6].

### Isolation of cells from Human PBMC using elutriation methods

Leukopheresis units were obtained from volunteer donors using the procedures outlined in our approved human use protocol, reviewed by the established Institutional Review Board at WRAIR. The written informed consent document was provided to the volunteers in advance of the procedure.

We obtained PBMC (74 blood draws over a period of ~2.5 years, and collected from ~8–10 AM to minimize variability) from healthy human volunteers who had been screened to be HIV and Hepatitis B negative, were from 19–61 years of age and both male and female. Human monocytes and lymphocytes of peripheral blood mononuclear cells were purified from leukopacks of healthy donors by centrifugation over lymphocyte separation medium (Organaon Tecknika, NC). Monocytes and lymphocytes were then further purified by counter flow centrifugation-elutriation with pyrogen-free, Ca^2+^- and Mg^2+^-free phosphate-buffered saline as the eluant. The resulting monocytes and lymphocyte preparations had greater than 95% viability. Monocytes and lymphocytes were mixed in the ratio 1:4 and were used immediately. Cell cultures were maintained in RPMI media at 37°C.

#### Exposure of monocytes and lymphocytes to the various pathogenic agents

Lymphoid cells were then exposed to a pathogenic agent under conditions (dose, exposure time) deemed optimal for biological activity for each agent by the pathogen specialist. The multiplicity of infection (MOI) of the bacteria or viruses to lymphoid cells was as follows: anthrax spores (1 or 3), VEE (1 or 3), DEN-2, (0.2 or 1), *Brucella *(2), and plague (20). Following a 30-min infection period cells were washed once with Hanks Balance Salt Solution (HBSS, Invitrogen, Rockville, MD). Both uninfected and infected cells were maintained in RPMI Medium 1640 with 10% human AB serum at 37°C 5% CO2 for the different time periods post exposure. Cells were harvested and total RNA isolated using Trizol reagent (Invitrogen, Carlsbad, CA).

The bacterial toxins, CT (3 nM), SEB (100 ng/mL), and BoNT-A (1 nM), were added to newly plated cells in flasks for the time period specified. Cells, incubated in the absence and presence of the toxins, were collected by centrifugation. Trizol™ (Invitrogen, Carlsbad, CA,) was added to the cells for RNA isolation and the cells were frozen at -70°C until use.

#### RNA isolation and cDNA arrays

RNA was isolated according to the Trizol method (Invitrogen, Carlsbad, California) followed with DNAse digestion [7]. The custom cDNA slides contained ~10,000 genes (Additional file 2). Stratagene reference RNA was labeled with Cy3 and used to compare with RNA (Cy5) from either control or exposed samples. RNA was labeled using Micromax-TSA labeling kits (Perkin Elmer, Boston, MA), hybridized and scanned in an Axon scanner. GenePix 3.0 (Axon) was used to analyze the scanned image. For studies using Human cDNA membranes (Clontech Laboratories, Palo Alto, California), RNA samples were labeled with radioactive ^33^P. After washing, the blots were exposed to Kodak screen and scanned in a BIORAD multifluor scanner. Atlas Image software (BD Biosciences Clontech) was used for spot alignment and normalization of the scanned arrays.

#### Statistical analyses and data scrutiny

**Figure 1:**
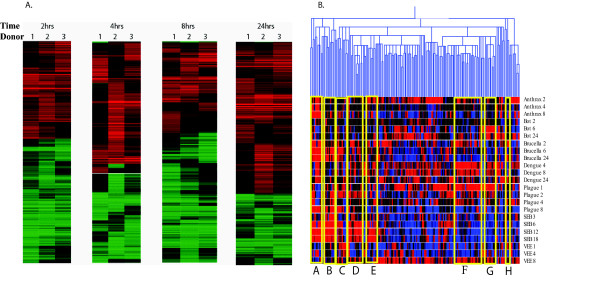
(a) *B. anthracis *exposure to PBMC from 3 different donors. Data shown are from exposures at 2, 4, 8 and 24 h. Data from each exposure time period were separately evaluated in order to identify common trends among the three donors (males, ages 61, 41, and 27 years old (respectively) with diverse ethnicity). (b). Comparisons of gene profiles for 8 pathogenic agents. Human PBMC were exposed to each of these pathogenic agents for at least 3 appropriate time periods. RNA was isolated, and the reverse transcript hybridized to cDNA arrays. Unique gene patterns were induced by BTAs. Cluster diagram of gene expression patterns use Gene Spring analysis to illustrate groups of genes that show discriminatory patterns for various threat agents. These genes were compared for their expression patterns across all agents and time points. Red is up regulated, blue is down regulated and black is no change compared to the control sample. The expression patterns illustrate how one can differentiate pathogenic agents by selection of sets of gene expression patterns for examination. (Gene accession ID numbers, rather than gene names, are all provided legibly in the graphs of Additional files).

We have adhered to "MIAME" (minimum information about microarray experiments) for all our studies. For each pathogen, 3–6 successive time periods were studied and for each time period, data from 2–4 separate experiments were obtained and, using the data from these multiple experiments, 2-way ANOVA analysis were carried out. GeneSpring version 5.0 (Silicon Genetics, San Carlos, CA) and Partek Pro 5.0 (St. Charles, MO) were used to visualize and analyze the data. Welch's ANOVA (p < 0.05) was performed followed by Benjamini Correction [8] for various sets of data, to find genes that varied significantly across samples and to identify patterns of gene regulation in PBMC exposed to various pathogens. For custom microarrays, we used the scatter plot smoother, Lowess algorithm [9], to normalize for dye bias among samples. We filtered the array data at 2 steps. In the first step, data filtration allowed only elements for which intensities in both channels were above twice background intensity. In the second step, elements that had intensities below twice the background intensity in one channel only were set at twice background levels. Last, to identify patterns of gene expression among different pathogens, *k*-means and self-organizing map clustering analyses were performed. Complete linkage hierarchical clustering of an uncentered Pearson correlation similarity matrix was also applied using the Eisen Cluster software [10], and the results were visualized with the program TreeView. We have used the major dataset (data from Figure 1b) as a training set to apply a class prediction method (GeneSpring 6.1) that uses the k-nearest neighborhood algorithm to classify blinded samples used as test sets.

#### Feature selection, computation and classification

Extracting discrete data: We applied the Greedy algorithm approach (46).

For each of the 8 pathogens at each time point evaluated (29) (where a condition is the combination of a pathogen and a time interval), we compute for each gene a regulation type which is a nonempty subset of {U, D, S}. For a given condition and gene, the regulation type contains U (respectively D, S) if for at least one array for that condition, using either sum or median normalization techniques and either difference or ratio criteria, the gene appears to be up regulated (respectively down regulated, stay the same). Thus we model both variation between donors and experimental error. The regulation type {U, D} is treated as if it were {U, D, S}; i.e. we consider it to provide no information.

Ordering the genes: For each value of n, we would like to select the n genes which best distinguish between the 29 conditions. Since this is an intractable task, we compute an approximation by ordering the genes according to a heuristic, and for each n, choose the first n genes from the list. There are two straightforward greedy approaches to generating such a list. In the grow approach we start with an empty list and at each step add the gene that gives the new list with the best discrimination power. In the shrink approach we generate the list in reverse order by starting with the full set of genes and removing the one that that leaves the remaining set with the best discrimination power.

We estimate the discrimination power of a set of genes by computing an integer vector of length 812, containing an entry for each of the 29 × 28 ordered pairs of distinct conditions, which itself is a sum computed over all genes in the set. The values summed are the number of elements in the regulation type for the first condition of the pair that do not occur in the regulation type for the second condition. This vector is then sorted least element first. To compare the discrimination power of two gene sets, their vectors are computed and compared lexicographically, with larger vector considered to correspond to the gene set with better discrimination power. The first 50 genes to be ranked by this method are listed in the Additional file 3.

#### *In vivo* anthrax exposure

Animal work was conducted in compliance with the Animal Welfare Act and other federal statutes and regulations relating to animals and experiments involving animals and adheres to principles stated in the Guide for the Care and Use of Laboratory Animals, NRC Publication, 1996 edition. Nine anthrax-naïve rhesus macaque NHPs were exposed to approximately 8 LD_50 _of B. anthracis spores (Ames strain) by a head-only aerosol exposure system. Blood was drawn before exposure to determine baseline values. After exposure, 3 animals were euthanized at each 24, 48 and 72 h and blood taken for various analyses, including gene response patterns. A full necropsy was performed to collect biological samples for use in B. anthracis diagnostic assay development.

#### Primer set design

Primer sets were designed for selected genes for expression profile confirmation. Additional file 4 lists the accession numbers and sequences for both sense and antisense primers.

#### Real-time PCR

Total RNA from the all the pathogenic agent studies was reverse-transcribed simultaneously using the same master mix. The cDNA was then used to perform real-time PCR using BIORAD I cycler and the light cycler DNA master SYBR green I kit (Roche Diagnostics, Indianapolis, Indiana). The 18S gene was used as an endogenous control to normalize the HIF-1, GBP, and C5AR genes. Serial 10-fold dilutions of lymphoid cDNA were used to determine the PCR efficiency of each primer set. The slope value was applied to the formula E = 10^-1/m ^- 1 where m = slope value. The Ct (threshold cycle) values for all the genes were converted to fold change using the formula (1 + E)^ΔCt^, where E denotes the efficiency of the primer set for a gene. ΔCt denotes the difference between the Ct values of control and treated samples of a given gene. (Personal Communication, C. Baker, National Institutes of Health)

## Results

### Host gene expression *in vitro*

Microarray analysis was carried out at 3–6 time periods post exposure of PBMC to each pathogen or vehicle. Prior studies [11] showed specific gene sets related to sex, age and other parameters, therefore it was important to first identify genes that are normally variant among healthy humans. Data from only the control samples of these healthy donors were subjected to ANOVA (p =< 0.05) and 6% of the genes varied widely among the individuals who were healthy human donors. These genes that showed inconsistent expression profiles were excluded from further comparisons among the data sets from both control and exposed samples. This provided a baseline to confidently identify transcriptional responses induced by bacteria (anthrax, plague, *Brucella*), toxins (CT, SEB, BoNTA), or viruses (Dengue, VEE). Expression ratios of 10,000 genes on the custom array (accession numbers of which are listed in Additional file 2) and genes (Additional file 5) in Human Atlas 1.2 were determined by comparing the levels of mRNA in control and pathogen-treated PBMC paired for each exposure time frame. Each measurement was carried out at least 3 times.

### Consistency of responses

We used PBMC from at least 3 different donors, exposing cells to pathogen or vehicle for specified periods of time. Figure 1a is a cluster analysis of exposures to B. anthracis for 2, 4, 8 and 24 h exposures. The result from the 3 different donors (male, ages 61, 27, 41) is closely replicated among the donors.

### Unique gene patterns induced by BTAs

**Figure 2:**
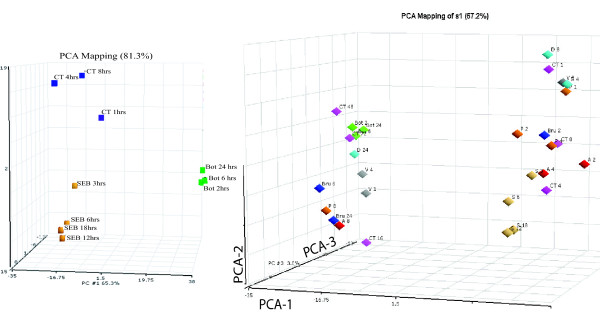
PCA relational analysis to show how the gene profiles (various exposure times) cluster for each toxin (a) and the relationship among the various pathogens (b). Human PBMC were exposed to each of these pathogenic agents for at least 3 appropriate time periods. RNA was isolated, and the reverse transcript hybridized to cDNA arrays.

The gene responses were dissected to identify sets of genes that will differentiate one agent from another based on the patterns of host gene induction. The GeneSpring (Silicon Genetics, California) clustering diagram illustrates gene expression patterns that can discriminate among the various pathogenic agents (Fig. 1b) by identification of sets of genes where up regulation (red) and down regulation (blue) is seen for specific pathogens. The combination of these selected genes can be the foundation for designing specific diagnostic assays for exposure to one or more agents. For example, gene sets A and B (column labels at the bottom of the dendogram), differentiate *B. anthracis *from all other pathogenic agents except Dengue; gene sets F and G readily illustrate expression patterns that differentiate gene responses to these 2 pathogenic agent. Similarly, gene sets D and E and H show host responses to SEB that are distinguishing; BoNTA and VEE show similar patterns with gene sets A and B, but are readily separated by gene set C. Principal component analysis (PCA) (Fig. 2a) illustrates clustering relationships that show marked differences in overall gene patterns among the 3 toxins used in this study. Additionally, gene patterns for the earliest exposure for SEB or CT clustered less closely with the later exposure times (Fig. 2b), but when observed relative to all pathogens, the four exposure time periods for SEB were relatively closely clustered. A striking observation (Fig. 2b) is that for all pathogens except SEB, the longest exposure times differ markedly from the clusters of the early time periods. For *B. anthracis, Y. pestis, B. melitensis*, and CT, those late exposure times cluster together for these various pathogens. This loss of pathogen-specific responses *in vitro* after lengthy exposure was not seen for the *in vivo* studies.

### Use of training and test data sets for classifying test exposures

To determine whether the microarray data obtained in this study can be used to predict the exposure type of an uncharacterized sample or condition, we applied a supervised learning method for class prediction (GeneSpring) that uses the k-nearest neighbor algorithm. When algorithm was applied on the data set (training set) to predict the exposure type of a data set obtained from an exposure to *Y. pestis *(test set), we were able to correctly predict the type of exposure with a p < 0.02. We previously reported that a set of predictor genes was identified when samples from exposures of piglets to SEB were used as test sets [12,13].

### Functional classification of genes differentially regulated

**Figure 3:**
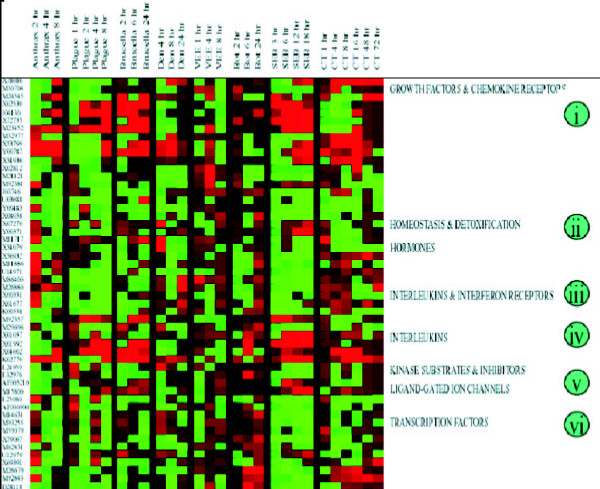
Functional categories of genes that show similarities and differences between these pathogens. Accession numbers are shown associated with Figure 2, Additional files. Human PBMC were exposed to each of these pathogenic agents for at least 3 appropriate time periods. RNA was isolated, and the reverse transcript hybridized to cDNA arrays.

Gene ontological analysis was carried out for the genes that were differentially expressed. Comparison of gene responses, based on functional similarities, not surprisingly, showed many up regulated genes coding for inflammatory mediators (Fig. 3). We clustered and sorted the differentially expressed genes by their functional classification. These functional classifications are depicted in Figure 3. For gene group (*i*) "Growth Factor, Cytokines & Chemokines," anthrax, *Brucella *and SEB showed major up regulation of most genes coding for inflammatory mediators; the other 5 agents had mixed or modest effects. Similarly, categories (*iii*) "Interleukins and Interferon Receptors" and (*iv*) "Interleukins" showed up regulation by most pathogens, notable exceptions being the viruses. Down regulated genes, though seen extensively throughout the study, displayed functional clustering for each pathogenic agent such as (*ii*) "Homeostasis & detoxification," (*v*) "Ligand-gated ion channels," and so forth. Plague induced high levels of interleukin-6, macrophage inflammatory protein-1 beta, tumor necrosis factor-alpha (TNF-α), and granulocyte macrophage colony stimulating factor (GM-CSF) when compared with *Brucella *and anthrax. Not surprisingly, the superantigen SEB displayed kinetic patterns for over expression of interferon-γ, IL-2, IL-6, MIP-1α, and GM-CSF (Fig. 3). There are major differences in expression of death receptors, homeostasis, and caspases, examples of which include defensins and certain oxidases (homeostasis) that are down regulated by plague and SEB (Fig. 3, bullet *ii*). A large number of transcription factors are down regulated by anthrax, *Brucella*, and SEB, but plague consistently down regulated the widest range of these genes.

### Gene responses induced by BTAs *in vivo*; comparison with *in vitro* changes

**Figure 4:**
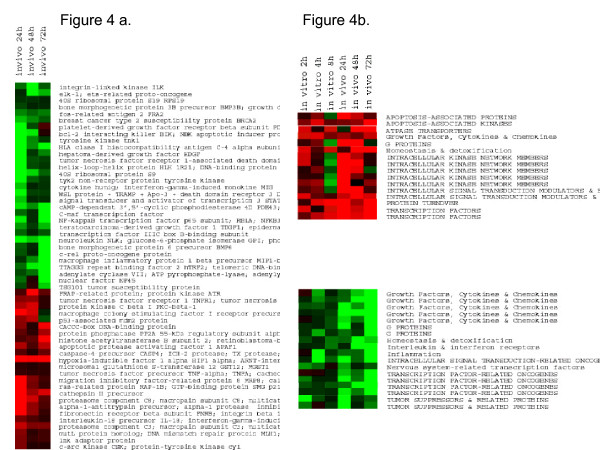
Comparison of host gene responses in vivo and in vitro exposures to anthrax. Gene expression profiles in PBMC from healthy human donors exposed to anthrax spores in vitro were compared with gene expression patterns obtained in PBMC taken at 24, 48 and 72 hr after exposure of NHP to anthrax spores by aerosol challenge. (a) Gene cluster analysis of significantly altered genes in vivo. (b) comparison of gene expression profile between in vivo and in vitro exposures.

**Figure 5:**
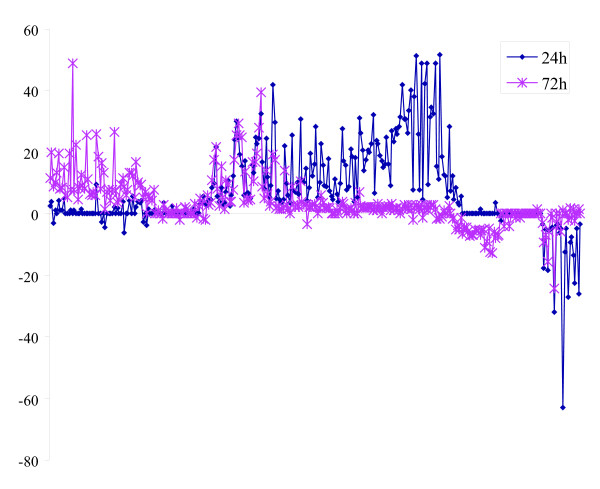
Clustered sets of genes to illustrate stage-specific vs. commonly expressed genes for in vivo exposures of NHP at 24, 72 h or at both time periods.

To determine gene changes induced by BTAs in an animal model, NHP were exposed to *B. anthracis *spores by aerosol challenge. This model has been characterized previously to mimic inhalation anthrax in humans. Blood samples were collected 24 h, 48 h, and 72 h post exposure (by 72 h the NHP were beginning to show signs of the illness, which progresses very rapidly to lethality). The gene expression profiles for *in vitro* exposure of PBMC to anthrax spores were compared with those found in isolated PBMC at various time periods from NHP. Even by 24 h, a robust response was observed (Fig. 4a), showing up regulation of genes coding for proteases; proteosome components c2, c3, c5; various cytokines; pro-apoptotic genes; cyclic adenosine monophosphate (cAMP)-related kinases, cAMP regulated transcription factors; and hypoxia inducible factor-1 (HIF-1). Down regulated genes included tyrosine kinases, cytokine receptors, growth factors, and adenosine diphosphate (ADP) ribosylation factors. Comparison of the *in vivo* results with the *in vitro* changes induced by anthrax (Fig. 4b), showed remarkable similarities in gene patterns. Clearly many more changes were observed *in vivo* than *in vitro*. Certain surface antigens showed significant alteration that was unique to anthrax exposure. Diagrams were constructed to identify sets of genes that were up regulated (Fig. 5) at either 24 (blue) or 72 h (red); other gene sets showed up regulation at both time periods (center of graph, genes in both blue and red). Similarly, certain gene sets showed unique and common down-regulation patterns (far right, Fig. 5).

**Figure 6:**
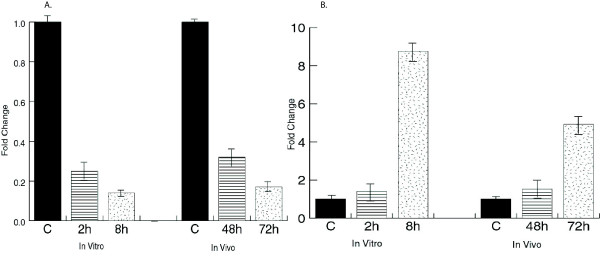
Confirmation of selected gene changes by RT-PCR with in vitro and in vivo samples for IL-6 (a) and Transducin beta-1 subunit, GNB1, (b).

**Figure 7:**
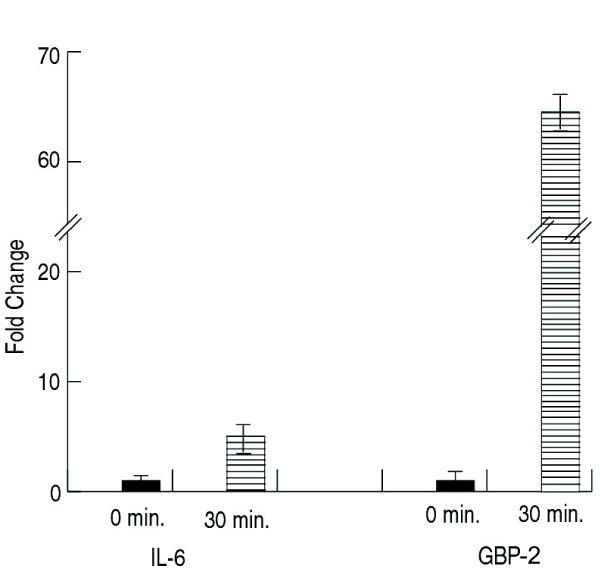
Expression of GBP-2 and IL-6 genes after in vivo SEB exposure of NHP for 30 min. Gene expression profiles in PBMC from NHP exposed to SEB for 30 min. RNA samples were isolated and used in the PCR assays using primers specific for IL-6 and GBP-2.

A few genes were selected that showed changes induced by *B. anthracis *exposure were confirmed by RT-PCR, and the level of expression was compared both *in vitro* and *in vivo* after anthrax exposure. The *in vivo*/*in vitro* trends were very similar for many genes including IL-6 (Fig. 6a) and Transducin beta-1 subunit (GNB1) (Fig. 6b). Altered regulation of that G-protein was not seen with the other pathogenic agents. In an experiment of SEB exposure to NHP (Fig. 7), IL-6 and guanylate binding protein GBP-2 were up regulated (6- and 65-fold, respectively) by 30 min post-exposure and the increased expression persisted through 24 h (the last time point tested, data not shown). Among all the pathogens studied so far, SEB was found to be the only pathogen to dramatically alter GBP-2.

### Evaluating the gene selections

**Figure 8:**
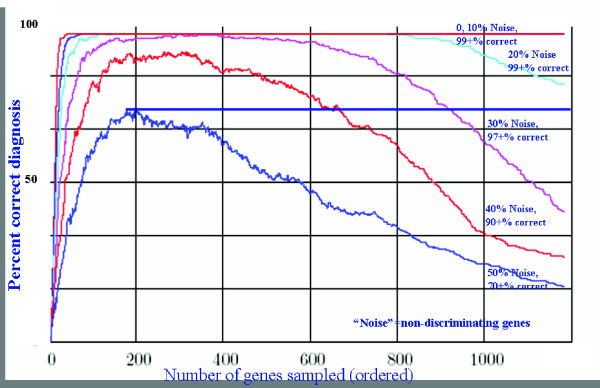
Ordered genes and resulting percentage correct classifications. For a given number of genes, a set of genes most able to discriminate between disease states is selected. Simulations of noisy readings of patient gene expression levels are performed for varying levels of noise. Each colored line plots the percentage of correct classifications versus the number of genes used to make the classification for one particular percentage of random values in the simulated readings. With no noise, very few genes are required to discriminate perfectly. With high noise levels (say, 50%), even 1000 genes cannot reliably discriminate well.

In order to evaluate the quality of the set of genes selected by the two methods above, a set of 10 simulated samples was generated for each condition by randomly choosing a set of values consistent with the conditions regulation types. Sets of samples in which values had a 10%, 20%, 30%, 40% and 50% chance of being chosen at random were also generated. Each test sample was then classified using the chosen set of genes by scoring it against each of the 29 conditions. A condition scored 1 for every gene on which its regulation type was consistent with the sample. The test sample was considered to be classified correctly if the condition from which it was originally generated had the unique highest score. The results using the list of genes obtained by the "grow" (respectively "shrink") method are shown in Figure 8.

Comparison of gene selection approaches: Because there is overlap between the gene types for some pairs of conditions, there must exist a sample consistent with some condition that cannot be classified correctly as that condition. Nevertheless for the generated data, both approaches obtained perfect classification at the 20% noise level for selections of between 150 and 775 genes. Even at the 30% noise level the grow method achieved perfect classification with 297–305 genes and the shrink method achieved perfect classification with 340–342 genes. Interestingly, in the added noise cases, gene selections above a certain size start to perform worse. This is because removing genes that play little role in distinguishing between conditions removes added noise without removing much discrimination power.

We have used simulated random noise perturbing the input data samples, since we had no prior basis to assume any specific bias in noise (direction or subset of genes). If noise levels on a given sample were for some reason biased toward the identified patterns of some other disease for that subject, our technique will not perform as well as it does against random noise. Since at this point possible bias or coloration of the noise is unknown, we experimented with a range of noise levels, including levels well higher than the total noise experienced in modern gene expression platforms.

### Gene profiles to discriminate control from infected lymphoid cells

**Figure 9:**
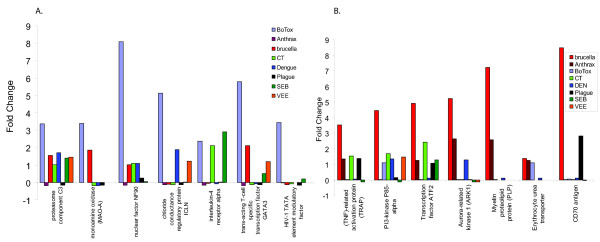
Expression patterns of genes that were at baseline levels in all controls and showed unique expression patterns in (a) BoNT-A exposures or (b) *B. melitensis *exposures compared to all 8 pathogens.

To identify common gene profiles that existed among all these individual donors, we used GeneSpring to identify shared baseline expression levels of genes examined for 75 control samples. As these control datasets were subjected to various analyses, after excluding normally varying genes, we noticed genes that were expressed at low levels in the control samples but were significantly overexpressed in response to one or more pathogens. We selected genes that showed a dramatic change in expression level and could be used to discriminate among the pathogens. Gene profiles from two pathogens are shown with BoNT-A (Fig. 9a) or *B. melitensis *(Fig. 9b) in which the indicated genes readily differentiated it from the other 7 BTAs. Although some of these genes were slightly up regulated by one or more of the other pathogens, even those few genes illustrate the possibilities of distinguishing BoNT-A or *B. melitensis *from each of the other 7 pathogens.

### Confirmation of gene changes by real-time PCR

**Figure 10:**
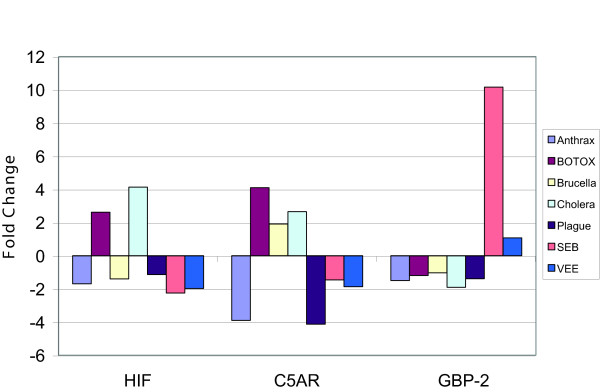
Real-time PCR determination of gene expression in response to each of 8 pathogenic agents. Primers were designed for these 3 genes and 18S, which was used as a reference gene for comparison of these 3 test genes. GBP-2 was a gene that was identified as being massively up regulated by SEB using differential display PCR (Mendis, et al) and was of particular interest to us.

To further confirm the expression levels of a few selected genes, we performed semi-quantitative real-time PCR using the same RNA samples isolated from lymphoid cells that had been exposed to the various pathogenic agents/vehicle and used to carry out the microarray experiments. Gene expression for 3 selected genes are compared based on real-time PCR along with gene array results. Expression of GBP-2, which was significantly up regulated by exposure of lymphoid cells to SEB (~10-fold by this technique vs. 6-fold from the microarray analysis), was not altered by any other pathogenic agents studied. Also up regulation was observed for HIF-1 and C5AR by BoNTA and CT and there was good agreement with data from the microarray studies (Fig. 10).

## Discussion

The objective of this study was to use host gene expression responses to aid in detection of exposure to biological threat agents, we aimed to a) discriminate among various pathogenic agents that start with similar flu-like symptoms [14] yet lead to severe illness by various routes (Additional file 1) select sets of genes that can convincingly be used to differentiate normal from exposed samples c) identify sets of genes that could be used for very early detection of exposure or estimating stage of illness post-exposure and d) continue to characterize similarities and differences in host responses for *in vitro* exposures to show some predictability for host gene profiles in NHP models that replicate the illness induced in humans. In another study, we found sets of genes from SEB exposures *in vitro* also were predictive of *in vivo* responses in a piglet model of SEB-induced lethal shock [12].

The approach depends on circulating lymphoid cell mRNA responses reflecting a historical record (by their mRNA responses) of encounters with pathogenic agents. Use of unique gene patterns for diagnosis has been shown for certain cancers [15-17]; another study showed that various pathogens induced unique gene responses in lymphocytes [18] and in dendritic cells [19]. To minimize variability as reported [11] in baseline gene expression among donors, in our study blood was collected at the same time of the day for all donors. Of the 8 pathogenic agents in this study, most have been characterized as having rapid effects on lymphoid cells [14,20]. For BoNT-A and VEE, the primary target tissues are inaccessible, although VEE has been shown to interact with lymphoid cells [21]. The gene expression data suggest that pathogen binding to specific receptors on the human PBMC initiates a series of events that contribute to the ultimate illness, producing host responses indicative of a particular pathogenic agent (Additional file 6) or showing common responses typical of a severe inflammatory reaction (Fig. 3).

The kinetics of the course of the infection and the disease process is essential in the study of gene changes induced in the host by these pathogens (Additional file 6). Upon inhalation of *Y. pestis *by NHP, infected alveolar macrophages migrate to the liver and spleen [22,23] where they proliferate rapidly (~24 h). It is thought that LPS from the bacterial cell wall of *Y. pestis *(Gram-negative) induces circulatory collapse and widespread organ failure, leading to death within days [24]. SEB, CT, and LPS can induce rapid onset of illness (even less than 1 h), involving loss of regulation of vascular tone, vascular leakage and end-stage organ failure, in 1 to 3 days [14,25,26]. *B. anthracis *(Gram-positive) is transmitted as spores, which, upon inhalation, become engulfed by macrophages and transported to lymph nodes [20,27-31]. Upon production of a sufficient bacterial load, flu-like symptoms occur followed by sudden onset of respiratory distress, progressive shock, and death. As recent events have demonstrated, with cases of inhalation anthrax, treatments initiated after patients became seriously symptomatic can be marginally successful [27]. In this study, host gene expression responses in NHP to *B. anthracis *exposure were seen at the earliest time point examined, 24 h (Fig. 4b). In contrast, in these same NHP, a sufficient pathogen load to be identified by culture techniques did not occur until day 3 and clinical diagnosis would not be possible until ~ day 4–5, much too late to initiate reliably effective treatments.

Studies of pathogenesis show up regulated cytokine genes as a common response for many pathogens [14,32] and that would not, necessarily, distinguish among them (Fig 3). Therefore, we focused on host responses that may potentially identify stage-specific targets and can also serve as early diagnostic markers. The regulation of certain early genes is transient and may relate to factors that participate in recruitment of monocytes to the sites of infection. The genes that are expressed late relate to DNA damage-inducing proteins, hypoxia-inducible proteins, and proteases. Characterization of apoptosis as a result of exposure was reported for SEB, DEN-2 [33], plague [34], and anthrax [20,27,35] in numerous cell types, including those of lymphoid origin. We observed induction of apoptotic genes by these agents in our studies (Additional file 6). In contrast, *Brucellae *is known to inhibit apoptosis in their mononuclear phagocytic host cells [36] and we also observed pro-apoptotic genes to be down regulated by Brucella. In regard to the findings with plague exposure, it is important to note that these infections occurred under conditions that limit the ability of *Yersinia *outer proteins (YOP) to alter host cell physiology by down regulation of cytokines [34] and oxygen radicals [37] in calcium-free cultures of macrophages. We observed up regulation of certain cytokines after 1–2 hr of exposure to *Y. pestis *and a pattern of gene expression that can explain reduced synthesis of oxygen radicals. We suspect that different biochemical mechanisms contribute to pathogenesis when YOPs are produced [38,39].

In contrast to common infectious diseases, human cases of exposure to some of the biological threat agents are rare. Indeed, for certain of these pathogens, there is no appropriate animal model that replicates the illness as it appears in humans. Furthermore, dose effects and other variations suggest the need to investigate *in vitro* approaches that show some correlations to *in vivo* findings. For *in vivoB. anthracis *studies, PBMC from the spore-exposed animals was collected 24, 48, and 72 h. The *in vitro* study utilized PBMC (from healthy donors) exposed to spores for various time periods. We found many more gene expression changes *in vivo* than *in vitro*, perhaps because *in vivo* changes include both primary and secondary responses. Comparisons of *in vitro*/*in vivo* results showed similarities in genes that code for lymphoid receptors/signaling pathways that, when taken as a group, showed a pattern specific for *B. anthracis *and include a G-protein Transducin beta subunit (GNB1), cAMP related genes, Calmodulin regulated genes, cytokines and MAPKK (mitogen activated protein kinase kinase), some of which had previously been reported in response to anthrax exposures [40]. Not unexpectedly, genes coding for cytokines showed similarities *in vitro* vs *in vivo* (Fig 5 and 6). Our microarray data were in accordance with recent reports by Pickering et al. that showed up regulation of some of the cytokines in response to infection by *B. anthracis *spores including TNF-α, IL-8, IL-1β, GM-CSF, IFN-γ and IL-6 [41]. However, these genes, alone, would not necessarily distinguish anthrax from other pathogens. In general, the genes expressed by 4 h *in vitro* and 72 h *in vivo* were similar and correlated to the symptoms that appear after progression of inhalation anthrax in NHP.

Aerosol challenge of SEB in NHPs up regulated (65-fold) the gene coding for interferon-regulated GBP-2 (Fig. 7) but *in vitro* (Figure 10) it was up-regulated 10-fold. *In vivo*, GBP-2 upregulation occurred by 15 min post exposure. Other pathogens showed minor or no effects on the expression of that particular G-protein (Figure 10). This may not be surprising in light of seminal studies showing that CT and pertussis toxin work through different guanine triphosphate (GTP)-binding proteins to regulate intracellular cAMP levels [42,43]. This study confirmed gene expression responses induced by CT, anthrax, and *Brucella *that are known participants in regulation of adenylyl cyclase as well as those relating to ADP ribosylation factor [44-46] (Additional file 6). We observed a down regulation of the host adenylyl cyclase but an up regulation of cAMP-related genes upon anthrax exposure in NHP samples (Figure 4a). Because *B. anthracis *has its own adenylyl cyclase, it may be playing a role in affecting cAMP-related genes of the host [29,47].

Since most biothreat pathogen exposures start with flu-like symptoms, discriminating them from common pathogenic illnesses for early diagnosis at a treatable stage is one of the critical issue. Even though these 8 pathogens initially cause similar symptoms, such as malaise, fever, headache, and cough, unique sets of genes are induced by each and can be related to the course of illness [14] (Additional files 1 and 6). Using these signature gene profiles to assess possible exposure to pathogenic agents or to differentiate them from non-lethal illnesses, when the classical identification of a pathogen is not conclusive, has the potential to fill a gap in the arsenal of diagnostic tools.

## Conclusion

Rapid detection, before the symptoms appear or even at various stages of illness offers the opportunity to initiate stage-specific therapeutic approaches to ameliorate the devastating results of these pathogenic agents. The use of host genomic markers offers an option to differentiate classes of pathogen exposure, gauge severity of impending illness and apply appropriate therapeutic strategies.

^1^Genes were selected and their expression profiles compared with gene array and real-time PCR. 18S was used as a reference gene for comparison of these 3 test genes.

^2^GBP-2 was a gene that was identified as being massively up regulated by SEB using differential display PCR (C. Mendis, et al).

nd = not determined

## Competing interests

The authors declare that they have no competing interests.

## Authors' contributions

RD and RH drafted the manuscript, performed the genomic analysis, data mining and the apoptosis studies. GVL; BK; XH; CP; MJ; LS; NK; DH and LL carried out the exposures to the various pathogens. SE and PL participated in the statistical analysis of the microarray data. PR; AD; AM; CM; CC; AR; SP and RN participated in the microarray studies for the different pathogens. MJ conceived of the study, and participated in its design and coordination. GVL; DY and EH participated in the design and coordination on the study. All authors read and approved the final manuscript.

## Disclaimer

Material has been reviewed by the Walter Reed Army Institute of Research. There is no objection to its presentation and/or publication. The opinions or assertions contained herein are the private views of the author, and are not to be construed as official, or as reflecting true views of the Department of the Army or the Department of Defense.

## Pre-publication history

The pre-publication history for this paper can be accessed here:



## Supplementary Material

Additional file 1 Comparison of time course of progression of illness for selected BTAs and pathogens: Graph courtesy of COL George Korch, USAMRIID, Ft. Detrick, Maryland. Hatched marks indicate onset of flu-like symptoms (fever, headache, chills) for each agent, *X *indicates time frame in which death usually occurs (case fatality rate, CFR), and the number of *X*s suggest the degree of lethality if untreated early in the course of illness. (i) The toxins (yellow bars) cause onset of acute illness within a few hours, but side effects can persist for many weeks. (ii) Bacterial BTAs (pink bars) follow different time course after infection ranging from days to weeks. Many bacterial infections begin with flu-like symptoms, but proceed to respiratory distress and lethal shock within ~ a week. In contrast, a prolonged illness is common in brucellosis, which is caused by Gram-negative bacteria (*B. melitensis. B. suis*, and *B. abortus*) that are highly infectious via aerosol route. (iii) Viruses (green bars) VEE and DEN infections each progress differently because VEE can proceed to the meninges of the brain, developing into encephalitis. In the case of dengue, the incubation period is 3 to 15 days, with the acute febrile illness lasting 3–5 days. The period of mortality is associated with cessation of the febrile illness or with secondary complications in DHF. Click here for file

Additional file 2 A list of genes on the custom array. This table shows a list of the genes that are present on the microarrays used in this study. Click here for file

Additional file 3 List of the first 50 genes that were selected using the Grow/Shrink method. This table lists the genes that passed the statistical analysis using the Grow/Shrink method. Click here for file

Additional file 4 List of the accession numbers and sequences of the primer sets used in this study. Click here for file

Additional file 5 Lists the genes of the Clontech 1.2 human array. Click here for file

Additional file 6 Comparisons of gene profiles for 8 pathogenic agents. Cluster analysis of gene expression profiles of PBMC exposed to the 8 pathogens. Human PBMC were exposed to each of these pathogenic agents for appropriate time periods, and the results are sorted based on functional responses, rather than clustering of similar gene expression patterns. RNA was isolated, reverse transcribed and hybridized to cDNA arrays. Red bars indicate up regulation, green bars show down regulation of the genes, and selection of genes for significance is defined in methods. The numbered bullets (right margin of the figure) indicate families of genes showing similarities or unique properties. Gene accession numbers are shown to the left of the figure. Click here for file
