# Supplementary material for: Early indicators of exposure to biological threat agents using host gene profiles in peripheral blood mononuclear cells
Source: BMC Infect Dis. 2008 Jul 30;8:104. doi: 10.1186/1471-2334-8-104 (PMC2542375; doi:10.1186/1471-2334-8-104)
Supplement: Additional file 2 — A list of genes on the custom array. This table shows a list of the genes that are present on the microarrays used in this study. [file 1471-2334-8-104-S2.pdf]

Gene/protein	Accession number
Human DNA sequence from clone RP3-453C12	BE858375
hypothetical protein MGC5306	AW410368
chemokine (C-C motif) receptor-like 2	AF015525
transcriptional intermediary factor 1	AL365456
1-acylglycerol-3-phosphate O-acyltransfe	BG323806
ESTs	AI918219
GLI-Kruppel family member HKR3	XM_001302
cytochrome P450, subfamily IIA (phenobar	AW577399
Junk, low PCR	EMPTY
inositol polyphosphate phosphatase-like	AA640010
KIAA0595 protein	NM_015062
gamma-aminobutyric acid (GABA) B recepto	XM_011493
S100 calcium-binding protein, beta (neur	AV717894
tafazzin (cardiomyopathy, dilated 3A (X-	AA485993
ESTs, Weakly similar to ALU4_HUMAN ALU S	AV661518
sodium channel, voltage-gated, type V, a	NM_000335
distal-less homeobox 4	BG479855
homeo box D4	XM_010802
Homo sapiens cDNA FLJ11027 fis, clone PL	AK001889
Junk, low PCR	EMPTY
splicing factor, arginine/serine-rich 8	AA256490
RAE1 (RNA export 1, S.pombe) homolog	BG107329
ATPase, Na+/K+ transporting, beta 3 poly	AU151263
hypothetical protein	AI052537
Junk, low PCR	EMPTY
ribosomal protein S27a	BG479933
TATA element modulatory factor 1	NM_007114
butyrophilin, subfamily 3, member A1	BE767964
KIAA0092 gene product	XM_005991
milk fat globule-EGF factor 8 protein	AI340354
erythrocyte membrane protein band 4.1-li	AL121895
Homo sapiens cDNA FLJ10935 fis, clone OV	AK001797
inactivation escape 1	NM_003669
chromosome 8 open reading frame 2	AI954732
dual specificity phosphatase 6	NM_001946
dihydropyrimidinase-like 4	XM_011864
CGI-43 protein	BF110838
KIAA0887 protein	AA026620
secreted frizzled-related protein 1	NM_003012
A kinase (PRKA) anchor protein 6	XM_012369
RAN, member RAS oncogene family	BG323372
hypothetical protein FLJ22622	AI420820
amino-terminal enhancer of split	AL552021
actinin, alpha 2	NM_001103
macrophage scavenger receptor 1	D13264
RAB13, member RAS oncogene family	AV735080
carbonic anhydrase III, muscle specific	NM_005181
Bloom syndrome	XM_012436
mutS (E. coli) homolog 6	BG434326
ems1 sequence (mammary tumor and squamou	BE391814

thyroid hormone receptor interactor 10	AW885403
6-phosphofructo-2-kinase/fructose-2,6-bi	XM_003245
Junk, low PCR	EMPTY
RNB6	AW168175
HIV TAT specific factor 1	AA281368
KIAA1332 protein	AW977525
thioredoxin-like, 32kD	NM_004786
vitamin D (1,25- dihydroxyvitamin D3) re	AI874346
mitogen-activated protein kinase kinase	AL135644
hypothetical protein FLJ20718	NM_017939
small inducible cytokine subfamily B (Cy	XM_003507
Junk, low PCR	EMPTY
hyaluronoglucosaminidase 3	AF036035
interleukin 4 receptor	NM_000418
KIAA0257 protein	D87446
thyroid hormone receptor coactivating pr	BG286394
protein regulator of cytokinesis 1	BF965920
ubiquitin-conjugating enzyme E2A (RAD6 h	AI278702
nuclear receptor coactivator 1	W72793
KIAA0135 protein	AA459101
UDP glycosyltransferase 1 family, polype	NM_019093
adrenomedullin	NM_001124
ESTs, Weakly similar to AF279265 1 putat	AI220943
X11L-binding protein 51	XM_012929
RNA-binding protein S1, serine-rich doma	AL559988
tektin 2 (testicular)	AB033823
paired basic amino acid cleaving enzyme	XM_007719
NADH dehydrogenase (ubiquinone) 1 alpha	AA657981
lipase, hepatic	AV660407
transcription factor 8 (represses interl	U12170
Junk, low PCR	EMPTY
Junk, low PCR	EMPTY
similar to murine leucine-rich repeat pr	AK001991
Junk, low PCR	EMPTY
proteasome (prosome, macropain) 26S subu	BE908811
Ras homolog enriched in brain 2	AW519065
nuclear factor of activated T-cells, cyt	NM_004554
ESTs, Weakly similar to protein that is	AI889276
protein kinase C-like 2	NM_006256
colony stimulating factor 2 (granulocyte	BE669962
ESTs	BF791969
hypothetical protein FLJ12619	AU138364
solute carrier family 9 (sodium/hydrogen	AA022704
BRCA1 associated RING domain 1	AA558464
BTG family, member 3	BE896159
retinoic acid induced 3	BF212425
nel (chicken)-like 1	NM_006157
RAB4, member RAS oncogene family	BC004309
egf-like module containing, mucin-like,	AC004262
KIAA0440 protein	AB007900
Junk, low PCR	EMPTY

hypothetical protein MGC3101	BF196967
tissue inhibitor of metalloproteinase 3	W96324
mesoderm development candidate 2	AK026606
KIAA0317 gene product	AA625056
KIAA0704 protein	AY008372
ubiquitin-conjugating enzyme E2G 1 (homo	BC002775
seven transmembrane domain protein	AA453425
Human Xq28 cosmid, creatine transporter	EMPTY
adrenergic, alpha-2C-, receptor	AI492607
methyl-CpG binding domain protein 1	NM_002384
diubiquitin	BG290370
ESTs	AA973023
ESTs	AI871106
apolipoprotein E	BF967316
hyaluronoglucosaminidase 2	AU137033
ESTs	AA490264
phosphorylase kinase, beta	AL079451
cell division protein FtsJ	AI784385
inositol 1,4,5-trisphosphate 3-kinase C	AK024596
hypothetical protein FLJ11294	N62430
transcription factor AP-2 gamma (activat	AA513207
actin, gamma 2, smooth muscle, enteric	NM_001615
basigin (OK blood group)	NM_001728
H1 histone family, member X	BE746713
gamma-aminobutyric acid (GABA) A recepto	XM_012441
Human eIF-2-associated p67 homolog mRNA,	EMPTY
bromodomain adjacent to zinc finger doma	AW173580
ESTs, Weakly similar to Similar to phyto	AI276004
Junk, low PCR	EMPTY
cytokine receptor-like molecule 9	BF663575
ESTs	AA805202
paired box gene 8	BE748366
epoxide hydrolase 2, cytoplasmic	AI301066
ESTs	AI167284
tyrosyl-tRNA synthetase	NM_003680
Homo sapiens cDNA: FLJ21914 fis, clone H	AW615335
centrin, EF-hand protein, 3 (CDC31 yeast	AA291295
Homo sapiens mRNA; cDNA DKFZp586P1622 (f	BF002920
homolog of yeast long chain polyunsatura	BF966630
KIAA0909 protein	AB020716
purinergic receptor P2X, ligand-gated io	NM_002562
integrin, alpha L (antigen CD11A (p180),	AC002310
Homo sapiens selectin E (endothelial adh	XM_001578
Human liver glutamate dehydrogenase mRNA	EMPTY
hypothetical protein FLJ22344	AW592194
Homo sapiens regenerating gene type IV m	AA527185
small inducible cytokine subfamily A (Cy	AA127067
deoxyguanosine kinase	AA625191
hypothetical protein FLJ10081	BE045531
HYA22 protein	AI701239
ribosomal protein, large, P1	AA316727

proteasome (prosome, macropain) subunit,	BE395161
DEAD/H (Asp-Glu-Ala-Asp/His) box polypep	AW188131
Junk, low PCR	EMPTY
CDP-diacylglycerol--inositol 3-phosphati	AL540653
S164 protein	BG260998
proprotein convertase subtilisin/kexin t	AW043952
Junk, low PCR	EMPTY
ficolin (collagen/fibrinogen domain-cont	AL161983
Probe hTg737 (polycystic kidney disease,	NM_006531
adaptor-related protein complex 1, sigma	AL522843
Human aminoacylase-1 (ACY1) mRNA, comple	EMPTY
Homo sapiens cDNA: FLJ22288 fis, clone H	AI026907
transcription factor AP-2 alpha (activat	AI360090
midline 1 (Opitz/BBB syndrome)	AF230977
Junk, low PCR	EMPTY
ESTs	AA677934
bone morphogenetic protein 5	NM_021073
Junk, low PCR	EMPTY
ESTs	AI027957
CCCTC-binding factor (zinc finger protei	NM_006565
Homo sapiens cDNA FLJ11606 fis, clone HE	AW971355
zinc finger protein zfp47	AW958059
chromosome 21 open reading frame 4	BE142872
protein-L-isoaspartate (D-aspartate) O-m	AI220702
small inducible cytokine subfamily A (Cy	NM_002988
ATP-binding cassette, sub-family B (MDR/	BE855729
KIAA0809 protein	AK026053
cadherin 13, H-cadherin (heart)	NM_001257
Unknown - Human Control Plate Well H5	EMPTY
Homo sapiens PAK2 mRNA, complete cds	AA992535
Homo sapiens cDNA FLJ10776 fis, clone NT	AI268342
KIAA0217 protein	AL580225
KIAA0998 protein	AV687594
potassium voltage-gated channel, Isk-rel	N27186
collagen, type IX, alpha 2	XM_010599
fibrinogen, B beta polypeptide	AI969333
ESTs	AI203241
Homo sapiens clone 23870 mRNA sequence	AF007128
fetal Alzheimer antigen	AA280072
protease, serine, 4 (trypsin 4, brain)	X71345
Homo sapiens channel-kinase 1 (CHAK1) mR	AI049589
splicing factor, arginine/serine-rich 1	AI362459
deoxythymidylate kinase (thymidylate kin	AW951047
sulfotransferase family, cytosolic, 1A,	L25275
Junk, low PCR	EMPTY
diacylglycerol kinase, gamma (90kD)	NM_001346
Unknown - Human Control Plate Well H10	EMPTY
hypothetical protein FLJ13782	AI380932
histone deacetylase 3	BF591721
dopamine beta-hydroxylase (dopamine beta	Y00096
hypothetical protein DKFZp434O1427	AI521977

Homo sapiens cDNA: FLJ23070 fis, clone L	AA018860
proline-serine-threonine phosphatase int	AF038603
zinc finger protein 268	X78926
Junk, low PCR	EMPTY
SWI/SNF related, matrix associated, acti	BF688178
DKFZP434N014 protein	AA813332
tumor protein p53 (Li-Fraumeni syndrome)	NM_000546
ESTs	AI288937
hypothetical protein FLJ10737	BE798924
zinc finger protein 184 (Kruppel-like)	N80080
protein phosphatase 2 (formerly 2A), reg	AL045885
proteinase 3 (serine proteinase, neutrop	AC004799
imidazoline receptor candidate	AU154312
Empty	EMPTY
sorcin	BE904707
ESTs, Weakly similar to Z136_HUMAN ZINC	AI689087
group-specific component (vitamin D bind	AL532086
GDP-mannose pyrophosphorylase B	AI307331
protein translocation complex beta	AI281953
echinoderm microtubule-associated protei	XM_007243
membrane-spanning 4-domains, subfamily A	L35848
Novel human gene mapping to chomosome 22	AI197929
ESTs	AW970221
ESTs	AA281909
serine (or cysteine) proteinase inhibito	AI249743
KIAA0664 protein	AI925292
Homo sapiens mRNA; cDNA DKFZp586F1322 (f	AL050172
glutathione S-transferase A2	BE795593
cyclin-dependent kinase 5, regulatory su	BE797921
heat shock transcription factor 1	BG473635
sialic acid binding Ig-like lectin 6	AV712452
Empty	EMPTY
Homo sapiens cDNA FLJ13050 fis, clone NT	AI041374
ESTs	AA905409
coagulation factor X	AL521984
Homo sapiens cDNA: FLJ22044 fis, clone H	AI435795
acid phosphatase 5, tartrate resistant	AI871813
potassium large conductance calcium-acti	NM_002247
thrombospondin 2	NM_003247
ESTs	AI493892
hypothetical protein FLJ11175	AI469703
VRK3 for vaccinia related kinase 3	AI566028
diptheria toxin resistance protein requi	AL578050
ESTs	AI027571
calpain 5	XM_006193
ATPase, H+ transporting, lysosomal (vacu	BG252677
myosin, light polypeptide 6, alkali, smo	AW630801
prohibitin	BE621794
hypothetical protein FLJ10509	AK023160
Empty	EMPTY
ESTs, Moderately similar to KIAA0745 pro	AA679589

hypothetical protein FLJ21901	AI870699
BCL2-like 2	D87461
tumor necrosis factor (ligand) superfami	BG272792
E1B-55kDa-associated protein 5	AK021455
hemoglobin, zeta	XM_007935
ovo (Drosophila) homolog-like 1	XM_006508
ESTs	AW104458
ESTs	AI015927
ESTs	H94812
hexabrachion (tenascin C, cytotactin)	X56160
Homo sapiens clone PP1226 unknown mRNA	AA568168
metallothionein 1E (functional)	H72532
ESTs	AI336993
protein tyrosine kinase 9	AA261988
v-kit Hardy-Zuckerman 4 feline sarcoma v	NM_000222
signal transducer and activator of trans	AJ012463
Empty	EMPTY
ATP-binding cassette, sub-family B (MDR/	AI680780
KIAA0591 protein	BF793974
minichromosome maintenance deficient (S.	BG527393
novel protein similar to archaeal, yeast	AW976182
zinc finger protein 266	AU138421
bassoon (presynaptic cytomatrix protein)	AB007894
TAL1 (SCL) interrupting locus	NM_003035
glutaminase	AI005170
hypothetical protein MGC11352	AW339191
thyroid receptor interacting protein 15	AA721403
granulysin	NM_006433
chromosome 8 open reading frame 4	BF965415
Homo sapiens armadillo repeat gene delet	XM_009846
testis enhanced gene transcript (BAX inh	AA932166
Homo sapiens clone 24787 mRNA sequence	AF070526
ATP-binding cassette, sub-family C (CFTR	NM_005688
X-box binding protein 1	AI264133
Empty	EMPTY
ESTs	AW474263
Junk, low PCR	EMPTY
nucleolar phosphoprotein p130	AL553791
ESTs	AA640969
Tax interaction protein 40	AB043634
topoisomerase (DNA) III alpha	XM_008635
signal transducer and activator of trans	NM_005419
tumor protein p53-binding protein, 1	AA521389
hypothetical protein	W74196
Homo sapiens MSH55 gene, partial cds; an	AI004324
N-deacetylase/N-sulfotransferase (hepara	U18932
nucleoporin 50kD	BF056262
adaptor-related protein complex 3, beta	U81504
nucleobindin 1	BF970402
No ID Incyte EST	EMPTY
acyl-Coenzyme A oxidase 3, pristanoyl	R54351

nuclear factor of kappa light polypeptid	NM_003998
Empty	EMPTY
ESTs	AA772405
ESTs	AI144285
hydroxyacyl-Coenzyme A dehydrogenase/3-k	NM_000183
Junk, low PCR	EMPTY
polymerase (RNA) II (DNA directed) polyp	BE615013
forkhead box F1	XM_008078
oligophrenin 1	NM_002547
RAB23, member RAS oncogene family	AA694297
suppressor of Ty (S.cerevisiae) 6 homolo	AU121122
protein kinase PKNbeta	AI741632
protein tyrosine phosphatase, non-recept	NM_002833
hypothetical protein	AI378492
CD164 antigen, sialomucin	BF980976
Junk, low PCR	EMPTY
glycerol-3-phosphate dehydrogenase 1 (so	AI378525
bridging integrator 1	BG250172
adaptor-related protein complex 2, mu 1	BG033362
sorting nexin 1	BF971272
hypothetical protein 384D8_6	BC000473
neuropathy target esterase	NM_006702
ESTs	BG030299
M-phase phosphoprotein 1	L16782
phosphotidylinositol transfer protein	NM_006224
fasciculation and elongation protein zet	AL534445
Junk, low PCR	EMPTY
roundabout (axon guidance receptor, Dros	NM_002941
connective tissue growth factor	AL547439
hypothetical protein, clone 2746033	BE077051
ESTs	BF939077
KIAA0068 protein	AA430381
E2F transcription factor 4, p107/p130-bi	BC000110
KIAA0717 protein	AY009093
frizzled (Drosophila) homolog 1	AA587211
Homo sapiens cDNA FLJ10705 fis, clone NT	BG260122
KIAA0022 gene product	NM_014880
N-ethylmaleimide-sensitive factor attach	BF663204
siah binding protein 1; FBP interacting	AW245149
CDC14 (cell division cycle 14, S. cerevi	XM_001990
peroxisomal acyl-CoA thioesterase	AL528044
Junk, low PCR	EMPTY
phosphoinositide-3-kinase, catalytic, de	U86453
Junk, low PCR	EMPTY
protein tyrosine phosphatase, receptor t	AU133563
ATPase, Na+/K+ transporting, alpha 2 (+)	NM_000702
KIAA0203 gene product	AW771911
Homo sapiens mRNA; cDNA DKFZp564B176 (fr	AA993846
Homo sapiens HTPAP mRNA, complete cds	AL519380
phosphoserine phosphatase	NM_004577
chloride channel 1 , skeletal muscle (Th	Z25753

ribosomal protein S12	AA314429
WNT1 inducible signaling pathway protein	AA861514
KIAA0470 gene product	AB022659
BUB3 (budding uninhibited by benzimidazo	AL544475
37 kDa leucine-rich repeat (LRR) protein	NM_005824
Homo sapiens mRNA; cDNA DKFZp434B044 (fr	AI161099
high-mobility group (nonhistone chromoso	NM_006353
Homo sapiens clone 23645 mRNA sequence	AA469097
Junk, low PCR	EMPTY
disabled (Drosophila) homolog 2 (mitogen	XM_003869
cisplatin resistance associated	XM_001942
tumor protein p53-binding protein, 1	NM_005657
KIAA0543 protein	AB011115
Ras association (RalGDS/AF-6) domain fam	AL579009
ESTs	AA417290
chromosome 22 open reading frame 4	AW957738
KIAA0902 protein	XM_013146
tetraspan transmembrane 4 super family	BE502141
small proline-rich protein 3	NM_005416
insulin-like growth factor binding prote	AA374325
Homo sapiens clone 24583 mRNA sequence	AA251616
catenin (cadherin-associated protein), d	AF062332
ribonuclease, RNase A family, 2 (liver,	NM_002934
cyclin E1	BE536901
craniofacial development protein 1	BG031314
ESTs	T97838
butyrylcholinesterase	AU117425
tropomodulin	NM_003275
nitric oxide synthase 1 (neuronal)	NM_000620
ribonuclease, RNase A family, 4	NM_002937
potassium voltage-gated channel, subfami	AW451609
isovaleryl Coenzyme A dehydrogenase	XM_007611
ESTs	AV699806
hypothetical protein PRO2605	BE675206
EBNA-2 co-activator (100kD)	AU159323
KIAA0404 protein	AB007864
Junk, low PCR	EMPTY
multiple endocrine neoplasia I	AI082242
RAN binding protein 3	AW770120
H2A histone family, member X	AW247312
guanine nucleotide binding protein (G pr	XM_009867
general transcription factor IIIC, polyp	AW014926
KIAA0127 gene product	NM_014755
Homo sapiens KIAA0846 protein (KIAA0846)	XM_010840
cell division cycle 27	XM_008373
NGFI-A binding protein 2 (ERG1 binding p	NM_005967
putative T1/ST2 receptor binding protein	AL547027
LIV-1 protein, estrogen regulated	NM_012319
formyl peptide receptor-like 1	AF054013
hexosaminidase A (alpha polypeptide)	XM_007660
ESTs	AI243708

hypothetical protein DKFZp434E2135	BE504696
paired related homeobox protein	AF061970
interleukin 18 (interferon-gamma-inducin	AV733501
KIAA0673 protein	AW241964
hypothetical protein from clone 24796	R73321
synuclein, gamma (breast cancer-specific	AI863048
activin A receptor, type IB	NM_004302
ribonuclease P (38kD)	AI744554
PTH-responsive osteosarcoma B1 protein	Z24870
ELAV (embryonic lethal, abnormal vision,	BG324385
ESTs	N63894
GS3955 protein	BC002637
Junk, low PCR	EMPTY
ocular albinism 1 (Nettleship-Falls)	NM_000273
exostoses (multiple)-like 2	AK001450
twist (Drosophila) homolog (acrocephalos	X91662
gene from NF2/meningioma region of 22q12	NM_003678
ESTs	AI244273
hypothetical protein DKFZp434M0331	AL137720
neighbor of A-kinase anchoring protein 9	XM_009150
cystathionine-beta-synthase	AL561787
KIAA0415 gene product	AB007875
mutS (E. coli) homolog 5	AJ245661
latrophilin	AB018329
ectonucleotide pyrophosphatase/phosphodi	M57736
Human ionizing radiation resistance conf	EMPTY
HLA-B associated transcript-2	Z15025
suppression of tumorigenicity 14 (colon	AL548113
ESTs, Moderately similar to ALU1_HUMAN A	AI203371
interleukin 17 receptor	AW505520
methyltransferase-like 1	XM_006671
Junk, low PCR	EMPTY
DEAD/H (Asp-Glu-Ala-Asp/His) box polypep	NM_004398
ESTs, Moderately similar to ALU1_HUMAN A	AV728545
Hermansky-Pudlak syndrome	XM_011867
dipeptidylpeptidase IV (CD26, adenosine	XM_002211
centaurin-alpha 2 protein	XM_008289
RAB3A interacting protein (rabin3)-like	AI078157
Homo sapiens PAR5 gene, complete sequenc	AF019618
enhancer of zeste (Drosophila) homolog 1	AB004818
3-hydroxymethyl-3-methylglutaryl-Coenzym	BE543410
nucleolar protein 4	AB015339
carcinoembryonic antigen-related cell ad	AW951446
Human cytoplasmic beta-actin gene, compl	EMPTY
zona pellucida binding protein	AW594602
growth hormone releasing hormone recepto	NM_000823
neurotrophin 5 (neurotrophin 4/5)	AW189053
protocadherin 17	NM_014459
Junk, low PCR	EMPTY
Junk, low PCR	EMPTY
NDRG family, member 4	AB044947

ESTs, Moderately similar to ICE4_HUMAN C	AA041298
zinc finger protein 151 (pHZ-67)	BE336789
fucosyltransferase 3 (galactoside 3(4)-L	NM_000149
ESTs	AA835633
KIAA0764 gene product	BF224432
adaptor-related protein complex 3, sigma	BF032683
solute carrier family 9 (sodium/hydrogen	AL137689
checkpoint suppressor 1	NM_005197
peroxisome proliferative activated recep	AU131142
survival of motor neuron 1, telomeric	AA029190
H.sapiens mRNA for elongations factor Tu	EMPTY
thymopoietin	BF983366
hypothetical protein FLJ10871	AL527030
ESTs	AW241707
Junk, low PCR	EMPTY
Junk, low PCR	EMPTY
claudin 12	AA688200
Junk, low PCR	EMPTY
ESTs	AA584403
mitogen-activated protein kinase kinase	AI752491
fms-related tyrosine kinase 3	NM_004119
hypothetical protein FLJ12598	AW965791
ESTs	AA788764
LIM domain kinase 2	AA010725
vitamin A responsive; cytoskeleton relat	AF070523
Junk, low PCR	EMPTY
Junk, low PCR, low PCR	EMPTY
hematopoietic PBX-interacting protein	XM_001789
Human chaperonin protein (Tcp20) gene co	EMPTY
Junk, low PCR	EMPTY
retinoic acid- and interferon-inducible	BE889460
Junk, low PCR	EMPTY
fibroblast activation protein, alpha	NM_004460
Junk, low PCR	EMPTY
hypothetical protein FLJ13213	BG025116
DNA segment, numerous copies, expressed	AL570791
No ID Incyte EST	EMPTY
asparaginyl-tRNA synthetase	AI458786
KIAA0194 protein	AW732349
Junk, low PCR	EMPTY
hypothetical protein FLJ10853	BF222700
jerky (mouse) homolog	AF072467
diacylglycerol kinase, iota	XM_011554
chromosome 19 open reading frame 3	BF025686
cellular repressor of E1A-stimulated gen	XM_001888
chromosome 11 open reading frame 4	AL138175
Human ADP-ribosylation factor 1 (ARF1) m	EMPTY
KIAA0180 protein	AL135434
capping protein (actin filament) muscle	BC005338
Junk, low PCR	EMPTY
Junk, low PCR	EMPTY

hypothetical protein	L19183
ESTs	AA203252
KIAA0592 protein	AL080183
potassium large conductance calcium-acti	XM_011048
spinocerebellar ataxia 7 (olivopontocere	BG471299
erythropoietin receptor	NM_000121
ESTs	AI924226
transmembrane, prostate androgen induced	AA570597
ATP-binding cassette, sub-family D (ALD)	NM_005164
numb (Drosophila) homolog-like	NM_004756
SHB adaptor protein (a Src homology 2 pr	NM_003028
fibronectin leucine rich transmembrane p	NM_013231
growth arrest-specific 7	NM_005890
Human glyceraldehyde 3-phosphate dehydro	EMPTY
inositol polyphosphate-4-phosphatase, ty	NM_004027
complement component 1, q subcomponent b	BE619838
laminin, beta 1	N35773
cholesteryl ester transfer protein, plas	NM_000078
mutL (E. coli) homolog 1 (colon cancer,	BF910053
hypothetical protein similar to swine ac	AI249145
splicing factor 3a, subunit 3, 60kD	NM_006802
hypothetical protein FLJ10901	AA171760
thyroid hormone receptor interactor 4	BE740238
UV radiation resistance associated gene	AB012958
ESTs	AA284067
ESTs	AW080748
Junk, low PCR	EMPTY
v-akt murine thymoma viral oncogene homo	AI597912
DEAD/H (Asp-Glu-Ala-Asp/His) box binding	BF197063
Junk, low PCR	EMPTY
colony stimulating factor 3 receptor (gr	NM_000760
Empty	EMPTY
Junk, low PCR	EMPTY
KIAA0191 protein	D83776
transforming growth factor, beta 3	X14885
carboxypeptidase D	AU132669
Junk, low PCR	EMPTY
ESTs	AI242026
zinc finger, X-linked, duplicated A	AL034396
ubiquitin specific protease 24	AB028980
flavin containing monooxygenase 1	NM_002021
NADH dehydrogenase (ubiquinone) 1 alpha	BG498093
hypothetical protein PP2447	BE205924
uncharacterized bone marrow protein BM03	AA167536
KIAA0182 protein	BG288870
general transcription factor IIE, polype	AL135122
Homer, neuronal immediate early gene, 3	AL162099
Human glucose transporter pseudogene	AW502150
KIAA0831 protein	AW662896
Empty	EMPTY
phosphoinositide-3-kinase, catalytic, ga	NM_002649

membrane-spanning 4-domains, subfamily A	BG024663
proteasome (prosome, macropain) activato	BC001423
deleted in lymphocytic leukemia, 2	AW978447
Junk, low PCR	EMPTY
hypothetical protein FLJ22002	AA868786
DnaJ (Hsp40) homolog, subfamily C, membe	AW772531
ESTs	AI601252
v-rel avian reticuloendotheliosis viral	NM_006509
protein tyrosine phosphatase, non-recept	AL558909
ESTs	AA191424
olfactory receptor, family 2, subfamily	N73548
mammaglobin 2	NM_002407
myogenin (myogenic factor 4)	XM_001688
p21/Cdc42/Rac1-activated kinase 1 (yeast	AA912798
hect (homologous to the E6-AP (UBE3A) ca	NM_003922
glioma-amplified sequence-41	BF666302
Empty	EMPTY
cholinergic receptor, nicotinic, alpha p	AU099601
dynein, axonemal, light intermediate pol	XM_001609
KIAA0546 protein	AB011118
nuclear factor related to kappa B bindin	NM_006165
peroxisomal acyl-CoA thioesterase	BG331868
Homo sapiens cDNA FLJ20848 fis, clone AD	AI393152
KIAA0555 gene product	NM_014790
ESTs	N62279
isopentenyl-diphosphate delta isomerase	BF698441
chromodomain helicase DNA binding protei	NM_001270
ESTs	AI014510
homeo box D8	AA905044
death-associated protein kinase 2	AA989377
No ID Incyte EST	EMPTY
tryptophan 2,3-dioxygenase	AV653890
Junk, low PCR	EMPTY
protein kinase Njmu-R1	BE782124
Empty	EMPTY
malic enzyme 2, NAD(+)-dependent, mitoch	AW503701
arginyl-tRNA synthetase	XM_003963
procollagen C-endopeptidase enhancer	AF053356
TATA box binding protein (TBP)-associate	AA311701
polycystic kidney disease 2 (autosomal d	BG402881
PDZ-LIM protein mystique	AI057117
choline kinase-like	BE550069
ESTs, Weakly similar to JH0148 nucleolin	AA283717
death-associated protein kinase 3	AI380185
KIAA0530 protein	AB011102
ESTs	AA451709
ESTs, Weakly similar to RTA RAT PROBABLE	AI361281
chromosome 11 open reading frame 13	AL578305
KIAA0157 protein	AA333035
malate dehydrogenase 2, NAD (mitochondri	BE898537
zinc finger protein	AL540227

Junk, low PCR	EMPTY
Empty	EMPTY
cysteine-rich protein 1 (intestinal)	AW328383
EphB4	BG171180
serum-inducible kinase	NM_006622
doublecortin and CaM kinase-like 1	NM_004734
No ID Incyte EST	EMPTY
HDCMA18P protein	AI004357
ribonuclease, RNase A family, 3 (eosinop	NM_002935
Junk, low PCR	EMPTY
ataxin 2 related protein	BE270216
Junk, low PCR	EMPTY
hypothetical protein FLJ10567	AI204090
ESTs	BF515168
protein tyrosine phosphatase, receptor t	AB020704
protocadherin 8	XM_007133
purinergic receptor P2Y, G-protein coupl	NM_002566
Junk, low PCR	EMPTY
HMBA-inducible	NM_006460
Empty	EMPTY
Junk, low PCR	EMPTY
KIAA0512 gene product; ALEX2	NM_014782
sirtuin (silent mating type information	AA156947
LIM homeobox protein 2	AW055373
Junk, low PCR	EMPTY
ESTs, Moderately similar to ALU6_HUMAN A	AA400627
Junk, low PCR	EMPTY
TCR eta	AA258394
RNA binding protein; AT-rich element bin	AF201422
cell cycle progression 2 protein	AL521431
ESTs	AW016707
ESTs	AW469184
ras homolog gene family, member E	W03441
kynureninase (L-kynurenine hydrolase)	AW290980
KIAA0446 gene product	AW248403
Junk, low PCR	EMPTY
CDP-diacylglycerol synthase (phosphatida	AW298414
CGI-43 protein	AA723427
HCNP protein; XPA-binding protein 2	AA811391
glioma pathogenesis-related protein	NM_006851
COP9 subunit 6 (MOV34 homolog, 34 kD)	BE390564
hect domain and RLD 3	NM_014606
hypothetical protein FLJ11238	N51263
superiorcervical ganglia, neural specifi	AL535825
No ID Incyte EST	EMPTY
Homo sapiens clone 23903 mRNA sequence	AF035281
CD163 antigen	Z22971
Human chromosome 5q13.1 clone 5G8 mRNA	AA676310
Junk, low PCR	EMPTY
kininogen	AL532524
zinc metalloproteinase, STE24 (yeast, ho	AW192807

BH-protocadherin (brain-heart)	AB006757
GrpE-like protein cochaperone	BG290183
solute carrier family 25 (mitochondrial	BG491884
protein disulfide isomerase	NM_006849
Homo sapiens cDNA FLJ12797 fis, clone NT	AA846091
No ID Incyte EST	EMPTY
trinucleotide repeat containing 3	AW769819
KIAA0255 gene product	AI418892
killer cell lectin-like receptor subfami	NM_002260
ESTs	AA780077
nuclear receptor interacting protein 1	AF127577
aminolevulinate, delta-, synthase 1	BF969214
caspase 10, apoptosis-related cysteine p	NM_001230
lamin B receptor	NM_002296
matrilin 2	NM_030583
ESTs	BE537620
protein phosphatase 2, regulatory subuni	NM_006246
matrix metalloproteinase 11 (stromelysin	AI189375
ELAV (embryonic lethal, abnormal vision,	XM_005409
low density lipoprotein-related protein	NM_002332
serum/glucocorticoid regulated kinase	BC001263
gamma-aminobutyric acid (GABA) A recepto	BG002261
ESTs	AA847649
hypothetical protein FLJ12878	AU144289
matrin 3	BG179511
KIAA0274 gene product	NM_014845
malignant cell expression-enhanced gene/	BF984370
ESTs, Moderately similar to AF116721 89	N44825
Junk, low PCR	EMPTY
laminin receptor 1 (67kD, ribosomal prot	NM_002295
sodium channel, voltage-gated, type IX,	NM_002977
carboxypeptidase Z	NM_003652
centrin, EF-hand protein, 2	AI694310
Junk, low PCR	EMPTY
myosin, light polypeptide 5, regulatory	NM_002477
archain 1	NM_001655
protease, serine, 7 (enterokinase)	NM_002772
NADPH oxidase 1	AI791894
RNA (guanine-7-) methyltransferase	NM_003799
glycerol kinase	NM_000167
hypothetical protein FLJ10461	AI014927
ESTs	AA766638
multimerin	NM_007351
ubiquitin specific protease 5 (isopeptid	BE019497
Junk, low PCR	EMPTY
ESTs	AL565418
folate receptor 2 (fetal)	XM_005996
drebrin 1	NM_004395
target of myb1 (chicken) homolog	Z82244
carboxylesterase 1 (monocyte/macrophage	NM_001266
mitogen-activated protein kinase 8 inter	U79261

small glutamine-rich tetratricopeptide r	BG423904
glutamic-oxaloacetic transaminase 1, sol	XM_005783
B-cell CLL/lymphoma 6 (zinc finger prote	NM_001706
SMA5	AI278613
calumenin	BG254319
NADH dehydrogenase (ubiquinone) flavopro	AW051350
brain abundant, membrane attached signal	BG398345
solute carrier family 7 (cationic amino	BF058129
ESTs	AA721182
EphB3	NM_004443
SH3-domain GRB2-like 1	BE563866
protein kinase C, mu	NM_002742
ESTs	AI953049
BCL2-related protein A1	BF677029
cortistatin	AI022128
Bicaudal D (Drosophila) homolog 1	NM_001714
KIAA0121 gene product	D50911
Homo sapiens cDNA FLJ10169 fis, clone HE	BG259789
ESTs	AA844124
Wilms' tumour 1-associating protein	W78201
complement component 4B	NM_000592
transcription factor AP-2 gamma (activat	NM_003222
ATPase, H+ transporting, lysosomal (vacu	BE798912
Src-like-adapter	U44403
coatomer protein complex, subunit alpha	AL043506
ESTs	AA706272
ESTs	AI801190
KIAA0657 protein	BG031222
Junk, low PCR	EMPTY
nicotinamide N-methyltransferase	NM_006169
ESTs	AI026723
No ID Incyte EST	EMPTY
aldolase C, fructose-bisphosphate	T03444
mitogen-activated protein kinase 1	Z11694
mannosidase, beta A, lysosomal	XM_003399
fibrinogen, A alpha polypeptide	AL564557
chromosome 4 open reading frame 1	BE883695
piccolo (presynaptic cytomatrix protein)	AB011131
transferrin	NM_001063
polycystic kidney disease 1 (autosomal d	AW963270
claudin 1	AF101051
trophinin	BE894856
pituitary tumor-transforming 1	BE893878
Human liver mRNA fragment DNA binding pr	EMPTY
hypothetical protein FLJ20170	AL132874
modulator of apoptosis 1	AI888832
KIAA0355 gene product	NM_014686
ESTs	AA401425
ESTs	BE552115
LIM domain only 2 (rhombotin-like 1)	NM_005574
ADP-ribosylation factor 4	BF208090

ESTs, Weakly similar to ALU8_HUMAN ALU S	AA417878
putative oncogene protein hlc14-06-p	AL583286
Homo sapiens cDNA FLJ13835 fis, clone TH	AI913337
BCL2/adenovirus E1B 19kD-interacting pro	BG395997
hypothetical protein	AL529877
hypothetical protein	BG283735
airway trypsin-like protease	XM_011185
ribosomal protein S6 kinase, 70kD, polyp	NM_003952
glutamine-fructose-6-phosphate transamin	AK001242
cadherin 17, LI cadherin (liver-intestin	NM_004063
Human AMP deaminase (AMPD2) mRNA	EMPTY
Homo sapiens cDNA: FLJ22554 fis, clone H	AA465488
chondroitin sulfate proteoglycan 2 (vers	AI361996
Junk, low PCR	EMPTY
ESTs, Highly similar to A38712 fibrillar	AI199605
ESTs	W16688
high-mobility group (nonhistone chromoso	BG250825
alkylglycerone phosphate synthase	NM_003659
guanine nucleotide binding protein (G pr	AF092129
glutathione S-transferase theta 1	AI281103
exosome component Rrp41	BC002777
amiloride-sensitive cation channel 2, ne	XM_006754
p53 regulated PA26 nuclear protein	BF510689
UDP-N-acteylglucosamine pyrophosphorylas	AL520091
Junk, low PCR	EMPTY
serine (or cysteine) proteinase inhibito	AV692071
Lutheran blood group (Auberger b antigen	BE392568
zinc finger protein 230	XM_012865
Human acidic ribosomal phosphoprotein P0	EMPTY
hypothetical protein DKFZp761C169	AW302393
myogenic factor 3	BC000353
transcription elongation factor B (SIII)	BG334963
Homo sapiens 14q32 Jagged2 gene, complet	AA528133
ESTs	AW956498
BMX non-receptor tyrosine kinase	NM_001721
lipin 1	D80010
ESTs	AA854852
complement component 1, s subcomponent	NM_001734
KIAA1249 protein	AW513835
peptidylprolyl isomerase B (cyclophilin	BE868117
p53-inducible ribonucleotide reductase s	AK023605
hypothetical protein 24636	AI066576
Junk, low PCR	EMPTY
oxidative 3 alpha hydroxysteroid dehydro	AI658732
potassium intermediate/small conductance	NM_002250
double C2-like domains, alpha	BE646046
Human mRNA for histidyl-tRNA synthetase	EMPTY
ESTs, Weakly similar to T00377 KIAA0642	AI685431
ESTs, Weakly similar to CYA2_HUMAN ADENY	AA044828
Junk, low PCR	EMPTY
Homo sapiens mRNA for KIAA1750 protein,	AA948679

No ID Incyte EST	EMPTY
alcohol dehydrogenase 1 (class I), alpha	NM_000667
serine/threonine kinase 12	BE253249
KIAA0751 gene product	BF510067
zinc finger protein 7 (KOX 4, clone HF.1	NM_003416
Homo sapiens glutamate rich WD repeat pr	AW300643
homeo box D13	XM_002542
inhibitor of DNA binding 4, dominant neg	BF940116
hypothetical protein	BE739607
hyaluronoglucosaminidase 1	AL577920
MAGUK protein p55T; Protein Associated w	NM_016447
nidogen 2	W70102
inositol polyphosphate-5-phosphatase, 75	AK022846
Human mRNA for eukaryotic initiation fac	EMPTY
ESTs	BF510581
ESTs	AA558906
transmembrane 9 superfamily member 1	BF094556
TcD37 homolog	AW300687
ESTs	AA926899
prolyl endopeptidase	BF724523
four and a half LIM domains 1	BE250452
Homo sapiens cDNA FLJ10264 fis, clone HE	AW294133
parathyroid hormone-like hormone	AI591151
prothymosin, alpha (gene sequence 28)	AA708613
zinc finger protein 187	Z11773
MD-2 protein	AA825754
cullin 4A	AI638597
diacylglycerol kinase, theta (110kD)	NM_001347
plakophilin 2	NM_004572
Junk, low PCR, low PCR	EMPTY
LIM domain kinase 1	NM_002314
Human alpha-2-macroglobulin mRNA, comple	EMPTY
hypothetical protein DKFZp762K2015	W74507
RAN binding protein 9	AI190727
SET binding factor 1	BE737332
ESTs	AA913909
ESTs	AA004443
protein S (alpha)	NM_000313
Junk, low PCR	EMPTY
solute carrier family 6 (neurotransmitte	AA203542
growth arrest-specific 6	NM_000820
hypothetical protein MGC8407	AW128954
choroideremia (Rab escort protein 1)	BG291499
KIAA0955 protein	AA099277
KIAA0143 protein	AA031914
Junk, low PCR	EMPTY
keratocan	XM_006901
KIAA0726 gene product	NM_014718
retinoid X receptor, alpha	AF052092
Arabadopsis7-250	EMPTY
Junk, low PCR	EMPTY

ESTs	U55983
myosin, light polypeptide 2, regulatory,	BF790510
hypothetical protein FLJ13287	AU155156
Homo sapiens colon cancer antigen NY-CO-	AL043594
thyroid stimulating hormone receptor	S82807
Junk, low PCR	EMPTY
ESTs	AA808570
ESTs	AI589047
hypothetical protein FLJ21065	AI377637
activating transcription factor 7	BG325826
patched related protein translocated in	AI366859
ligase III, DNA, ATP-dependent	XM_008296
No ID Incyte EST	EMPTY
matrix Gla protein	BF668572
cytochrome P450, subfamily XVII (steroid	AV704387
ribonuclease HI, large subunit	BE045964
Arabadopsis9-250	EMPTY
aldehyde dehydrogenase 1 family, member	NM_000693
PR domain containing 10	AI610587
gp25L2 protein	BG163409
zinc finger protein 331	BE178002
KIAA0356 gene product	AJ002220
KIAA0124 protein	BG178424
ras homolog gene family, member C	BE876672
ESTs	BF879823
ESTs, Weakly similar to AF151859 1 CGI-1	AW275800
GK003 protein	AF226046
Junk, low PCR	EMPTY
ESTs	AW964490
single-minded (Drosophila) homolog 2	XM_009755
solute carrier family 28 (sodium-coupled	U62966
cholinergic receptor, nicotinic, alpha p	NM_000742
biotinidase	BG252311
glutathione S-transferase M3 (brain)	AL553432
Empty	EMPTY
basonuclin	NM_001717
Homo sapiens cDNA FLJ11801 fis, clone HE	AI884571
steroidogenic acute regulatory protein	AV710522
Junk, low PCR	EMPTY
U4/U6-associated RNA splicing factor	NM_004698
amyotrophic lateral sclerosis 2 (juvenil	AB038951
methylthioadenosine phosphorylase	XM_011800
hypothetical protein MGC2803	AL523612
ESTs, Weakly similar to unnamed protein	AI608836
ERO1 (S. cerevisiae)-like	BG104533
Junk, low PCR	EMPTY
iron-responsive element binding protein	BF002434
arachidonate 15-lipoxygenase, second typ	NM_001141
transforming growth factor, beta recepto	XM_005591
chromobox homolog 6	AI986443
Homo sapiens clone 24775 mRNA sequence	AA402981

5-methyltetrahydrofolate-homocysteine me	U73338
Empty	EMPTY
Homo sapiens clones 24622 and 24623 mRNA	AF070590
choline/ethanolaminephosphotransferase	AA588437
angiotensin receptor 1	NM_000685
Homo sapiens mRNA full length insert cDN	AI023426
exostoses (multiple) 2	AI869325
v-raf murine sarcoma 3611 viral oncogene	NM_001654
ubiquitination factor E4A (homologous to	BF691464
v-maf musculoaponeurotic fibrosarcoma (a	AI743068
ESTs	AA425325
ESTs	N92599
Junk, low PCR	EMPTY
Homo sapiens mRNA; cDNA DKFZp434J0617 (f	AI039472
amyloid beta (A4) precursor protein-bind	AI494375
SET translocation (myeloid leukemia-asso	AW591548
microsomal glutathione S-transferase 3	AV705554
collagen, type VI, alpha 2	AI635289
leucine-rich protein mRNA	AI651837
Empty	EMPTY
Homo sapiens mRNA; cDNA DKFZp564K0322 (f	BE463658
ESTs	AI061121
lectin, galactoside-binding, soluble, 8	AF074002
cyclin D binding Myb-like transcription	AU145780
DEAD/H (Asp-Glu-Ala-Asp/His) box polypep	BE409685
potassium voltage-gated channel, shaker-	NM_002234
cyclin-dependent kinase inhibitor 1C (p5	AI088356
ESTs	AA835640
Junk, low PCR	EMPTY
KIAA1488 protein	AA169411
protein kinase C, gamma	AF345987
cadherin 16, KSP-cadherin	AI733487
eukaryotic translation initiation factor	BG484643
ESTs, Highly similar to NAALADase II pro	AA488703
hepatocellular carcinoma-associated anti	AI992137
glucosaminyl (N-acetyl) transferase 3, m	NM_004751
mitogen-activated protein kinase-activat	NM_004635
Empty	EMPTY
ESTs, Weakly similar to cDNA EST CEESN66	AW968810
low molecular mass ubiquinone-binding pr	AA486825
far upstream element (FUSE) binding prot	U69127
ESTs	AW015010
hypothetical protein MGC2683	BE741777
huntingtin-associated protein interactin	NM_003947
runt-related transcription factor 1 (acu	D43968
ESTs	AA737568
ESTs	BF056901
KIAA1450 protein	AU116869
interferon gamma receptor 1	AL050337
endosulfine alpha	AA132749
nuclear factor (erythroid-derived 2)-lik	NM_003204

interleukin 7	NM_000880
acid sphingomyelinase-like phosphodieste	AI335007
tripeptidyl peptidase II	NM_003291
ubiquitin-conjugating enzyme E2E 1 (homo	BF103707
Human mRNA for unknown product, partial	D29810
hypothetical protein MGC10848	AA134751
methionine adenosyltransferase II, alpha	F07456
ESTs	AA400519
TGF(beta)-induced transcription factor 2	AF055012
paired basic amino acid cleaving system	NM_002570
serine (or cysteine) proteinase inhibito	AI133613
BTB and CNC homology 1, basic leucine zi	AI830904
polymeric immunoglobulin receptor	AW850633
early growth response 1	AL553329
EST	H96791
KIAA1481 protein	AB040914
cartilage associated protein	BE742024
dihydrofolate reductase	AU130196
death receptor 6	XM_004585
potassium voltage-gated channel, subfami	AL050404
SWI/SNF related, matrix associated, acti	BF221495
growth factor receptor-bound protein 14	NM_004490
CDC16 (cell division cycle 16, S. cerevi	NM_003903
ESTs, Weakly similar to contains similar	AA489126
ATPase, Class I, type 8B, member 1	NM_005603
ESTs	AA282067
NADH dehydrogenase (ubiquinone) 1 alpha	AV647700
KIAA0395 protein	AL022394
small nuclear ribonucleoprotein polypept	BE535667
cytochrome P450, subfamily IIJ (arachido	BE696195
Junk, low PCR	EMPTY
histone deacetylase 1	AL559849
NADH dehydrogenase (ubiquinone) 1 alpha	AW515946
mannan-binding lectin serine protease 2	AB033742
EGF-like-domain, multiple 4	AV751780
tubby like protein 1	XM_004287
peroxisome proliferative activated recep	AL022721
deoxythymidylate kinase (thymidylate kin	BF312526
KIAA1535 protein	AW243139
solute carrier family 9 (sodium/hydrogen	AC005600
nuclear receptor subfamily 0, group B, m	AI801227
ESTs	BE218905
glutathione peroxidase 1	AU133229
Junk, low PCR	EMPTY
protein predicted by clone 23733	AW958081
IQ motif containing GTPase activating pr	AI719158
TGF beta receptor associated protein -1	NM_004257
siah binding protein 1; FBP interacting	BE797675
ZYG homolog	NM_006336
Junk, low PCR	EMPTY
chromosome 21 open reading frame 5	AW070676

No ID Incyte EST	EMPTY
No ID Incyte EST	EMPTY
annexin A2	BF084103
matrix metalloproteinase 10 (stromelysin	NM_002425
DNA (cytosine-5-)-methyltransferase 3 be	AK001191
ATP-binding cassette, sub-family G (WHIT	AB056867
phosphorylase, glycogen; brain	NM_002862
diptheria toxin resistance protein requi	AL530308
ESTs	AI732582
threonyl-tRNA synthetase	BC000517
Junk, low PCR	EMPTY
GDNF family receptor alpha 1	NM_005264
BRCA1 associated protein	AW804509
Junk, low PCR	EMPTY
syntaxin binding protein 1	NM_003165
aquaporin 4	BG483248
splicing factor 3b, subunit 2, 145kD	BE799382
tyrosylprotein sulfotransferase 2	AI139748
hypothetical protein P15-2	AU123481
Homo sapiens hepatocellular carcinoma-as	AF257175
complement component 8, alpha polypeptid	NM_000562
ESTs	AA976388
dolichyl-phosphate mannosyltransferase p	AF061729
cathepsin Z	AI913006
protein tyrosine phosphatase, receptor t	NM_002838
Homo sapiens (clone B3B3E13) Huntington'	BG539199
hypothetical protein	AA885882
CD79A antigen (immunoglobulin-associated	AL560625
ESTs, Weakly similar to ALU1_HUMAN ALU S	AA459510
Junk, low PCR	EMPTY
phosphoenolpyruvate carboxykinase 2 (mit	XM_007298
coactivator-associated arginine methyltr	AF055027
general transcription factor IIF, polype	BG032043
egf-like module containing, mucin-like,	NM_001974
regulator of nonsense transcripts 1	D86988
ESTs	AI003801
Junk, low PCR	EMPTY
polycystic kidney disease 2 (autosomal d	BE311497
iduronate 2-sulfatase (Hunter syndrome)	AW896303
v-maf musculoaponeurotic fibrosarcoma (a	AF055376
KIAA0492 protein	AB007961
hypothetical protein similar to mouse H	AW439814
neutrophil cytosolic factor 2 (65kD, chr	XM_002200
monoamine oxidase A	M69226
ESTs	N62301
Rho guanine nucleotide exchange factor (	NM_005435
Junk, low PCR	EMPTY
carboxypeptidase A3 (mast cell)	NM_001870
adenylosuccinate lyase	BE795635
dopamine receptor D2	S62137
Junk, low PCR	EMPTY

CD1A antigen, a polypeptide	M28825
Wiskott-Aldrich syndrome (eczema-thrombo	AF196970
ESTs	AA749317
zinc finger protein 83 (HPF1)	AI873839
KIAA0893 protein	R58914
putative protein	AW071792
Junk, low PCR	EMPTY
Xq28, 2000bp sequence contg. ORF	BE298183
protein kinase C, alpha binding protein	AU159268
KIAA0056 protein	AK025549
Human mRNA for alpha-catenin, complete c	EMPTY
serine/threonine kinase 9	NM_003159
ADP-ribosylation factor-like 3	AU098377
Homo sapiens mRNA full length insert cDN	BE858194
vaccinia related kinase 2	AL549153
protein phosphatase 2, regulatory subuni	BF971966
ring finger protein 2	AI242656
KIAA0112 protein; homolog of yeast ribos	AI380567
small nuclear ribonucleoprotein polypept	AI493442
Opa-interacting protein 5	BE045993
sulfotransferase, estrogen-preferring	AA448993
serine/threonine protein kinase MASK	XM_010457
Homo sapiens mRNA; cDNA DKFZp762O2215 (f	AA804873
H3 histone family, member K	NM_003536
sulfotransferase family, cytosolic, 2A,	AV707067
cyclin-dependent kinase (CDC2-like) 10	BF969521
KIAA1357 protein	BF591161
H3 histone, family 3B (H3.3B)	BG338936
Human guanine nucleotide-binding protein	EMPTY
Junk, low PCR	EMPTY
DKFZP566C134 protein	AF004292
ESTs	AI803694
histatin 3	AA376718
retinoblastoma-binding protein 8	BE891984
Homo sapiens mRNA; cDNA DKFZp586J1119 (f	AL136919
Junk, low PCR	EMPTY
chromosome 9 open reading frame 9	BE548310
platelet-derived growth factor alpha pol	AA525143
collapsin response mediator protein 1	BF343909
COBW-like protein	BE857130
hypothetical protein DKFZp434H247	AL137304
solute carrier family 1 (glutamate trans	NM_006671
H2B histone family, member Q	BE245642
procollagen-proline, 2-oxoglutarate 4-di	AL574109
guanine nucleotide binding protein (G pr	BG395645
protocadherin gamma subfamily B, 6	XM_011235
Human squalene synthetase (ERG9) mRNA, c	EMPTY
polymerase (RNA) III (DNA directed) (32k	NM_006467
neuronal cell adhesion molecule	BG153395
KIAA1545 protein	AI475804
core-binding factor, runt domain, alpha	NM_004349

stromal cell derived factor receptor 1	AI264293
Junk, low PCR	EMPTY
mannose-6-phosphate receptor (cation dep	AU152962
ESTs	AA627448
diaphorase (NADH/NADPH) (cytochrome b-5	AA921756
Junk, low PCR	EMPTY
hypothetical protein FLJ23548	AI050751
protein phosphatase 3 (formerly 2B), cat	AL353950
Junk, low PCR	EMPTY
trinucleotide repeat containing 5	N31024
slit (Drosophila) homolog 2	BG023818
Junk, low PCR, low PCR	EMPTY
surfeit 2	BE781163
Human mRNA for flavoprotein subunit of c	EMPTY
ATPase, H+ transporting, lysosomal (vacu	AL527529
corticotropin releasing hormone-binding	AV646516
ESTs	N33786
ubiquitin specific protease 9, Y chromos	Y13618
ADP-ribosylation factor related protein	BF435047
Incyte EST	NO ID
transcription factor-like 1	AL573798
ESTs	AA114849
poly(A)-binding protein, nuclear 1	AI637772
Junk, low PCR	EMPTY
ESTs	N27406
KIAA1449 protein	AK025513
RecQ protein-like 4	AB026546
protein phosphatase 1D magnesium-depende	NM_003620
No ID Incyte EST	EMPTY
hypothetical protein FLJ22195	AF052106
angio-associated, migratory cell protein	AL542809
Human translational initiation factor 2	EMPTY
biliverdin reductase A	AI765830
internexin neuronal intermediate filamen	BE781432
EST	AI032307
ATPase, Ca++ transporting, cardiac muscl	NM_004320
amyloid beta precursor protein-binding p	NM_003905
ESTs, Weakly similar to 2004399A chromos	BE043098
acetylcholinesterase (YT blood group)	NM_015831
ESTs	AI125795
matrix metalloproteinase-like 1	AJ003144
solute carrier family 8 (sodium/calcium	NM_021097
lipoyltransferase	NM_015929
PTD013 protein	AW134621
sulfotransferase family, cytosolic, 1A,	BE745439
FYN oncogene related to SRC, FGR, YES	Z97989
interferon-induced protein 75, 52kD	AI796501
nephronophthisis 1 (juvenile)	NM_000272
phospholipase A2, group VI (cytosolic, c	AF102988
Homo sapiens protein tyrosine kinase (Sy	EMPTY
acyl-Coenzyme A dehydrogenase, short/bra	AV645726

putative lymphocyte G0/G1 switch gene	BE873759
ESTs	AA599585
ELAV (embryonic lethal, abnormal vision,	NM_001420
myosin, light polypeptide kinase	AW951177
Junk, low PCR	EMPTY
cathepsin L2	AB001928
ESTs	AA776940
integrin, alpha 2b (platelet glycoprotei	NM_000419
cysteine dioxygenase, type I	NM_001801
ATP-dependent RNA helicase	AW277045
ESTs	BF966000
Homo sapiens clone 24803 mRNA sequence	AF070628
secretory carrier membrane protein 2	AA018203
phosphodiesterase 8B	AW204224
Junk, low PCR, low PCR	EMPTY
Junk, low PCR	EMPTY
Arabadopsis1-250	EMPTY
melanoma antigen, family A, 9	NM_005365
solute carrier family 20 (phosphate tran	AL581504
adaptor-related protein complex 1, gamma	NM_003917
deiodinase, iodothyronine, type II	AF007144
Junk, low PCR	EMPTY
Homo sapiens MSTP031 mRNA, complete cds	AI191090
Junk, low PCR	EMPTY
Junk, low PCR	EMPTY
grancalcin	BG545676
zinc finger protein 200	NM_003454
Junk, low PCR	EMPTY
putative helicase RUVBL	BF440027
hepatocyte growth factor-regulated tyros	AK026523
hypothetical protein LOC55565	XM_008084
ubiquitin-conjugating enzyme E2A (RAD6 h	AL545489
cystatin F (leukocystatin)	AW467354
KIAA0727 protein	N26257
Arabadopsis3-250	EMPTY
potassium voltage-gated channel, Shab-re	NM_004975
trinucleotide repeat containing 1	AV703020
defensin, alpha 1, myeloid-related seque	M26602
Junk, low PCR	EMPTY
Junk, low PCR	EMPTY
Junk, low PCR	EMPTY
Junk, low PCR	EMPTY
Homo sapiens cDNA: FLJ23227 fis, clone C	AF052138
CBF1 interacting corepressor	AW150369
high-mobility group (nonhistone chromoso	BG529476
ESTs	AA992398
DNA (cytosine-5-)-methyltransferase 3 al	BE644774
butyrate response factor 2 (EGF-response	AW418782
sperm associated antigen 1	AF311312
keratin 16 (focal non-epidermolytic palm	AU147404
Junk, low PCR	EMPTY

f-box and leucine-rich repeat protein 5	AW262944
Arabadopsis5-250	EMPTY
afamin	NM_001133
protein phosphatase 3 (formerly 2B), cat	NM_021132
VPS28 protein	AF067420
Junk, low PCR	EMPTY
related RAS viral (r-ras) oncogene homol	BF591022
KIAA0732 protein	AI423441
KIAA0320 protein	AB002318
hypothetical protein PRO2849	AW001045
Fc fragment of IgG, low affinity IIIb, r	J04162
KIAA0379 protein	AB002377
Homo sapiens cDNA FLJ11946 fis, clone HE	AK022008
Junk, low PCR	EMPTY
six transmembrane epithelial antigen of	AF186249
homolog of Yeast RRP4 (ribosomal RNA pro	BF974190
hydroxysteroid (17-beta) dehydrogenase 2	NM_002153
Junk, low PCR	EMPTY
cyclin D1 (PRAD1: parathyroid adenomatos	AI580948
Empty	EMPTY
casein, alpha	NM_001890
Junk, low PCR	EMPTY
glutamate dehydrogenase 1	NM_005271
putative transmembrane protein	NM_012342
Junk, low PCR	EMPTY
ESTs, Weakly similar to T12682 hypotheti	AW952320
coagulation factor V (proaccelerin, labi	NM_000130
hypothetical protein FLJ21820	AA843690
interleukin-1 receptor-associated kinase	BE537931
calpain 4, small subunit (30K)	BG419439
mannosidase, alpha, class 1A, member 2	AW021115
hypothetical protein FLJ10392	AB033038
multiple endocrine neoplasia I	Y12338
nuclear transcription factor Y, alpha	XM_011362
RNA-binding protein regulatory subunit	BF663649
peroxisomal biogenesis factor 11A	AU134363
RNA, U transporter 1	AI983683
Empty	EMPTY
solute carrier family 18 (vesicular mono	L14269
fibroblast growth factor 9 (glia-activat	AI869879
macrophage receptor with collagenous str	NM_006770
cytochrome P450, subfamily XXVIIB (25-hy	NM_000785
regulatory factor X, 5 (influences HLA c	AW337740
Junk, low PCR	EMPTY
receptor (TNFRSF)-interacting serine-thr	NM_003804
myosin IXA	AU149468
laminin, gamma 1 (formerly LAMB2)	NM_002293
brain-specific angiogenesis inhibitor 2	AI498960
ESTs	AW976204
ESTs	AI433866
aquaporin 7	AW779701

solute carrier family 3 (cystine, dibasi	BG399771
thyroid hormone receptor coactivating pr	AW020536
small inducible cytokine subfamily A (Cy	AC004382
KIAA0874 protein	AB020681
Empty	EMPTY
peripheral myelin protein 2	XM_005095
small proline-rich protein 2A	BE714149
leucine-rich neuronal protein	XM_004694
cysteinyl-tRNA synthetase	BG386134
GS2 gene	BG106467
glycine cleavage system protein H (amino	BG032241
Junk, low PCR	EMPTY
Junk, low PCR	EMPTY
glutamic-pyruvate transaminase (alanine	NM_005309
c-fos induced growth factor (vascular en	NM_004469
KIAA1039 protein	AA714570
glucocorticoid receptor DNA binding fact	AI275597
protein tyrosine phosphatase, receptor t	AA398679
tumor rejection antigen (gp96) 1	BG327949
KIAA0672 gene product	AI922429
Junk, low PCR	EMPTY
protein tyrosine phosphatase, receptor t	AA281524
Junk, low PCR	EMPTY
myosin, heavy polypeptide 11, smooth mus	NM_022870
ESTs	AW021108
hypothetical protein FLJ20477	AW300554
CD44 antigen (homing function and Indian	M24915
insulin receptor	NM_000208
Homo sapiens cDNA FLJ14241 fis, clone OV	AA410508
Junk, low PCR	EMPTY
glutamate receptor, metabotropic 8	XM_011620
hypothetical protein FLJ10874	BF594485
Junk, low PCR	EMPTY
Junk, low PCR	EMPTY
membrane-spanning 4-domains, subfamily A	XM_006275
RNA binding motif protein, X chromosome	BG167309
NAG-7 protein	XM_003180
Junk, low PCR	EMPTY
G protein-coupled receptor kinase 6	NM_002082
aldo-keto reductase family 1, member C1	BE786113
ESTs, Weakly similar to ubiquitous TPR m	AI656717
interleukin 16 (lymphocyte chemoattracta	AI652705
hypothetical protein	BF447431
hypothetical protein FLJ22757	AW449018
NO ID Incyte EST	EMPTY
thioredoxin	AV763087
CGI-53 protein	AU150645
small inducible cytokine subfamily A (Cy	AC004382
CAT56 protein	AL583224
ESTs	H98083
Junk, low PCR	EMPTY

ESTs, Weakly similar to organic anion tr	AA033971
trans-Golgi network protein (46, 48, 51k	XM_002569
MAD (mothers against decapentaplegic, Dr	NM_005904
solute carrier family 23 (nucleobase tra	AW016678
chromosome 19 open reading frame 3	AW409924
capping protein (actin filament) muscle	BC000144
integrin, alpha 3 (antigen CD49C, alpha	XM_008431
dihydrofolate reductase	NM_000791
nuclear factor (erythroid-derived 2), 45	NM_006163
hypothetical protein KIAA1165	R82692
ESTs	AA778938
calpain 9 (nCL-4)	NM_006615
cathepsin K (pycnodysostosis)	AL544409
protein kinase C, nu	AW515068
cytokeratin 20	X73501
serum response factor (c-fos serum respo	XM_011430
ESTs, Weakly similar to DYLX_HUMAN CYTOP	AA405485
deafness, autosomal dominant 5	NM_004403
hypothetical protein FLJ10600	AI811688
butyrophilin, subfamily 3, member A1	AI680403
thrombospondin 3	BF898431
N-acetylglucosamine-1-phosphodiester alp	BF594186
alcohol dehydrogenase 2 (class I), beta	AV681547
transient receptor potential channel 3	NM_003305
KIAA0367 protein	AB002365
solute carrier family 12, (potassium-chl	AL162458
6-pyruvoyl-tetrahydropterin synthase/dim	AV702374
ESTs, Weakly similar to T12492 hypotheti	N51980
ubinuclein 1	AA157291
Junk, low PCR	EMPTY
proteasome (prosome, macropain) subunit,	BG386309
albumin	W84549
activity-regulated cytoskeleton-associat	AF193421
aconitase 1, soluble	AI762790
hypothetical protein FLJ12270	NM_024673
5' nucleotidase (CD73)	AW072424
hypothetical protein	AA400872
single-stranded DNA-binding protein	BE220360
cell division cycle 4-like	M83822
Homo sapiens Cri-du-chat region mRNA, cl	U52827
chromosome 2 open reading frame 1	AI312130
thyroid hormone receptor interactor 7	AU144496
ketohexokinase (fructokinase)	NM_006488
C9orf10 protein	W96064
DnaJ (Hsp40) homolog, subfamily A, membe	Y13350
hypothetical protein from EUROIMAGE 1669	AA446469
hypothetical protein, clone 24751	AI087056
potassium voltage-gated channel, delayed	BC004148
proteasome (prosome, macropain) 26S subu	AU118540
KIAA1018 protein	BF476140
lymphocyte antigen 75	BG290654

caspase 2, apoptosis-related cysteine pr	XM_004692
hypothetical protein FLJ13956	AA292417
Junk, low PCR	EMPTY
hypothetical protein MGC10764	AW469061
solute carrier family 14 (urea transport	NM_007163
deafness, autosomal dominant 5	AL035855
protease, serine, 16 (thymus)	AA580758
mitogen-activated protein kinase kinase	NM_004672
splicing factor 3a, subunit 1, 120kD	BE894372
uteroglobin	U01102
cytochrome c oxidase subunit VIIb	AV695162
TATA box binding protein (TBP)-associate	XM_007831
hypothetical protein	AL532462
FLJ00005 protein	AI669556
ribosomal protein S28	AW102850
kallikrein 3, (prostate specific antigen	M24543
neurotrimin	W52908
KIAA0342 gene product	AB002340
ras homolog gene family, member B	AL523759
Homo sapiens cDNA: FLJ20882 fis, clone A	AW574632
Lsm1 protein	NM_014462
KIAA1324 protein	AI683001
gap junction protein, alpha 8, 50kD (con	XM_001660
major histocompatibility complex, class	AI249268
potassium inwardly-rectifying channel, s	NM_000890
nuclear receptor subfamily 2, group C, m	R54467
paternally expressed 3	H13563
sarcoglycan, alpha (50kD dystrophin-asso	NM_000023
Human Xq28 cosmid, creatine transporter	EMPTY
pM5 protein	BE910130
trinucleotide repeat containing 11 (THR-	AF132033
DKFZP586G011 protein	BE090926
solute carrier family 4, anion exchanger	NM_000342
CCAAT-box-binding transcription factor	NM_005760
ESTs	AI937119
programmed cell death 1	NM_005018
mago-nashi (Drosophila) homolog, prolife	AW955286
DKFZP586P0123 protein	AI633987
hypothetical protein FLJ13164	AW418525
Junk, low PCR	EMPTY
cyclin K	AI553937
CASP2 and RIPK1 domain containing adapto	AI219605
KIAA0484 protein	AB007953
interleukin enhancer binding factor 2, 4	AU135389
eukaryotic translation initiation factor	BE255189
UNC13 (C. elegans)-like	XM_005627
Human eIF-2-associated p67 homolog mRNA,	EMPTY
galactosidase, alpha	AL577581
hemoglobin, beta	BG529867
Homo sapiens clone 25056 mRNA sequence	BF591424
Human cytochrome P450-IIB (hIIB3) mRNA,	M29873

matrix metalloproteinase 11 (stromelysin	NM_005940
ESTs	AA846909
patched (Drosophila) homolog	U43148
ORF	BC001372
hypothetical protein FLJ20296	AI435207
Homo sapiens, Similar to RIKEN cDNA 1600	AL575864
ESTs, Weakly similar to unnamed protein	BE855471
hypothetical protein FLJ21031	AA772319
hypothetical protein	AL136892
dihydroorotate dehydrogenase	M94065
No ID Incyte EST	EMPTY
Homo sapiens clone 24461 mRNA sequence	AF070577
flap structure-specific endonuclease 1	AU142907
Human hydroxymethylglutaryl-CoA lyase mR	EMPTY
Junk, low PCR	EMPTY
ferritin, light polypeptide	AA554735
kinesin family member 4A	AI598036
colony stimulating factor 1 (macrophage)	BE005887
kallikrein 1, renal/pancreas/salivary	M12706
ESTs	AI221188
Junk, low PCR	EMPTY
cyclin T2	XM_002412
protein disulfide isomerase related prot	AA911269
Junk, low PCR	EMPTY
ESTs, Highly similar to C10_HUMAN PUTATI	AW024816
ESTs	AA142842
dynein, axonemal, light polypeptide 4	AA278275
phosphatidylinositol-4-phosphate 5-kinas	NM_005028
guanine nucleotide binding protein (G pr	AI735771
DEAD/H (Asp-Glu-Ala-Asp/His) box polypep	U75968
CD3Z antigen, zeta polypeptide (TiT3 com	AL557555
Human aminoacylase-1 (ACY1) mRNA, comple	EMPTY
KIAA0668 protein	AL021707
chondroitin sulfate proteoglycan 3 (neur	NM_004386
Homo sapiens Chromosome 16 BAC clone CIT	AI310515
2'-5'oligoadenylate synthetase 2	XM_007001
ATP-binding cassette, sub-family C (CFTR	NM_000352
ESTs	AW079507
uridine monophosphate synthetase (orotat	AU133065
exostoses (multiple) 1	BF057267
Homo sapiens, Similar to hypothetical pr	AW072332
Homo sapiens cDNA FLJ11003 fis, clone PL	AL536250
ESTs	AA682865
ESTs	AA740289
enoyl-Coenzyme A, hydratase/3-hydroxyacy	NM_001966
gamma-tubulin complex protein 2	W25414
proteasome (prosome, macropain) subunit,	AU139712
RAB28, member RAS oncogene family	NM_004249
paired-like homeodomain transcription fa	AF048721
Unknown - Human Control Plate Well H6	EMPTY
eukaryotic translation initiation factor	BE892954

zinc finger protein 267	AU128446
ESTs	AI824320
leukocyte immunoglobulin-like receptor,	NM_006840
Junk, low PCR	EMPTY
hypothetical protein FLJ11088	AI371837
ribosomal protein S6 kinase, 90kD, polyp	NM_004586
zinc finger protein 22 (KOX 15)	AI128612
Junk, low PCR	EMPTY
poly(A)-binding protein, cytoplasmic 1-l	AA732070
ESTs	AA418054
C2H2 (Kruppel-type) zinc finger protein	AK023456
hypothetical protein MGC2722	AI096559
discoidin domain receptor family, member	AI520839
peroxisome proliferative activated recep	AI200451
immunoglobulin (CD79A) binding protein 1	NM_001551
short-chain alcohol dehydrogenase family	NM_005794
Unknown - Human Control Plate Well H10	EMPTY
heat shock 70kD protein 4	BE742483
Homo sapiens, clone MGC:12980, mRNA, com	BC005068
microfibrillar-associated protein 3	BF971389
microtubule-associated protein 4	AL580657
v-raf murine sarcoma viral oncogene homo	NM_004333
putative helicase RUVBL	AA481600
Junk, low PCR	EMPTY
prostaglandin-endoperoxide synthase 2 (p	AL033533
melanoma differentiation associated prot	AA099689
hypothetical protein FLJ23476	N47573
Homo sapiens cDNA: FLJ23546 fis, clone L	AK027199
ESTs, Weakly similar to I38588 reverse t	AW207770
AE-binding protein 1	NM_001129
ESTs	AL138326
baculoviral IAP repeat-containing 5 (sur	AF077350
cell division cycle 34	AI476817
KIAA0419 gene product	AF074665
Empty	EMPTY
nuclear cap binding protein subunit 1, 8	NM_002486
TIA1 cytotoxic granule-associated RNA-bi	M96954
ESTs	BF478055
aspartoacylase (aminoacylase 2, Canavan	XM_008504
dystrophin (muscular dystrophy, Duchenne	AA461118
KIAA0633 protein	AA476314
ESTs, Moderately similar to ALU5_HUMAN A	BG030868
solute carrier family 29 (nucleoside tra	BG335558
luteinizing hormone beta polypeptide	AV751658
Fc fragment of IgG, low affinity IIIa, r	NM_000569
KIAA0401 protein	AB007861
hypothetical protein	BG328180
trinucleotide repeat containing 9	AK025084
translocase of inner mitochondrial membr	AL522889
No ID Incyte EST	EMPTY
hepatic leukemia factor	M95585

branched chain keto acid dehydrogenase E	AV722161
Empty	EMPTY
thyroid hormone receptor interactor 6	AF025437
hypothetical protein FLJ10201	AU148893
nuclear receptor coactivator 1	AA495962
transcriptional activator of the c-fos p	BF339435
solute carrier family 25 (mitochondrial	J04982
Junk, low PCR	EMPTY
surfactant, pulmonary-associated protein	BF224345
mitochondrial ribosomal protein L19	BE886912
potassium voltage-gated channel, delayed	XM_009523
eukaryotic translation initiation factor	BE003309
Human glucocorticoid receptor alpha mRNA	U25029
achaete-scute complex (Drosophila) homol	AI348578
Homo sapiens clone 23821 mRNA sequence	AF038194
contactin 2 (axonal)	AI366526
Junk, low PCR	EMPTY
phenylalanine-tRNA synthetase-like	NM_004461
oxidase (cytochrome c) assembly 1-like	BE785682
Empty	EMPTY
CD58 antigen, (lymphocyte function-assoc	NM_001779
phosphoserine aminotransferase	BG400371
ESTs	F08958
progastricsin (pepsinogen C)	NM_002630
Junk, low PCR	EMPTY
pre-mRNA splicing factor 17	AA527214
histone deacetylase 3	N57752
Junk, low PCR	EMPTY
amyloid beta (A4) precursor protein (pro	X06989
iron-responsive element binding protein	AW150637
deleted in liver cancer 1	NM_006094
ESTs, Moderately similar to ALU1_HUMAN A	AI425049
KIAA0638 protein	BG329723
potassium voltage-gated channel, Shaw-re	XM_006393
EST, Highly similar to 1611455A ros1 gen	AI935615
solute carrier family 25 (carnitine/acyl	AL576290
KIAA0307 gene product	NM_014862
Empty	EMPTY
macrophage stimulating 1 receptor (c-met	NM_002447
glycine C-acetyltransferase (2-amino-3-k	AI638764
ESTs, Weakly similar to IEFS_HUMAN TRANS	AA838362
protein phosphatase 2 (formerly 2A), reg	XM_012105
CD1C antigen, c polypeptide	NM_001765
EST	R58925
ESTs	W35287
Junk, low PCR	EMPTY
exostoses (multiple)-like 3	AF083551
Kreisler (mouse) maf-related leucine zip	AA583143
Human clone 23948 mRNA sequence	U79293
Junk, low PCR	EMPTY
ESTs, Weakly similar to S55024 nebulin,	BE502910

calcium binding protein P22	AU121489
Junk, low PCR	EMPTY
polymerase (DNA directed), delta 1, cata	BE274988
amphiphysin (Stiff-Mann syndrome with br	NM_001635
Empty	EMPTY
stimulated trans-acting factor (50 kDa)	BG164410
ESTs	AF052496
ESTs	BE018176
protein tyrosine phosphatase, non-recept	NM_007039
proliferation-associated 2G4, 38kD	AU120518
geranylgeranyl diphosphate synthase 1	AV748079
ESTs	AA436249
S100 calcium-binding protein A13	AI056032
Homo sapiens diacylglycerol kinase, delt	XM_002384
ESTs, Weakly similar to T00370 hypotheti	AA203236
Junk, low PCR	EMPTY
solute carrier family 7, (cationic amino	BG291471
uroplakin 1B	AB002155
solute carrier family 7 (cationic amino	X57303
epilepsy, progressive myoclonus type 2,	AL582866
DNA segment on chromosome X and Y (uniqu	N42070
5-aminoimidazole-4-carboxamide ribonucle	BG250822
Empty	EMPTY
gap junction protein, alpha 1, 43kD (con	AL575958
M-phase phosphoprotein 1	AA426191
WAS protein family, member 2	AI632159
KIAA0528 gene product	AB011100
small nuclear ribonucleoprotein polypept	AL514750
ESTs	AA862853
hypothetical protein FLJ20671	AI337864
3-phosphoinositide dependent protein kin	NM_002613
solute carrier family 4, sodium bicarbon	NM_003759
ESTs	BG111216
pyrroline-5-carboxylate reductase 1	AU132733
hypothetical protein FLJ12838	AA250932
KIAA0844 protein	NM_014951
dickkopf (Xenopus laevis) homolog 1	AL552970
DKFZp434P211 protein	NM_014549
cadherin 1, type 1, E-cadherin (epitheli	NM_004360
insulin receptor substrate 1	NM_005544
natural killer cell transcript 4	AI539055
apolipoprotein C-IV	NM_001646
ESTs	AI081832
GATA-binding protein 6	NM_005257
apoptotic protease activating factor	AB007873
carnitine O-octanoyltransferase	AW190832
aldehyde dehydrogenase 4 family, member	NM_003748
annexin A3	AA876532
Junk, low PCR	EMPTY
Homo sapiens cDNA: FLJ21960 fis, clone H	AA465704
adaptor-related protein complex 1, gamma	AI246767

ESTs, Weakly similar to T2D4_HUMAN TRANS	AI092784
heat shock 70kD protein-like 1	AI955159
unr-interacting protein	BG286903
Homo sapiens clone 23798 and 23825 mRNA	AL134611
Snf2-related CBP activator protein	AB002307
hypothetical protein	NM_013298
dolichyl-phosphate mannosyltransferase p	AI423312
3'-phosphoadenosine 5'-phosphosulfate sy	NM_005443
procollagen-proline, 2-oxoglutarate 4-di	BE298899
ESTs	N48809
acyl-Coenzyme A oxidase 1, palmitoyl	AL550517
Junk, low PCR	EMPTY
Homo sapiens cDNA FLJ11381 fis, clone HE	AA928014
KIAA0258 gene product	BC001725
visinin-like 1	H73600
phosphodiesterase 7A	AW369822
ESTs, Highly similar to KIAA1395 protein	BE857715
Homo sapiens cDNA FLJ14246 fis, clone OV	AW675540
ESTs	BE501444
cholinergic receptor, muscarinic 3	AI524284
SPARC-like 1 (mast9, hevin)	NM_004684
tubulin-specific chaperone a	AA593605
ribosomal protein S15a	BG285655
glycine receptor, beta	AF094755
nuclear receptor subfamily 5, group A, m	NM_004959
2,3-bisphosphoglycerate mutase	NM_001724
sorting nexin 17	AU142124
hypothetical protein	AI004747
deoxyribonuclease II, lysosomal	AL526369
Junk, low PCR	EMPTY
hypothetical protein FLJ11790	AI018181
SH2 domain protein 2A	NM_003975
nucleolar autoantigen (55kD) similar to	AI475246
tyrosylprotein sulfotransferase 1	NM_003596
ESTs	AW271620
anterior gradient 2 (Xenepus laevis) hom	AI888294
ESTs	BF434499
Homo sapiens clone 25038 mRNA sequence	AF131824
KIAA0143 protein	D63477
Homo sapiens chromosome 19, cosmid R2689	AA534193
adaptor-related protein complex 4, mu 1	AL554598
cytochrome c oxidase subunit VIa polypep	AI421088
potassium inwardly-rectifying channel,su	AF061118
IK cytokine, down-regulator of HLA II	AL573955
suppression of tumorigenicity 13 (colon	BG036342
integral type I protein	AW192074
Meis (mouse) homolog 3	AI278906
glucokinase (hexokinase 4, maturity onse	NM_000162
UDP-glucose:glycoprotein glucosyltransfe	AW503100
T cell activation, increased late expres	XM_011080
selenoprotein W, 1	BG476052

Junk, low PCR	EMPTY
Homo sapiens cDNA: FLJ21880 fis, clone H	AA020911
ESTs	AW300965
hypothetical protein	AA256521
apoptosis-associated tyrosine kinase	BF940808
proteoglycan 1, secretory granule	AV734015
G protein-coupled receptor	NM_006564
Homo sapiens clone 24627 mRNA sequence	AF070618
serine/threonine-protein kinase PRP4 hom	NM_003913
GDP dissociation inhibitor 1	BG395127
GT198, complete ORF	BF056976
thyroid hormone receptor interactor 11	NM_004239
cyclin E2	NM_004702
eukaryotic translation initiation factor	AA595774
DKFZP586G0522 protein	AF272151
Homo sapiens cDNA: FLJ22563 fis, clone H	AI393566
dystroglycan 1 (dystrophin-associated gl	NM_004393
toll-like receptor 5	AF051151
wingless-type MMTV integration site fami	XM_003085
CGI-94 protein	AA860466
hypothetical protein FLJ10726	AL536999
ESTs	BE673908
KIAA0748 gene product	NM_014796
v-abl Abelson murine leukemia viral onco	BG424635
ADP-ribosylation factor-like 7	BE311543
actin related protein 2/3 complex, subun	BG036956
mutL (E. coli) homolog 3	XM_007426
DC12 protein	AF035296
hypothetical protein MGC5149	U79260
antizyme inhibitor	NM_015878
ESTs	AI168025
ectonucleoside triphosphate diphosphohyd	NM_001776
protein tyrosine phosphatase, receptor t	BE895330
Homo sapiens cDNA: FLJ23004 fis, clone L	AI205555
Junk, low PCR	EMPTY
acidic protein rich in leucines	AA987219
sodium channel, voltage-gated, type IX,	NM_002977
Homo sapiens cDNA: FLJ22050 fis, clone H	AA044755
similar to APOBEC1	AA853167
ESTs	BF221704
Homo sapiens mRNA; cDNA DKFZp761I2123 (f	AL136572
carbamoyl-phosphate synthetase 2, aspart	BG393415
Human clone 23839 mRNA sequence	U79249
lipase, hormone-sensitive	NM_005357
excision repair cross-complementing rode	NM_000123
latent transforming growth factor beta b	AF051344
Human mRNA for mitochondrial short-chain	EMPTY
fatty acid amide hydrolase	NM_001441
KIAA0442 protein	AK025298
protein kinase, cAMP-dependent, regulato	NM_004157
methylmalonyl Coenzyme A mutase	NM_000255

hypothetical protein FLJ12666	AW952494
zinc finger protein 33a (KOX 31)	AL161931
granzyme A (granzyme 1, cytotoxic T-lymp	AA283172
ESTs	AA994709
hypothetical protein FLJ11200	AA476815
hypothetical protein	AI608624
hypothetical protein FLJ14033 similar to	AW270837
ESTs	AA653638
protein-L-isoaspartate (D-aspartate) O-m	T19312
v-src avian sarcoma (Schmidt-Ruppin A-2)	XM_009641
regulatory solute carrier protein, famil	AI268381
KIAA1043 protein	AI271769
high density lipoprotein binding protein	NM_005336
Human cytoplasmic beta-actin gene, compl	EMPTY
transcription factor EC	NM_012252
KIAA0263 gene product	D87452
immature colon carcinoma transcript 1	XM_008185
regulator of G-protein signalling 2, 24k	AW302145
Sam68-like phosphotyrosine protein, T-ST	AA112001
kallikrein 2, prostatic	AF188747
ESTs, Highly similar to IFT2_HUMAN INTER	AI609624
Homo sapiens cDNA: FLJ21897 fis, clone H	BE259271
G-protein gamma-12 subunit	AI138429
KIAA1572 protein	BF435359
Homo sapiens cDNA: FLJ22046 fis, clone H	AI167988
Junk, low PCR	EMPTY
calcium channel, voltage-dependent, beta	XM_006783
f-box and leucine-rich repeat protein 4	XM_004359
guanylate kinase 1	T36282
Protein inhibitor of activated STAT X	N25685
general transcription factor IIA, 2 (12k	BG431140
H.sapiens mRNA for elongations factor Tu	EMPTY
numb (Drosophila) homolog	AL541223
B-cell CLL/lymphoma 7B	BF341713
Junk, low PCR	EMPTY
nucleobindin 2	NM_005013
WD repeat domain 3	AL121993
Junk, low PCR	EMPTY
Junk, low PCR	EMPTY
ESTs	AA700126
Homo sapiens cDNA FLJ13596 fis, clone PL	AA758701
tetraspan NET-6 protein	BG547021
hypothetical protein	NM_019042
K562 cell-derived leucine-zipper-like pr	AI298083
quinoid dihydropteridine reductase	AA159812
antigenic determinant of recA protein (m	AW001823
fucosyltransferase 7 (alpha (1,3) fucosy	NM_004479
cytokine-inducible kinase	NM_004073
voltage-dependent anion channel 3	AW337218
Human chaperonin protein (Tcp20) gene co	EMPTY
selenium binding protein 1	AL531951

glutamate receptor, metabotropic 3	NM_000840
glycogen synthase kinase 3 alpha	AL536089
TATA box binding protein	AL563148
ESTs	AV758288
microsomal glutathione S-transferase 2	W73858
protease, serine, 1 (trypsin 1)	NM_002769
Homo sapiens mRNA for FLJ00024 protein,	AI673501
ESTs	AL037069
delta-like 4 homolog (Drosophila)	AB036931
Junk, low PCR	EMPTY
ESTs	AI809797
topoisomerase (DNA) II alpha (170kD)	BF795918
coronin, actin-binding protein, 2B	AL533625
Snf2-related CBP activator protein	BE266406
No ID Incyte EST	EMPTY
RAB3A, member RAS oncogene family	NM_002866
Human ADP-ribosylation factor 1 (ARF1) m	EMPTY
guanine nucleotide binding protein (G pr	BG031959
nuclear factor of kappa light polypeptid	AL121928
KIAA0376 protein	AB002374
Junk, low PCR	EMPTY
ESTs, Weakly similar to JC5270 neuron-sp	AW410140
transglutaminase 4 (prostate)	NM_003241
ribosomal protein L29	BG108245
ESTs	AA830050
ESTs	AA417966
hypothetical protein FLJ23414	AA232651
ESTs	AI920902
Homo sapiens cDNA FLJ11415 fis, clone HE	AA663075
Junk, low PCR	EMPTY
peroxisome biogenesis factor 10	BE326641
solute carrier family 22 (organic cation	AL353625
KIAA0782 protein	AI702299
Kruppel-like factor 5 (intestinal)	XM_007199
H.sapiens PMI1 mRNA for phosphomannose i	EMPTY
chemokine (C-C motif) receptor 2	XM_002923
tumor necrosis factor (ligand) superfami	NM_000639
deoxyhypusine synthase	AL520040
dystrobrevin, beta	NM_021907
ESTs	AA040945
A kinase (PRKA) anchor protein 1	NM_003488
Junk, low PCR	EMPTY
DKFZP586F1018 protein	BF478223
ESTs	AA496222
KIAA1102 protein	AI027810
SBBI26 protein	NM_018846
ESTs	AI373658
RAE1 (RNA export 1, S.pombe) homolog	AW590594
neuronal pentraxin II	U26662
KIAA0906 protein	AB020713
cholinergic receptor, nicotinic, alpha p	U62434

prominin (mouse)-like 1	AU124416
Empty	EMPTY
transcription factor 2, hepatic; LF-B3;	BF109358
Junk, low PCR	EMPTY
ESTs, Moderately similar to ALU7_HUMAN A	AI241633
KIAA0282 protein	AF220036
phosphate cytidylyltransferase 1, cholin	AL521633
calcium modulating ligand	AW474551
KIAA0077 protein	BE784260
ESTs, Weakly similar to OGT1_HUMAN UDP-N	BE218499
makorin, ring finger protein, 3	NM_005664
slit (Drosophila) homolog 3	BE378989
adenylyl cyclase-associated protein 2	AW779995
hypothetical protein FLJ10815	AL133676
KIAA0877 protein	AI399686
No ID Incyte EST	EMPTY
tachykinin receptor 2	AI971217
RNA polymerase II transcriptional regula	AU145545
CDC45 (cell division cycle 45, S.cerevis	NM_003504
Empty	EMPTY
SKIP for skeletal muscle and kidney enri	AL530949
Junk, low PCR	EMPTY
zinc finger protein 106	F25339
tumor necrosis factor receptor superfami	BG436824
zinc finger protein 174	NM_003450
surfactant, pulmonary-associated protein	NM_006926
interferon-induced protein with tetratri	NM_001548
hypothetical protein hCLA-iso	AI339374
retinoic acid responsive	AL523854
hypothetical protein AF301222	AA897593
ESTs, Weakly similar to A48042 lysosomal	N24741
calpastatin	BG168693
Homo sapiens mRNA; cDNA DKFZp434A1010 (f	AL137579
branched chain aminotransferase 1, cytos	AI970531
No ID Incyte EST	EMPTY
Homo sapiens clone 24889 mRNA sequence	AW576274
lumican	AU137979
Empty	EMPTY
clones 23667 and 23775 zinc finger prote	BG288421
myogenic factor 6 (herculin)	NM_002469
Junk, low PCR	EMPTY
splicing factor, arginine/serine-rich 9	AL525031
6-phosphofructo-2-kinase/fructose-2,6-bi	XM_011863
Junk, low PCR	EMPTY
histidine decarboxylase	D16583
adaptor-related protein complex 3, mu 2	NM_006803
folate receptor 1 (adult)	AL555980
hypothetical protein FLJ12085	AA608723
cyclin-dependent kinase 9 (CDC2-related	AI076591
Homo sapiens mRNA for KIAA1413 protein,	AB037834
tryptase gamma 1	AF175759

potassium inwardly-rectifying channel, s	NM_004983
ESTs	BE250134
ESTs	AA934119
NADH dehydrogenase (ubiquinone) 1 alpha	AA782463
Empty	EMPTY
annexin A4	AA773563
heat shock 105kD	AA232636
protocadherin 11	AA865592
general transcription factor IIE, polype	NM_002095
phosphodiesterase 4A, cAMP-specific (dun	U97584
Junk, low PCR	EMPTY
suc1-associated neurotrophic factor targ	NM_006653
Junk, low PCR	EMPTY
frizzled (Drosophila) homolog 5	XM_010838
ESTs	BF508148
ESTs, Weakly similar to dJ963K23.2 [H.sa	AI282666
vacuolar protein sorting 45B (yeast homo	AU160506
LIM domain binding 2	AF064493
solute carrier family 22 (organic cation	AL570413
Junk, low PCR	EMPTY
carbohydrate (chondroitin 6/keratan) sul	AI041015
amyloid beta precursor protein (cytoplas	D86981
Empty	EMPTY
malate dehydrogenase 1, NAD (soluble)	BF105286
endothelin converting enzyme 1	NM_001397
ESTs, Weakly similar to KIAA1435 protein	AI907874
TATA box binding protein (TBP)-associate	BE394272
Junk, low PCR	EMPTY
keratin 5 (epidermolysis bullosa simplex	M19723
glutamate-cysteine ligase, catalytic sub	NM_001498
PDGFA associated protein 1	BE746861
GATA-binding protein 4	XM_005135
ESTs	BF939733
Homo sapiens chromosome 19, cosmid R2837	AA219003
snail 1 (drosophila homolog), zinc finge	AW268385
Junk, low PCR	EMPTY
killer cell lectin-like receptor subfami	AF027164
ESTs	T89501
arginine-glutamic acid dipeptide (RE) re	AW665548
KIAA0117 protein	D38491
Empty	EMPTY
ribophorin I	BE780549
melanoma antigen, family A, 6	BF792356
ESTs	AA453902
KIAA0319 gene product	NM_014809
pumilio (Drosophila) homolog 1	NM_014676
clusterin-like 1 (retinal)	NM_014410
parvalbumin	AV723498
Junk, low PCR	EMPTY
killer cell immunoglobulin-like receptor	AF285436
hypothetical protein FLJ14225	AA148817

Junk, low PCR	EMPTY
Junk, low PCR	EMPTY
metalloprotease 1 (pitrilysin family)	AK023476
XIAP associated factor-1	BG541762
KIAA0489 protein	AB007958
ephrin-A1	BG256240
unc119 (C.elegans) homolog	AL583682
leukocyte tyrosine kinase	AW451303
syntaxin binding protein 2	XM_008937
ESTs	AA831572
ESTs, Weakly similar to ALU2_HUMAN ALU S	AA447777
KIAA0335 gene product	NM_014803
Junk, low PCR	EMPTY
glypican 4	AA887423
dihydrolipoamide S-succinyltransferase (	BC000302
actin related protein 2/3 complex, subun	BE545861
mitochondrial ribosome recycling factor	AL577765
CS box-containing WD protein	BF110766
Homo sapiens mRNA for WDC146, complete c	AA927896
mannosidase, alpha, class 1A, member 1	NM_005907
Homo sapiens clone 24538 mRNA sequence	AW338844
Homo sapiens clone 23798 and 23825 mRNA	AF035308
topoisomerase (DNA) II binding protein	AW027550
ectonucleotide pyrophosphatase/phosphodi	BE700679
chymotrypsinogen B1	AW951193
v-Ki-ras2 Kirsten rat sarcoma 2 viral on	AI740449
KIAA0123 protein	BG327417
ESTs	AI061381
ESTs	AA233152
glypican 1	XM_002321
nuclear receptor subfamily 1, group D, m	NM_021724
ESTs	AI097383
KIAA0551 protein	AB011123
transcription elongation factor B (SIII)	AA521128
Breakpoint cluster region protein, uteri	W02877
tight junction protein 1 (zona occludens	NM_003257
ESTs, Weakly similar to ubiquitous TPR m	AA973353
DNA replication factor	BE264419
betaine-homocysteine methyltransferase	AL531105
suppressor of Ty (S.cerevisiae) 3 homolo	AF064804
tumor suppressing subtransferable candid	BE746125
plasminogen activator, urokinase	NM_002658
anchor attachment protein 1 (Gaa1p, yeas	BE271496
eukaryotic translation initiation factor	AL117412
KIAA0368 protein	BG110603
SH3-domain binding protein 1	BE552177
ESTs	AW172431
protein phosphatase 1, regulatory subuni	XM_004553
feline sarcoma (Snyder-Theilen) viral (v	NM_002005
hypothetical protein FLJ10486	AA884787
UDP-N-acetyl-alpha-D-galactosamine:(N-ac	NM_001478

general transcription factor IIH, polype	BF059061
hypothetical protein FLJ12691	BE786271
Junk, low PCR	EMPTY
ESTs	AI056392
killer cell lectin-like receptor subfami	AF023840
histamine receptor H1	NM_000861
KIAA0857 protein	AF153085
voltage-dependent anion channel 1	AV717783
aminopeptidase puromycin sensitive	AU131519
bone morphogenetic protein receptor, typ	NM_001204
galactokinase 1	NM_000154
xeroderma pigmentosum, complementation g	NM_004628
hypothetical protein FLJ12389 similar to	AI697801
Gem-interacting protein	AA055973
neuropeptide Y receptor Y1	NM_000909
KIAA0763 gene product	AU143342
ESTs	AA286901
Junk, low PCR	EMPTY
anti-Mullerian hormone receptor, type II	AF172932
KIAA0187 gene product	AW205569
Homo sapiens cDNA FLJ13408 fis, clone PL	AI436552
ESTs	AI937789
step II splicing factor SLU7	BG539628
solute carrier family 16 (monocarboxylic	NM_003051
amyloid beta (A4) precursor protein-bind	U62325
Junk, low PCR	EMPTY
DnaJ (Hsp40) homolog, subfamily A, membe	NM_001539
insulin	AW582955
Cas-Br-M (murine) ectropic retroviral tr	BE043961
IQ motif containing GTPase activating pr	NM_006633
A kinase (PRKA) anchor protein (yotiao)	BG482762
ESTs	AA046941
spinocerebellar ataxia 2 (olivopontocere	NM_002973
pim-2 oncogene	AU149260
DKFZP547E1010 protein	AW970696
Junk, low PCR	EMPTY
Junk, low PCR	EMPTY
epithelial protein lost in neoplasm beta	AK023649
Homo sapiens partial TCF-4 gene for T-ce	AJ270770
dimethylarginine dimethylaminohydrolase	BF515233
golgi autoantigen, golgin subfamily a, 4	BE901929
pericentriolar material 1	H00568
mesothelin	AI813749
Homo sapiens clone 24462 mRNA sequence	AF070584
polymerase (DNA directed), delta 2, regu	BE563333
vaccinia related kinase 1	AA312869
ubiquitin carrier protein	AI571293
mastermind (Drosophila), homolog of	AF221759
hypothetical protein FLJ21127	AA536020
hypothetical protein	AI924350
eukaryotic translation initiation factor	BE514642

proteasome (prosome, macropain) subunit,	AI923541
A kinase (PRKA) anchor protein 8	BG256627
Sjogren syndrome antigen A2 (60kD, ribon	AW162240
homeo box A5	M26679
Homo sapiens cDNA: FLJ20925 fis, clone A	AW519080
alcohol dehydrogenase 3 (class I), gamma	NM_000669
Junk, low PCR	EMPTY
NBR2	NM_005821
STAT induced STAT inhibitor 3	AB006967
homeo box B2	NM_002145
MAP/microtubule affinity-regulating kina	BF448156
sema domain, immunoglobulin domain (Ig),	NM_006378
plakophilin 1 (ectodermal dysplasia/skin	NM_000299
Human coatomer protein (HEPCOP) mRNA, co	EMPTY
protein phosphatase 1, catalytic subunit	AA520993
KIAA0332 protein	AA994431
ESTs	AI004957
phosphoinositide-3-kinase, regulatory su	XM_010581
neurotrophic tyrosine kinase, receptor,	S76476
hypothetical protein MGC5487	AA905113
Novel human gene mapping to chomosome 13	BF439351
bromodomain-containing 2	AI423143
Homo sapiens cDNA FLJ10158 fis, clone HE	AI383770
ESTs	AV682305
ESTs	BE465414
hypothetical protein FLJ22625	BF966625
lymphotoxin beta (TNF superfamily, membe	AW978651
zinc finger protein 74 (Cos52)	XM_009848
general transcription factor IIH, polype	AW401633
STIP1 homology and U-Box containing prot	AL560352
NCK-associated protein 1	XM_010767
Human alpha-N-acetylgalactosaminidase mR	EMPTY
transducin-like enhancer of split 1, hom	AA223480
rearranged L-myc fusion sequence	NM_012421
zinc finger protein 313	AF131742
ARS component B	X99977
granzyme M (lymphocyte met-ase 1)	NM_005317
ESTs, Highly similar to JC7326 bood POZ	AW005824
FKBP-associated protein	BC001257
keratin 18	BG387311
Homo sapiens glucocorticoid receptor AF-	AI333482
Homo sapiens cDNA: FLJ22785 fis, clone K	AI890347
ESTs	AW470039
hypothetical protein FLJ22638	BE559877
endothelial differentiation, lysophospha	AV705262
solute carrier family 10 (sodium/bile ac	NM_000452
neuropeptide FF-amide peptide precursor	AI417333
protein expressed in thyroid	NM_014297
serine (or cysteine) proteinase inhibito	AL531502
Human acidic ribosomal phosphoprotein P0	EMPTY
hypothetical protein FLJ11193	AF038176

solute carrier family 6 (neurotransmitte	NM_014228
ESTs, Weakly similar to unnamed protein	AV720103
hexokinase 3 (white cell)	NM_002115
nuclear receptor subfamily 1, group H, m	XM_006165
Arg/Abl-interacting protein ArgBP2	AA939266
eukaryotic translation elongation factor	BE615446
toll-like receptor 3	AL570789
Homo sapiens mRNA; cDNA DKFZp564M0264 (f	AI377950
Homo sapiens colon cancer-associated ant	AL519958
DKFZP434H132 protein	AW468805
GTP-binding protein ragB	AV704109
Homo sapiens immunoglobulin mu chain ant	AA490743
cyclin-dependent kinase 6	H70635
serine/threonine kinase 17a (apoptosis-i	NM_004760
arrestin, beta 1	BC003636
actin, gamma 1	BG422944
Human topoisomerase I mRNA, complete cds	EMPTY
H326	AA242795
Human pre TCR alpha mRNA, partial cds	AL035587
DKFZP727G051 protein	AA194312
chemokine (C-C motif) receptor 5	AF009962
tryptase, alpha	NM_003293
Homo sapiens cDNA FLJ11616 fis, clone HE	AA825430
Ral guanine nucleotide exchange factor R	NM_014636
Junk, low PCR	EMPTY
KIAA0758 protein	AI093508
ESTs	BE875636
ESTs, Weakly similar to 1313184B alpha1	AA458648
ESTs	AI792251
neuronal pentraxin II	U26662
non-metastatic cells 4, protein expresse	AV704094
tissue factor pathway inhibitor 2	AL550357
Melanoma associated gene	AF200348
LIM protein (similar to rat protein kina	BG054550
Lactate Dehydrogenase A	EMPTY
Human insulin-like growth factor binding	AU132011
Ras-GTPase-activating protein SH3-domain	BG252290
hypothetical protein FLJ20186	BF688383
KIAA0524 protein	AJ290445
carboxypeptidase A1 (pancreatic)	NM_001868
Homo sapiens cDNA: FLJ23507 fis, clone L	AA682502
Homo sapiens immunoglobulin mu chain ant	BF975992
secretory carrier membrane protein 1	NM_004866
ESTs, Weakly similar to TESK_HUMAN TESTI	AI539296
KIAA0911 protein	AI334166
cathepsin F	BE502253
KIAA1171 protein	BE044292
purinergic receptor (family A group 5)	AI823889
MAX binding protein	BE311558
Junk, low PCR	EMPTY
Junk, low PCR, low PCR	EMPTY

galactose-1-phosphate uridylyltransferas	BF347284
Human alpha-2-macroglobulin mRNA, comple	EMPTY
Junk, low PCR	EMPTY
Junk, low PCR	EMPTY
Homo sapiens cDNA FLJ14206 fis, clone NT	AA494119
Junk, low PCR	EMPTY
PRO1748 protein	BE739599
Homo sapiens cDNA: FLJ22841 fis, clone K	AA451700
cytochrome P450, subfamily IIC (mephenyt	M61853
solute carrier family 6 (neurotransmitte	XM_003160
Homo sapiens cDNA: FLJ21693 fis, clone C	AA455058
brefeldin A-inhibited guanine nucleotide	BE301810
ESTs	R13843
Junk, low PCR	EMPTY
chromosome 6 open reading frame 9	AW955299
No ID Incyte EST	EMPTY
cell division cycle 2-like 1 (PITSLRE pr	BE268611
sorbitol dehydrogenase	BE872940
KIAA0063 gene product	NM_014876
Arabadopsis7-125	EMPTY
Human SH3 domain-containing protein SH3P	U61167
acetyl-Coenzyme A acetyltransferase 1 (a	AI683154
ESTs	AW183185
proline-rich protein BstNI subfamily 3	BF088455
KIAA0301 protein	N67901
endosulfine alpha	AW074504
Homo sapiens mRNA; cDNA DKFZp434O1230 (f	AA399341
hypoxia-inducible factor 1, alpha subuni	AU134078
N-acetyltransferase 2 (arylamine N-acety	AV658656
Tax1 (human T-cell leukemia virus type I	AA565928
Junk, low PCR	EMPTY
Homo sapiens cDNA FLJ11477 fis, clone HE	AA858162
cancer/testis antigen 2	AX024689
Junk, low PCR	EMPTY
sema domain, immunoglobulin domain (Ig),	AF030698
nuclear receptor subfamily 2, group F, m	BC002669
C-type (calcium dependent, carbohydrate-	XM_006626
Arabadopsis9-125	EMPTY
Junk, low PCR	EMPTY
prostaglandin E receptor 4 (subtype EP4)	NM_000958
RNA helicase family	AA632279
Junk, low PCR	EMPTY
epidermal growth factor receptor pathway	NM_004447
ESTs, Weakly similar to KIAA1196 protein	R59703
Junk, low PCR	EMPTY
bone morphogenetic protein 1	NM_006129
hydroxy-delta-5-steroid dehydrogenase, 3	AL541089
hypothetical protein FLJ20727	AA534418
rat regenerating islet-derived-like, hum	NM_006508
host cell factor 2	AW628925
No ID Incyte EST	EMPTY

HS1 binding protein	BE565456
membrane-bound transcription factor prot	AU099021
KIAA0317 gene product	NM_014821
isocitrate dehydrogenase 3 (NAD+) alpha	BG429703
Empty	EMPTY
myosin phosphatase, target subunit 1	AW847768
KIAA0220 protein	AC003007
TATA-binding protein-binding protein	AA894779
calreticulin	AU117505
BCS1 (yeast homolog)-like	NM_004328
ESTs	BF059298
solute carrier family 25 (carnitine/acyl	AA779691
trefoil factor 3 (intestinal)	AA633399
MAD (mothers against decapentaplegic, Dr	AF009678
paired immunoglobulin-like receptor alph	BE672423
trehalase (brush-border membrane glycopr	AI793190
actin, alpha 1, skeletal muscle	AW131988
Junk, low PCR	EMPTY
KIAA0773 gene product	AL533847
phosphotriesterase related	BG170397
unc-51 (C. elegans)-like kinase 1	NM_003565
vasoactive intestinal peptide receptor 1	NM_004624
Empty	EMPTY
farnesyl diphosphate synthase (farnesyl	BF568094
hypothetical protein DKFZp547M136 simila	AW241407
Junk, low PCR	EMPTY
integrin, alpha 4 (antigen CD49D, alpha	NM_000885
G protein pathway suppressor 2	AA167701
Homo sapiens, clone IMAGE:3632533, mRNA,	BE673413
KIAA0863 protein	AA629323
KIAA1051 protein	AB028974
guanylate cyclase 1, soluble, alpha 2	NM_000855
membrane-spanning 4-domains, subfamily A	AV758743
solute carrier family 25 (mitochondrial	AL574883
Junk, low PCR	EMPTY
phosphofructokinase, liver	AL041002
chaperonin containing TCP1, subunit 7 (e	AA075945
KIAA0853 protein	AL136745
intercellular adhesion molecule 3	AU122157
succinate dehydrogenase complex, subunit	NM_003002
Empty	EMPTY
spinocerebellar ataxia 1 (olivopontocere	XM_004164
ESTs	AW974802
Homo sapiens mRNA; cDNA DKFZp761J191 (fr	BE855498
ubiquitin-conjugating enzyme E2D 2 (homo	AI553806
ribosomal protein L38	NM_000999
eukaryotic translation initiation factor	AW247934
DKFZP434N093 protein	AW627377
aldolase A, fructose-bisphosphate	BG481883
proteasome (prosome, macropain) 26S subu	BG398881
ESTs, Moderately similar to ALU1_HUMAN A	AA253017

ribose 5-phosphate isomerase A (ribose 5	BF969830
ESTs, Weakly similar to ORF2: function u	BE222281
interleukin 12A (natural killer cell sti	M65291
hypothetical protein	AI420520
Homo sapiens clone 24758 mRNA sequence	AF070568
U2(RNU2) small nuclear RNA auxillary fac	BE792382
structure specific recognition protein 1	AI635077
Empty	EMPTY
bromodomain and PHD finger containing, 1	NM_004634
Junk, low PCR	EMPTY
No ID Incyte EST	EMPTY
Junk, low PCR	EMPTY
Homo sapiens cDNA: FLJ21927 fis, clone H	AI671747
hypothetical protein FLJ12827	BF061011
ESTs	AW957220
5T4 oncofetal trophoblast glycoprotein	XM_004148
paired box gene 2	NM_003988
Human DNA sequence from clone RP1-310O13	BE466024
cofactor required for Sp1 transcriptiona	AV702955
ESTs	AI302836
polymerase (RNA) II (DNA directed) polyp	AA598900
hydroxysteroid (17-beta) dehydrogenase 1	AU139139
angiopoietin-like factor	AL049653
hypothetical protein PRO2507	AF119883
fibrillin 2 (congenital contractural ara	X62009
lectin, galactoside-binding, soluble, 9	AB005894
ribosomal protein L10	BG260750
ESTs	AI089136
transmembrane 4 superfamily member 5	XM_008517
ciliary neurotrophic factor	NM_000614
G protein-coupled receptor 65	BE326632
KIAA0281 gene product	AL539552
nuclease sensitive element binding prote	AL044249
matrilin 1, cartilage matrix protein	AL576793
SMC2 (structural maintenance of chromoso	AI911543
ESTs	AA521497
Homo sapiens cDNA FLJ14302 fis, clone PL	AA490069
Homo sapiens clone 24670 mRNA sequence	AF055019
splicing factor 3b, subunit 1, 155kD	AL547280
ligase IV, DNA, ATP-dependent	NM_002312
Musashi (Drosophila) homolog 1	AK023262
adrenergic, beta, receptor kinase 1	BG397697
neuroepithelial cell transforming gene 1	BF055177
zinc finger protein homologous to Zfp103	AL157608
HMT1 (hnRNP methyltransferase, S. cerevi	BE793479
Junk, low PCR	EMPTY
UDP-Gal:betaGlcNAc beta 1,4- galactosylt	NM_003780
replication protein A3 (14kD)	BF030135
hypothetical protein dJ551D2.5	AA442098
N-acylsphingosine amidohydrolase (acid c	AW089586
KIAA0513 gene product	NM_014732

Junk, low PCR	EMPTY
EST	N62143
chromosome 16 open reading frame 5	BE677392
ESTs	AI685514
Kallmann syndrome 1 sequence	AA419498
KIAA0164 gene product	AI636054
Ca2+-dependent activator protein for sec	AK000873
interferon consensus sequence binding pr	AW964220
toll-like receptor 1	AL050262
HIV TAT specific factor 1	BE891981
heat shock 70kD protein 8	BG504802
ring finger protein 13	NM_007282
ESTs	AA978069
cell membrane glycoprotein, 110000M(r) (	BF033463
protease inhibitor 3, skin-derived (SKAL	L10343
ESTs	AA460697
PDZ domain containing guanine nucleotide	AL041825
cAMP responsive element binding protein-	AU131473
cystic fibrosis transmembrane conductanc	AW377268
ESTs	BF436141
hypothetical protein FLJ10326	AU154605
FLJ00005 protein	AK000005
RAB6A, member RAS oncogene family	AV717629
UDP-N-acetyl-alpha-D-galactosamine:polyp	XM_001319
stearoyl-CoA desaturase (delta-9-desatur	AF097514
ras homolog gene family, member	AI167227
novel RGD-containing protein	AL545403
G protein-coupled receptor 3	AL096774
KIAA0594 protein	AB011166
KIAA0610 protein	AL139377
ESTs	AI097570
ADP-ribosylation factor 3	BE005888
deoxynucleotidyltransferase, terminal	NM_004088
KIAA0643 protein	AI571428
adenine phosphoribosyltransferase	BG478064
Cockayne syndrome 1 (classical)	NM_000082
dual specificity phosphatase 5	U16996
phospholipase C, delta 4	AW207249
Junk, low PCR	EMPTY
ESTs	BG230619
synaptosomal-associated protein, 29kD	XM_009851
ATPase, H+ transporting, lysosomal (vacu	AI096638
kallikrein 8 (neuropsin/ovasin)	AF243527
folate receptor 3 (gamma)	NM_000804
sepiapterin reductase (7,8-dihydrobiopte	AA864778
nuclear autoantigen	NM_014574
guanine nucleotide binding protein 11	BF115554
sphingomyelin phosphodiesterase 2, neutr	NM_003080
Junk, low PCR	EMPTY
cleavage stimulation factor, 3' pre-RNA,	NM_001324
solute carrier family, member 4	NM_000441

mammalian inositol hexakisphosphate kina	AW771746
immunoglobulin superfamily, member 2	BE746215
Junk, low PCR	EMPTY
tumor protein p53-binding protein, 2	AI123916
ESTs, Weakly similar to A48809 carboxyle	N44535
protein phosphatase 1, regulatory (inhib	AI141349
ESTs, Weakly similar to 1207289A reverse	N51086
thymopoietin	U09088
ATPase, Ca++ transporting, cardiac muscl	AI620652
neurotensin receptor 1 (high affinity)	NM_002531
nicastrin	BE709358
calcium channel, voltage-dependent, L ty	NM_000720
leukocyte immunoglobulin-like receptor,	AF004231
transformer-2 alpha (htra-2 alpha)	BG250879
heat shock 90kD protein 1, beta	BG336532
KIAA1458 protein	N42901
Junk, low PCR	EMPTY
A kinase (PRKA) anchor protein (yotiao)	NM_005751
Homo sapiens mRNA; cDNA DKFZp434E1822 (f	AI090186
SWI/SNF related, matrix associated, acti	NM_003069
cytochrome c oxidase subunit VIIa polype	AI086994
v-ral simian leukemia viral oncogene hom	BE885050
Homo sapiens mRNA; cDNA DKFZp564O1016 (f	AI184424
Junk, low PCR	EMPTY
EST	AA156287
KIAA0295 protein	AI858946
methyl-CpG binding domain protein 2	AI679787
myotubularin related protein 4	BG012919
phosphatidylinositol transfer protein, m	AI561016
S-adenosylhomocysteine hydrolase	BF794088
v-yes-1 Yamaguchi sarcoma viral oncogene	NM_005433
Human mRNA for alpha-catenin, complete c	EMPTY
transducin (beta)-like 1	XM_010334
v-fos FBJ murine osteosarcoma viral onco	AV747778
pumilio (Drosophila) homolog 2	NM_015317
related to the N terminus of tre	AW273166
KIAA0993 protein	N57570
solute carrier family 15 (oligopeptide t	NM_005073
ribosomal protein L17	BG532413
Junk, low PCR	EMPTY
hypothetical protein FLJ23093	NM_024643
Homo sapiens pRGR1 mRNA, partial cds	N48184
ESTs	AW665078
ESTs	AA708964
X-ray repair complementing defective rep	AU139370
Human glucose transporter pseudogene	BF947160
ATPase, vacuolar, 14 kD	BG494658
ectonucleotide pyrophosphatase/phosphodi	XM_004258
FAT tumor suppressor (Drosophila) homolo	NM_005245
Human guanine nucleotide-binding protein	EMPTY
absent in melanoma 1	AU139521

FK506-binding protein 1A (12kD)	AA731598
apolipoprotein A-IV	NM_000482
CD8 antigen, alpha polypeptide (p32)	M12824
hypothetical protein FLJ20558	BG253598
Junk, low PCR	EMPTY
inositol 1,4,5-trisphosphate 3-kinase A	NM_002220
ESTs	AW341105
Junk, low PCR	EMPTY
ESTs, Highly similar to similar to GTPas	D12424
hypothetical protein FLJ12383	AU140727
ubiquitination factor E4B (homologous to	AI912256
ESTs	AI815534
ESTs	AI590607
small nuclear RNA activating complex, po	AV722570
DEK oncogene (DNA binding)	AU152618
FXYD domain-containing ion transport reg	BG166035
Human squalene synthetase (ERG9) mRNA, c	EMPTY
phosphorylase kinase, gamma 2 (testis)	Y11950
src kinase-associated phosphoprotein of	XM_012621
Junk, low PCR	EMPTY
mannosyl (alpha-1,6-)-glycoprotein beta-	U15128
Homo sapiens cDNA: FLJ23020 fis, clone L	BE738204
KIAA0606 protein; SCN Circadian Oscillat	AB011178
tumor protein D52-like 1	NM_003287
ESTs	AA923696
EST, Moderately similar to ALU6_HUMAN AL	AA909818
Homo sapiens chromosome 14 BAC 98L12	AA044197
Junk, low PCR	EMPTY
ESTs	AA620873
transforming growth factor beta-activate	XM_010000
laminin, gamma 2 (nicein (100kD), kalini	AA677534
serine/threonine kinase 10	NM_005990
Junk, low PCR, low PCR	EMPTY
Junk, low PCR	EMPTY
Human mRNA for flavoprotein subunit of c	EMPTY
N-myristoyltransferase 2	NM_004808
uracil-DNA glycosylase 2	XM_011203
similar to ubiquitin binding protein	BG033340
NADH dehydrogenase (ubiquinone) 1 beta s	BE748274
putative selenocysteine lyase	AI640735
glucose phosphate isomerase	AL537100
proteasome (prosome, macropain) subunit,	BG331434
ESTs	AW968630
ESTs	AI741326
hypothetical protein KIAA1165	AB032991
chloride channel Kb	AI311009
hypothetical protein FLJ22418	NM_024626
hemopoietic cell kinase	X58741
zinc finger protein 272	X78931
dynactin 3 (p22)	AW161839
vesicle-associated membrane protein 4	AL035296

lymphocyte cytosolic protein 1 (L-plasti	BF035921
Human translational initiation factor 2	EMPTY
Junk, low PCR	EMPTY
KIAA0602 protein	AL532562
skb1 (S. pombe) homolog	XM_007512
microsomal glutathione S-transferase 1	BF037095
protein kinase related to S. cerevisiae	AL521582
tyrosine 3-monooxygenase/tryptophan 5-mo	BF096115
protein tyrosine phosphatase type IVA, m	NM_003479
KIAA0783 gene product	AA253408
ESTs	AA047703
ESTs	BF448739
ESTs, Weakly similar to ALU1_HUMAN ALU S	AL133982
KIAA1210 protein	AA399312
chromosome condensation-related SMC-asso	BE883590
cyclin I	BG165160
capping protein (actin filament), gelsol	BF978545
Homo sapiens cDNA: FLJ23197 fis, clone R	AI079280
splicing factor, arginine/serine-rich 3	BG287081
Human ADP/ATP translocase mRNA, 3' end,	EMPTY
KIAA1080 protein; Golgi-associated, gamm	BC000284
regulator of G-protein signalling 4	BC000737
acyloxyacyl hydrolase (neutrophil)	NM_001637
casein kinase 1, delta	BE540323
ATPase, Class VI, type 11B	AB023173
proteasome (prosome, macropain) 26S subu	NM_002816
microtubule-associated protein 1A	U38291
ESTs	AA858016
EST	AA342725
ESTs	AA521146
EST	N62332
Junk, low PCR	EMPTY
REMOVED_FROM_DATABASE	Removed
KIAA0676 protein	AB014576
nucleolar phosphoprotein p130	NM_004741
neurogenic differentiation 1	AB018693
erythrocyte membrane protein band 4.2	M30646
Arabadopsis1-125	EMPTY
regulator of G-protein signalling 3	NM_021106
ELL gene (11-19 lysine-rich leukemia gen	AW248332
ESTs	AI821711
MCF.2 cell line derived transforming seq	NM_005369
Junk, low PCR	EMPTY
protein tyrosine phosphatase, receptor t	AU118845
transcription factor 3 (E2A immunoglobul	BF309519
GL002 protein	BF515939
N-deacetylase/N-sulfotransferase (hepara	NM_003635
Homo sapiens clone TCCCIA00164 mRNA sequ	AY007096
DKFZP586F1918 protein	AI685856
KIAA1068 protein	AA476949
spectrin, beta, non-erythrocytic 1	AI580089

Homo sapiens clone 23836 mRNA sequence	AF052120
sperm associated antigen 7	XM_008525
Homo sapiens clone 24790 mRNA sequence	AF052181
CD83 antigen (activated B lymphocytes, i	NM_004233
Arabadopsis3-125	EMPTY
cyclin H	BG531987
fragile histidine triad gene	NM_002012
Homo sapiens cDNA FLJ12830 fis, clone NT	AA731792
activating transcription factor 1	AA393203
proteasome (prosome, macropain) subunit,	BE621845
cleavage stimulation factor, 3' pre-RNA,	NM_001326
Homo sapiens similar to son of sevenless	XM_007270
Link guanine nucleotide exchange factor	AW510496
Junk, low PCR	EMPTY
KIAA0978 protein	N64780
hypothetical protein FLJ20534	AA418905
ESTs	AA213408
disrupted in schizophrenia 1	AA036723
arginine-glutamic acid dipeptide (RE) re	XM_001561
actin related protein 2/3 complex, subun	BG171111
histone deacetylase 6	AL041491
carnitine palmitoyltransferase II	BF026359
Arabadopsis5-125	EMPTY
TATA box binding protein (TBP)-associate	AU129734
NADH dehydrogenase (ubiquinone) Fe-S pro	NM_005006
ESTs	AA922329
transcription factor AP-2 alpha (activat	NM_003220
triosephosphate isomerase 1	BG325897
ankyrin repeat-containing protein	BC002686
postmeiotic segregation increased (S. ce	AU140605
ESTs	AW340089
KIAA0321 protein	AK024743
ESTs	AI697423
Homo sapiens cDNA FLJ12540 fis, clone NT	AI336520
Human chromosome 17q21 mRNA clone 1046:1	AI283313
chemokine (C-C motif) receptor 6	AL121935
protein predicted by clone 23627	AI700717
mucin 5, subtype B, tracheobronchial	AW867962
Cbp/p300-interacting transactivator, wit	AA432143
inositol polyphosphate-1-phosphatase	L08488
Empty	EMPTY
myosin, heavy polypeptide 2, skeletal mu	BF437516
JAK binding protein	AI139920
ESTs	AA416863
H2B histone family, member A	AA541589
NADH dehydrogenase (ubiquinone) Fe-S pro	NM_002496
interleukin 18 receptor 1	NM_003855
lymphocyte-activation gene 3	NM_002286
epidermal growth factor (beta-urogastron	NM_001963
guanylate cyclase activator 2B (uroguany	NM_007102
PC3-96 protein	BF108660

ESTs	AI536606
ESTs	AA252126
APG5 (autophagy 5, S. cerevisiae)-like	NM_004849
keratin 8	AA826807
cartilage paired-class homeoprotein 1	AA425489
hypothetical protein LOC57187	AA581714
iduronate 2-sulfatase (Hunter syndrome)	AI042325
Empty	EMPTY
mitogen-activated protein kinase kinase	AF116604
KIAA0073 protein	BG027629
BANP homolog, SMAR1 homolog	AW958414
nescient helix loop helix 2	M96740
Junk, low PCR	EMPTY
acidic 82 kDa protein mRNA	NM_014597
tumor protein p53-binding protein	AV752368
solute carrier family 6 (neurotransmitte	NM_003044
Junk, low PCR	EMPTY
Homo sapiens, Similar to KIAA0626 gene p	T95311
hypothetical protein FLJ22479	AI887856
40S ribosomal protein S27 isoform	AA927505
CD97 antigen	AI090294
melanoma antigen, family A, 11	NM_005366
AND-1 protein	AK001585
H2A histone family, member Y	BE748949
glioblastoma amplified sequence	BE502193
Empty	EMPTY
myristoylated alanine-rich protein kinas	BE738474
KIAA0061 protein	D31765
Homo sapiens regulator of G-protein sign	N98410
S100 calcium-binding protein A7 (psorias	AW450143
KIAA0341 protein	AB002339
microseminoprotein, beta-	BF964642
integrin, alpha 1	X68742
sucrase-isomaltase	NM_001041
protein phosphatase 1, regulatory (inhib	NM_002711
ESTs	N27332
similar to acetyl-coenzyme A synthetase	AI684951
ESTs	AI668938
PDZ-73 protein	AB006955
double C2-like domains, beta	NM_003585
T-box 5	XM_006833
eukaryotic translation initiation factor	BG258493
transmembrane 4 superfamily member 3	AI886151
sphingosine kinase 1	AI042283
Homo sapiens cDNA: FLJ23296 fis, clone H	AA189085
nucleoporin 88kD	XM_008532
calcium/calmodulin-dependent protein kin	AW327435
SELENOPHOSPHATE SYNTHETASE ; Human sele	XM_005845
ESTs, Highly similar to A42735 ribosomal	AI001784
potassium voltage-gated channel, KQT-lik	AJ006345
zinc finger protein 264	NM_003417

interferon induced transmembrane protein	BG506643
nuclear factor, interleukin 3 regulated	XM_005337
Junk, low PCR	EMPTY
pro-platelet basic protein (includes pla	XM_003506
lipoma HMGIC fusion partner-like 2	AA527516
cleft lip and palate associated transmem	AL531509
CD84 antigen (leukocyte antigen)	NM_003874
kinase insert domain receptor (a type II	AF063658
RAS p21 protein activator (GTPase activa	NM_002890
phospholipid scramblase 1	NM_021105
adenosine deaminase, tRNA-specific 1	AF125188
Junk, low PCR	EMPTY
neuron-specific protein	AW162151
muscleblind (Drosophila)-like	NM_021038
gap junction protein, alpha 4, 37kD (con	XM_001960
Homo sapiens cDNA FLJ12277 fis, clone MA	AK022339
KIAA0429 gene product	NM_014751
prolactin	BG393056
major histocompatibility complex, class	U83582
BCL2/adenovirus E1B 19kD-interacting pro	AI126040
latent transforming growth factor beta b	NM_000627
ATPase, Na+/K+ transporting, beta 2 poly	XM_008232
small inducible cytokine A1 (I-309, homo	NM_002981
cartilage intermediate layer protein, nu	AI342337
UDP-N-acetyl-alpha-D-galactosamine:polyp	X85019
prolylcarboxypeptidase (angiotensinase C	AL540876
nucleolar protein 1 (120kD)	NM_006170
Rac/Cdc42 guanine exchange factor (GEF)	D13631
ESTs, Weakly similar to R32611 1 [H.sapi	AI243808
HDCMC28P protein	AA454635
phosphoribosyl pyrophosphate synthetase	N52940
neural precursor cell expressed, develop	AW960243
zinc finger protein 239	XM_005780
Homo sapiens, clone MGC:13204, mRNA, com	BE262748
catenin (cadherin-associated protein), d	NM_001332
syntaxin 1A (brain)	AL583498
apolipoprotein B (including Ag(x) antige	J02610
KIAA0071 protein	AF070627
SWI/SNF related, matrix associated, acti	BF808164
Junk, low PCR	EMPTY
Junk, low PCR	EMPTY
regulator of G-protein signalling 11	AL539076
collagen, type II, alpha 1 (primary oste	XM_012271
adaptor-related protein complex 4, mu 1	AL530735
phosphoserine aminotransferase	AA978353
natriuretic peptide receptor A/guanylate	AA598841
Junk, low PCR	EMPTY
ESTs, Moderately similar to ALU8_HUMAN A	AI052588
Homo sapiens mRNA; cDNA DKFZp434E0727 (f	N45311
endothelial PAS domain protein 1	AW377189
zinc finger protein 24 (KOX 17)	NM_006965

ESTs	AA194266
LPS-induced TNF-alpha factor	AU117338
sarcoglycan, beta (43kD dystrophin-assoc	R55105
SWI/SNF related, matrix associated, acti	NM_003601
KIAA0404 protein	AB007864
dystonia 1, torsion (autosomal dominant;	AW305016
Junk, low PCR	EMPTY
mannosidase, alpha, class 2A, member 2	BF342980
KIAA0670 protein/acinus	AF124727
Homo sapiens cDNA FLJ14015 fis, clone HE	AI267209
meprin A, beta	NM_005925
kynureninase (L-kynurenine hydrolase)	AV717451
guanylate cyclase 2C (heat stable entero	NM_004963
ESTs	AI652089
PTD002 protein	BE552418
ribonucleotide reductase M1 polypeptide	BE257647
mitogen-activated protein kinase kinase	BE080188
interferon regulatory factor 5	BE562627
DKFZP564O1863 protein	BG337431
HIR (histone cell cycle regulation defec	NM_003325
glycogenin	AL555367
phospholipase C, epsilon	NM_006226
ATP binding protein associated with cell	BF589406
Small proline-rich protein SPRK [human,	AW238522
Junk, low PCR	EMPTY
hypothetical protein MGC5466	BE669926
Rho GDP dissociation inhibitor (GDI) alp	BE563593
ribosomal protein L11	AV761261
ceroid-lipofuscinosis, neuronal 3, juven	AU134339
guanine nucleotide binding protein (G pr	NM_002068
budding uninhibited by benzimidazoles 1	AL517461
ESTs	W88829
ESTs	N62780
glutamic-oxaloacetic transaminase 2, mit	AL560384
Junk, low PCR	EMPTY
Burkitt lymphoma receptor 1, GTP-binding	XM_006463
paired immunoglobulin-like receptor beta	BE646367
renin-binding protein	NM_002910
guanine nucleotide binding protein (G pr	AU132321
RAD9 (S. pombe) homolog	NM_004584
dishevelled 2 (homologous to Drosophila	AL522694
plastin 3 (T isoform)	BF683154
N-sulfoglucosamine sulfohydrolase (sulfa	NM_000199
nucleoporin-like protein 1	NM_007342
kinesin family member 3A	NM_007054
integrin, beta 8	AA569711
Homo sapiens clone 24672 mRNA sequence	AF070633
proprotein convertase subtilisin/kexin t	AI051182
prenylcysteine lyase	N45309
Human mRNA fragment encoding beta-tubuli	EMPTY
hypothetical protein F17127_1	BG055242

heat shock protein hsp70-related protein	AA782088
hypothetical protein MGC4276 similar to	AF038186
EST, Moderately similar to ALU7_HUMAN AL	AA827857
Homo sapiens clone HH114 unknown mRNA	AF114263
ESTs	AI133467
potassium inwardly-rectifying channel, s	NM_004982
ESTs	AA703418
Homo sapiens cDNA FLJ13536 fis, clone PL	N26306
ribonuclease H1	AI149247
phosphatase and tensin homolog (mutated	U93051
ESTs	AA419555
small inducible cytokine subfamily A (Cy	NM_005408
albumin	AL558086
mitogen-activated protein kinase kinase	BE560878
chorionic gonadotropin, beta polypeptide	BG434117
frizzled (Drosophila) homolog 2	NM_001466
Homo sapiens phosphoglycerate mutase (PG	EMPTY
activity-dependent neuroprotective prote	AW614603
ESTs	AW771861
apolipoprotein B mRNA editing enzyme, ca	AI380263
ESTs, Weakly similar to dJ1108D11.1 [H.s	AA135714
methylmalonate-semialdehyde dehydrogenas	AF130089
sparc/osteonectin, cwcv and kazal-like d	AC005213
death-associated protein 6	BG434307
apolipoprotein D	AI188519
Junk, low PCR	EMPTY
KIAA0725 protein	AK023218
golgi SNAP receptor complex member 2	BE729362
Homo sapiens cDNA FLJ11602 fis, clone HE	AA536187
core promoter element binding protein	AU118181
syndecan 4 (amphiglycan, ryudocan)	AI582184
interferon regulatory factor 2	NM_002199
membrane protein CH1	BE466870
Fanconi anemia, complementation group C	XM_005573
Human hydroxymethylglutaryl-CoA lyase mR	EMPTY
protein phosphatase methylesterase-1	BE391938
Junk, low PCR	EMPTY
ankyrin 3, node of Ranvier (ankyrin G)	NM_020987
microfibrillar-associated protein 2	BF435957
ESTs	AI221739
proteasome (prosome, macropain) subunit,	BG540430
Junk, low PCR	EMPTY
lung type-I cell membrane-associated gly	AI277113
platelet-derived growth factor receptor,	M21616
ESTs	AI034385
coagulation factor XI (plasma thrombopla	AF045649
mitogen-activated protein kinase kinase	BF941629
neuronatin	AL574648
interleukin 4	NM_000589
protein phosphatase 4 (formerly X), cata	BE887088
hypothetical protein	AA398519

follistatin-like 3 (secreted glycoprotei	NM_005860
Human DNA repair helicase (ERCC3) mRNA,	EMPTY
Homo sapiens cDNA FLJ13819 fis, clone TH	AA731112
hypothetical protein MGC1136	NM_024025
vesicle-associated membrane protein 2 (s	AU144323
KIAA0036 gene product	BE327043
x 006 protein	BF445491
aconitase 2, mitochondrial	XM_010018
troponin T1, skeletal, slow	AI806543
ESTs	W91975
eukaryotic translation initiation factor	AI498661
protein kinase, cAMP-dependent, catalyti	AW968078
adrenergic, beta-2-, receptor, surface	M15169
ESTs	AI741471
solute carrier family 25 (mitochondrial	BF685676
Junk, low PCR	EMPTY
cell division cycle 2-like 1 (PITSLRE pr	BE268611
ras-like protein	AI829684
double C2-like domains, alpha	BF311314
Unknown - Human Control Plate Well H7	EMPTY
non-kinase Cdc42 effector protein SPEC2	AK025620
ESTs	H08104
histidine triad nucleotide-binding prote	AW967295
Homo sapiens, Similar to RIKEN cDNA 1810	AI668769
ESTs	R61413
dihydropyrimidine dehydrogenase	U20938
polo (Drosophia)-like kinase	BG254810
ESTs	AA149287
zinc finger protein 198	AL138688
v-yes-1 Yamaguchi sarcoma viral related	AA694095
angiotensin receptor 2	NM_000686
DKFZP564D177 protein	AL117557
IMP (inosine monophosphate) dehydrogenas	BF316301
KIAA0256 gene product	BG107450
brain-specific angiogenesis inhibitor 2	NM_001703
Junk, low PCR	EMPTY
zinc finger protein with interaction dom	NM_006626
Unknown - Human Control Plate Well H11	EMPTY
ESTs	AA398512
TATA box binding protein (TBP)-associate	AA557387
cytochrome P450, subfamily XXIV (vitamin	AW022349
solute carrier family 12 (potassium/chlo	BF061743
ESTs	AI741528
fms-related tyrosine kinase 1 (vascular	NM_002019
glutamine-fructose-6-phosphate transamin	NM_002056
22kDa peroxisomal membrane protein-like	BE393339
TATA box binding protein (TBP)-associate	AU118099
RAB27A, member RAS oncogene family	AI468505
lymphoid blast crisis oncogene	BG033405
ADP-ribosylation factor-like 5	AA740582
cyclin C	AI962910

alkaline phosphatase, placental (Regan i	BF807922
cytochrome P450, subfamily VIIB (oxyster	AF029403
KIAA0819 protein	AI017700
jumping translocation breakpoint	AI928081
Empty	EMPTY
TGFB inducible early growth response 2	NM_003597
ESTs, Weakly similar to TC17_HUMAN TRANS	AA975206
thrombospondin 1	NM_003246
KIAA0475 gene product	BE738553
adaptor-related protein complex 2, sigma	BF982854
Junk, low PCR	EMPTY
Junk, low PCR	EMPTY
ESTs	AA406528
advillin	N51826
Junk, low PCR	EMPTY
inositol(myo)-1(or 4)-monophosphatase 1	AL576245
uncharacterized bone marrow protein BM04	N92570
CD74 antigen (invariant polypeptide of m	BG333618
claudin 11 (oligodendrocyte transmembran	AV721095
ryanodine receptor 1 (skeletal)	AW950824
hypothetical protein 628	AL571493
aldehyde dehydrogenase 1 family, member	AI050734
Empty	EMPTY
ESTs	N34849
low density lipoprotein receptor-related	AI167317
transcription factor 12 (HTF4, helix-loo	NM_003205
slit (Drosophila) homolog 3	AI741165
leukemia inhibitory factor receptor	XM_003857
activin A receptor, type IIB	NM_001106
KIAA0189 gene product	NM_014725
ESTs	W02250
ESTs	AI908616
ESTs	AA487202
Junk, low PCR	EMPTY
citron (rho-interacting, serine/threonin	AB023166
ADP-ribosyltransferase 3	AI201027
Junk, low PCR	EMPTY
killer cell lectin-like receptor subfami	NM_006611
general transcription factor IIH, polype	AL569805
sialyltransferase	NM_006456
Empty	EMPTY
Junk, low PCR	EMPTY
Homo sapiens PNAS-138 mRNA, complete cds	AI221507
purinergic receptor P2X, ligand-gated io	AI920988
putative zinc finger protein NY-REN-34 a	AA835489
cadherin 5, type 2, VE-cadherin (vascula	XM_007932
ets variant gene 5 (ets-related molecule	NM_004454
hypothetical protein FLJ11191	AL565661
insulin receptor tyrosine kinase substra	AI243179
LIM domain only 7	AA777749
hypothetical protein FLJ23017	AK022584

prostaglandin I2 (prostacyclin) synthase	AL118525
Homo sapiens C1orf19 mRNA, partial cds	AF288394
leukocyte immunoglobulin-like receptor,	NM_006847
xylulokinase (H. influenzae) homolog	AU123748
endothelial cell growth factor 1 (platel	BG331228
vesicle-associated membrane protein 3 (c	BF038613
POU domain, class 6, transcription facto	NM_002702
Empty	EMPTY
ESTs	N35985
chromodomain protein, Y chromosome-like	BF055337
KIAA0603 gene product	AW731708
ESTs	AA279546
chromodomain helicase DNA binding protei	NM_001272
protein phosphatase 2 (formerly 2A), cat	BF063245
major histocompatibility complex, class	BG327758
ESTs	N73807
ESTs, Weakly similar to T22587 hypotheti	AA521448
apolipoprotein A-I	AA993248
calpastatin	U38525
F-box only protein 8	AL543253
purinergic receptor P2X, ligand-gated io	NM_002560
ribosomal protein L35	AW410089
nuclear transcription factor, X-box bind	AU116819
heparan sulfate 6-O-sulfotransferase	BE870129
protein tyrosine phosphatase, non-recept	AU119325
Empty	EMPTY
ESTs, Weakly similar to A49364 59 protei	BE243545
ESTs, Weakly similar to transmembrane re	AL516579
calnexin	NM_001746
Homo sapiens cDNA FLJ13663 fis, clone PL	AA485349
immunoglobulin superfamily containing le	AL575394
type II Golgi membrane protein	NM_014498
solute carrier family 12 (sodium/chlorid	X91220
prolactin regulatory element binding	AL520029
ESTs	AI479494
secretogranin III	AV753389
phosphatidylinositol glycan, class Q	Z98883
ESTs	AI652114
KIAA0403 protein	AB007863
pre-T/NK cell associated protein	XM_012419
GRO1 oncogene (melanoma growth stimulati	BG491425
tumor necrosis factor receptor superfami	NM_001250
tumor protein 63 kDa with strong homolog	AI122593
Empty	EMPTY
ESTs	AW002681
Homo sapiens cDNA FLJ12641 fis, clone NT	N48003
eukaryotic translation initiation factor	U23028
ESTs	AA250925
inositol(myo)-1(or 4)-monophosphatase 2	BE734192
UDP-Gal:betaGlcNAc beta 1,4- galactosylt	X13223
Junk, low PCR	EMPTY

sarcoglycan, beta (43kD dystrophin-assoc	AI470049
zinc finger protein 173	NM_003449
Homo sapiens serine palmitoyl transferas	AL543565
phosphatidylinositol glycan, class H	AW629157
ESTs	BE812123
signal recognition particle 54kD	AA921832
nuclear receptor co-repressor 2	BF953357
cytochrome P450, subfamily IVF, polypept	XM_012890
prostaglandin E synthase	AI421214
natural killer cell receptor, immunoglob	AW964430
zinc finger protein 175	NM_007147
ESTs	AW450549
methylenetetrahydrofolate dehydrogenase	BC001014
ESTs	BE882535
SEX gene	BF725116
integrin, alpha 5 (fibronectin receptor,	NM_002205
secreted frizzled-related protein 5	NM_003015
SWI/SNF related, matrix associated, acti	AJ011737
iroquois homeobox protein 5	U90304
v-myc avian myelocytomatosis viral oncog	BG256267
peptide chain release factor 3	AA602837
ESTs	AI091432
protein kinase, cAMP-dependent, regulato	AI693297
Junk, low PCR	EMPTY
voltage-dependent anion channel 1	AU129639
phosphatidylinositol glycan, class F	AA043151
Wolfram syndrome 1 (wolframin)	AW589668
carcinoembryonic antigen-related cell ad	X14831
5-methyltetrahydrofolate-homocysteine me	XM_003862
hypothetical protein FLJ20277	BE501346
inositol 1,4,5-triphosphate receptor, ty	XM_010943
Junk, low PCR	EMPTY
KIAA0323 protein	AB002321
neurotrophic tyrosine kinase, receptor,	NM_006180
proline and glutamic acid rich nuclear p	BC002875
cysteine-rich, angiogenic inducer, 61	AL549837
transient receptor potential channel 1	AW949452
Junk, low PCR	EMPTY
hypothetical protein from EUROIMAGE 1977	BE047160
ESTs, Highly similar to immunoglobulin k	BF317113
cisplatin resistance-associated overexpr	XM_008419
ATP synthase, H+ transporting, mitochond	AV763549
kidney- and liver-specific gene	BG400118
Junk, low PCR	EMPTY
hypothetical protein from clone 643	AL513814
excision repair cross-complementing rode	NM_000122
ELL-RELATED RNA POLYMERASE II, ELONGATIC	NM_012081
ESTs	AA056332
putative nucleic acid binding protein RY	N90718
ESTs	BG236383
solute carrier family 24 (sodium/potassi	AF062921

KIAA0350 protein	AI074018
ALL1-fused gene from chromosome 1q	BG498770
small nuclear RNA activating complex, po	NM_003086
KIAA0316 gene product	AB002314
prp28, U5 snRNP 100 kd protein	BC002366
ESTs, Weakly similar to ALU5_HUMAN ALU S	AI589474
ESTs	AI685256
hypothetical protein FLJ20244	AI333215
signal sequence receptor, delta (translo	BF793092
caspase 3, apoptosis-related cysteine pr	AU125557
KIAA1441 protein	AL519335
oxidative-stress responsive 1	AA780605
KIAA0130 gene product	NM_014815
gonadotropin-releasing hormone 1 (leutin	X01059
bone morphogenetic protein receptor, typ	NM_001203
zinc finger protein 207	BE383414
nucleolar cysteine-rich protein	BE221844
Junk, low PCR	EMPTY
KIAA0212 gene product	BG255753
fucosyltransferase 8 (alpha (1,6) fucosy	AF052088
adenomatosis polyposis coli	AA621432
endoplasmic reticulum lumenal protein	BE896199
smg GDS-ASSOCIATED PROTEIN	BG433239
ESTs	AW954965
ESTs, Moderately similar to ALU1_HUMAN A	AV751069
selenophosphate synthetase 2	AA604652
KIAA0087 gene product	NM_014769
Homo sapiens clone 24405 mRNA sequence	AA136361
MAP-kinase activating death domain	NM_003682
KIAA0800 gene product	AI628198
KIAA0052 protein	D29641
ubiquitin carboxyl-terminal esterase L1	AL536004
protein tyrosine phosphatase, non-recept	XM_009511
ubiquitin specific protease 11	BG392606
methyl-CpG binding domain protein 1	BE563133
actinin, alpha 4	AU133641
synaptophysin-like protein	XM_004840
CD5 antigen-like (scavenger receptor cys	NM_005894
tumor necrosis factor (ligand) superfami	NM_000074
Homo sapiens clone 23728 mRNA sequence	AF038199
sarcoglycan, epsilon	NM_003919
ESTs	AA034116
ESTs	AA988599
small proline-rich protein 1B (cornifin)	BE932403
FK506-binding protein 1A (12kD)	BF971255
paraoxonase 2	R14496
cofactor required for Sp1 transcriptiona	AF070563
SEC24 (S. cerevisiae) related gene famil	NM_014822
lactotransferrin	M73700
galactosylceramidase (Krabbe disease)	AU123951
endothelin 3	AL035250

aldehyde dehydrogenase 1 family, member	NM_000692
Junk, low PCR	EMPTY
diphtheria toxin receptor (heparin-bindi	AC004634
Homo sapiens clone 23808 mRNA sequence	H09106
KIAA0534 protein	AB011106
PHD finger protein 1	BF342325
serologically defined colon cancer antig	AF039698
catalase	BG287806
ESTs	AW299520
ESTs, Weakly similar to unnamed protein	AI081356
ELK1, member of ETS oncogene family	AW973479
WNT1 inducible signaling pathway protein	NM_003882
Huntingtin interacting protein B	BE735468
hepatocellular carcinoma antigen gene 52	NM_022097
kallikrein 11	AB013730
collagen, type VII, alpha 1 (epidermolys	XM_003244
Human mRNA for mitochondrial short-chain	EMPTY
general transcription factor IIIC, polyp	NM_001520
growth arrest-specific 1	NM_002048
Junk, low PCR	EMPTY
solute carrier family 25, member 13 (cit	AC002540
t-complex-associated-testis-expressed 1-	NM_006520
ESTs, Weakly similar to myosin phosphata	AI354994
dihydropyrimidinase-like 3	AU133750
hypothetical protein FLJ10525	AK024587
prostaglandin E receptor 2 (subtype EP2)	NM_000956
Junk, low PCR	EMPTY
mitogen-activated protein kinase kinase	R23548
intracellular membrane-associated calciu	AI093502
Homo sapiens brain my050 protein mRNA, c	BF953537
plexin B1	NM_002673
2',5'-oligoadenylate synthetase 1 (40-46	AL582281
Homo sapiens clone 24819 mRNA sequence	AF070574
3-hydroxy-3-methylglutaryl-Coenzyme A sy	XM_001425
Human cytoplasmic beta-actin gene, compl	EMPTY
phosphate regulating gene with homologie	Y08111
transcriptional coactivator	AI088790
Junk, low PCR	EMPTY
Nijmegen breakage syndrome 1 (nibrin)	XM_005310
LanC (bacterial lantibiotic synthetase c	NM_006055
hypothetical protein FLJ20258	BF112023
SWI/SNF related, matrix associated, acti	NM_003074
Cyt19 protein	AI563922
Human clone 23932 mRNA sequence	U79257
Homo sapiens cadherin 15, M-cadherin (my	XM_012533
myo-inositol 1-phosphate synthase A1	AL519005
KIAA1228 protein	AB033054
abl-interactor 12 (SH3-containing protei	AF260261
Norrie disease (pseudoglioma)	AU135898
KIAA0528 gene product	AA649049
Junk, low PCR, low PCR	EMPTY

KIAA0264 protein	AL536034
Human U1 snRNP-specific protein A gene	EMPTY
Homo sapiens cDNA FLJ13496 fis, clone PL	AK025501
inhibin, alpha	AL540575
Homo sapiens unknown mRNA	AI822125
corticotropin releasing hormone receptor	NM_004382
colony stimulating factor 1 receptor, fo	NM_005211
ESTs	BF448096
KIAA0107 gene product	XM_003259
Homo sapiens mRNA; cDNA DKFZp761C082 (fr	AI824328
Junk, low PCR	EMPTY
Human normal keratinocyte mRNA	AK025686
DKFZP434A236 protein	AI636686
hypothetical protein FLJ12903	AA861625
KIAA0798 gene product	XM_012887
acetyl-Coenzyme A acetyltransferase 1 (a	NM_000019
C3H-type zinc finger protein; similar to	BG289426
Junk, low PCR, low PCR	EMPTY
upstream binding transcription factor, R	NM_014233
H.sapiens mRNA for DNA (cytosin-5)-methy	EMPTY
REMOVED_FROM_DATABASE	EMPTY
ESTs	BF671584
ESTs	AA909734
CD48 antigen (B-cell membrane protein)	AL121985
plasminogen activator, tissue	BE394944
hypothetical protein FLJ13352	AA928735
granzyme B (granzyme 2, cytotoxic T-lymp	M57888
Junk, low PCR	EMPTY
small nuclear ribonucleoprotein polypept	AV762663
MAD2 (mitotic arrest deficient, yeast, h	BG527529
ring finger protein 3	BF979824
hypothetical protein FLJ23316	AA526956
piwi (Drosophila)-like 1	AF104260
No ID Incyte EST	EMPTY
platelet-derived growth factor receptor,	AW887370
reticulon 4	BF964327
aldehyde dehydrogenase 7 family, member	NM_001182
Human alpha tubulin	EMPTY
troponin I, cardiac	X90780
brain and reproductive organ-expressed (	AI421704
ESTs	N67991
phosphodiesterase 3B, cGMP-inhibited	NM_000922
Junk, low PCR	EMPTY
ESTs	AI985316
serine (or cysteine) proteinase inhibito	BE812329
ras homolog gene family, member A	AI123587
KIAA0557 protein	BC004125
Junk, low PCR	EMPTY
chromosome 14 open reading frame 4	AA523364
CGI-141 protein	AV696876
fetal hypothetical protein	AW958325

uroplakin 1A	AW629730
Junk, low PCR	EMPTY
Junk, low PCR	EMPTY
keratin 1 (epidermolytic hyperkeratosis)	XM_006848
H.sapiens PMI1 mRNA for phosphomannose i	EMPTY
Junk, low PCR	EMPTY
methylmalonate-semialdehyde dehydrogenas	XM_012348
ESTs	AI580135
ectonucleoside triphosphate diphosphohyd	XM_003296
coatomer protein complex, subunit beta	AK001203
KIAA1641 protein	AW135250
calcium channel, voltage-dependent, gamm	NM_000727
ESTs	AA599294
aryl hydrocarbon receptor nuclear transl	U69202
Homo sapiens clone 161455 breast express	U66048
hypothetical protein DKFZp564D0462	AA442703
cofactor required for Sp1 transcriptiona	AI423909
KIAA0565 gene product	AB011137
replication factor C (activator 1) 1 (14	AW840383
radical fringe (Drosophila) homolog	BF339382
Junk, low PCR	EMPTY
KIAA0353 protein	AB002351
Empty	EMPTY
tyrosine 3-monooxygenase/tryptophan 5-mo	AL556551
D123 gene product	BE792735
centromere protein C 1	NM_001812
replication protein A2 (32kD)	BG333934
endometrial bleeding associated factor (	XM_010640
phosphatidylcholine transfer protein	BE048697
phosphodiesterase 3A, cGMP-inhibited	AJ005036
stress-associated endoplasmic reticulum	AA100925
integrin, alpha M (complement component	NM_000632
KIAA0545 protein	AB011117
ESTs	AA910081
DKFZP434N043 protein	AA426267
AD-017 protein	AI800723
dystrobrevin, alpha	U46746
myeloid/lymphoid or mixed-lineage leukem	M78435
Junk, low PCR	EMPTY
nucleotide-sugar transporter similar to	AW134821
Empty	EMPTY
KIAA0433 protein	NM_015216
Fas (TNFRSF6)-associated via death domai	AA430751
tyrosine 3-monooxygenase/tryptophan 5-mo	AL551616
ets variant gene 2	AF000671
nucleoside phosphorylase	AU142621
ESTs	AI026618
Junk, low PCR	EMPTY
Human DNA sequence from clone RP1-18C9 o	AI498140
programmed cell death 2	BG054774
Homo sapiens cig5 mRNA, partial sequence	AF026941

hypothetical protein FLJ21832	AW136437
hypothetical protein FLJ11269	AW968805
KIAA0710 gene product	BE177965
choroideremia-like (Rab escort protein 2	AL133390
docking protein 2, 56kD	XM_005319
Junk, low PCR	EMPTY
nuclear receptor co-repressor 1	BE502011
Empty	EMPTY
protein tyrosine phosphatase, non-recept	NM_005401
Junk, low PCR	EMPTY
integrin, beta-like 1 (with EGF-like rep	NM_004791
synaptojanin 1	BF109166
RNA binding motif protein 10	AU122630
Homo sapiens mRNA full length insert cDN	AA931319
N-ethylmaleimide-sensitive factor attach	BE904078
ESTs	AI440167
myeloid/lymphoid or mixed-lineage leukem	Z69744
proteasome (prosome, macropain) 26S subu	BE728626
paired immunoglobulin-like receptor beta	AA007559
ESTs	AA004211
B-cell CLL/lymphoma 1	Z23022
occludin	NM_002538
Junk, low PCR	EMPTY
Junk, low PCR	EMPTY
yeast Sec31p homolog	NM_016211
Empty	EMPTY
ATPase, Cu++ transporting, alpha polypep	XM_013141
TNF receptor-associated factor 6	NM_004620
proteasome (prosome, macropain) 26S subu	XM_012340
myocilin, trabecular meshwork inducible	NM_000261
Junk, low PCR	EMPTY
Homo sapiens cDNA FLJ13542 fis, clone PL	AA720894
N-methylpurine-DNA glycosylase	AL527544
hypothetical protein FLJ12969	AA992143
restin (Reed-Steinberg cell-expressed in	NM_002956
Junk, low PCR	EMPTY
ESTs	AA443725
Homo sapiens chromosome 9 open reading f	AI803293
putative membrane protein	AA678046
collagen, type IV, alpha 3 (Goodpasture	M81379
regulator of G-protein signalling 7	AL566862
myosin regulatory light chain 2, smooth	AW085196
HSKM-B protein	AI222092
Empty	EMPTY
adenosine monophosphate deaminase 2 (iso	XM_002152
KIAA0156 gene product	NM_014706
DEAD/H (Asp-Glu-Ala-Asp/His) box polypep	AF279891
KIAA0231 protein	D86984
Junk, low PCR	EMPTY
ESTs	AA057365
EphA1	Z27409

KIAA0998 protein	AA533049
syntaxin 8	BG253163
Junk, low PCR	EMPTY
putative protein-tyrosine kinase	AA938871
ESTs	AA280220
chromosome condensation 1-like	AK023010
No ID Incyte EST	EMPTY
ribosomal protein L13	BG331404
Junk, low PCR	EMPTY
KIAA0455 gene product	AB007924
Empty	EMPTY
bone morphogenetic protein 4	BE612406
nuclear receptor subfamily 4, group A, m	AI819173
transmembrane trafficking protein	BE738423
fetal Alzheimer antigen	NM_004459
KIAA0339 gene product	AB002337
ankyrin 2, neuronal	AA975079
ectodermal-neural cortex (with BTB-like	BC000418
butyrobetaine (gamma), 2-oxoglutarate di	R41886
Rhesus blood group-associated glycoprote	AU121299
NADH dehydrogenase (ubiquinone) Fe-S pro	BF791786
ESTs	T83063
ESTs	BE673931
KIAA0740 gene product	BE934846
Dombrock blood group	X95826
adenylate cyclase activating polypeptide	NM_001118
Junk, low PCR	EMPTY
immunoglobulin superfamily, member 4	NM_014333
ESTs	H17304
steroid dehydrogenase homolog	AF078850
runt-related transcription factor 3	XM_001616
RNA binding motif protein 3	AU143092
carbonic anhydrase I	AI791402
potassium inwardly-rectifying channel, s	AA946757
Junk, low PCR	EMPTY
Junk, low PCR	EMPTY
trinucleotide repeat containing 4	AF284423
H1 histone family, member 2	AI127167
KIAA0205 gene product	NM_014873
Ewing sarcoma breakpoint region 1	NM_005243
bullous pemphigoid antigen 1 (230/240kD)	M69225
trinucleotide repeat containing 12	BG475196
immunoglobulin kappa constant	AW407113
Homo sapiens cDNA FLJ13847 fis, clone TH	AK023909
Junk, low PCR	EMPTY
collagen, type IV, alpha 4	XM_010841
hypothetical protein FLJ12701	AA657536
Homo sapiens mRNA; cDNA DKFZp434I1216 (f	AA442451
Junk, low PCR	EMPTY
insulin-like growth factor 2 (somatomedi	AW411300
Junk, low PCR	EMPTY

hypothetical protein FLJ20608	AI031800
Golgi apparatus protein 1	AK027032
nephroblastoma overexpressed gene	NM_002514
topoisomerase (DNA) I	NM_003286
JM4 protein	AI096619
mucin 5, subtype B, tracheobronchial	Y09788
axin	AF009674
JM5 protein	BE392434
MADS box transcription enhancer factor 2	AI952550
cytochrome c oxidase subunit VIa polypep	NM_005205
T-cell leukemia/lymphoma 1A	BE268420
ephrin-B2	AI127370
TAT-INTERACTIVE PROTEIN, 72-KD	BC003154
lysophospholipase II	AL162101
Homo sapiens mRNA; cDNA DKFZp586K1721 (f	AI199131
FBJ murine osteosarcoma viral oncogene h	AU118031
general transcription factor IIB	BG426106
glutaredoxin (thioltransferase)	BG399074
Junk, low PCR	EMPTY
aggrecan 1 (chondroitin sulfate proteogl	NM_001135
spermidine synthase	BF026320
small inducible cytokine subfamily A (Cy	AV734258
carboxylesterase 2 (intestine, liver)	NM_003869
lipase, gastric	XM_005871
myotubularin related protein 3	AF233437
signal transducer and activator of trans	AA478534
inositol 1,4,5-trisphosphate 3-kinase B	AA737679
FXYD domain-containing ion transport reg	AW959802
hypothetical protein A-211C6.1	AL537232
HIV-1 Rev binding protein	AA969897
NADH dehydrogenase (ubiquinone) Fe-S pro	AF013160
ESTs, Weakly similar to ALU1_HUMAN ALU S	AI732395
collagen, type V, alpha 3	AI740960
Junk, low PCR	EMPTY
pim-1 oncogene	NM_002648
zinc finger protein 145 (Kruppel-like, e	AF060568
ESTs, Weakly similar to rho-type GTPase-	AA046743
interleukin 6 (interferon, beta 2)	M54894
KIAA0014 gene product	NM_014665
tumor necrosis factor (ligand) superfami	AW022060
tetracycline transporter-like protein	L11669
DNA fragmentation factor, 45 kD, alpha p	BF309422
phosphoprotein enriched in astrocytes 15	XM_001279
sema domain, immunoglobulin domain (Ig),	AV724223
adenosine monophosphate deaminase 2 (iso	BE778615
integral membrane protein 2B	NM_021999
G protein-coupled receptor 9	XM_010135
Homo sapiens insulin-degrading enzyme (I	XM_005890
solute carrier family 6 (neurotransmitte	NM_001045
ESTs	AW071615
ESTs	T05686

FSHD region gene 1	T41211
lectin, galactoside-binding, soluble, 3	BG334264
Junk, low PCR	EMPTY
ESTs	N57273
huntingtin (Huntington disease)	BF834293
TATA box binding protein (TBP)-associate	BE882192
KIAA0421 protein	AB007881
Junk, low PCR	EMPTY
low density lipoprotein receptor defect	AA476831
adducin 1 (alpha)	D44640
fasciculation and elongation protein zet	BE891611
MUF1 protein	BC004953
H4 histone family, member D	AI494175
downregulated in ovarian cancer 1	NM_014890
papillary renal cell carcinoma (transloc	BG481089
thrombospondin 4	NM_003248
ESTs	AA843915
hypothetical protein MGC2477	T49802
RAS guanyl releasing protein 2 (calcium	U78170
cyclin-dependent kinase inhibitor 2A (me	AI803069
guanine nucleotide binding protein (G pr	XM_003265
hypothetical protein FLJ21047	AA293625
collagen, type XI, alpha 1	XM_001849
insulinoma-associated 1	BE793964
ribosomal protein S6 kinase, 90kD, polyp	AL022069
forkhead box M1	AL525810
Junk, low PCR	EMPTY
Homo sapiens mRNA; cDNA DKFZp547E184 (fr	AF038201
KIAA0111 gene product	AL568756
Mad4 homolog	AL040187
Rho GDP dissociation inhibitor (GDI) alp	AL576133
KIAA0423 protein	AB007883
DNA segment, single copy probe LNS-CAI/L	BE301711
RAB31, member RAS oncogene family	AI521354
Human coatomer protein (HEPCOP) mRNA, co	EMPTY
actinin, alpha 2	AA582234
ESTs	H54558
glutathione transferase zeta 1 (maleylac	AL556458
eukaryotic translation elongation factor	AI689610
Junk, low PCR	EMPTY
tousled-like kinase 2	BG432591
caspase 5, apoptosis-related cysteine pr	NM_004347
mannosyl (beta-1,4-)-glycoprotein beta-1	AI914746
nuclear factor of kappa light polypeptid	AI935157
Homo sapiens cDNA FLJ20767 fis, clone CO	AI074716
zinc finger protein 195	NM_007152
plasmolipin	Z38289
DiGeorge syndrome critical region gene D	AW163428
chymotrypsin C (caldecrin)	NM_007272
interferon stimulated gene (20kD)	NM_002201
interleukin 13 receptor, alpha 1	NM_001560

catenin (cadherin-associated protein), b	NM_001904
Human alpha-N-acetylgalactosaminidase mR	EMPTY
transducin (beta)-like 2	AK026529
SH3 protein	AW245943
PRP4/STK/WD splicing factor	NM_004697
ESTs	BF477698
ESTs	BG484509
integrin, alpha 6	NM_000210
intercellular adhesion molecule 2	AW577907
molybdopterin synthase sulfurylase	AI570446
KIAA0036 gene product	NM_014642
hypothetical protein FLJ12549	BF589346
HtrA-like serine protease	AL577683
ESTs	AI536733
hepatocyte growth factor-regulated tyros	AI417306
myosin, light polypeptide 6, alkali, smo	AV716792
paraoxonase 2	AU138383
DKFZP566I1024 protein	BF887879
calcium channel, voltage-dependent, alph	Z84492
Human capping protein alpha mRNA, partia	EMPTY
CUG triplet repeat, RNA-binding protein	BE410460
ESTs	AI653368
KDEL (Lys-Asp-Glu-Leu) endoplasmic retic	Z97056
glioma tumor suppressor candidate region	AA290865
No ID Incyte EST	EMPTY
arginine vasopressin receptor 1A	XM_006934
splicing factor 3b, subunit 4, 49kD	BG032727
ESTs, Weakly similar to ALU1_HUMAN ALU S	AA927288
KIAA0174 gene product	BG403408
proliferation-associated 2G4, 38kD	BE042986
Junk, low PCR	EMPTY
Homo sapiens, Similar to hypothetical pr	AV724266
hypothetical protein MGC2650	AI885381
cholinergic receptor, nicotinic, beta po	NM_000749
kallikrein 10	BF837996
myosin, heavy polypeptide 9, non-muscle	BF304650
pregnancy-associated plasma protein A	BG256949
Human topoisomerase I mRNA, complete cds	EMPTY
hypothetical protein DKFZp762O076	BF967957
KIAA0653 protein, B7-like protein	AI421115
KIAA0303 protein	AB002301
ESTs, Weakly similar to NPL4_HUMAN NUCLE	AA626170
PFTAIRE protein kinase 1	AA954361
minichromosome maintenance deficient (S.	BE250461
Junk, low PCR	EMPTY
ESTs	AI652919
Homo sapiens clone 23763 unknown mRNA, p	BE894483
eukaryotic translation initiation factor	AA403154
protein tyrosine phosphatase, receptor t	NM_007050
thyroid receptor interacting protein 15	AW295348
G antigen 2	NM_001472

Homo sapiens integrin, beta 1 (fibronect	BE880168
mitogen-activated protein kinase kinase	AI299943
heterogeneous nuclear ribonucleoprotein	BE904546
dihydrolipoamide S-acetyltransferase (E2	Y00978
Lactate Dehydrogenase A	EMPTY
G protein coupled receptor interacting p	AA552144
LPAP for lysophosphatidic acid phosphata	AW518020
small nuclear ribonucleoprotein D3 polyp	BF220008
PRO1073 protein	AA233775
ESTs	AI610676
tubulin, beta, 5	AL536237
T54 protein	BF220170
ESTs	AI282277
cadherin 4, type 1, R-cadherin (retinal)	AL109911
zinc finger protein 185 (LIM domain)	XM_010082
EphA7	NM_004440
ESTs, Moderately similar to R3HU12 ribos	AA813267
neuroblastoma, suppression of tumorigeni	BE908218
major histocompatibility complex, class	BF974114
myosin IB	AL546537
serine (or cysteine) proteinase inhibito	BE856006
aspartate beta-hydroxylase	S83325
Human MRL3 mRNA for ribosomal protein L3	EMPTY
Homo sapiens cDNA: FLJ22182 fis, clone H	AA482223
ESTs	AA574440
nuclear VCP-like	NM_002533
KIAA1588 protein	AI278552
Homo sapiens clone IMAGE:1963178, mRNA s	AF339785
protein kinase, cGMP-dependent, type II	NM_006259
Junk, low PCR	EMPTY
lipopolysaccharide specific response-7 p	AA634565
sarcolipin	XM_006331
TLS-associated serine-arginine protein 2	AU121116
Junk, low PCR	EMPTY
KIAA1237 protein	AW192581
paired-like homeodomain transcription fa	BG330708
gamma-aminobutyric acid (GABA) A recepto	NM_000808
ADP-ribosylation factor 4-like	BG545770
copine I	AW867967
methionine-tRNA synthetase	BE410528
Arabadopsis7-62.5	EMPTY
eukaryotic translation elongation factor	BE799451
cell cycle related kinase	BE467048
inter-alpha (globulin) inhibitor, H2 pol	NM_002216
Homo sapiens, clone IMAGE:3354845, mRNA,	AA897744
adenylate cyclase 3	XM_010843
adenosine A2b receptor	AL553646
peptide YY	NM_004160
ESTs	AI214437
ESTs	BG027062
elaC (E.coli) homolog 1	AA524364

sodium channel, nonvoltage-gated 1 alpha	NM_001038
hypothetical protein FLJ13258 similar to	BE300676
serine/threonine kinase 15	NM_003600
hypothetical protein DKFZp434P0116	AB015331
homeo box B6	AJ270993
H.sapiens gene from PAC 747L4	AL035297
No ID Incyte EST	EMPTY
Arabadopsis9-62.5	EMPTY
ribosomal protein L41	NM_021104
ESTs	AA086224
chloride intracellular channel 1	AA291390
RecQ protein-like 5	AI123482
HMT1 (hnRNP methyltransferase, S. cerevi	BG430177
Bet1 (S. cerevisiae) homolog	AW452133
adenosine A3 receptor	X76981
DKFZP434N043 protein	AL080143
phosphatidylinositol glycan, class Q	AI287286
baculoviral IAP repeat-containing 3	AI417860
KIAA0040 gene product	AW963093
ESTs	BG167914
v-myb avian myeloblastosis viral oncogen	BG328514
nuclear factor I/X (CCAAT-binding transc	D51465
replication factor C (activator 1) 5 (36	AL532485
glutathione peroxidase 2 (gastrointestin	AI626059
mitogen-activated protein kinase-activat	XM_007011
Empty	EMPTY
Junk, low PCR	EMPTY
Homo sapiens cDNA FLJ11918 fis, clone HE	AA810748
sialyltransferase 8 (alpha-2, 8-polysial	NM_005668
hypothetical protein	AA435912
zinc finger protein 273	AU132789
phosphoprotein regulated by mitogenic pa	AU125722
proline-rich protein BstNI subfamily 2	K03208
Homo sapiens cDNA FLJ12198 fis, clone MA	AI445726
protein phosphatase 1B (formerly 2C), ma	AW074517
Homo sapiens cDNA FLJ10196 fis, clone HE	AA460239
G-rich RNA sequence binding factor 1	BF034561
Junk, low PCR	EMPTY
Cdc42 effector protein 2	AF163840
CMRF35 leukocyte immunoglobulin-like rec	NM_006678
B-cell CLL/lymphoma 9	AL543085
F-box only protein 7	AA429862
protein phosphatase 6, catalytic subunit	NM_002721
Empty	EMPTY
NADH dehydrogenase (ubiquinone) 1 beta s	BE391452
hypothetical protein FLJ12934	AA809485
calcium channel, voltage-dependent, beta	AB054985
hypothetical protein FLJ12707	AL138078
S100 calcium-binding protein A4 (calcium	AV713833
phospholipase D1, phophatidylcholine-spe	NM_002662
ADP-ribosylation factor-like 1	AL560086

No ID Incyte EST	EMPTY
myosin 5C	AI916670
hypothetical protein	AI301320
D-dopachrome tautomerase	BG478996
hypothetical protein FLJ10052	AI417581
dishevelled 1 (homologous to Drosophila	AF006011
nucleophosmin (nucleolar phosphoprotein	AL547236
3-hydroxy-3-methylglutaryl-Coenzyme A sy	AA305679
DNA segment on chromosome 21 (unique) 20	AL526119
ATPase, H+ transporting, lysosomal (vacu	NM_001690
Empty	EMPTY
ESTs	AA868747
ESTs	AA421078
pleckstrin homology, Sec7 and coiled/coi	AI538459
RNA binding motif protein 8B	AW276137
ribosomal protein L35	BF310946
v-abl Abelson murine leukemia viral onco	NM_007314
Junk, low PCR	EMPTY
carbonic anhydrase X	H23176
No ID Incyte EST	EMPTY
ribosomal protein L37a	AI707851
interleukin enhancer binding factor 3, 9	AF007140
Human DNA sequence from clone 747H23 on	AI300201
ribosomal protein L23a	AA857067
T-box 1	BE468028
SWI/SNF related, matrix associated, acti	R56503
sushi-repeat-containing protein, X chrom	NM_006307
transglutaminase 1 (K polypeptide epider	NM_000359
Empty	EMPTY
ESTs	AA917800
long fatty acyl-CoA synthetase 2 gene	AV705292
N-myc downstream regulated	AL530564
lanosterol synthase (2,3-oxidosqualene-l	AI248897
3-hydroxybutyrate dehydrogenase (heart,	AA834609
phospholamban	BF574758
Machado-Joseph disease (spinocerebellar	NM_004993
ESTs	R71449
DKFZP434F091 protein	AA460671
Homo sapiens, Similar to RIKEN cDNA 2310	BG036183
paralemmin	XM_009280
ESTs, Weakly similar to calphotin [D.mel	AW172460
spondyloepiphyseal dysplasia, late, pseu	NM_015890
lipoprotein, Lp(a)	NM_005577
calcitonin/calcitonin-related polypeptid	X03662
Junk, low PCR	EMPTY
ATP-dependent RNA helicase	BE466582
polymerase (RNA) III (DNA directed) (62k	AL558755
EST	AI025099
guanine nucleotide binding protein-like	BE798151
ESTs	AI057233
hydroxyprostaglandin dehydrogenase 15-(N	AL542647

tyrosine kinase with immunoglobulin and	NM_005424
extra spindle poles, S. cerevisiae, homo	NM_012291
meprin A, alpha (PABA peptide hydrolase)	NM_005588
E74-like factor 4 (ets domain transcript	AL136450
Junk, low PCR	EMPTY
putative N6-DNA-methyltransferase	AI016585
ESTs	AI400463
Ac-like transposable element	XM_010415
transglutaminase 2 (C polypeptide, prote	AL552373
YDD19 protein	BG289236
membrane interacting protein of RGS16	U91321
TEA domain family member 3	AI571142
annexin A7	NM_004034
SWI/SNF related, matrix associated, acti	BE254018
KIAA1688 protein	AW207595
M-phase phosphoprotein 9	AA843678
No ID Incyte EST	EMPTY
Homo sapiens germline mRNA sequence	L35592
tumor protein D52	NM_005079
KIAA0414 protein	NM_014007
acid phosphatase 2, lysosomal	BE745075
golgi autoantigen, golgin subfamily a, 1	AW005258
keratin 4	AW265621
ESTs	AW973003
ESTs	N66954
hypothetical protein FLJ11191	AI376803
potassium voltage-gated channel, Shaw-re	BE797730
regulatory factor X, 2 (influences HLA c	BF893497
CTD (carboxy-terminal domina, RNA polyme	NM_004715
hypothetical protein FLJ10377	NM_018077
histone deacetylase 5	BF305705
phosphatidic acid phosphatase type 2B	AW131816
guanine nucleotide binding protein (G pr	AW024699
Homo sapiens mRNA from chromosome 5q21-2	AB002449
Junk, low PCR	EMPTY
KIAA0173 gene product	AL043927
zinc finger protein 133 (clone pHZ-13)	AW055275
zinc finger protein	AW954552
symplekin; Huntingtin interacting protei	BG472289
damage-specific DNA binding protein 2 (4	NM_000107
annexin A8	XM_005878
ESTs	AA994554
hypothetical protein FLJ10430	XM_007962
solute carrier family 6 (neurotransmitte	XM_006999
No ID Incyte EST	EMPTY
syndecan 4 (amphiglycan, ryudocan)	NM_002999
WD40 protein Ciao1	BG251014
casein kinase 1, epsilon	AW192876
multiple PDZ domain protein	AJ001319
transcription factor Dp-1	NM_007111
DKFZP564G2022 protein	W72795

Homo sapiens cDNA: FLJ21930 fis, clone H	U90916
Junk, low PCR	EMPTY
Junk, low PCR	EMPTY
acidic protein rich in leucines	BF307256
serine (or cysteine) proteinase inhibito	NM_000624
developmentally regulated GTP-binding pr	AL543480
neuropeptide Y receptor Y6 (pseudogene)	NM_006173
enolase 3, (beta, muscle)	NM_001976
ESTs	AA706003
ESTs	AI026691
KIAA0246 protein	NM_015136
ESTs	BF514243
fibroblast growth factor 13	XM_010269
GCN1 (general control of amino-acid synt	BG403565
VAMP (vesicle-associated membrane protei	N38854
carbonic anhydrase IV	XM_008313
plakophilin 4	NM_003628
ESTs, Moderately similar to ALU7_HUMAN A	AI291128
casein kinase 2, alpha prime polypeptide	BF000288
Junk, low PCR	EMPTY
keratin 15	AU121430
putative breast adenocarcinoma marker (3	BF570351
RAD54 (S.cerevisiae)-like	NM_003579
spastic paraplegia 7, paraplegin (pure a	BF570017
CDC20 (cell division cycle 20, S. cerevi	BG256659
intercellular adhesion molecule 1 (CD54)	M24283
ESTs	BF054973
ESTs	AI439534
hypothetical SBBI03 protein	AY007109
Homo sapiens clone 24820 mRNA sequence	AF070547
ESTs	AW611957
Homo sapiens mRNA; cDNA DKFZp586F2224 (f	AI655015
KIAA0863 protein	AK023032
homeo box B13	AI884491
poly(rC)-binding protein 1	BF337301
hypothetical protein	AA883734
hexokinase 2	BG492090
hypothetical protein FLJ12649	AA815048
kinesin family member 5B	NM_004521
NIPSNAP, C. elegans, homolog 1	BG107345
Junk, low PCR	EMPTY
aminolevulinate, delta-, synthase 2 (sid	NM_000032
CDP-diacylglycerol synthase (phosphatida	AI635747
placental growth factor, vascular endoth	BF431974
ESTs	AW844818
SWI/SNF related, matrix associated, acti	BF222740
Junk, low PCR	EMPTY
solute carrier family 21 (organic anion	AF085224
ATPase, Na+/K+ transporting, alpha 3 pol	AL538608
ESTs	BE328009
hypothetical protein P1 p373c6	BE549809

ribonuclease/angiogenin inhibitor	AL577761
Human ADP-ribosylation factor (ARF3) mRN	EMPTY
aldo-keto reductase family 1, member D1	XM_011122
receptor-interacting serine-threonine ki	BG170405
ESTs	AA495928
Junk, low PCR	EMPTY
KIAA0247 gene product	NM_014734
No ID Incyte EST	EMPTY
complement component 5 receptor 1 (C5a l	AL573453
suppressor of fused	BF439552
sphingomyelin phosphodiesterase 1, acid	NM_000543
splicing factor proline/glutamine rich (	BG251495
ESTs	AA148085
fucosyltransferase 4 (alpha (1,3) fucosy	AI370004
Homo sapiens clone 24566 mRNA sequence	BF732853
Rho GTPase activating protein 8	BE091961
ets variant gene 6 (TEL oncogene)	BE217961
tryptophan hydroxylase (tryptophan 5-mon	AA719435
TGFB inducible early growth response	AV714317
H.sapiens mRNA for splicing factor SF3a1	EMPTY
interleukin 8 receptor, beta	AA480683
amylase, alpha 2B; pancreatic	XM_010549
Junk, low PCR	EMPTY
chemokine-like receptor 1	U79526
Breakpoint cluster region protein, uteri	AF044774
ret finger protein	AA583188
EphA4	NM_004438
ESTs	AI004188
tumor necrosis factor receptor superfami	BE386504
spleen tyrosine kinase	NM_003177
Homo sapiens cDNA FLJ14201 fis, clone NT	AU138357
DnaJ (Hsp40) homolog, subfamily A, membe	AL567229
leukemia-associated phosphoprotein p18 (	AL575997
ADP-ribosylation factor domain protein 1	AF230399
dystrobrevin, alpha	AW513384
Junk, low PCR, low PCR	EMPTY
B-cell CLL/lymphoma 3	NM_005178
Human mRNA for platelet-type phosphofruc	EMPTY
central cannabinoid receptor	AF107262
discs, large (Drosophila) homolog 2 (cha	NM_001364
Junk, low PCR	EMPTY
Junk, low PCR	EMPTY
deoxyribonuclease I-like 1	NM_006730
hypothetical protein	W72641
type 1 tumor necrosis factor receptor sh	AW665276
KIAA1373 protein	AW294479
potassium inwardly-rectifying channel, s	BG288548
CD34 antigen	AL035091
hypothetical protein FLJ10718	BF182778
HMT1 (hnRNP methyltransferase, S. cerevi	BF110626
mutS (E. coli) homolog 4	AF104243

crystallin, alpha A	XM_009788
receptor (calcitonin) activity modifying	BG036385
Junk, low PCR, low PCR	EMPTY
pre-B-cell colony-enhancing factor	BG110735
Human eukaryotic initiation factor 2B-ep	EMPTY
hypothetical protein	AF007130
telomeric repeat binding factor 2	BF982638
Junk, low PCR	EMPTY
Junk, low PCR	EMPTY
nitrogen fixation cluster-like	AA904951
ESTs	AW440572
HMG-box containing protein 1	AK025284
ESTs, Weakly similar to YOJ1_CAEEL HYPOT	AI308071
PPAR binding protein	NM_004774
isocitrate dehydrogenase 3 (NAD+) beta	BG475573
Homo sapiens clone TCCCIA00427 mRNA sequ	BF224021
Junk, low PCR	EMPTY
G protein-coupled receptor 19	NM_006143
retinol dehydrogenase 5 (11-cisand 9-cis	BE221672
surfeit 5	AL527070
prostate cancer overexpressed gene 1	AA228367
rTS beta protein	NM_017512
Human dihydrolipoamide dehydrogenase mRN	EMPTY
Chediak-Higashi syndrome 1	NM_000081
formyl peptide receptor-like 1	BG541691
Junk, low PCR	EMPTY
lamin B2	AU130032
peroxisomal farnesylated protein	AL571857
hypothetical protein FLJ22004	AA423854
somatostatin	AW583834
PTPRF interacting protein, binding prote	AA459628
phosphodiesterase 4C, cAMP-specific (dun	U66349
transmembrane 7 superfamily member 2	AL556984
ESTs	AA977532
Homo sapiens EST from clone 491476, full	AL355685
Junk, low PCR	EMPTY
No ID Incyte EST	EMPTY
hephaestin	AW960584
Junk, low PCR, low PCR	EMPTY
integrin, beta 2 (antigen CD18 (p95), ly	NM_000211
Human ADP/ATP translocase mRNA, 3' end,	EMPTY
Junk, low PCR	EMPTY
nuclear DNA-binding protein	AI274315
Junk, low PCR	EMPTY
CD3G antigen, gamma polypeptide (TiT3 co	AU122431
Junk, low PCR	EMPTY
Homo sapiens mRNA; cDNA DKFZp762G207 (fr	AI344283
Junk, low PCR	EMPTY
Junk, low PCR	EMPTY
myxovirus (influenza) resistance 1, homo	BF972585
UV-B repressed sequence, HUR 7	X98307

ESTs	AI821362
ESTs	BE221193
sialyltransferase 8C (alpha2,3Galbeta1,4	XM_008782
KIAA0507 protein	AB007976
E4F transcription factor 1	XM_012510
Junk, low PCR, low PCR	EMPTY
enolase 2, (gamma, neuronal)	BG169625
Arabadopsis1-62.5	EMPTY
karyopherin alpha 3 (importin alpha 4)	NM_002267
interleukin 1 receptor antagonist	BG288796
solute carrier family 5 (inositol transp	AI867198
sal (Drosophila)-like 2	AB002358
Junk, low PCR	EMPTY
hypothetical protein KIAA1335	BE674356
TU3A protein	AU134947
ESTs	AI022463
holocytochrome c synthase (cytochrome c	AW951033
Junk, low PCR	EMPTY
chromosome 21 open reading frame 35	AW242517
hypothetical protein from EUROIMAGE 3636	BF059615
Homo sapiens cDNA: FLJ22071 fis, clone H	AA236923
Homo sapiens ankyrin-like with transmemb	XM_005187
Homo sapiens chromosome 9, P1 clone 1165	AC004472
ectonucleoside triphosphate diphosphohyd	AI927632
DnaJ (Hsp40) homolog, subfamily C, membe	AI418894
Arabadopsis3-62.5	EMPTY
LIM domain-containing preferred transloc	XM_011029
hypothetical protein PP591	AI636333
Homo sapiens clone 23570 mRNA sequence	AF038202
Junk, low PCR	EMPTY
granzyme K (serine protease, granzyme 3;	AA947915
Junk, low PCR	EMPTY
replication factor C (activator 1) 5 (36	AL525471
ESTs	AA844712
putative methyltransferase	BG287883
Junk, low PCR	EMPTY
No ID Incyte EST	EMPTY
ESTs	W52563
beta-1,3-glucuronyltransferase 3 (glucur	XM_006048
ATP-binding cassette, sub-family C (CFTR	AI074459
glutathione S-transferase A3	N40887
Junk, low PCR	EMPTY
KIAA0759 protein	AA527389
Arabadopsis5-62.5	EMPTY
SH3-containing protein SH3GLB2	BE256828
DKFZP727M231 protein	AL117480
tropomyosin 2 (beta)	AA283746
Junk, low PCR	EMPTY
kinectin 1 (kinesin receptor)	Z22551
ESTs	AA844438
Junk, low PCR	EMPTY

ESTs	AI129587
integrin beta 4 binding protein	BG283650
KIAA0118 protein	AF091035
hepatocellular carcinoma-associated anti	BE790903
ESTs	AI479352
KIAA0706 gene product	XM_008526
epididymal secretory protein (19.5kD)	BG397837
thyroid hormone receptor interactor 10	BE743785
Junk, low PCR	EMPTY
KIAA0692 protein	AU133410
Empty	EMPTY
E74-like factor 1 (ets domain transcript	AL559590
putative chemokine receptor; GTP-binding	AA903016
neurotensin	BG283732
gamma-glutamyl carboxylase	BE326952
retinoblastoma-binding protein 6	AA991554
Homo sapiens, clone IMAGE:3458173, mRNA,	AA843628
GM2 ganglioside activator protein	XM_003987
heat shock factor binding protein 1	W51947
isocitrate dehydrogenase 2 (NADP+), mito	AL545953
ubiquinol-cytochrome c reductase (6.4kD)	AW163237
ESTs	AA719822
No ID Incyte EST	EMPTY
chitinase 1 (chitotriosidase)	AI097512
Junk, low PCR	EMPTY
BLu protein	AI921941
T-box 19	N73939
hypothetical protein MGC3077	AA543045
Empty	EMPTY
splicing factor, arginine/serine-rich (t	BC000160
LIM domain only 7	AW953043
secreted frizzled-related protein 4	XM_004706
exportin, tRNA (nuclear export receptor	NM_007235
hypothetical protein FLJ11149	AF038172
zinc finger protein 131 (clone pHZ-10)	AI184913
A kinase (PRKA) anchor protein 5	NM_004857
hypothetical protein FLJ20736	AK024786
chloride channel 4	NM_001830
signal sequence receptor, beta (transloc	BE887942
CD209 antigen	AI288509
hypothetical protein FLJ11011	AW779265
apoptosis inhibitor 5	AW504175
poliovirus receptor	NM_006505
transporter similar to yeast MRS2	BF508841
Junk, low PCR	EMPTY
paraneoplastic antigen MA1	XM_012347
Empty	EMPTY
Junk, low PCR	EMPTY
casein kinase 1, alpha 1	AW504120
aldehyde dehydrogenase 5 family, member	L34820
suppressor of var1 (S.cerevisiae) 3-like	BE784830

EphA5	X95425
ESTs	AL566989
apolipoprotein A-II	AV682594
Homo sapiens, clone IMAGE:3448343, mRNA,	AW167097
guanylate binding protein 2, interferon-	NM_004120
degenerative spermatocyte (homolog Droso	BE467911
KIAA1553 protein	AK026996
hypothetical protein FLJ13110	AL080222
A kinase (PRKA) anchor protein (yotiao)	NM_005751
acidic epididymal glycoprotein-like 1	X95238
peroxisomal biogenesis factor 14	AI167893
Junk, low PCR	EMPTY
PRKC, apoptosis, WT1, regulator	AI547265
myeloid/lymphoid or mixed-lineage leukem	AI742296
vimentin	AL571953
KIAA1138 protein	AW628917
zinc finger protein ZNF140-like protein	BF055237
Junk, low PCR	EMPTY
Junk, low PCR	EMPTY
ESTs	AA016306
Junk, low PCR	EMPTY
Junk, low PCR	EMPTY
ESTs	N51056
keratin 2A (epidermal ichthyosis bullosa	NM_000423
No ID Incyte EST	EMPTY
NCK adaptor protein 1	AI591085
pancreatic elastase IIB	NM_015849
prostate stem cell antigen	NM_005672
Notch (Drosophila) homolog 4	D63395
uridine monophosphate kinase	AA470769
jagged 2	AF111170
clathrin, light polypeptide (Lca)	AU150900
ubiquitin protein ligase E3A (human papi	AW503947
hypothetical protein FLJ20327	BG235988
Homo sapiens mRNA; cDNA DKFZp434M229 (fr	AA862533
contactin associated protein 1	NM_003632
hemopexin	AI133162
hypothetical C2H2 zinc finger protein FL	AW241587
T brachyury (mouse) homolog	NM_003181
apyrase, lysosomal	AB002390
Homo sapiens mRNA; cDNA DKFZp566M063 (fr	AA255478
lymphocyte antigen 117	AF000426
Junk, low PCR	EMPTY
calcium channel, voltage-dependent, L ty	Z26308
osteoglycin (osteoinductive factor, mime	AI088387
mesoderm specific transcript (mouse) hom	AV693992
matrin 3	NM_018834
Kelch-like ECH-associated protein 1	BC003156
transcription factor AP-2 beta (activati	AL049693
erythrocyte membrane protein band 4.1 (e	BF868865
fibroblast growth factor receptor 3 (ach	AI078769

RAB22A, member RAS oncogene family	N73955
hypothetical protein FLJ20343	AL519357
gene near HD on 4p16.3 with homology to	AK025692
mitogen-activated protein kinase 7	AL563390
KIAA0804 protein	AW273420
Junk, low PCR	EMPTY
cytochrome P450, subfamily IIIA, polypep	AI114634
eukaryotic translation initiation factor	AI354797
tuberous sclerosis 1 (hamartin)	AF013168
tropomodulin 3 (ubiquitous)	BF222010
stem-loop (histone) binding protein	AW150631
cadherin 2, type 1, N-cadherin (neuronal	X57548
UDP glycosyltransferase 2 family, polype	NM_001073
G protein-coupled receptor	U67784
PTK6 protein tyrosine kinase 6	BE563232
astrotactin	AB006627
imprinted in Prader-Willi syndrome	U12897
Homo sapiens cDNA: FLJ21449 fis, clone C	AK025102
small nuclear RNA activating complex, po	AA628164
rab6 GTPase activating protein (GAP and	BF344490
interferon regulatory factor 1	NM_002198
growth associated protein 43	AL567524
ESTs	AA608634
DEAD/H (Asp-Glu-Ala-Asp/His) box polypep	NM_004660
DEAD/H (Asp-Glu-Ala-Asp/His) box polypep	NM_004396
son of sevenless (Drosophila) homolog 1	AA574131
regulator of G-protein signalling 16	NM_002928
ESTs, Weakly similar to fatty acid omega	R53457
poliovirus receptor-related 1 (herpesvir	BF036447
retinoblastoma 1 (including osteosarcoma	NM_000321
hypothetical protein FLJ10520	AL041526
polymerase (DNA directed), delta 1, cata	BE274988
dentatorubral-pallidoluysian atrophy (at	BG424682
secretogranin II (chromogranin C)	NM_003469
AXL receptor tyrosine kinase	NM_021913
reticulon 1	BF345503
hypothetical protein FLJ20764	AI889219
Homo sapiens mRNA; cDNA DKFZp564K142 (fr	AA046836
KIAA0116 protein	AW614427
EphA2	AI568135
protein similar to E.coli yhdg and R. ca	AI242551
GTP-binding protein Rho7	AI554560
fibroblast growth factor receptor 2 (bac	NM_022971
Homo sapiens mRNA full length insert cDN	AA670046
Sp3 transcription factor	X68560
ESTs, Weakly similar to A42210 alpha-1-m	AI052516
T cell receptor beta locus	AA311658
squalene epoxidase	AF098865
hypothetical protein P1 p373c6	XM_011495
Fzr1 protein	BF309617
histone deacetylase 2	BG035543

peroxiredoxin 2	AL523978
utrophin (homologous to dystrophin)	NM_007124
succinate dehydrogenase complex, subunit	AI537920
ESTs, Moderately similar to ALU1_HUMAN A	AW082759
Homo sapiens cDNA FLJ13446 fis, clone PL	AW025290
KIAA0556 protein	AB011128
cathepsin H	XM_007633
ESTs	N32429
karyopherin beta 2b, transportin	XM_009068
KIAA0005 gene product	NM_014670
Homo sapiens mRNA; cDNA DKFZp564L0822 (f	AI351523
Homo sapiens clone 23785 mRNA sequence	AF035307
Junk, low PCR	EMPTY
actin binding protein; macrophin (microf	XM_010728
protein tyrosine phosphatase, receptor t	AK023850
CCR4-NOT transcription complex, subunit	NM_014516
cathepsin S	AL544870
E2F transcription factor 5, p130-binding	AI566856
upstream regulatory element binding prot	AF161390
Homo sapiens creatine kinase B mRNA, com	EMPTY
myotubular myopathy 1	AW950692
defender against cell death 1	AV714320
ESTs, Weakly similar to tuftelin [M.musc	AA150200
flavin containing monooxygenase 4	AL031274
thyroid transcription factor 1	BE671319
similar to DNA-directed RNA polymerase I	BC004882
ATP-binding cassette, sub-family B (MDR/	AW949716
tripartite motif protein TRIM2	BG163696
Homo sapiens, clone IMAGE:3353119, mRNA,	BE302221
EST	T66813
desmoglein 1	XM_008810
ESTs	AI459175
S100 calcium-binding protein A2	AA593632
Junk, low PCR	EMPTY
cysteine desulfurase	AV704896
P311 protein	AF119859
mitogen-activated protein kinase kinase	NM_030662
Homo sapiens phosphoglycerate mutase (PG	EMPTY
paraoxonase 3	BF513914
POU domain, class 2, transcription facto	NM_002697
hypothetical protein FLJ20283	AI205635
paired box gene 6 (aniridia, keratitis)	XM_012065
phosphatidylinositol 4-kinase, catalytic	BG025389
ESTs	AW206247
solute carrier family 4, sodium bicarbon	AF053755
hook2 protein	BF348626
ESTs	R44477
LIM protein (similar to rat protein kina	AA196322
adenylate cyclase 9	AI248827
ESTs	AA007626
mannosidase, alpha, class 1A, member 1	AU119410

caveolin 1, caveolae protein, 22kD	BG541572
hypothetical protein FLJ12242	NM_024681
KIAA0068 protein	D38549
discs, large (Drosophila) homolog 5	NM_004747
Human IMP dehydrogenase type 1 mRNA comp	EMPTY
cytochrome b-245, beta polypeptide (chro	X04011
hypothetical protein	BG331987
Homo sapiens clone 24421 mRNA sequence	AA593775
SMC1 (structural maintenance of chromoso	NM_006306
tyrosine 3-monooxygenase/tryptophan 5-mo	AU120828
syntaxin 1A (brain)	AA522687
sodium channel, voltage-gated, type IV,	NM_000334
cathepsin W (lymphopain)	AL540662
Junk, low PCR	EMPTY
ESTs	AA044642
Homo sapiens mRNA for partial 3'UTR, seq	BG054543
ribosomal protein L18	AW664584
protein phosphatase 3 (formerly 2B), reg	AL580466
KIAA0476 gene product	AL133753
proteasome (prosome, macropain) 26S subu	AA604027
cholecystokinin	XM_003225
platelet-activating factor acetylhydrola	NM_000437
Human DNA repair helicase (ERCC3) mRNA,	EMPTY
phosphatidic acid phosphatase type 2C	BF445055
Junk, low PCR	EMPTY
ribosomal protein S6 kinase, 90kD, polyp	AW068493
protein tyrosine phosphatase, non-recept	U12128
RAB5A, member RAS oncogene family	BC001267
hypothetical protein MGC3222	BE710919
heat shock 70kD protein 6 (HSP70B')	NM_002155
cadherin 3, type 1, P-cadherin (placenta	XM_007891
gamma-aminobutyric acid (GABA) A recepto	AW517530
DKFZP564D177 protein	AW168826
ESTs	AW020663
Homo sapiens cDNA FLJ13558 fis, clone PL	AA976778
PTK7 protein tyrosine kinase 7	AL157486
butyrophilin, subfamily 3, member A2	BF882131
RAN binding protein 8	XM_006935
Junk, low PCR	EMPTY
hypothetical protein FLJ20030	AI589003
Unknown - Human Control Plate Well H7	EMPTY
pericentrin	AB007862
Junk, low PCR	EMPTY
ESTs	AA650148
heterogeneous nuclear ribonucleoprotein	AL516486
small inducible cytokine subfamily D (Cy	AL529472
Homo sapiens cDNA: FLJ23117 fis, clone L	AV744804
Junk, low PCR	EMPTY
transcription factor NRF	Y07707
ESTs	N92652
Human DNA sequence from clone 261K5 on c	AL050350

ESTs	AW316760
ESTs	AI500009
KIAA0704 protein	AY008372
class-I MHC-restricted T cell associated	XM_006313
Rho guanine exchange factor (GEF) 16	BG328764
MpV17 transgene, murine homolog, glomeru	AI074843
von Hippel-Lindau syndrome	NM_000551
Unknown - Human Control Plate Well H11	EMPTY
active BCR-related gene	BG250875
Junk, low PCR	EMPTY
ESTs	AI273177
N-acetyltransferase 1 (arylamine N-acety	R79401
lectin, galactoside-binding, soluble, 1	BF977413
ESTs	AW955047
KIAA0308 protein	AB002306
period (Drosophila) homolog 2	BF508974
stromal cell-derived factor 2	BF196663
ESTs	N98696
hypothetical protein FLJ20366	XM_005157
hypothetical protein FLJ12553	NM_024700
lysyl oxidase-like 1	AI963477
S100 calcium-binding protein A5	AW090645
retinoid X receptor, alpha	XM_011778
peripheral benzodiazepine receptor-assoc	AB014512
SEC24 (S. cerevisiae) related gene famil	NM_004922
Empty	EMPTY
Human mRNA for SB classII histocompatibi	AV759427
MAD (mothers against decapentaplegic, Dr	NM_005901
ESTs	AI269928
Junk, low PCR	EMPTY
gene with multiple splice variants near	NM_003704
Homo sapiens cDNA FLJ11727 fis, clone HE	AI566139
DHHC1 protein	AW269604
ribosomal protein S10	AV757346
B7 protein	XM_006973
ESTs	N71691
Junk, low PCR	EMPTY
Ste-20 related kinase	AL546457
Down syndrome critical region gene 3	BE326803
Rho guanine exchange factor (GEF) 16	BE391191
FAT tumor suppressor (Drosophila) homolo	AL157443
ras homolog gene family, member H	BG257364
proteasome (prosome, macropain) 26S subu	AA191445
Empty	EMPTY
yes-associated protein 65 kDa	AA433865
hypothetical protein FLJ23188	AA291644
solute carrier family 21 (organic anion	AA806497
defensin, alpha 6, Paneth cell-specific	XM_005296
Junk, low PCR	EMPTY
adaptor-related protein complex 1, gamma	BF059254
hypothetical protein FLJ13164	AU143256

Homo sapiens cDNA: FLJ23260 fis, clone C	U90911
KIAA0336 gene product	NM_014635
ESTs	AW968618
reticulocalbin 2, EF-hand calcium bindin	AL120373
Human DNA sequence from clone RP11-108L7	AW673741
keratin, hair, acidic,3B	Y16789
hypothetical protein R31240_1	AI435954
Junk, low PCR	EMPTY
CCAAT/enhancer binding protein (C/EBP),	BF343807
protein phosphatase 1, regulatory subuni	BG105725
Empty	EMPTY
Junk, low PCR	EMPTY
Junk, low PCR	EMPTY
3-hydroxy-3-methylglutaryl-Coenzyme A sy	AW117731
SRY (sex determining region Y)-box 22	AL034548
tubulin, gamma 1	BC000619
ESTs	AW973290
ubiquitination factor E4B (homologous to	AW304977
ATP-binding cassette, sub-family C (CFTR	U91318
KIAA0573 protein	AB011145
hypothetical protein FLJ10928	BF593164
fibroblast growth factor 2 (basic)	NM_002006
hypothetical protein FLJ21845	AW301700
Vertebrate LIN7 homolog 1, Tax interacti	AA442886
gamma-glutamyltransferase-like activity	XM_009881
insulin-like growth factor 2 (somatomedi	BC000939
Homo sapiens clone 24694 mRNA sequence	AF070620
nuclear protein, ataxia-telangiectasia l	D83243
Empty	EMPTY
Junk, low PCR	EMPTY
origin recognition complex, subunit 6 (y	AU160162
Homo sapiens mRNA; cDNA DKFZp434A2417 (f	AI915221
cyclin-dependent kinase inhibitor 1A (p2	L26165
phosphofructokinase, muscle	BE780960
hypothetical protein FLJ10008	AK024113
protocadherin 20	AF169693
ATP-binding cassette, sub-family F (GCN2	BE891494
phenylalanine hydroxylase	NM_000277
Human glucose transporter pseudogene	N66384
hydroxyacyl-Coenzyme A dehydrogenase/3-k	BG477867
ESTs	AI949876
SH3-containing protein SH3GLB1	AA436295
non-metastatic cells 5, protein expresse	AL043778
KIAA0508 protein	AB007977
Werner syndrome	AA287923
RAD51 (S. cerevisiae) homolog C	BC000667
Empty	EMPTY
sperm associated antigen 9	BE566830
Huntingtin-interacting protein A	AW511804
ferritin, light polypeptide	AI092317
Wiskott-Aldrich syndrome protein interac	NM_003387

ATX1 (antioxidant protein 1, yeast) homo	AI276280
ESTs	AW292120
ESTs	AA876616
amiloride-sensitive cation channel 3, te	XM_004681
tumor necrosis factor (ligand) superfami	AI908454
phospholipid scramblase 3	AF159442
RAN binding protein 1	BE728883
ESTs	BF574959
protein tyrosine phosphatase, receptor t	NM_003626
leucine rich repeat (in FLII) interactin	BG261015
Human mRNA for ZFM1 protein alternativel	D26121
polyamine-modulated factor 1	BF968865
MYC-associated zinc finger protein (puri	BG260604
Empty	EMPTY
Junk, low PCR	EMPTY
hypothetical protein FLJ22127	AI929630
hypothetical protein FLJ10140	AI273359
Junk, low PCR	EMPTY
KIAA0326 protein	AK026115
putative glycolipid transfer protein	AU153213
putative prostate cancer susceptibility	BE858252
zinc finger protein 162	AL555297
gamma-aminobutyric acid (GABA) A recepto	NM_000814
Homo sapiens cDNA FLJ10041 fis, clone HE	AW205601
heterogeneous nuclear ribonucleoprotein	NM_002137
Homo sapiens mRNA full length insert cDN	AA406526
hypothetical protein	AI888578
hypothetical protein FLJ10339	BE780989
dynamin 2	BG423048
tumor necrosis factor receptor superfami	AI239571
B melanoma antigen	NM_001187
putative cyclin G1 interacting protein	BG281971
arginyl aminopeptidase (aminopeptidase B	AL390139
transforming, acidic coiled-coil contain	AW514948
Junk, low PCR	EMPTY
heat shock transcription factor 2	BC005329
ESTs	N99610
period (Drosophila) homolog 1	BE615751
cytochrome c oxidase subunit IV	AI061652
N-acetylgalactosaminidase, alpha-	AA759294
ESTs	AA013274
ESTs	AW665482
No ID Incyte EST	EMPTY
zinc finger protein 195	AW025438
mitochondrial ribosomal protein L3	AA580433
ribosome binding protein 1 (dog 180kD ho	BE646396
proteasome (prosome, macropain) inhibito	NM_006814
matrix metalloproteinase 10 (stromelysin	BE740153
protein tyrosine phosphatase, non-recept	NM_002835
RAN binding protein 2-like 1	AI859613
solute carrier family 3 (activators of d	AL567017

hypothetical protein FLJ12806	AW274012
Junk, low PCR	EMPTY
regulator of G-protein signalling 12	AF030109
slit (Drosophila) homolog 1	AI937520
KIAA0378 protein	AB002376
stromal cell-derived factor 2	BG431313
tumor necrosis factor receptor superfami	BC001281
ESTs	H48664
Junk, low PCR	EMPTY
adaptor protein containing pH domain, PT	AA555008
transforming growth factor, beta 2	AW303586
KIAA0239 protein	AJ251833
sialic acid binding Ig-like lectin 5	NM_003830
Junk, low PCR	EMPTY
phosphodiesterase 2A, cGMP-stimulated	XM_006369
glutamate receptor, ionotropic, kainate	U16125
forkhead box G1B	XM_007233
eukaryotic translation initiation factor	AU120178
Junk, low PCR	EMPTY
Williams-Beuren syndrome chromosome regi	BE531346
solute carrier family 7 (cationic amino	NM_003046
hypothetical protein MGC4707	AI936193
integrin, beta 3 (platelet glycoprotein	NM_000212
serine/threonine kinase 13 (aurora/IPL1-	AI564072
Junk, low PCR	EMPTY
Homo sapiens cDNA: FLJ21635 fis, clone C	R38925
leucine zipper-EF-hand containing transm	AI702539
sphingosine-1-phosphatase	AI581085
G protein-coupled receptor 51	AF056085
palmitoyl-protein thioesterase 2	AI025037
Nck, Ash and phospholipase C binding pro	AB005216
POP7 (processing of precursor, S. cerevi	BE206450
annexin A11	AL547276
mitogen-activated protein kinase 4	XM_008806
insulin-like growth factor-binding prote	M62403
guanosine monophosphate reductase	AL533154
interleukin 22 receptor	BF509148
translocating chain-associating membrane	AL523342
recombination activating gene 1	NM_000448
ligatin	BF445670
spindle pole body protein	AW518581
Human TB1 gene mRNA, 3' end	AA723646
RNA-binding protein gene with multiple s	NM_006867
ESTs	AI656113
Homo sapiens cDNA FLJ11570 fis, clone HE	AA233707
gemin4	AV713188
immediate early response 3	N32077
KIAA0095 gene product	AU131118
karyopherin alpha 6 (importin alpha 7)	U68730
amphiregulin (schwannoma-derived growth	AL546917
lipoprotein lipase	NM_000237

ATP-binding cassette, sub-family C (CFTR	AF085692
phosphatidylinositol-4-phosphate 5-kinas	AB011161
serum amyloid A2	AA829286
ESTs	AW592254
tumor differentially expressed 1	AL552738
flavin containing monooxygenase 2	AL021026
ESTs	N52073
Homo sapiens clone 23700 mRNA sequence	AF038185
acid phosphatase 1, soluble	AL573837
lysophospholipase-like	BG331714
Junk, low PCR	EMPTY
L13 protein	AA989508
PTD017 protein	AA152202
TED protein	NM_015686
lymphotoxin beta (TNF superfamily, membe	AW188005
somatostatin receptor 2	NM_001050
inhibitor of growth family, member 1-lik	AI160411
coagulation factor XIII, A1 polypeptide	XM_004467
caspase 9, apoptosis-related cysteine pr	AB015653
progesterone binding protein	AW192720
Junk, low PCR	EMPTY
ESTs	AW139612
leupaxin	NM_004811
microsomal triglyceride transfer protein	AL563954
ESTs	AA053722
Junk, low PCR	EMPTY
advanced glycosylation end product-speci	U89336
phosphatidylinositol glycan, class K	AI926246
hypoxia-inducible protein 2	AL537916
hypothetical protein FLJ13117	AU155482
hypothetical protein FLJ10687	AU152152
ribosomal protein L10a	BF975849
coilin	AI825820
PIBF1 gene product	AI758409
ESTs	AI142842
transcription elongation factor B (SIII)	AI312889
protease, serine, 2 (trypsin 2)	BE969948
H.sapiens mRNA for elongation factor-1-g	EMPTY
envoplakin	NM_001988
zinc finger protein 177	NM_003451
Homo sapiens clone DT1P1B6 mRNA, CAG rep	AI912004
tryptophanyl-tRNA synthetase	BF795451
hypothetical protein FLJ12820	NM_022451
DNA segment, single copy, probe pH4 (tra	NM_005436
ring finger protein 10	BG338102
Homo sapiens cDNA: FLJ21718 fis, clone C	AK025371
B-cell CLL/lymphoma 11A (zinc finger pro	XM_002695
ESTs	AI077541
ESTs	AI653002
copine III	AF077226
KIAA0050 gene product	NM_014716

small inducible cytokine A7 (monocyte ch	XM_012649
Homo sapiens mRNA; cDNA DKFZp564I112 (fr	AW452604
Fukuyama type congenital muscular dystro	BE467704
small acidic protein	AA164983
Human mRNA for calmodulin	EMPTY
leukocyte-associated Ig-like receptor 1	AL575990
eukaryotic translation elongation factor	BF243409
dolichyl-diphosphooligosaccharide-protei	BG519732
propionyl Coenzyme A carboxylase, alpha	NM_000282
hepatocellular carcinoma-associated anti	AB032992
primase, polypeptide 2A (58kD)	NM_000947
3-hydroxyanthranilate 3,4-dioxygenase	NM_012205
ESTs	AA483426
KIAA1615 protein	AA234930
ESTs	AW195399
CDV-1 protein	H99645
DKFZP564N1363 protein	AW300985
surfeit 5	BE205842
apical protein, Xenopus laevis-like	NM_001649
CGI-01 protein	AF132936
LIM and SH3 protein 1	AW189528
calcitonin receptor-like	U17473
Human U1 snRNP-specific protein A gene	EMPTY
lecithin-cholesterol acyltransferase	NM_000229
Sjogren's syndrome/scleroderma autoantig	NM_006396
Friedreich ataxia	AA363205
MHC class II transactivator	NM_000246
ESTs, Moderately similar to ALU7_HUMAN A	AI042148
cAMP responsive element binding protein-	U89337
cell division cycle 25B	AL109804
ESTs	AW117243
Junk, low PCR	EMPTY
Homo sapiens colon cancer-associated ant	AI584082
zinc finger protein 255	N99348
Junk, low PCR	EMPTY
No ID Incyte EST	EMPTY
Down syndrome cell adhesion molecule	AL163281
KIAA0771 protein	AB018314
purinergic receptor P2Y, G-protein coupl	AJ006945
tryptase beta 1	M33494
H.sapiens mRNA for DNA (cytosin-5)-methy	EMPTY
cathepsin B	AL546380
insulin-like growth factor 1 (somatomedi	M11568
Junk, low PCR	EMPTY
frizzled (Drosophila) homolog 6	BG180759
hypothetical protein FLJ20337	AI279972
corticotropin releasing hormone	AW513199
cAMP responsive element binding protein	R68639
ESTs	AA219088
Homo sapiens cDNA FLJ20769 fis, clone CO	AK000776
ESTs, Weakly similar to CGHU7L collagen	AW183487

protein kinase C, theta	AW780437
Junk, low PCR	EMPTY
Homo sapiens integrin, beta 1 (fibronect	AW513695
G protein-coupled receptor kinase 2 (Dro	NM_005307
KIAA0795 protein	AB018338
EGF-like-domain, multiple 5	AB011542
fatty-acid-Coenzyme A ligase, long-chain	BE889785
Human alpha tubulin	EMPTY
NADH dehydrogenase (ubiquinone) 1 beta s	BG492618
regulator of mitotic spindle assembly 1	AU139950
RNA binding motif, single stranded inter	NM_016836
ELK3, ETS-domain protein (SRF accessory	XM_006890
leucine zipper protein 1	H98657
Junk, low PCR	EMPTY
Homo sapiens clone 23767 and 23782 mRNA	BF037093
ESTs	AA847550
5(3)-deoxyribonucleotidase (dNT-2); nucl	XM_008173
ESTs	AI963740
ESTs, Weakly similar to (defline not ava	AI243872
nuclear receptor co-repressor 1	AA468619
interferon-stimulated transcription fact	BF525953
zinc finger protein 91 (HPF7, HTF10)	AU123049
polycystic kidney disease 2-like 1	AF094827
hypothetical protein	BG180936
parathyroid hormone receptor 1	NM_000316
Human mRNA (HA0643) for ORF (Canis oligo	EMPTY
immunoglobulin superfamily, member 1	AV727120
KIAA0605 gene product	BE062792
KIAA0431 protein	BG105308
dynein, cytoplasmic, heavy polypeptide 1	AB002323
hypothetical protein FLJ20605	AI080050
zinc finger protein 143 (clone pHZ-1)	AF071771
protein tyrosine phosphatase, receptor t	AF152378
leptin receptor	AW302522
ESTs, Weakly similar to A46010 X-linked	AA278841
putative ankyrin-repeat containing prote	AA621188
ESTs, Weakly similar to T12519 hypotheti	AI393577
Junk, low PCR	EMPTY
ribosomal protein S6 kinase, 70kD, polyp	M60725
collagen, type X, alpha 1 (Schmid metaph	X72579
No ID Incyte EST	EMPTY
Junk, low PCR	EMPTY
mitogen-activated protein kinase-activat	NM_004759
Empty	EMPTY
stomatin-like 2	BE314702
chloride channel Kb	XM_002085
ESTs	BF343036
peroxisomal long-chain acyl-coA thioeste	AA825544
Junk, low PCR	EMPTY
death-associated protein	BG292296
pancreatic lipase-related protein 2	XM_005975

Junk, low PCR	EMPTY
KIAA0352 gene product	NM_014830
calsyntenin-2	AW206575
SAR1 protein	AI086814
Breakpoint cluster region protein, uteri	BF477399
Norrie disease (pseudoglioma)	XM_010262
fer (fps/fes related) tyrosine kinase (p	NM_005246
solute carrier family 6 (neurotransmitte	XM_003866
chromosome 8 open reading frame 1	AI688382
villin 2 (ezrin)	AU136810
Empty	EMPTY
Junk, low PCR	EMPTY
phospholipid transfer protein	AU139503
ESTs	BE503917
tumor necrosis factor receptor superfami	AA987627
optic atrophy 1 (autosomal dominant)	AB011139
translin-associated factor X	BG504997
solute carrier family 26 (sulfate transp	NM_000112
Homo sapiens clone IMAGE:119716, mRNA se	AF339768
KIAA0535 gene product	AI741795
Homo sapiens clone 022f05 My030 protein	AW613587
N-acetylglucosamine-phosphate mutase	AU154539
KIAA0634 protein	AW409774
silver (mouse homolog) like	BF978444
neuropeptide Y receptor Y2	U42766
interleukin 11	AI620537
RNA helicase-related protein	AL571412
gene predicted from cDNA with a complete	AL522814
Empty	EMPTY
nuclear receptor subfamily 3, group C, m	AU139241
COX10 (yeast) homolog, cytochrome c oxid	AU132781
ESTs	AA621267
ARP1 (actin-related protein 1, yeast) ho	BE905357
tumor necrosis factor receptor superfami	AL542093
estrogen receptor 1	XM_011463
Kell blood group	NM_000420
glucokinase (hexokinase 4) regulatory pr	NM_001486
Rhesus blood group, D antigen	X63094
ESTs	AI093668
ESTs	N24490
ESTs	N48365
glycoprotein hormones, alpha polypeptide	AU137688
dUTP pyrophosphatase	AW276291
RAB11B, member RAS oncogene family	AL574150
similar to S. cerevisiae Sec6p and R. no	AF055006
spermine synthase	BE880227
Empty	EMPTY
adipose differentiation-related protein	AX025098
midkine (neurite growth-promoting factor	AW966037
zinc finger protein 265	AU153893
laminin receptor 1 (67kD, ribosomal prot	BF973196

Junk, low PCR	EMPTY
retinoblastoma-binding protein 2	NM_005056
growth differentiation factor 1	AI936592
Junk, low PCR	EMPTY
phosphatidylinositol-4-phosphate 5-kinas	BF530414
KIAA1163 protein	AA758688
DKFZP564M112 protein	AA889506
EST	N25920
RAS guanyl releasing protein 1 (calcium	AA278696
Homo sapiens clone 24739 mRNA sequence	AF070571
butyrophilin, subfamily 2, member A2	AA877627
DnaJ (Hsp40) homolog, subfamily B, membe	AV729634
thioredoxin reductase 1	BE787478
Empty	EMPTY
Nef-associated factor 1	NM_006058
fructose-1,6-bisphosphatase 1	L10320
protein associated with PRK1	AW955122
syndecan 2 (heparan sulfate proteoglycan	AW951095
ADP-ribosylation factor-like 6 interacti	BG399003
cytosolic ovarian carcinoma antigen 1	NM_006375
heparin-binding growth factor binding pr	BE747141
forkhead box E1 (thyroid transcription f	U89995
farnesyl-diphosphate farnesyltransferase	X69141
ESTs, Moderately similar to ALU7_HUMAN A	AA877123
Homo sapiens mRNA; cDNA DKFZp761E2216 (f	BE350967
Homo sapiens cDNA FLJ11245 fis, clone PL	AA235116
Homo sapiens calcium channel, voltage-de	AI417964
tocopherol (alpha) transfer protein (ata	NM_000370
T cell receptor beta locus	AF043179
RAN binding protein 7	BF000473
platelet/endothelial cell adhesion molec	M37780
Empty	EMPTY
aquaporin 1 (channel-forming integral pr	NM_000385
potassium inwardly-rectifying channel, s	U65406
NADH dehydrogenase (ubiquinone) Fe-S pro	BE797420
transporter protein; system N1 Na+ and H	NM_006841
Human clone CE29 7.2 (CAC)n/(GTG)n repea	U00954
rabaptin-5	NM_004703
phosphomannomutase 2	BG477402
cadherin 18, type 2	NM_004934
spectrin, beta, erythrocytic (includes s	AW207639
ESTs	AI150360
Homo sapiens cDNA: FLJ22696 fis, clone H	AA424109
No IDI ncyte EST	EMPTY
immunoglobulin superfamily, member 6	AA557260
deleted in azoospermia-like	NM_001351
ESTs, Highly similar to AC006014 8 simil	AV727808
nel (chicken)-like 2	XM_010930
Homo sapiens GT212 mRNA	L38935
CD1B antigen, b polypeptide	AA983871
Junk, low PCR	EMPTY

platelet derived growth factor C	BF002799
Human DNA sequence from clone RP1-12G14	AW194419
KIAA0328 protein	AB002326
fibroblast growth factor receptor 1 (fms	NM_023111
KIAA1194 protein	W47579
Wiskott-Aldrich syndrome-like	XM_004867
neutral sphingomyelinase (N-SMase) activ	NM_003580
ESTs	AI796794
KIAA0417 gene product	AB007877
KIAA1339 protein	AB037760
ATPase, Ca++ transporting, plasma membra	X63575
Human clone 23614 mRNA sequence	AI091322
death effector domain-containing	AA931258
Homo sapiens cDNA FLJ10447 fis, clone NT	H15124
eukaryotic translation termination facto	BE817697
serine (or cysteine) proteinase inhibito	NM_006919
homogentisate 1,2-dioxygenase (homogenti	NM_000187
quiescin Q6	NM_002826
Homo sapiens, Similar to KIAA0843 protei	AA479885
chromosome 12 open reading frame 5	AA897774
PAX transcription activation domain inte	U80735
Lysosomal-associated multispanning membr	NM_006762
ESTs, Highly similar to NAALADase L prot	N52534
FK506-binding protein 1B (12.6 kD)	BC002614
RAD51 (S. cerevisiae) homolog (E coli Re	AL524788
hypothetical protein FLJ13386	BE327246
ubiquitin-conjugating enzyme E2G 2 (homo	XM_009804
hypothetical protein SP329	AF177339
Human BRCA2 region, mRNA sequence CG011	U50536
transducin-like enhancer of split 1, hom	NM_005077
p53-induced protein	AL542839
KIAA0770 protein	AL117456
phosphoribosyl pyrophosphate synthetase	AU117327
coagulation factor II (thrombin) recepto	M62424
reticulocalbin 1, EF-hand calcium bindin	NM_002901
matrix metalloproteinase 2 (gelatinase A	XM_012503
ESTs	AI860245
KIAA0721 protein	N70239
polymerase (RNA) mitochondrial (DNA dire	NM_005035
elastase 3, pancreatic (protease E)	D00306
U6 snRNA-associated Sm-like protein	AK024217
phosphatidylinositol (4,5) bisphosphate	AF187891
oculocutaneous albinism II (pink-eye dil	AI097486
Junk, low PCR	EMPTY
death associated transcription factor 1	AL035669
ESTs	AW820889
hexose-6-phosphate dehydrogenase (glucos	Z98044
KIAA0076 gene product	AI754198
cleavage stimulation factor, 3' pre-RNA,	NM_001325
protein kinase, AMP-activated, alpha 2 c	AL035705
Junk, low PCR	EMPTY

ubiquitin-activating enzyme E1-like	NM_003335
eukaryotic translation elongation factor	AU145072
Junk, low PCR	EMPTY
Homo sapiens cDNA FLJ11658 fis, clone HE	AA142849
succinate dehydrogenase complex, subunit	AW249904
delta sleep inducing peptide, immunoreac	AL561046
cathepsin L	AL570879
ESTs	AA704555
VGF nerve growth factor inducible	BE855486
SRY (sex determining region Y)-box 9 (ca	NM_000346
hypothetical protein HDCMC04P	AA460875
alkaline phosphatase, intestinal	NM_001631
hypothetical protein FLJ20647	AA732032
glutathione synthetase	AL573289
YY1 transcription factor	AV740554
heterogeneous nuclear ribonucleoprotein	AI277400
palmitoyl-protein thioesterase 1 (ceroid	NM_000310
interferon gamma receptor 2 (interferon	AL550285
jun B proto-oncogene	AU141279
osteoclast stimulating factor 1	BG032161
chromosome 3p21.1 gene sequence	BF975337
bHLH protein DEC2	AI978761
BM022 protein	AI766190
Junk, low PCR	EMPTY
Junk, low PCR	EMPTY
hypothetical protein FLJ11362	AW444842
natural killer cell group 7 sequence	S69115
ESTs, Weakly similar to I38428 T-complex	AA621014
hypothetical protein FLJ11021 similar to	AI375382
putative methyltransferase	BE274407
ESTs	N76867
calcium channel, voltage-dependent, beta	NM_000724
proteasome (prosome, macropain) 26S subu	BG035188
agouti (mouse) related protein	NM_001138
U5 snRNP-specific 40 kDa protein (hPrp8-	AL157420
solute carrier family 9 (sodium/hydrogen	NM_006359
Junk, low PCR	EMPTY
calcyclin binding protein	BG285728
lysophospholipase I	AI889004
bassoon (presynaptic cytomatrix protein)	F03960
SEC14 (S. cerevisiae)-like 1	AA811770
protein phosphatase 1, regulatory (inhib	XM_006813
nuclear receptor subfamily 4, group A, m	XM_006843
transcription factor 7-like 2 (T-cell sp	AA918322
PL6 protein	AI934503
zinc finger protein 32 (KOX 30)	W21271
DKFZP586B0519 protein	AL558585
fibroblast growth factor 7 (keratinocyte	AI075338
CGI-204 protein	AA133124
Homo sapiens Chromosome 16 BAC clone CIT	AC002301
nucleophosmin/nucleoplasmin 3	AI631542

ribosomal protein S14	BF663040
topoisomerase (DNA) I	R60160
nuclear body protein Sp140	U36500
RAR-related orphan receptor C	NM_005060
Human mRNA for 3-oxoacyl-CoA peroxisomal	EMPTY
steroidogenic acute regulatory protein r	NM_006804
retinoid X receptor, gamma	XM_001875
ESTs, Weakly similar to DP1_HUMAN POLYPO	AA528121
chromatin assembly factor 1, subunit B (	NM_005441
KIAA1046 protein	AA810084
hypothetical protein FLJ10925	AU154931
fucosidase, alpha-L- 1, tissue	NM_000147
kallikrein 7 (chymotryptic, stratum corn	NM_005046
KIAA0974 protein	BE669938
ESTs	AW005593
ESTs	AI026771
NPC1 (Niemann-Pick disease, type C1, gen	XM_004654
solute carrier family 1 (glutamate/neutr	AW950075
hypothetical protein FLJ11560	BF340474
G protein-coupled receptor kinase 5	NM_005308
Junk, low PCR, low PCR	EMPTY
acetyl-Coenzyme A transporter	D88152
H.sapiens mRNA for alpha-centractin	EMPTY
hypothetical protein	BE858411
Junk, low PCR	EMPTY
hypothetical protein FLJ12748	NM_024871
immunoglobulin heavy constant gamma 3 (G	AJ390260
collagen, type IV, alpha 1	XM_007094
Junk, low PCR	EMPTY
Sjogren syndrome antigen A1 (52kD, ribon	BE897247
chromosome 1 open reading frame 2	NM_006589
solute carrier family 20 (phosphate tran	AA977515
ESTs, Weakly similar to Y961_HUMAN HYPOT	AW300098
Rho guanine exchange factor (GEF) 12	AA234500
KIAA0649 gene product	AA861712
ESTs, Moderately similar to ALU7_HUMAN A	BE173815
diacylglycerol kinase, epsilon (64kD)	NM_003647
opiate receptor-like 1	AL574931
aminoacylase 1	AL578299
neuro-oncological ventral antigen 2	BE501446
Human capping protein alpha mRNA, partia	EMPTY
Junk, low PCR	EMPTY
B-cell translocation gene 1, anti-prolif	AI348005
formyltetrahydrofolate dehydrogenase	XM_002998
ATPase, H+ transporting, lysosomal (vacu	AL548523
hypothetical protein ET	AL545372
No ID Incyte EST	EMPTY
RAR-related orphan receptor A	NM_002943
ATP synthase, H+ transporting, mitochond	AU143446
ESTs	AA721277
Homo sapiens mRNA; cDNA DKFZp761M02121 (	AW673709

ESTs	H81066
Junk, low PCR	EMPTY
interleukin 1 receptor accessory protein	NM_002182
nebulin	NM_004543
KRAB-associated protein 1	BG386849
Protein P3	X12458
adenylate kinase 1	XM_005601
Human mRNA for 26S proteasome subunit p9	EMPTY
heterogeneous nuclear ribonucleoprotein	AU138031
cellular retinoic acid-binding protein 2	NM_001878
diacylglycerol kinase, gamma (90kD)	AW014722
Junk, low PCR	EMPTY
matrix metalloproteinase 12 (macrophage	U78045
makorin, ring finger protein, 1	AF117233
melanocortin 1 receptor (alpha melanocyt	XM_008010
phosphoribosylformylglycinamidine syntha	BG337624
KIAA1037 protein	AW131609
KIAA1107 protein	R55697
hypothetical protein FLJ10948	AA805411
ESTs	AA127761
synaptogyrin 2	AL545227
spindlin-like	BG033823
protein kinase, AMP-activated, gamma 1 n	AW411228
Spi-B transcription factor (Spi-1/PU.1 r	X66079
crystallin, alpha B	BF727296
Lactate Dehydrogenase A	EMPTY
regulator of G-protein signalling 5	AI674877
FEM-1 (C.elegans) homolog b	AF204883
Homo sapiens mRNA; cDNA DKFZp586D1122 (f	AL050166
katanin p80 (WD40-containing) subunit B	NM_005886
Junk, low PCR	EMPTY
ESTs, Moderately similar to ALU5_HUMAN A	AA256510
phospholipase C, delta 1	NM_006225
solute carrier family 23 (nucleobase tra	AL389886
progesterone membrane binding protein	BE858855
Ras homolog enriched in brain 2	AI889521
ESTs	AL120562
Junk, low PCR	EMPTY
thioredoxin-like	AI479766
KIAA0874 protein	AI692537
putative dimethyladenosine transferase	BG178374
neuroblastoma, suppression of tumorigeni	BF725092
neural cell adhesion molecule 2	NM_004540
Human MRL3 mRNA for ribosomal protein L3	EMPTY
Human clone 23719 mRNA sequence	U79256
baculoviral IAP repeat-containing 1	NM_004536
ESTs	AW183010
Junk, low PCR	EMPTY
insulin-like growth factor binding prote	BE336944
aldehyde dehydrogenase 7 family, member	AA644249
polymyositis/scleroderma autoantigen 2 (	AU133245

phosphorylase, glycogen; liver (Hers dis	AI539344
Homo sapiens cDNA: FLJ23332 fis, clone H	AK026985
suppressor of S. cerevisiae gcr2	XM_005726
ARF protein	XM_008376
ESTs	AA398450
poly(A)-binding protein, cytoplasmic 1-l	AK026760
Rho-associated, coiled-coil containing p	D87931
proteasome (prosome, macropain) 26S subu	AI199620
retinoblastoma-binding protein 7	BE378321
serine threonine protein kinase	AU121423
Arabadopsis7-31.25	EMPTY
homocysteine-inducible, endoplasmic reti	AA126606
suppressor of variegation 3-9 (Drosophil	NM_003173
Homo sapiens cDNA: FLJ22361 fis, clone H	AU147110
ectonucleotide pyrophosphatase/phosphodi	AC005587
DNA (cytosine-5-)-methyltransferase 1	BF439104
KIAA1073 protein	R46367
hypothetical protein FLJ21079	BF795598
KIAA0537 gene product	NM_014840
heat shock 27kD protein 2	NM_001541
ESTs	BE408838
Junk, low PCR	EMPTY
ESTs	AI376573
DKFZP434D193 protein	AW176766
FSH primary response (LRPR1, rat) homolo	XM_010071
sarcoglycan, delta (35kD dystrophin-asso	AI085599
apical protein, Xenopus laevis-like	NM_001649
troponin C2, fast	XM_009487
Arabadopsis9-31.25	EMPTY
mannose phosphate isomerase	NM_002435
myomesin (M-protein) 2 (165kD)	XM_005198
LBP protein 32	BE045743
Homo sapiens cDNA: FLJ22281 fis, clone H	AI079954
translocated promoter region (to activat	AW275000
gap junction protein, beta 5 (connexin 3	BF509618
hypothetical protein PRO0971	AI246762
putative receptor protein	BF314801
RNA binding motif, single stranded inter	NM_002898
hypothetical protein FLJ23053	AA011187
transducin (beta)-like 3	BG491144
Junk, low PCR	EMPTY
tumor necrosis factor (ligand) superfami	NM_003809
glutamate receptor, ionotropic, N-methyl	NM_000833
surfeit 1	AL549808
c-src tyrosine kinase	BG391140
dual-specificity tyrosine-(Y)-phosphoryl	D86550
Empty	EMPTY
coagulation factor XII (Hageman factor)	NM_000505
sialyltransferase 4A (beta-galactosidase	AF186191
Junk, low PCR	EMPTY
neurexin 1	AB035356

uromodulin (uromucoid, Tamm-Horsfall gly	BG430266
Homo sapiens clone 25048 mRNA sequence	AL134750
DKFZP586B0923 protein	AA044848
neuropilin 2	AF280546
purine-rich element binding protein A	NM_005859
KIAA0493 protein	AW516811
Junk, low PCR	EMPTY
No ID Incyte EST	EMPTY
succinate dehydrogenase complex, subunit	BE747410
vascular endothelial growth factor	AI827301
vanin 1	XM_004354
Junk, low PCR	EMPTY
mitochondrial carrier family protein	AC003083
Empty	EMPTY
Junk, low PCR	EMPTY
ESTs	BG402091
hypothetical protein FLJ22195	BE301214
chitobiase, di-N-acetyl-	AA688097
CASP8 and FADD-like apoptosis regulator	AU117908
KIAA0210 gene product	BF062197
sema domain, immunoglobulin domain (Ig),	AI081830
hypothetical protein FLJ20274	AC004381
RE1-silencing transcription factor	NM_005612
ubiquitin carrier protein E2-C	AA403150
transketolase (Wernicke-Korsakoff syndro	BE531024
succinate-CoA ligase, ADP-forming, beta	AW470095
Junk, low PCR	EMPTY
oxysterol binding protein	AI992266
EST	AI824705
UDP glycosyltransferase 2 family, polype	NM_001077
phosphoribosylglycinamide formyltransfer	BE018161
Empty	EMPTY
ataxia telangiectasia mutated (includes	U67093
NO ID Incyte EST	EMPTY
hypothetical protein FLJ10955	AI670067
Junk, low PCR	EMPTY
solute carrier family 25 (mitochondrial	BF668036
KIAA0923 protein	AI017856
KIAA1140 protein	AB032966
ClpP (caseinolytic protease, ATP-depende	AW025478
Junk, low PCR	EMPTY
ESTs	AA258392
homeo box A4	XM_004917
Zic family member 2 (odd-paired Drosophi	AF188733
KIAA0729 protein	BE173435
thousand and one amino acid protein kina	NM_004783
BCL2-associated athanogene 5	N25712
plexin B2	AK025415
Homo sapiens clone 23940 mRNA sequence	BC003539
Empty	EMPTY
serine (or cysteine) proteinase inhibito	NM_002640

ESTs	AA394082
ESTs	AI207103
protein phosphatase 1G (formerly 2C), ma	AI992326
signal transducer and activator of trans	AI582321
ESTs	AI241826
phorbolin-like protein MDS019	AI280224
dyskeratosis congenita 1, dyskerin	BE796463
desmocollin 2	NM_024422
DEAD/H (Asp-Glu-Ala-Asp/His) box polypep	AI056019
Junk, low PCR	EMPTY
Junk, low PCR	EMPTY
nerve growth factor receptor (TNFR super	XM_008437
peroxisome receptor 1	X84899
platelet activating receptor homolog	AV653286
allograft inflammatory factor 1	Y14768
Meis1 (mouse) homolog	NM_002398
synuclein, alpha (non A4 component of am	AW156890
Homo sapiens clone 24747 mRNA sequence	AF055009
Homo sapiens cDNA FLJ12563 fis, clone NT	BF437675
CDC-like kinase 2	BG286233
v-myb avian myeloblastosis viral oncogen	BG387620
ESTs, Moderately similar to 810024C cyto	AI949200
CD151 antigen	BE787930
RAD21 (S. pombe) homolog	BG504544
Junk, low PCR	EMPTY
Homo sapiens HSPC268 mRNA, partial cds	BF110215
KIAA1093 protein	N64803
adenomatosis polyposis coli	AA813571
diacylglycerol kinase, beta (90kD)	AB018261
Junk, low PCR	EMPTY
potassium inwardly-rectifying channel, s	AA056665
Junk, low PCR	EMPTY
transcobalamin I (vitamin B12 binding pr	XM_006282
asparagine synthetase	NM_001673
aryl hydrocarbon receptor-interacting pr	AL551147
serine (or cysteine) proteinase inhibito	AL541945
KIAA0185 protein	AI635341
putative c-Myc-responsive	AW973317
sema domain, immunoglobulin domain (Ig),	NM_012431
KIAA0675 gene product	N46327
Junk, low PCR	EMPTY
ubiquitin-conjugating enzyme E2L 6	AW976741
opioid-binding protein/cell adhesion mol	U79251
hypothetical protein DKFZp434K0920	AA587614
Junk, low PCR	EMPTY
Homo sapiens mRNA; cDNA DKFZp762N226 (fr	AL359937
renin	AI074210
EphA1	NM_005232
transcription elongation factor A (SII)-	AW006970
KIAA0415 gene product	AB007875
protein phosphatase 2, regulatory subuni	AV708790

interferon, alpha-inducible protein (clo	NM_022872
deoxycytidine kinase	AA912978
golgi resident protein GCP60	AL558223
Junk, low PCR	EMPTY
activin A receptor type II-like 1	XM_006839
potassium voltage-gated channel, shaker-	U16953
ESTs, Weakly similar to ALUC_HUMAN !!!!	BE464819
epiregulin	BG235918
gelsolin (amyloidosis, Finnish type)	NM_000177
forkhead box I1	NM_012188
ESTs	AI801963
DKFZP566D133 protein	AA115176
hypothetical protein DKFZp434F2322	BF196093
chromodomain helicase DNA binding protei	AI679237
Junk, low PCR	EMPTY
zinc finger protein 6 (CMPX1)	XM_013097
H.sapiens DNA for cyp related pseudogene	X90579
coagulation factor III (thromboplastin,	AI924814
UDP-Gal:betaGlcNAc beta 1,4- galactosylt	BF229298
Human hbc647 mRNA sequence	U68494
DNA segment on chromosome 12 (unique) 24	BG392353
chromosome 4 open reading frame 1	AI499467
p300/CBP-associated factor	NM_003884
v-rel avian reticuloendotheliosis viral	NM_002908
Homo sapiens mRNA; cDNA DKFZp564O2364 (f	AL117623
HUS1 (S. pombe) checkpoint homolog	Y16893
Junk, low PCR	EMPTY
Junk, low PCR	EMPTY
ESTs, Weakly similar to ALU1_HUMAN ALU S	BF444979
fibrinogen-like 1	N92944
ESTs	AA863490
Homo sapiens HSFE-1 mRNA, partial cds	AF072164
coproporphyrinogen oxidase (coproporphyr	BG498060
a disintegrin and metalloproteinase doma	BF590530
CD5 antigen (p56-62)	AI797836
Homo sapiens clone 24707 mRNA sequence	AI376818
Junk, low PCR	EMPTY
procollagen-lysine, 2-oxoglutarate 5-dio	AV711206
Junk, low PCR	EMPTY
ESTs	AA455180
N-myristoyltransferase 1	M86707
sema domain, seven thrombospondin repeat	NM_003966
sperm associated antigen 9	AU151713
signaling lymphocytic activation molecul	XM_010593
RAB5B, member RAS oncogene family	NM_002868
Junk, low PCR	EMPTY
ESTs, Weakly similar to ISHUSS protein d	AA468796
Homo sapiens mRNA; cDNA DKFZp566B213 (fr	AI244975
olfactory receptor, family 7, subfamily	BG032285
KIAA0623 gene product	BE763755
Junk, low PCR	EMPTY

lectomedin-2	XM_008974
zinc finger protein 204	AW338359
DEAD/H (Asp-Glu-Ala-Asp/His) box polypep	AU125885
cholecystokinin B receptor	XM_006034
dual-specificity tyrosine-(Y)-phosphoryl	NM_006482
p53 inducible protein	AB032994
ESTs	BG500474
ATP-binding cassette, sub-family A (ABC1	AU140108
Junk, low PCR	EMPTY
zinc finger protein 202	BE962995
a disintegrin-like and metalloprotease (	AB002364
Junk, low PCR	EMPTY
Junk, low PCR	EMPTY
ESTs	AA281719
ESTs	BG484250
Homo sapiens, clone IMAGE:3357127, mRNA,	AW305005
immunoglobulin lambda locus	M87790
Junk, low PCR	EMPTY
Homo sapiens mRNA; cDNA DKFZp566E0124 (f	AI694583
KIAA0842 protein	AL137297
UDP-glucose pyrophosphorylase 2	AV714998
carboxypeptidase E	NM_001873
Human cytochrome c-1 gene, complete cds	EMPTY
HIV-1 Tat interactive protein, 60 kDa	AL519981
basic transcription element binding prot	XM_005584
dynamin 1-like	NM_005690
meiotic recombination (S. cerevisiae) 11	NM_005591
KIAA0459 protein	AA868112
myosin, light polypeptide 3, alkali; ven	XM_002896
ATP synthase, H+ transporting, mitochond	BE746047
Junk, low PCR	EMPTY
Incyte EST	EMPTY
HSPC156 protein	AV726478
ESTs, Weakly similar to ALU1_HUMAN ALU S	AA004426
CCR4-NOT transcription complex, subunit	N69096
protein kinase C binding protein 2	AF221520
Fc fragment of IgE, low affinity II, rec	AI936513
amphiregulin (schwannoma-derived growth	AL546917
mitochondrial translational release fact	AI884353
calcium channel, voltage-dependent, gamm	AF134640
H.sapiens mRNA for splicing factor SF3a1	EMPTY
zinc finger protein 76 (expressed in tes	BE617478
DNA-binding transcriptional activator	NM_006316
small nuclear ribonucleoprotein polypept	AI693964
small inducible cytokine A5 (RANTES)	AA569974
ESTs, Weakly similar to B7 [M.musculus]	AW514067
Junk, low PCR	EMPTY
dihydrolipoamide branched chain transacy	NM_001918
G antigen, family B, 1 (prostate associa	AA972716
cyclin D binding Myb-like transcription	AA912946
sine oculis homeobox (Drosophila) homolo	BG433378

TASP for testis-specific adriamycin sens	BE219611
Homo sapiens cDNA FLJ13555 fis, clone PL	AI743376
cytoskeleton-associated protein 1	AI378136
No ID Incyte EST	EMPTY
ATP-binding cassette, sub-family G (WHIT	AU118354
very long-chain acyl-CoA synthetase; lip	AB014531
tachykinin receptor 1	NM_015727
Human mRNA for platelet-type phosphofruc	EMPTY
GCN5 (general control of amino-acid synt	BF725471
myeloid/lymphoid or mixed-lineage leukem	NM_005938
GATA-binding protein 3	BC003070
myosin, light polypeptide 1, alkali; ske	BF981829
iroquois homeobox protein 5	AI499593
synaptotagmin V	XM_012840
protein tyrosine phosphatase, receptor t	AL121905
DNA-dependent protein kinase catalytic s	BE042882
hypothetical protein FLJ10262	AA609749
ESTs, Weakly similar to T23985 hypotheti	BG028985
Junk, low PCR	EMPTY
Homo sapiens cDNA FLJ10684 fis, clone NT	AI768720
GRO3 oncogene	XM_003508
LIM domain only 1 (rhombotin 1)	AI364139
vesicle-associated soluble NSF attachmen	BG330450
ESTs	BE504140
ATP-binding cassette, sub-family F (GCN2	BF308051
Human eukaryotic initiation factor 2B-ep	EMPTY
Homo sapiens, Similar to RuvB (E coli ho	BC004503
ring finger protein 5	AI570435
SWI/SNF related, matrix associated, acti	XM_006756
myeloid cell leukemia sequence 1 (BCL2-r	BE408610
Homo sapiens cDNA: FLJ22844 fis, clone K	AI051635
keratin, cuticle, ultrahigh sulphur 1	X63755
caspase 4, apoptosis-related cysteine pr	BF666770
ESTs	AI619701
Homo sapiens mRNA; cDNA DKFZp434P0316 (f	BE467478
ESTs	AA007282
Homo sapiens cDNA: FLJ22704 fis, clone H	AI285677
myosin VIIA (Usher syndrome 1B (autosoma	AA994181
protein kinase, cGMP-dependent, type I	XM_011869
protein Z, vitamin K-dependent plasma gl	AB033749
microtubule-associated protein, RP/EB fa	BE348538
KIAA0830 protein	AI186024
guanylate cyclase 1, soluble, beta 3	NM_000857
Human dihydrolipoamide dehydrogenase mRN	EMPTY
KIAA0170 gene product	NM_014641
similar to S. pombe dim1+	BE275227
bone morphogenetic protein receptor, typ	NM_004329
CD1D antigen, d polypeptide	NM_001766
Junk, low PCR	EMPTY
selectin P ligand	XM_006867
cadherin 1, type 1, E-cadherin (epitheli	AI690659

ESTs	F09687
hypothetical protein FLJ10466	AI081858
ESTs	AA846182
zinc finger protein 281	AA948396
Homo sapiens mRNA; cDNA DKFZp547E052 (fr	AI022306
E4F transcription factor 1	BE245249
CDC14 (cell division cycle 14, S. cerevi	AF064104
receptor (calcitonin) activity modifying	AI244379
fibulin 5	AL570066
endothelin receptor type A	XM_003485
Human ubiquitin-activating enzyme E1 (UB	EMPTY
KIAA0564 protein	AK025432
solute carrier family 17 (sodium phospha	NM_006632
membrane component, chromosome 11, surfa	BG252978
superoxide dismutase 2, mitochondrial	BG035651
KIAA0750 gene product	XM_006081
immunoglobulin superfamily, member 1	NM_001555
nuclear factor of kappa light polypeptid	AL575644
ESTs	BF739952
KIAA0955 protein	AA287702
ESTs	AI056517
ESTs	AI080726
hypothetical protein FLJ12479	AW241775
glypican 3	AL543321
fascin (Strongylocentrotus purpuratus) h	NM_012418
STAT induced STAT inhibitor-2	XM_006822
KIAA0427 gene product	NM_014772
integrin, alpha 7	AJ228836
Arabadopsis1-31.25	EMPTY
cellular retinoic acid-binding protein 1	XM_007579
Junk, low PCR	EMPTY
ESTs	AA678193
zinc finger protein 84 (HPF2)	NM_003428
synaptogyrin 1	NM_004711
Human clone 23826 mRNA sequence	AW953678
zinc finger protein 161	AA195154
ESTs	AI051798
KIAA0134 gene product	NM_014681
ESTs	AA743092
hypothetical protein FLJ10597	AA764946
ESTs	AW298595
CD163 antigen	AI129762
KIAA0140 gene product	NM_014661
Homo sapiens protein kinase C-alpha mRNA	AF035594
KIAA0471 gene product	NM_014857
protein tyrosine phosphatase, receptor t	NM_002844
Arabadopsis3-31.25	EMPTY
coated vesicle membrane protein	AU136987
Homo sapiens clone 23686 and 23885 mRNA	AF007146
Homo sapiens cDNA FLJ13510 fis, clone PL	AI817281
thyroid hormone receptor interactor 12	NM_004238

cerebellar degeneration-related protein	AU120904
zinc finger protein 262	NM_005095
protein tyrosine phosphatase, non-recept	BC001746
polymerase (RNA) III (DNA directed) (32k	AK025490
sodium channel, nonvoltage-gated 1, gamm	L36592
chromosome 21 open reading frame 51	AI686692
Homo sapiens cDNA FLJ11311 fis, clone PL	AL050081
Homo sapiens mRNA; cDNA DKFZp564B2062 (f	AA514530
Homo sapiens clone 24626 mRNA sequence	AF052141
f-box and WD-40 domain protein 1B	AA436684
huntingtin interacting protein-1-related	AB014555
myosin X	AU151619
pre-T/NK cell associated protein	XM_011662
Arabadopsis5-31.25	EMPTY
glycoprotein M6B	AI540344
hypothetical protein from BCRA2 region	AU130970
ESTs	AA699839
arachidonate 5-lipoxygenase-activating p	AW950669
Junk, low PCR	EMPTY
KIAA0146 protein	AU130263
pancreatic polypeptide	NM_002722
ESTs	BF064257
Junk, low PCR	EMPTY
coronin, actin-binding protein, 2B	AI986153
chromosome 11 hypothetical protein ORF4	BF062346
ESTs	AA135870
hepatocyte nuclear factor 3, gamma	XM_012811
polymerase (DNA directed), gamma 2, acce	AI701141
KIAA0635 gene product	AW872736
aldehyde dehydrogenase 8 family, member	AI051566
Homo sapiens clone 24711 mRNA sequence	AF055029
Empty	EMPTY
defensin, alpha 4, corticostatin	NM_001925
Homo sapiens clone 23568, 23621, 23795,	BG029113
ESTs	AA658167
complement component 3a receptor 1	NM_004054
polymerase (RNA) II (DNA directed) polyp	BE796653
protein phosphatase 2 (formerly 2A), cat	NM_002715
protein phosphatase 2, regulatory subuni	NM_002719
solute carrier family 25 (mitochondrial	AV743379
kinesin-like 6 (mitotic centromere-assoc	NM_006845
hypothetical protein FLJ20623	AA112479
ESTs	AA446606
Junk, low PCR	EMPTY
H.sapiens novel gene from PAC 117P20, ch	AA594756
Homo sapiens, clone IMAGE:3506210, mRNA,	AF016903
Homo sapiens clone 23608 mRNA sequence	AF038193
Homo sapiens clone 23632 mRNA sequence	AW601612
KIAA0009 gene product	AW161286
Empty	EMPTY
major histocompatibility complex, class	NM_002118

Junk, low PCR	EMPTY
ESTs	AA776789
solute carrier family 29 (nucleoside tra	AI500247
desmoglein 2	XM_008801
pyruvate kinase, muscle	AW007619
calbindin 1, (28kD)	NM_004929
regulatory factor X, 3 (influences HLA c	NM_002919
Junk, low PCR	EMPTY
chromosome 1 open reading frame 12	AW408810
ESTs	AI263389
Homo sapiens cDNA FLJ14181 fis, clone NT	AI051256
huntingtin-associated protein 1 (neuroan	AK022007
complement component 4-binding protein,	AV652811
regulator of G-protein signalling 14	BE394825
activator of S phase kinase	AV728361
heterogeneous nuclear ribonucleoprotein	BF879916
Empty	EMPTY
mannose receptor, C type 1	NM_002438
kruppel-related zinc finger protein hcKr	XM_001785
protein kinase, cAMP-dependent, regulato	AK023535
phorbolin (similar to apolipoprotein B m	XM_009996
autoantigen	AW967379
collagen, type VIII, alpha 1	XM_002965
inositol polyphosphate-5-phosphatase, 14	U53470
lipopolysaccharide-binding protein	XM_012965
paired box gene 3 (Waardenburg syndrome	XM_002580
zinc finger protein	AW975225
Junk, low PCR	EMPTY
ESTs	BE676495
postmeiotic segregation increased (S. ce	AA278390
Homo sapiens clone 23556 mRNA sequence	AF035312
odz (odd Oz/ten-m, Drosophila) homolog 1	AW779760
katanin p60 (ATPase-containing) subunit	BF436125
guanylate cyclase 1, soluble, alpha 3	NM_000856
v-erb-b2 avian erythroblastic leukemia v	W73967
hypothetical protein FLJ00052	AK024460
Putative prostate cancer tumor suppresso	AI962856
fatty acid binding protein 6, ileal (gas	NM_001445
cyclin G2	AI271688
ESTs	BF694680
tumor necrosis factor receptor superfami	D86042
phosphatidylserine receptor	AB011157
interleukin 13	NM_002188
DEAD/H (Asp-Glu-Ala-Asp/His) box polypep	AL529007
heterogeneous nuclear ribonucleoprotein	AL518953
homeo box B7	XM_008559
karyopherin (importin) beta 1	BE254681
proteasome (prosome, macropain) 26S subu	AI568351
sodium channel, nonvoltage-gated 1, delt	NM_002978
ubiquitin activating enzyme E1-like prot	AL520089
phosphoinositide-3-kinase, catalytic, be	NM_006219

aldehyde dehydrogenase 3 family, member	NM_000694
hypothetical protein MPMGp800B12492Q3	AA506687
hypothetical protein FLJ11350	AI469468
tropomyosin 4	BC002827
KIAA0601 protein	AB011173
RAB33A, member RAS oncogene family	AA809304
ESTs	AA813998
exonuclease 1	AC004783
ubiquitin-conjugating enzyme E2I (homolo	BF038750
galactosamine (N-acetyl)-6-sulfate sulfa	NM_000512
pleckstrin homology, Sec7 and coiled/coi	NM_004762
prostaglandin-endoperoxide synthase 1 (p	U63846
EphB6	NM_004445
ribosomal protein L12	BG282851
myosin, heavy polypeptide 9, non-muscle	NM_002473
GSK-3 binding protein FRAT2	AB045118
uroplakin 3	XM_010042
propionyl Coenzyme A carboxylase, beta p	AJ006487
zinc finger protein 147 (estrogen-respon	XM_012593
ESTs, Highly similar to ALU8_HUMAN ALU S	AA844129
serine/threonine kinase with Dbl- and pl	AI479318
CD3E antigen, epsilon polypeptide (TiT3	AW950965
Junk, low PCR	EMPTY
S164 protein	AI636032
hypothetical protein DKFZp586E1923	AL096817
nerve growth factor, beta polypeptide	NM_002506
junction plakoglobin	AA826828
defensin, alpha 5, Paneth cell-specific	NM_021010
Junk, low PCR	EMPTY
Junk, low PCR	EMPTY
ribonuclease P (30kD)	NM_006413
wingless-type MMTV integration site fami	NM_004625
activated leucocyte cell adhesion molecu	AI050952
hexokinase 2	Z46354
promyelocytic leukemia	W38850
hypothetical protein FLJ10803	AI863186
TRAF and TNF receptor-associated protein	NM_016614
ESTs	AA481433
Homo sapiens cDNA FLJ10151 fis, clone HE	AW295967
B cell RAG associated protein	NM_014863
follicular lymphoma variant translocatio	NM_002035
syntaxin 4A (placental)	BC002436
Junk, low PCR	EMPTY
chromodomain helicase DNA binding protei	NM_001271
kinesin heavy chain member 2	BF205254
amyloid beta (A4) precursor protein-bind	NM_001164
glucosidase, beta; acid (includes glucos	NM_000157
Fc fragment of IgE, high affinity I, rec	BG542554
G protein-coupled receptor 37 (endotheli	AC004925
U2 small nuclear ribonucleoprotein auxil	NM_005089
solute carrier family 16 (monocarboxylic	AI565092

delta-like homolog (Drosophila)	BF969929
poly(rC)-binding protein 2	AA375284
complement component 8, beta polypeptide	NM_000066
MAD (mothers against decapentaplegic, Dr	NM_005359
ESTs	AW271626
Human Chromosome 16 BAC clone CIT987SK-A	AA888034
Human transposon-like element mRNA	BG529632
activated p21cdc42Hs kinase	XM_010938
isocitrate dehydrogenase 3 (NAD+) gamma	AL545619
Junk, low PCR	EMPTY
natriuretic peptide receptor B/guanylate	XM_005396
KIAA0411 gene product	AB007871
cubilin (intrinsic factor-cobalamin rece	XM_011904
RAP1A, member of RAS oncogene family	AL049557
KIAA0218 gene product	NM_014760
Junk, low PCR	EMPTY
Homo sapiens clone IMAGE 21721	AI637917
cyclin D1 (PRAD1: parathyroid adenomatos	X59798
protein kinase C, epsilon	NM_005400
hypothetical protein	BG479153
Junk, low PCR	EMPTY
interleukin 2 receptor, beta	NM_000878
EST	R53900
hypothetical protein	N58073
nucleotide binding protein	AU154673
arginase, type II	NM_001172
Junk, low PCR	EMPTY
ESTs, Weakly similar to ALU1_HUMAN ALU S	AA423971
G protein-coupled receptor 64	NM_005756
tyrosine hydroxylase	NM_000360
regulator of G-protein signalling 13	AF030107
KIAA0554 protein	AB011126
RAD1 (S. pombe) homolog	AI870850
ribosomal protein, large, P0	BF669160
tissue inhibitor of metalloproteinase 1	AL555891
IMP (inosine monophosphate) dehydrogenas	AL525547
zinc finger protein 220	NM_006766
hypothetical protein FLJ10305	AL555680
synaptosomal-associated protein, 25kD	AL536948
KIAA0461 protein	AI470495
Human protein kinase mRNA, complete cds	EMPTY
Homo sapiens mRNA; cDNA DKFZp434B0328 (f	BE868502
ESTs	BF340290
KIAA0365 gene product	AB002363
Junk, low PCR	EMPTY
ESTs	AI828837
macrophage stimulating 1 (hepatocyte gro	U28054
surfeit 1	AI074241
ESTs	AI623308
Homo sapiens similar to J KAPPA-RECOMBIN	XM_011187
ESTs	AI239923

superoxide dismutase 1, soluble (amyotro	AI421041
hypothetical protein FLJ22688	AA252187
formin-like	BG235961
H3 histone, family 3A	BE888633
eukaryotic translation initiation factor	AL520387
modulator recognition factor I	M62324
inositol 1,4,5-triphosphate receptor, ty	NM_002224
H.sapiens mRNA for a cell surface protei	EMPTY
polymerase (DNA-directed), mu	AA694217
ESTs	N74451
ubiquitin B	BG546472
EST	AA868598
Homo sapiens pinch-2 protein mRNA, compl	AA570650
ectonucleoside triphosphate diphosphohyd	XM_009435
KIAA0141 gene product	NM_014773
ESTs	AA626699
galactose-4-epimerase, UDP-	AW950343
Homo sapiens cDNA FLJ11723 fis, clone HE	AI758406
survival of motor neuron protein interac	AB037701
hypothetical protein FLJ22390	AA782824
sigma receptor (SR31747 binding protein	AL521605
ribosomal protein S7	AV725843
protein phosphatase 1, regulatory (inhib	XM_005884
bone marrow stromal cell antigen 1	AI418773
BCL2-antagonist of cell death	AW007022
Human superoxide dismutase (SOD-1) mRNA,	EMPTY
ESTs	AA058640
LIM domain-containing preferred transloc	AA470748
TATA box binding protein (TBP)-associate	AI681931
ESTs	R15922
ESTs	AA057503
ubiquitin-like 1 (sentrin)	BG529395
similar to vaccinia virus HindIII K4L OR	AL565758
Junk, low PCR	EMPTY
tetratricopeptide repeat domain 3	BF800512
inhibin, alpha	BE552259
KIAA0054 gene product; Helicase	NM_014877
solute carrier family 26, member 6	NM_022911
cyclic AMP phosphoprotein, 19 kD	AF084555
calcium and integrin binding protein (DN	BF685744
Homo sapiens clone 24400 mRNA sequence	AF052145
stromal cell-derived factor 1	AL538307
hypothetical protein MGC3067	AK023846
Human mRNA for mitochondrial 3-ketoacyl-	EMPTY
hypothetical protein FLJ12383	AU148105
Junk, low PCR	EMPTY
basic helix-loop-helix domain containing	NM_003670
acid cluster protein 33	N25699
ESTs	H21160
phospholipase C, gamma 1 (formerly subty	AL022394
putative GTP-binding protein similar to	XM_009956

peroxisome proliferative activated recep	N90328
C-terminal binding protein 2	NM_001329
ESTs	AI418830
sialophorin (gpL115, leukosialin, CD43)	NM_003123
frizzled (Drosophila) homolog 7	AI351279
Homo sapiens clone 24421 mRNA sequence	AF070641
DiGeorge syndrome gene D	L77561
ubiquitin specific protease 19	AB020698
karyopherin alpha 2 (RAG cohort 1, impor	BE889289
neurochondrin	AC004865
Unknown - Human Control Plate Well H8	EMPTY
ESTs	W56900
testis-specific kinase 2	AI564142
cullin 1	AL560070
Junk, low PCR	EMPTY
geranylgeranyl diphosphate synthase 1	AI298328
adenylate cyclase 2 (brain)	AI160340
protein kinase, cAMP-dependent, regulato	NM_002736
gap junction protein, beta 3, 31kD (conn	AW276400
SEC14 (S. cerevisiae)-like 1	XM_008142
lymphoid blast crisis oncogene	AI492045
signal-induced proliferation-associated	AF029789
solute carrier family 25 (mitochondrial	AI382550
minichromosome maintenance deficient (S.	AI246721
N-acetylated alpha-linked acidic dipepti	XM_006526
Junk, low PCR	EMPTY
KIAA0187 gene product	NM_014753
Homo sapiens clone 23676 mRNA sequence	AF035278
Unknown - Human Control Plate Well H11	EMPTY
ESTs	AW451074
Homo sapiens mRNA; cDNA DKFZp434E109 (fr	AI653967
klotho	NM_004795
SWI/SNF related, matrix associated, acti	BF977892
ESTs	AA780295
chloride channel 5 (nephrolithiasis 2, X	X81836
alcohol dehydrogenase 4 (class II), pi p	XM_011116
ESTs	AA034416
Junk, low PCR	EMPTY
ESTs, Weakly similar to serin protease w	AL545499
sperm associated antigen 1	XM_011690
novel putative protein similar to YIL091	XM_001712
electron-transferring-flavoprotein dehyd	NM_004453
Homo Sapiens mRNA, partial cDNA sequence	AJ001873
potassium voltage-gated channel, Shal-re	AF166003
phosphomannomutase 2	AW173669
Homo sapiens mRNA; cDNA DKFZp434P086 (fr	AA813471
Empty	EMPTY
fibromodulin	AL551623
Junk, low PCR	EMPTY
protein tyrosine phosphatase, non-recept	AL034562
Junk, low PCR	EMPTY

erythroid differentiation and denucleati	AW827085
highly expressed in cancer, rich in leuc	AA878068
Junk, low PCR	EMPTY
ESTs	AA256765
ESTs	N45099
Incyte EST	EMPTY
hypothetical protein AF053356_CDS3	AI424097
ESTs	BE901320
nuclear antigen Sp100	BF516261
Junk, low PCR	EMPTY
polymerase (DNA-directed), alpha (70kD)	NM_002689
protein kinase, cAMP-dependent, regulato	BG480549
glycogen synthase 1 (muscle)	AL556228
Empty	EMPTY
ESTs	N33584
homolog of mouse quaking QKI (KH domain	AW612422
laminin, beta 3 (nicein (125kD), kalinin	AL023754
ATP-binding cassette, sub-family A (ABC1	AA737119
Junk, low PCR	EMPTY
nitric oxide synthase 3 (endothelial cel	X76303
neuronal PAS domain protein 2	XM_002406
pro-melanin-concentrating hormone	BF110313
ESTs	AW241275
MAGUK protein p55T; Protein Associated w	AI147946
KIAA0010 gene product	NM_014671
ESTs	AA935265
deleted in oral cancer (mouse, homolog)	BG291410
Junk, low PCR	EMPTY
NADH dehydrogenase (ubiquinone) 1 beta s	BF244629
retinoblastoma-binding protein 7	X72841
pyruvate dehydrogenase (lipoamide) alpha	AU130883
Empty	EMPTY
ESTs, Weakly similar to ALU8_HUMAN ALU S	AA338736
ESTs, Weakly similar to Z184_HUMAN ZINC	T90470
Tax interaction protein 1	NM_014604
tyrosine 3-monooxygenase/tryptophan 5-mo	BF969911
Homo sapiens clone 23649 and 23755 unkno	AK024992
small nuclear RNA activating complex, po	AI453282
Junk, low PCR	EMPTY
Junk, low PCR	EMPTY
Homo sapiens cDNA FLJ11174 fis, clone PL	W51999
nasopharyngeal carcinoma susceptibility	AI207579
proteolipid protein (Pelizaeus-Merzbache	AL533222
phosphoprotein associated with GEMs	AW450854
methyl-CpG binding domain protein 1	BE747161
ribosomal protein L31	AV764048
zinc finger protein 261	AL532349
X-ray repair complementing defective rep	AA909333
Fc fragment of IgG, low affinity IIa, re	R25297
Empty	EMPTY
ESTs	N94196

Homo sapiens cDNA: FLJ20869 fis, clone A	AA664392
FOXJ2 forkhead factor	AF038177
hypothetical protein FLJ11171	AI283077
RAR (RAS like GTPASE)	BE621282
nucleolar protein (KKE/D repeat)	NM_006392
angiopoietin 1	BG496539
ESTs, Weakly similar to ALUA_HUMAN !!!!	AI796410
Homo sapiens cDNA FLJ12743 fis, clone NT	AK022805
ESTs	BF514823
imogen 38	NM_005830
similar to mouse Xrn1 / Dhm2 protein	AI741259
sulfotransferase family, cytosolic, 2B,	XM_009111
collagen, type XIX, alpha 1	XM_004150
prolactin-induced protein	BF965123
tumor-associated calcium signal transduc	BG259957
RNA binding motif protein 14	R92878
Empty	EMPTY
Homo sapiens mRNA; cDNA DKFZp566M0947 (f	AA450122
ESTs	AA905765
SMT3 (suppressor of mif two 3, yeast) ho	BF966739
CCR4-NOT transcription complex, subunit	AI769416
basement membrane-induced gene	XM_001646
protein kinase C, zeta	NM_002744
Junk, low PCR	EMPTY
No ID Incyte EST	EMPTY
KIAA1340 protein	AA627207
endoplasmic reticulum resident protein 5	N39195
cell division cycle 2, G1 to S and G2 to	BG033634
LCAT-like lysophospholipase	AU154510
procollagen (type III) N-endopeptidase	BE514420
GIOT-2 for gonadotropin inducible transc	NM_016264
aquaporin 9	NM_020980
neurofilament, heavy polypeptide (200kD)	NM_021076
D-aspartate oxidase	NM_003649
Empty	EMPTY
Homo sapiens cDNA: FLJ21606 fis, clone C	AI283270
ESTs	AA479347
GTP-binding protein overexpressed in ske	XM_005162
indolethylamine N-methyltransferase	AF128847
ubiquinol-cytochrome c reductase core pr	AL535015
Junk, low PCR	EMPTY
adenylate cyclase activating polypeptide	T10971
hypothetical protein FLJ20287	AI537153
a disintegrin and metalloproteinase doma	U86755
hypothetical protein PRO2706	AI473772
collagen, type XIV, alpha 1 (undulin)	M64108
hypothetical protein FLJ22548 similar to	AA846576
neuromedin B	BF109836
SRY (sex determining region Y)-box 3	AL121875
ribosomal protein S6 kinase, 90kD, polyp	AF080000
runt-related transcription factor 1 (acu	AW963298

ATPase, Class II, type 9A	AA429479
carboxyl ester lipase (bile salt-stimula	BG506707
ESTs, Weakly similar to ALU1_HUMAN ALU S	AI253616
KIAA0575 gene product	NM_014668
Junk, low PCR	EMPTY
Homo sapiens cDNA FLJ10325 fis, clone NT	AW270860
KIAA0265 protein	D87454
paired mesoderm homeobox 2b	NM_003924
eukaryotic translation initiation factor	NM_001414
Homo sapiens carbamoyl-phosphate synthet	XM_010819
interferon-induced protein 35	U72882
Junk, low PCR	EMPTY
hypothetical protein FLJ20094	AA708627
KIAA1564 protein	H92360
No ID Incyte EST	EMPTY
NAG-5 protein	AF149297
hypothetical protein A-211C6.1	BG108225
cathepsin O	N20599
insulin-like growth factor 2 receptor	BG325212
polymerase (RNA) III (DNA directed) (39k	XM_009639
ESTs	AI492971
hypothetical protein FLJ13868	NM_022744
Junk, low PCR	EMPTY
KIAA1085 protein	AL046316
ubiquitin fusion degradation 1-like	BE794596
msh (Drosophila) homeo box homolog 1 (fo	AI912103
peptidylglycine alpha-amidating monooxyg	S75037
Junk, low PCR	EMPTY
small inducible cytokine subfamily E, me	AV707896
19A24 protein	AI948861
Junk, low PCR	EMPTY
KIAA0706 gene product	AB014606
adenomatous polyposis coli like	AA490746
hypothetical protein PRO1847	BF352534
KIAA0453 protein	BG340388
peroxisomal biogenesis factor 3	AI332675
diacylglycerol kinase, zeta (104kD)	AL529034
eukaryotic translation initiation factor	AW469966
ESTs	AW972759
Junk, low PCR	EMPTY
Junk, low PCR	EMPTY
UDP glycosyltransferase 2 family, polype	BF689099
protein (peptidyl-prolyl cis/trans isome	BF000059
myosin IXB	NM_004145
translocation protein 1	XM_002971
desmoglein 3 (pemphigus vulgaris antigen	NM_001944
P450 (cytochrome) oxidoreductase	AF258341
Homo sapiens mRNA; cDNA DKFZp434C0820 (f	AW341691
ESTs	AI810024
serologically defined colon cancer antig	AF039697
gastrulation brain homeo box 2	BF115559

keratin, hair, basic, 3	NM_002282
ras responsive element binding protein 1	AA968736
UDP-Gal:betaGlcNAc beta 1,3-galactosyltr	AF154848
syntrophin, alpha 1(dystrophin-associate	AL550070
Junk, low PCR	EMPTY
epoxide hydrolase 1, microsomal (xenobio	NM_000120
CD36 antigen (collagen type I receptor,	AL581011
Junk, low PCR	EMPTY
coagulation factor VIII-associated (intr	AL582270
vascular cell adhesion molecule 1	AL037831
mutated in colorectal cancers	NM_002387
toll-like receptor 2	NM_003264
mitogen-activated protein kinase kinase	X90846
hypothetical protein DKFZp434E2216	AI453069
Junk, low PCR	EMPTY
ESTs	BG429420
proline arginine-rich end leucine-rich r	NM_002725
enoyl Coenzyme A hydratase 1, peroxisoma	AI628513
fucose-1-phosphate guanylyltransferase	XM_001491
chromosome 21 open reading frame 4	BF797654
KIAA1528 protein	AA284881
pre-mRNA splicing factor similar to S. c	AF038391
MADS box transcription enhancer factor 2	L08895
origin recognition complex, subunit 2 (y	BF109758
mitochondrial translational initiation f	NM_002453
ESTs, Weakly similar to Z263_HUMAN ZINC	AW006067
Homo sapiens clone 23860 mRNA sequence	AW069649
protein phosphatase 5, catalytic subunit	BC001970
minichromosome maintenance deficient (S.	AU149507
procollagen-lysine, 2-oxoglutarate 5-dio	NM_000935
glycosylphosphatidylinositol specific ph	L11702
homolog of yeast ubiquitin-protein ligas	BE391083
polymyositis/scleroderma autoantigen 1 (	AA580641
ESTs	AA889797
properdin P factor, complement	AW236221
phosphodiesterase 6A, cGMP-specific, rod	NM_000440
homeo box D1	NM_024501
myotubularin related protein 1	BF110149
esterase D/formylglutathione hydrolase	AA827229
myeloid differentiation primary response	AW965179
Junk, low PCR	EMPTY
ryanodine receptor 1 (skeletal)	AC005933
breakpoint cluster region	BF528055
hypothetical protein FLJ20333	R27552
protein phosphatase 2A, regulatory subun	AU125210
cyclin D2	NM_001759
ESTs, Moderately similar to 2020260A his	AW148944
Homo sapiens PAK2 mRNA, complete cds	AF092132
baculoviral IAP repeat-containing 4	NM_001167
kinesin family member 3B	NM_004798
Junk, low PCR	EMPTY

ESTs	AI917371
LIM domain binding 2	AU141400
Homo sapiens DNA, cosmid clones TN62 and	AW962223
arginyltransferase 1	AF079099
rho/rac guanine nucleotide exchange fact	BG248725
KIAA0871 protein	NM_014961
protein phosphatase 1, catalytic subunit	BG289736
H.sapiens mRNA for elongation factor-1-g	EMPTY
Microfibril-associated glycoprotein-2	AL048540
bleomycin hydrolase	AA044158
Junk, low PCR	EMPTY
BN51 (BHK21) temperature sensitivity com	NM_001722
nuclear transport factor 2 (placental pr	BE409333
EST	N59792
fatty acid binding protein 7, brain	AI369673
Homo sapiens mRNA; cDNA DKFZp586N0121 (f	AL133118
NAD(P) dependent steroid dehydrogenase-l	AL519589
KIAA0430 gene product	AB007890
ESTs	AA044052
ESTs	AA160871
gamma-aminobutyric acid (GABA) A recepto	NM_000811
peptidylprolyl isomerase (cyclophilin)-l	BG387775
radical fringe (Drosophila) homolog	BE250669
KIAA0849 protein	AI362018
ribosomal protein S6 kinase, 90kD, polyp	NM_002953
Human mRNA for calmodulin	EMPTY
nuclear factor of activated T-cells, cyt	AC004531
Homo sapiens mRNA from chromosome 5q21-2	AB002448
Junk, low PCR	EMPTY
cytochrome P450, subfamily XIA (choleste	AA143153
X-prolyl aminopeptidase (aminopeptidase	BG341619
ESTs, Weakly similar to ALUC_HUMAN !!!!	BF573849
TG-interacting factor (TALE family homeo	AL549846
ESTs	BF112168
antigen identified by monoclonal antibod	AA928468
interferon, alpha-inducible protein 27	BG535739
ESTs	W63702
ESTs	AI039268
testis specific protein, Y-linked	NM_003308
lymphoid nuclear protein related to AF4	BE676320
A kinase (PRKA) anchor protein (gravin)	XM_004539
Junk, low PCR, low PCR	EMPTY
ring finger protein 1	AL576514
Human U1 snRNP-specific protein A gene	EMPTY
amylo-1,6-glucosidase, 4-alpha-glucanotr	XM_001700
annexin A1	BG541130
KIAA1292 protein	AI265848
vacuolar protein sorting 45B (yeast homo	BF796127
KIAA0391 gene product	NM_014672
CGI-83 protein	BF793554
actin binding LIM protein 1	NM_006719

Homo sapiens clone 23570 mRNA sequence	AA975205
phosphoribosyl pyrophosphate synthetase-	NM_002767
atrophin-1 interacting protein 4	AF038564
hypothetical protein	AA971154
Homo sapiens cDNA: FLJ22642 fis, clone H	AK026295
ESTs	AI350755
KIAA0477 gene product	XM_010723
transcriptional adaptor 2 (ADA2, yeast,	AL526501
Junk, low PCR, low PCR	EMPTY
cyclin-dependent kinase 3	NM_001258
Human mRNA for eukaryotic initiation fac	EMPTY
protein phosphatase 2 (formerly 2A), reg	AL389975
creatine kinase, mitochondrial 1 (ubiqui	BE873170
Junk, low PCR	EMPTY
complement component 8, gamma polypeptid	U08198
Junk, low PCR	EMPTY
ESTs	AW440369
calponin 2	AU124309
Homo sapiens AD034 mRNA, complete cds	AI651413
proteasome (prosome, macropain) subunit,	BC000268
nuclear factor (erythroid-derived 2)-lik	NM_004289
ESTs	AA290901
ESTs	AW167404
ESTs, Weakly similar to ALU7_HUMAN ALU S	AA478625
cullin 5	NM_003478
phenylalanine-tRNA synthetase-like	AU148249
v-maf musculoaponeurotic fibrosarcoma (a	XM_012691
protein disulfide isomerase related prot	NM_004911
Human ADP-ribosylation factor mRNA, comp	EMPTY
manic fringe (Drosophila) homolog	AA760630
malic enzyme 1, NADP(+)-dependent, cytos	NM_002395
Junk, low PCR	EMPTY
Junk, low PCR	EMPTY
ribosomal protein L26	BG259955
calcitonin gene-related peptide-receptor	AI202801
PCTAIRE protein kinase 1	AW949594
KIAA0202 protein	AA987764
parathymosin	AA459196
RNA binding motif protein 9	NM_014309
RCE1, prenyl protein protease (yeast hom	AF121951
Homo sapiens, clone IMAGE:3611719, mRNA,	BG257698
gamma-aminobutyric acid (GABA) A recepto	AI801878
NADH dehydrogenase (ubiquinone) 1, subco	AW965948
matrix metalloproteinase 19	BE857968
Junk, low PCR	EMPTY
calpain 2, (m/II) large subunit	NM_001748
Human mRNA (HA0643) for ORF (Canis oligo	EMPTY
Junk, low PCR	EMPTY
SA (rat hypertension-associated) homolog	AI632754
hypothetical protein FLJ14033 similar to	AU119698
glutaminyl-tRNA synthetase	AA312973

aldo-keto reductase family 7, member A2	AI869298
ankyrin repeat-containing protein ASB-2	AA195189
proteolipid protein 2 (colonic epitheliu	BF035725
KIAA0943 protein	AW170330
chloride channel, nucleotide-sensitive,	AU143440
villin-like	NM_015873
EST	R44120
KIAA1534 protein	AI061267
nucleolar protein p40; homolog of yeast	W07797
ephrin-A3	NM_004952
KIAA0895 protein	AB020702
Junk, low PCR	EMPTY
Homo sapiens clone DT1P1A10 mRNA, CAG re	BE744766
Empty	EMPTY
protein phosphatase 3 (formerly 2B), cat	AA504039
Homo sapiens ARTS protein (PNUTL2) mRNA,	AF176379
UDP-glucose dehydrogenase	NM_003359
Junk, low PCR	EMPTY
Junk, low PCR	EMPTY
hypothetical protein FLJ22313	AI625386
plexin B3	XM_010173
ESTs	AW088230
FYN-binding protein (FYB-120/130)	AF198052
dermatan sulphate proteoglycan 3	AI128100
cleavage and polyadenylation specific fa	AA203670
ESTs	AA039479
Junk, low PCR	EMPTY
kinectin 1 (kinesin receptor)	AI916662
proenkephalin	AI459701
arginine vasopressin receptor 2 (nephrog	AF101728
cofilin 2 (muscle)	BE503345
Empty	EMPTY
zinc finger protein 197	NM_006991
pyruvate dehydrogenase (lipoamide) beta	BC000439
general transcription factor IIH, polype	AF078847
trichohyalin	L09190
ELAV (embryonic lethal, abnormal vision,	R55730
Junk, low PCR	EMPTY
Junk, low PCR	EMPTY
ESTs	AI696501
glucan (1,4-alpha-), branching enzyme 1	L07956
Junk, low PCR	EMPTY
hypothetical protein DKFZp761C07121	NM_017594
ESTs	AA137230
calcium/calmodulin-dependent protein kin	AI925424
ubiquinol-cytochrome c reductase binding	BG025705
nuclear pore complex interacting protein	AI025901
Junk, low PCR	EMPTY
metaxin 2	AW237281
Empty	EMPTY
Junk, low PCR	EMPTY

protease, cysteine, 1 (legumain)	NM_005606
translation factor sui1 homolog	AA693947
FXYD domain-containing ion transport reg	AA826766
small inducible cytokine subfamily B (Cy	NM_001565
ESTs, Weakly similar to ALU1_HUMAN ALU S	AA280866
insulin-like growth factor binding prote	AL542262
F-box only protein 22	AI193151
adaptor protein with pleckstrin homology	NM_020979
Junk, low PCR	EMPTY
ESTs	AW024492
Homo sapiens clone 23929 mRNA sequence	AI004937
KIAA0445 gene product	AL537590
Human BRCA2 region, mRNA sequence CG006	U50535
dynein, axonemal, heavy polypeptide 17	AL122077
Junk, low PCR	EMPTY
ubiquitin-activating enzyme E1C (homolog	XM_003039
Empty	EMPTY
ESTs, Highly similar to ITF2_HUMAN TRANS	AA936434
mitogen-activated protein kinase kinase	AF042838
KIAA0290 protein	AB006628
Junk, low PCR	EMPTY
Junk, low PCR	EMPTY
dimethylarginine dimethylaminohydrolase	AA454088
myelin-associated oligodendrocyte basic	AV726230
Homo sapiens cDNA FLJ11375 fis, clone HE	AI005282
tumor necrosis factor, alpha-induced pro	AI028304
upregulated by 1,25-dihydroxyvitamin D-3	AU139227
DR1-associated protein 1 (negative cofac	AW293621
chloride intracellular channel 4	AI084276
lymphocyte adaptor protein	BE276268
serologically defined colon cancer antig	BC001149
Splicing factor, arginine/serine-rich, 4	AF031165
pancreatic zymogen granule membrane asso	AB035541
vav 1 oncogene	BE049420
Empty	EMPTY
mitogen-activated protein kinase kinase	XM_008654
F-box only protein 24	AL136811
sterol carrier protein 2	AA662425
Junk, low PCR	EMPTY
ZAP3 protein	BE176466
KIAA0713 protein	AA908770
insulin-like growth factor 1 receptor	NM_000875
peptide deformylase-like protein	AI859289
aspartyl-tRNA synthetase	AU123068
Junk, low PCR	EMPTY
Homo sapiens mRNA; cDNA DKFZp434E0626 (f	AL137364
ESTs	AW295838
protein arginine N-methyltransferase 3(h	AI986271
H.sapiens gene from PAC 106H8	AL035301
serine protease inhibitor, Kunitz type 1	AL557359
Junk, low PCR	EMPTY

Homo sapiens cDNA FLJ20153 fis, clone CO	AA807673
Empty	EMPTY
transducin-like enhancer of split 2, hom	NM_003260
REMOVED_FROM_DATABASE	EMPTY
KIAA0373 gene product	NM_014684
glutamate receptor, ionotropic, N-methyl	NM_000835
Junk, low PCR	EMPTY
Homo sapiens mRNA; cDNA DKFZp667I103 (fr	R55801
kallikrein B, plasma (Fletcher factor) 1	AW471415
KIAA0376 protein	BE646204
pre-mRNA splicing factor 17	NM_015891
small inducible cytokine subfamily A (Cy	AW965098
ESTs	AW268404
Homo sapiens cDNA FLJ12371 fis, clone MA	AK022433
a disintegrin-like and metalloprotease (	NM_021599
hypothetical protein similar to cadherin	AF035300
HCGII-7 protein	X81001
Junk, low PCR	EMPTY
stanniocalcin 2	AB012664
KIAA0964 protein	BG033853
Homo sapiens mRNA; cDNA DKFZp586K1123 (f	AA262763
cardiac ankyrin repeat protein	NM_014391
activating transcription factor 6	AW372543
ataxia-telangiectasia group D-associated	AA131550
Homo sapiens mRNA; cDNA DKFZp586E1124 (f	AW512676
Junk, low PCR	EMPTY
core-binding factor, runt domain, alpha	AL121906
nuclear transcription factor, X-box bind	BG327231
ADP-ribosyltransferase (NAD+; poly (ADP-	NM_006437
Junk, low PCR	EMPTY
phosphoinositide-specific phospholipase	AL049593
BCL2-associated athanogene	AA628507
neutrophil cytosolic factor 1 (47kD, chr	AI682248
H2A histone family, member A	AV763030
catenin (cadherin-associated protein), a	BF793401
ubiquitin specific protease 10	BE543108
guanine nucleotide binding protein (G pr	NM_006578
ESTs	AA968940
vacuolar protein sorting 29 (yeast homol	AA722987
solute carrier family 9 (sodium/hydrogen	M96067
farnesyltransferase, CAAX box, alpha	AL574829
Homo sapiens development and differentia	AW451462
KIAA0672 gene product	AA706335
Junk, low PCR	EMPTY
aldo-keto reductase family 1, member B11	NM_004812
cadherin 19, type 2	NM_021153
ubiquitin carboxyl-terminal esterase L3	AA513206
lysosomal-associated membrane protein 1	XM_007184
lipase A, lysosomal acid, cholesterol es	NM_000235
Junk, low PCR	EMPTY
butyrate response factor 1 (EGF-response	AU127757

G-2 and S-phase expressed 1	AI298140
CREB binding protein (Rubinstein-Taybi s	AW370316
karyopherin alpha 1 (importin alpha 5)	U20620
lectin, mannose-binding, 1	AA057813
ESTs	Z44764
Homo sapiens C1orf19 mRNA, partial cds	AI524582
BRCA1 associated protein-1 (ubiquitin ca	BG109611
cartilage oligomeric matrix protein (pse	NM_000095
similar to rat HREV107	AI375733
ESTs	AA970499
deiodinase, iodothyronine, type II	AU118870
L-3-hydroxyacyl-Coenzyme A dehydrogenase	BF340259
gastric inhibitory polypeptide	XM_008460
selectin L (lymphocyte adhesion molecule	NM_000655
KIAA0914 gene product	AF009202
phosphorylase kinase, alpha 1 (muscle)	AI366791
DEAD/H (Asp-Glu-Ala-Asp/His) box polypep	AW351829
PDZ domain containing guanine nucleotide	AI092560
solute carrier family 6 (neurotransmitte	XM_001465
RAN binding protein 8	BG388312
putative mitochondrial outer membrane pr	AU152183
MYLE protein	AW073158
ESTs	AA972074
hypothetical protein FLJ13119	AA418693
regucalcin (senescence marker protein-30	NM_004683
retinol-binding protein 4, interstitial	AI207655
peripheral myelin protein 22	AI144212
No ID Incyte EST	EMPTY
solute carrier family 22 (organic cation	NM_002555
PC4 and SFRS1 interacting protein 2	AW192949
tight junction protein 2 (zona occludens	NM_004817
thioredoxin, mitochondrial	AI862181
integral membrane protein 1	NM_002219
cyclin-dependent kinase inhibitor 2C (p1	NM_001262
integrin-linked kinase	XM_006424
isocitrate dehydrogenase 1 (NADP+), solu	AI284981
cadherin, EGF LAG seven-pass G-type rece	AB011536
Homo sapiens clone 24488 mRNA sequence	AI918131
pyruvate dehydrogenase kinase, isoenzyme	AU131650
KIAA0084 protein	AL561563
MAD (mothers against decapentaplegic, Dr	AA081871
hypothetical protein FLJ23209	AI241095
HIV-1 rev binding protein 2	BE380146
pleiotrophin (heparin binding growth fac	AU118193
LIM and senescent cell antigen-like doma	NM_004987
tRNA isopentenylpyrophosphate transferas	AW966003
Junk, low PCR	EMPTY
mal, T-cell differentiation protein	NM_022439
kinesin-like 1	NM_004523
KIAA0155 gene product	NM_014633
sodium channel, voltage-gated, type I, b	XM_009325

forkhead box O3A	AU134033
platelet factor 4	AI739539
absent in melanoma 1	AA574200
Homo sapiens cDNA: FLJ23602 fis, clone L	AF060511
hypothetical protein FLJ22530	BG340369
spectrin SH3 domain binding protein 1	AF006516
zinc finger protein 8 (clone HF.18)	AI671579
hypothetical protein DKFZp434E2135	AK024850
ESTs	AA599692
ilvB (bacterial acetolactate synthase)-l	XM_009304
KIAA0305 gene product	BE645239
Human clone 137308 mRNA, partial cds	BF997727
ESTs	AA233762
neuronal pentraxin I	NM_002522
vinculin	NM_014000
membrane-spanning 4-domains, subfamily A	XM_006275
Junk, low PCR	EMPTY
carboxypeptidase M	NM_001874
Junk, low PCR	EMPTY
interferon-induced, hepatitis C-associat	XM_001628
No ID Incyte EST	EMPTY
acetyl LDL receptor; SREC	AI623132
putative L-type neutral amino acid trans	AK025649
HLA-B associated transcript-3	AW245105
KIAA0683 gene product	NM_016111
Human mRNA for 3-oxoacyl-CoA peroxisomal	EMPTY
Junk, low PCR	EMPTY
Homo sapiens clone 24812 mRNA sequence	AA947660
splicing factor, arginine/serine-rich 2,	NM_004719
stromal antigen 3	AI572906
No ID Incyte EST	EMPTY
bromodomain-containing 3	AW953714
solute carrier family 2 (facilitated glu	NM_001042
ESTs	W37338
Junk, low PCR	EMPTY
chromosome 11 open reading frame2	AI539419
kallikrein 6 (neurosin, zyme)	NM_002774
DKFZP434I116 protein	AL559272
huntingtin interacting protein 2	AI022979
ribosomal protein L23	AI349581
hepatocellular carcinoma associated prot	BF969633
Junk, low PCR, low PCR	EMPTY
MADS box transcription enhancer factor 2	NM_005920
H.sapiens mRNA for alpha-centractin	EMPTY
chromosome 11open reading frame 11	AB014559
KIAA1587 protein	N66646
splicing factor, arginine/serine-rich 11	AA169732
DKFZP434P1750 protein	AA484742
zinc finger protein 189	AW192671
protein kinase, interferon-inducible dou	AW083130
caveolin 2	AI458487

ESTs, Weakly similar to ALU1_HUMAN ALU S	F12749
statherin	M18371
lipopolysaccharide specific response-68	AK024978
RAP2A, member of RAS oncogene family	BF794939
Homo sapiens cDNA: FLJ22106 fis, clone H	AI040790
calcium channel, voltage-dependent, beta	AL565681
Junk, low PCR	EMPTY
mutS (E. coli) homolog 5	AL122094
carbonyl reductase 3	AI658832
cathepsin D (lysosomal aspartyl protease	AI031979
Human mercurial-insensitive water channe	EMPTY
postsynaptic protein CRIPT	BE748814
histone deacetylase 7A	AK026767
Junk, low PCR	EMPTY
ESTs	BE295812
hypothetical protein FLJ10913	AI309555
potassium inwardly-rectifying channel, s	U39196
ribosomal protein L19	BF691720
low density lipoprotein receptor-related	N71497
keratin 13	BC002661
epithelial V-like antigen 1	AW874086
argininosuccinate lyase	BF316073
ESTs	AI192537
glutaminase	XM_002709
hypocretin (orexin) neuropeptide precurs	NM_001524
WD-repeat protein	BE296528
presenilin 1 (Alzheimer disease 3)	AW376952
jumonji (mouse) homolog	AW076028
Human mRNA for 26S proteasome subunit p9	EMPTY
membrane-spanning 4-domains, subfamily A	BF108683
ESTs	AA782778
inner membrane protein, mitochondrial (m	BG164144
Junk, low PCR	EMPTY
stromal cell protein	AV703538
phospholipase A2 receptor 1, 180kD	AW614826
transmembrane 4 superfamily member 2	AL568153
RAB2, member RAS oncogene family	AA749237
KIAA1036 protein	BE349025
ESTs	BE349129
APEX nuclease (multifunctional DNA repai	AI207650
inorganic pyrophosphatase	BG170080
myosin IB	BE395925
leucine-rich, glioma inactivated 1	NM_005097
STAT induced STAT inhibitor 3	AI922872
B-cell CLL/lymphoma 6 (zinc finger prote	AI954940
chromogranin B (secretogranin 1)	AW247798
Human sterol 27-hydroxylase (CYP27) mRNA	EMPTY
Homo sapiens cDNA FLJ13155 fis, clone NT	AA664156
Golgi apparatus protein 1	AI632109
enhancer of rudimentary (Drosophila) hom	BG504520
KIAA0652 gene product	AA568469

Junk, low PCR	EMPTY
potassium channel, subfamily K, member 1	NM_002245
receptor tyrosine kinase-like orphan rec	NM_005012
ESTs	AA702138
isoprenylcysteine carboxyl methyltransfe	AL580586
aspartyl aminopeptidase	BC000653
KIAA0144 gene product	NM_014847
hypothetical protein FLJ21347	BF339524
polymerase (RNA) II (DNA directed) polyp	AW086431
Junk, low PCR	EMPTY
ras-related C3 botulinum toxin substrate	NM_005052
expressed in activated T/LAK lymphocytes	BF759621
KIAA0013 gene product	NM_014783
Human histone H2B.1 mRNA, 3' end	EMPTY
hypothetical protein	NM_016553
CGI-145 protein	AA418388
uncoupling protein 2 (mitochondrial, pro	AA897099
golgi SNAP receptor complex member 1	AW970923
SRp25 nuclear protein	BG110393
G protein-coupled receptor 17	BF346549
protein phosphatase 1, regulatory (inhib	NM_006241
phosphate cytidylyltransferase 1, cholin	AI767533
bystin-like	XM_004156
Homo sapiens cDNA: FLJ21962 fis, clone H	AA219620
KIAA0539 gene product	NM_014825
ESTs	AI868274
gap junction protein, beta 1, 32kD (conn	AI492172
leucine-rich neuronal protein	XM_004694
Homo sapiens clone 23809 mRNA sequence	AI651122
transcription elongation factor B (SIII)	AW070971
hypothetical protein 24432	AI272922
Arabadopsis8-250	EMPTY
zinc finger protein 45 (a Kruppel-associ	NM_003425
cofactor required for Sp1 transcriptiona	AA452411
ataxia telangiectasia and Rad3 related	U49844
Junk, low PCR	EMPTY
ribosomal protein S27 (metallopanstimuli	AI253609
interferon (alpha, beta and omega) recep	AL544600
beta-transducin repeat containing	AF129530
Homo sapiens mRNA; cDNA DKFZp761E13121 (	AI698258
KIAA1435 protein	BE301038
type I transmembrane protein Fn14	AI313436
mitogen-activated protein kinase 10	T75436
VAMP (vesicle-associated membrane protei	AU153750
gap junction protein, beta 1, 32kD (conn	AW300531
SEC24 (S. cerevisiae) related gene famil	XM_003603
microsomal NAD+-dependent retinol dehydr	XM_006765
mesoderm development candidate 2	AK026606
complement component 6	NM_000065
Arabadopsis10-250	EMPTY
protein kinase, cAMP-dependent, catalyti	NM_002731

zinc finger protein 24 (KOX 17)	AA721989
thyroid hormone receptor interactor 13	BE090548
KIAA0620 protein	AL527865
calsequestrin 2 (cardiac muscle)	NM_001232
golgi autoantigen, golgin subfamily b, m	NM_004487
transforming growth factor, alpha	XM_002732
ESTs	AA644408
albumin	N24732
DKFZP586B2420 protein	BE796027
Junk, low PCR	EMPTY
Homo sapiens cDNA FLJ13226 fis, clone OV	AW029320
proline-rich Gla (G-carboxyglutamic acid	XM_008983
prepronociceptin	BE378858
hypothetical protein	NM_012066
DEAD/H (Asp-Glu-Ala-Asp/His) box polypep	BE439603
platelet-derived growth factor receptor-	AL541699
Empty	EMPTY
Junk, low PCR	EMPTY
complement component C1q receptor	AI937452
ribosomal protein L7	AV740966
Homo sapiens clone 24921 mRNA sequence	H45848
leptin (murine obesity homolog)	NM_000230
Breakpoint cluster region protein, uteri	BF237719
Junk, low PCR	EMPTY
ESTs	AA043381
hypothetical protein PRO1580	R72857
hypothetical protein FLJ11856	AI399827
interferon induced transmembrane protein	BF699055
ESTs	AI888220
Junk, low PCR	EMPTY
melanoma antigen, family A, 12	L18877
cadherin 12, type 2 (N-cadherin 2)	NM_004061
hematopoietically expressed homeobox	XM_005888
UBX domain-containing 1	AI702899
Empty	EMPTY
ESTs, Weakly similar to RSU1_HUMAN RAS S	AA724544
hypothetical protein MGC3265	AA701491
xeroderma pigmentosum, complementation g	NM_000380
TRF2-interacting telomeric RAP1 protein	BG428020
splicing factor, arginine/serine-rich 4	NM_005626
kynurenine 3-monooxygenase (kynurenine 3	Y13153
phosphodiesterase 6D, cGMP-specific, rod	XM_002246
ESTs	W87927
BTB (POZ) domain containing 2	AW195430
NRAS-related gene	BF001217
complement component 3	NM_000064
ESTs	BE218830
cyclic nucleotide gated channel alpha 1	AI800043
filamin C, gamma (actin-binding protein-	AF146692
TNF receptor-associated factor 2	NM_021138
spectrin, beta, non-erythrocytic 2	BF034402

MORF-related gene X	AU147348
Empty	EMPTY
ESTs	AA826199
lamin B receptor	N93426
ribosomal protein S26	AI075912
ESTs	R83912
Homo sapiens, Similar to dodecenoyl-Coen	BE464854
Bruton agammaglobulinemia tyrosine kinas	AL035422
syntaphilin	NM_014723
Junk, low PCR	EMPTY
RAB2, member RAS oncogene family	AW301641
ESTs	BF060905
uroporphyrinogen decarboxylase	AL548063
ESTs	BE463603
checkpoint suppressor 1	AA777764
regenerating islet-derived 1 beta (pancr	AW951196
dipeptidylpeptidase VI	NM_001936
chromosome 11 open reading frame 13	AL544102
ARP3 (actin-related protein 3, yeast) ho	AL040940
Empty	EMPTY
ESTs	AA700590
Homo sapiens ubiquitin protein ligase (U	AW589568
serine (or cysteine) proteinase inhibito	AL550163
No ID Incyte EST	EMPTY
zinc finger protein 211	NM_006385
membrane cofactor protein (CD46, trophob	Y00651
5'-nucleotidase (purine), cytosolic type	NM_012229
carbonyl reductase	AA433946
Homo sapiens LYST-interacting protein LI	AF141342
CGI-83 protein	AA863239
solute carrier family 35 (CMP-sialic aci	AU135905
hypothetical protein FLJ20425	AI708352
stratifin	BE734249
KIAA0878 protein	AU138104
serine (or cysteine) proteinase inhibito	AL542381
vacuolar protein sorting 41 (yeast homol	U87281
stannin	AW247309
Junk, low PCR	EMPTY
ESTs, Weakly similar to ALUC_HUMAN !!!!	AW590793
heat shock 70kD protein 5 (glucose-regul	AL043206
ESTs	AA522544
synaptogyrin 3	AL534610
Junk, low PCR	EMPTY
polymerase (RNA) II (DNA directed) polyp	BE389988
peptidyl-prolyl isomerase G (cyclophilin	AW299990
spectrin, alpha, non-erythrocytic 1 (alp	XM_005369
vesicle docking protein p115	AV715367
hypothetical protein MGC5178	AI862457
Homo sapiens cDNA: FLJ22063 fis, clone H	BF000639
nucleolar protein p40; homolog of yeast	BG476679
RNA binding motif protein, Y chromosome,	NM_005058

tumor necrosis factor (ligand) superfami	AF064090
zinc finger protein 220	AA777633
peroxisomal biogenesis factor 11B	AI743597
cleavage and polyadenylation specific fa	AL520678
RAP1, GTPase activating protein 1	XM_001822
hypothetical protein PRO1489	AI536671
proteasome (prosome, macropain) activato	BG387808
ESTs	AI869974
RNA 3'-terminal phosphate cyclase	NM_003729
nuclear cap binding protein subunit 2, 2	BG403935
2,4-dienoyl CoA reductase 1, mitochondri	AF069291
solute carrier family 4, anion exchanger	BF002399
faciogenital dysplasia (Aarskog-Scott sy	XM_010215
arsA (bacterial) arsenite transporter, A	AL519675
Junk, low PCR	EMPTY
ESTs	AA135958
EST, Moderately similar to ORF derived f	AA946800
potassium voltage-gated channel, KQT-lik	NM_004519
nuclear transcription factor Y, alpha	AL031778
Homo sapiens cDNA: FLJ21897 fis, clone H	AW296083
hypothetical protein	BF732636
tubulin, alpha 1 (testis specific)	BE742772
coatomer protein complex, subunit beta 2	NM_004766
Junk, low PCR	EMPTY
KIAA0138 gene product	BE856759
Junk, low PCR	EMPTY
keratin 7	AA307373
chaperonin containing TCP1, subunit 6A (	NM_001762
calcitonin-related polypeptide, beta	AI937230
arachidonate 5-lipoxygenase	AI799008
torsin family 1, member B (torsin B)	AF317129
potassium large conductance calcium-acti	XM_003644
calnexin	BF966833
nascent-polypeptide-associated complex a	AI819147
cytochrome P450 isoform 4F12	AB035131
E2F transcription factor 2	NM_004091
coenzyme Q, 7 (rat, yeast) homolog	AI393585
GLE1 (yeast homolog)-like, RNA export me	BG284700
dachshund (Drosophila) homolog	NM_004392
DiGeorge syndrome critical region gene 6	BC000682
BENE protein	AI808771
ribosomal protein L18a	F28484
baculoviral IAP repeat-containing 2	XM_006266
Junk, low PCR	EMPTY
ATP-binding cassette, sub-family E (OABP	NM_002940
interleukin enhancer binding factor 1	AL577802
small inducible cytokine subfamily B (Cy	BC005292
integrin, beta 4	BE735810
centaurin beta2	D26069
microfibrillar-associated protein 4	AL569291
male-specific lethal-3 (Drosophila)-like	BF057183

hypothetical protein	AL357533
non-histone chromosome protein 2 (S. cer	AL157644
No ID Incyte EST	EMPTY
methyl-CpG binding domain protein 3	BE799139
acid phosphatase, prostate	X52174
Homo sapiens mRNA; cDNA DKFZp434I0812 (f	N92548
small inducible cytokine B subfamily (Cy	NM_006419
carnitine acetyltransferase	XM_005616
glutamate receptor, ionotropic, kainate	H15417
holocarboxylase synthetase (biotin-[prop	AP000697
Junk, low PCR	EMPTY
Junk, low PCR	EMPTY
chromosome 18 open reading frame 1	NM_004338
interferon-stimulated protein, 15 kDa	BG484172
Human PAC clone RP3-515N1 from 22q11.2-q	BE262782
limbic system-associated membrane protei	NM_002338
Homo sapiens clone 23783 mRNA sequence	AF054996
ESTs	AI298599
pre-mRNA splicing SR protein rA4	AI937268
suppression of tumorigenicity 5	NM_005418
frizzled (Drosophila) homolog 9	NM_003508
origin recognition complex, subunit 4 (y	AU131934
myosin, heavy polypeptide-like (110kD)	XM_006761
KIAA0650 protein	BG111493
bromodomain-containing 4	NM_014299
KIAA0222 gene product	W03196
Homo sapiens clone 23551 mRNA sequence	AI206408
Junk, low PCR	EMPTY
Junk, low PCR	EMPTY
poly(A)-binding protein, cytoplasmic 4 (	NM_003819
Sjogren's syndrome nuclear autoantigen 1	BC004118
neuropilin 1	AF280547
baculoviral IAP repeat-containing 3	U37546
BarH-like homeobox 2	AJ243512
collagen, type IX, alpha 3	AI459547
No ID Incyte EST	EMPTY
Junk, low PCR	EMPTY
RAB6A, member RAS oncogene family	AF119836
KIAA0851 protein	XM_003253
Fc fragment of IgG, receptor, transporte	AL571972
Junk, low PCR	EMPTY
Homo sapiens clones 24714 and 24715 mRNA	R56641
adenosine deaminase, RNA-specific	NM_015840
Human uroporphyrinogen III synthase mRNA	EMPTY
L1 cell adhesion molecule (hydrocephalus	NM_000425
Junk, low PCR	EMPTY
osmosis responsive factor	NM_012382
MAX protein	BC003525
v-erb-b2 avian erythroblastic leukemia v	NM_001982
replication factor C (activator 1) 3 (38	AA907044
insulin induced gene 1	BC001880

heparan sulfate (glucosamine) 3-O-sulfot	AI342806
small nuclear ribonucleoprotein polypept	AA742521
Homo sapiens mRNA from chromosome 5q21-2	AB002438
Junk, low PCR	EMPTY
sentrin/SUMO-specific protease	AA279764
hypothetical protein FLJ20320	NM_017765
X-prolyl aminopeptidase (aminopeptidase	XM_010251
KIAA0622 protein; Drosophila	AB014522
Homo sapiens cDNA FLJ13384 fis, clone PL	AK023446
nuclear domain 10 protein	AA463834
Homo sapiens adenylyl cyclase-associated	EMPTY
testis-specific ankyrin motif containing	AW780029
Duffy blood group	AF055992
Junk, low PCR	EMPTY
kinesin-like 5 (mitotic kinesin-like pro	H63163
Ras association (RalGDS/AF-6) domain fam	NM_014737
aryl hydrocarbon receptor nuclear transl	AI473905
opiate receptor-like 1	AL574931
histone deacetylase 7B	AA629911
heat shock 70kD protein 1B	AF134726
glycophorin A (includes MN blood group)	AI133438
ESTs	AI791162
chromosome 6 open reading frame 11	AI739276
intercellular adhesion molecule 4, Lands	NM_001544
absent in melanoma 2	NM_004833
S-adenosylmethionine decarboxylase 1	BF676785
Junk, low PCR, low PCR	EMPTY
DEAD/H (Asp-Glu-Ala-Asp/His) box polypep	U41387
Human mRNA for phospholipase C-alpha, co	EMPTY
HS1 binding protein	AL523082
Junk, low PCR	EMPTY
ariadne (Drosophila) homolog 2	AW206596
tumor necrosis factor, alpha-induced pro	XM_002762
ferrochelatase (protoporphyria)	AL526206
Homo sapiens mRNA; cDNA DKFZp761B1514 (f	AL136558
GABAA receptor gamma 3 subunit [human, f	S82769
KIAA1466 protein	AA460333
ribosomal protein S23	BG331575
insulin promoter factor 1, homeodomain t	NM_000209
hypothetical protein FLJ20113	AL526404
Junk, low PCR	EMPTY
Human clone 23564 mRNA sequence	U90910
acetyl LDL receptor; SREC	NM_003693
WW domain binding protein 4 (formin bind	AV685394
Junk, low PCR, low PCR	EMPTY
calpain 1, (mu/I) large subunit	BE907919
Human chromatin assembly factor-I p60 su	EMPTY
KIAA0241 protein	D87682
acylphosphatase 1, erythrocyte (common)	AW958424
ESTs	AA725502
KIAA0062 protein	D31887

hydroxyacyl-Coenzyme A dehydrogenase, ty	AU154299
Junk, low PCR	EMPTY
general transcription factor IIIA	XM_007218
Homo sapiens, clone IMAGE:3834272, mRNA,	BC004869
catenin (cadherin-associated protein), a	AL120743
small nuclear RNA activating complex, po	BG028846
ESTs	BF195738
CGI-68 protein	BG110799
Human Chromosome 16 BAC clone CIT987SK-A	N51995
protocadherin gamma subfamily A, 1	AF152507
death effector domain-containing	AJ010973
homeo box D9	BE219356
GTP-binding protein homologous to Saccha	AL552252
Human cytoplasmic chaperonin hTRiC5 mRNA	EMPTY
killer cell lectin-like receptor subfami	AW612744
pinin, desmosome associated protein	Y10351
Junk, low PCR	EMPTY
alanine-glyoxylate aminotransferase (oxa	NM_000030
growth arrest and DNA-damage-inducible,	AI718055
Homo sapiens cDNA: FLJ21869 fis, clone H	BF185587
protein kinase C substrate 80K-H	BF725749
KIAA1025 protein	BE466662
KIAA0286 protein	AB006624
gastrulation brain homeo box 1	L11239
hypothetical protein	AV729065
mitogen-activated protein kinase kinase	BE350101
major histocompatibility complex, class	BE168491
KIAA0469 gene product	AL043058
nuclear factor of activated T-cells, cyt	U08015
Junk, low PCR, low PCR	EMPTY
collagen, type V, alpha 2	NM_000393
Human ubiquitin-activating enzyme E1 (UB	EMPTY
SEC10 (S. cerevisiae)-like 1	BE089535
erythrocyte membrane protein band 4.1-li	BF799654
ESTs	AW631160
zinc finger protein 263	NM_005741
choline kinase	XM_006098
Junk, low PCR	EMPTY
transducer of ERBB2, 1	NM_005749
hypothetical protein FLJ10936	AI915925
Junk, low PCR	EMPTY
autoimmune regulator (automimmune polyen	AJ009610
ESTs	BG386640
Junk, low PCR	EMPTY
mouse double minute 2, human homolog of;	U33199
ubiquitin A-52 residue ribosomal protein	AV708340
golgi autoantigen, golgin subfamily a, 5	NM_005113
Junk, low PCR, low PCR	EMPTY
Junk, low PCR	EMPTY
Arabadopsis2-250	EMPTY
solute carrier family 34 (sodium phospha	NM_003052

transmembrane trafficking protein	BE561078
mevalonate (diphospho) decarboxylase	BG324529
Junk, low PCR	EMPTY
nuclear factor I/C (CCAAT-binding transc	BE563136
RAN binding protein 7	BF432106
androgen receptor (dihydrotestosterone r	NM_000044
ESTs	AA149736
REMOVED_FROM_DATABASE	EMPTY
Junk, low PCR	EMPTY
anaphase-promoting complex subunit 7	BF060903
ESTs, Moderately similar to ALU5_HUMAN A	BF198006
interferon-stimulated transcription fact	AU155099
interleukin 11	NM_000641
xenotropic and polytropic retrovirus rec	AL133058
mucin 1, transmembrane	AW369450
SH3 domain binding glutamic acid-rich pr	AI422208
Arabadopsis4-250	EMPTY
4-hydroxyphenylpyruvate dioxygenase	NM_002150
KIAA0074 protein	D38553
eukaryotic translation initiation factor	BE960808
ATP-binding cassette, sub-family A (ABC1	XM_001290
peptidylprolyl isomerase F (cyclophilin	AL531948
Homo sapiens cDNA: FLJ22096 fis, clone H	AA541417
renal tumor antigen	BF037188
alpha-methylacyl-CoA racemase	XM_003872
Human D9 splice variant B mRNA, complete	AI278347
Junk, low PCR	EMPTY
ESTs	AA461302
ESTs, Weakly similar to ALU1_HUMAN ALU S	BF108970
dual specificity phosphatase 11 (RNA/RNP	AI478561
ribosomal protein S21	D52096
KIAA0767 protein	BF917126
Junk, low PCR	EMPTY
ESTs	AA702952
Arabadopsis6-250	EMPTY
adrenergic, alpha-1B-, receptor	NM_000679
endonuclease G	AW250109
heat shock transcription factor 4	AI741735
Junk, low PCR	EMPTY
Junk, low PCR	EMPTY
CGI-85 protein	XM_006125
diaphanous (Drosophila, homolog) 2	XM_013068
hypothetical protein FLJ12701	AU160615
gastrointestinal peptide	NM_014471
Junk, low PCR	EMPTY
ESTs, Moderately similar to ALU4_HUMAN A	AA527159
ESTs	AI365505
aryl hydrocarbon receptor	AA844153
a disintegrin and metalloproteinase doma	AA043347
Junk, low PCR	EMPTY
Junk, low PCR	EMPTY

extracellular matrix protein 2, female o	NM_001393
Empty	EMPTY
Homo sapiens unknown mRNA, sequence	BE781764
KIAA0522 protein	AB011094
H factor 1 (complement)	AU136270
parathyroid hormone	W39202
complement component 5	XM_005340
KIAA1708 protein	AI520742
Homolog of yeast DNA-crosslink repair ge	D42045
hypothetical protein	AW612524
Junk, low PCR	EMPTY
glycoprotein A33 (transmembrane)	NM_005814
ATP synthase, H+ transporting, mitochond	AI311103
hypothetical protein FLJ11136	NM_018336
promyelocytic leukemia	AA522918
Homo sapiens clone 24507 mRNA sequence	AF052148
KIAA0864 protein	N31673
prostaglandin F receptor (FP)	XM_001630
GTPase Rab14	AA906459
Empty	EMPTY
KIAA0101 gene product	AV716856
cytochrome c	AI625569
deformed epidermal autoregulatory factor	AF068897
CD8 antigen, beta polypeptide 1 (p37)	NM_004931
makorin, ring finger protein, 1	NM_013446
PAN2 protein	XM_010748
mitochondrial intermediate peptidase	AA524277
ESTs	AI651814
guanine nucleotide binding protein (G pr	NM_002071
nuclear factor of kappa light polypeptid	BG469491
ESTs	AA912133
Homo sapiens mRNA; cDNA DKFZp564N2464 (f	AA988352
KIAA0375 gene product	AI184624
CD33 antigen (gp67)	XM_005083
KIAA0467 protein	BE818955
Junk, low PCR	EMPTY
vacuolar proton pump delta polypeptide	H82183
Empty	EMPTY
Junk, low PCR	EMPTY
No ID Incyte EST	EMPTY
hemoglobin, gamma A	XM_006556
adenosine kinase	NM_006721
proline oxidase 2	XM_009830
Junk, low PCR	EMPTY
for protein disulfide isomerase-related	AL559582
Junk, low PCR	EMPTY
fucosyltransferase 1 (galactoside 2-alph	AW664940
melanoma antigen, family A, 4	U10687
ESTs	AI143921
papillomavirus regulatory factor PRF-1	AL526737
KIAA0252 protein	D87440

transient receptor potential channel 7	XM_009803
NY-REN-24 antigen	BE301421
Junk, low PCR	EMPTY
hypothetical protein FLJ11282	XM_007885
Homo sapiens cDNA: FLJ23005 fis, clone L	AK026658
suppression of tumorigenicity 7	AY009153
niban protein	BE645822
voltage-gated sodium channel beta-3 subu	AI089593
Kruppel-like factor 1 (erythroid)	AI040588
secretin receptor	NM_002980
hairy/enhancer-of-split related with YRP	AI421289
distal-less homeo box 5	XM_004848
ATP-binding cassette, sub-family B (MDR/	X06181
male-specific lethal-3 (Drosophila)-like	AI207004
lymphocyte cytosolic protein 2 (SH2 doma	NM_005565
activating transcription factor 1	AI004969
uroporphyrinogen III synthase (congenita	BF913759
phosphotidylinositol transfer protein, b	AL035843
Human protocadherin 42 mRNA, 3' end of c	L11369
interleukin 13 receptor, alpha 1	AI816791
sterol regulatory element binding transc	BG340581
eukaryotic translation initiation factor	XM_010474
seven in absentia (Drosophila) homolog 1	AI498404
stress-induced-phosphoprotein 1 (Hsp70/H	BG469870
two-pore channel 1, homolog	AA722704
ESTs	AA813723
G protein pathway suppressor 1	BG169279
glutathione S-transferase A4	BF701560
SEC22, vesicle trafficking protein (S. c	AI253626
ubiquitously transcribed tetratricopepti	AU139244
immunoglobulin-like transcript 8	NM_024318
hypothetical protein FLJ20121	AA010695
Junk, low PCR	EMPTY
ESTs	AW294859
protein tyrosine kinase 9-like (A6-relat	BF220316
membrane protein, palmitoylated 2 (MAGUK	AW450911
hepatic leukemia factor	AF035305
Rho-specific guanine nucleotide exchange	AB011093
KIAA0372 gene product	NM_014639
fragile X mental retardation 2	X95463
Junk, low PCR	EMPTY
KIAA0090 protein	AI131158
Homo sapiens cDNA: FLJ21333 fis, clone C	AI912413
KIAA1361 protein	W84433
copper chaperone for superoxide dismutas	NM_005125
dipeptidase 1 (renal)	NM_004413
Homo sapiens, Similar to hypothetical pr	AI867454
sperm associated antigen 8	NM_012436
thymidine kinase 1, soluble	BE271054
mannosidase, alpha, class 1A, member 2	N30111
prostaglandin-endoperoxide synthase 1 (p	S36219

Homo sapiens cDNA: FLJ23582 fis, clone L	H98817
enoyl-Coenzyme A, hydratase/3-hydroxyacy	XM_003155
claudin 7	AI279608
eukaryotic translation initiation factor	BE702316
TTK protein kinase	AU154042
cell division cycle 2-like 1 (PITSLRE pr	AI889200
mitogen-activated protein kinase 8	NM_002750
pleiomorphic adenoma gene-like 2	XM_009541
ATP synthase, H+ transporting, mitochond	AA527110
ESTs	AA004679
NGFI-A binding protein 1 (ERG1 binding p	AW952348
C18B11 homolog (44.9kD)	AL578605
ribosomal protein S20	BE727685
prostate cancer associated protein 1	AV727349
deleted in malignant brain tumors 1	NM_004406
fibronectin leucine rich transmembrane p	XM_006111
Homo sapiens mRNA; cDNA DKFZp547M072 (fr	AI554777
plectin 1, intermediate filament binding	NM_000445
Homo sapiens, Similar to leucine rich re	BC004958
solute carrier family 22 (organic cation	NM_003060
immediate early protein	BG255669
cytochrome P450, subfamily XIA (choleste	NM_000781
small EDRK-rich factor 1A (telomeric)	AF073518
chaperonin containing TCP1, subunit 5 (e	BG479968
troponin T2, cardiac	XM_001917
hairy (Drosophila)-homolog	BG290521
KIAA0089 protein	D42047
ESTs	AA514863
ESTs	AA507382
preferentially expressed antigen in mela	AI017284
protease, serine, 11 (IGF binding)	AL542818
No ID Incyte EST	EMPTY
Junk, low PCR	EMPTY
alpha-fetoprotein	AA334424
chromosome 1 open reading frame 21	AA533099
cell adhesion molecule with homology to	NM_006614
No ID Incyte EST	EMPTY
No ID Incyte EST	EMPTY
Homo sapiens cDNA FLJ14354 fis, clone Y7	AF055084
rhomboid (veinlet, Drosophila)-like	AJ272344
potassium intermediate/small conductance	BE397619
nuclear protein	NM_014497
VPS28 protein	BF663123
HRIHFB2206 protein	BF689587
nucleotide binding protein 1 (E.coli Min	XM_008031
uncharacterized hypothalamus protein HT0	AV704367
ESTs, Weakly similar to 16.7Kd protein [	BE644889
KIAA0268 protein	BE549553
B-cell associated protein	AV707380
ESTs	R13843
teratocarcinoma-derived growth factor 1	AU124747

desmoplakin (DPI, DPII)	NM_004415
Junk, low PCR	EMPTY
Junk, low PCR	EMPTY
tumor differentially expressed 1	BE740914
CCAAT/enhancer binding protein (C/EBP),	W39546
lymphoid-restricted membrane protein	NM_006152
No ID Incyte EST	EMPTY
Junk, low PCR	EMPTY
SCO (cytochrome oxidase deficient, yeast	AI332708
KIAA0296 gene product	NM_014699
Human protein kinase mRNA, complete cds	EMPTY
Homo sapiens mRNA; cDNA DKFZp434D0935 (f	AL117502
Junk, low PCR	EMPTY
ESTs	AA102645
Junk, low PCR	EMPTY
testis-specific kinase 1	NM_006285
Homo sapiens cDNA FLJ13221 fis, clone NT	AA912872
jerky (mouse) homolog-like	NM_003772
ATP-binding cassette, sub-family D (ALD)	XM_012350
hypothetical protein	AL578626
KIAA1468 protein	BF061509
Homo sapiens cDNA: FLJ22495 fis, clone H	BF195098
polymerase (DNA-directed) kappa	AA843527
Spi-B transcription factor (Spi-1/PU.1 r	XM_008993
Junk, low PCR	EMPTY
neuronatin	AW161393
Junk, low PCR, low PCR	EMPTY
mitogen-activated protein kinase kinase	NM_005043
H.sapiens mRNA for a cell surface protei	EMPTY
interleukin 3 receptor, alpha (low affin	AI636533
Junk, low PCR	EMPTY
ESTs, Moderately similar to ALU7_HUMAN A	N66178
forkhead box J1	XM_008137
apolipoprotein C-II	AV690565
zinc ribbon domain containing, 1	AI890173
Homo sapiens cDNA: FLJ23037 fis, clone L	AK026690
Pseudoautosomal GTP-binding protein-like	AL543887
Homo sapiens cDNA FLJ10158 fis, clone HE	AA486551
hypothetical protein	AW058345
hypothetical protein FLJ11280	AB037811
No ID Incyte EST	EMPTY
LIM domain kinase 2	NM_005569
sciellin	AK025320
sodium channel, nonvoltage-gated 1, beta	AJ005383
BCL2-like 1	BE780536
antigen identified by monoclonal antibod	AX009576
Human superoxide dismutase (SOD-1) mRNA,	EMPTY
KIAA0275 gene product	NM_014767
BCE-1 protein	AA489676
hypothetical protein FLJ20839	AI655182
Junk, low PCR	EMPTY

AXL receptor tyrosine kinase	NM_021913
PRO2047 protein	AA767511
KIAA0136 protein	AP000693
suppression of tumorigenicity 16 (melano	AW008256
similar to Caenorhabditis elegans protei	AW470642
prostate cancer associated protein 1	AW009886
Link guanine nucleotide exchange factor	BF436933
ESTs	AI446543
CD59 antigen p18-20 (antigen identified	AI829016
NK-receptor	NM_015868
baculoviral IAP repeat-containing 5 (sur	AL571008
transmembrane protein 1	XM_009794
ret proto-oncogene (multiple endocrine n	M31213
Human mRNA for mitochondrial 3-ketoacyl-	EMPTY
guanine nucleotide binding protein (G pr	AU142979
protein tyrosine phosphatase, receptor t	AL049570
RNB6	AW967052
mitogen-activated protein kinase 12	NM_002969
Junk, low PCR	EMPTY
PTD017 protein	AA628011
complement component 7	AL551659
troponin I, skeletal, slow	BF671724
KIAA0913 protein	BE049539
ESTs	W88769
Homo sapiens cDNA FLJ13474 fis, clone PL	AI147417
LUC7 (S. cerevisiae)-like	H24669
REMOVED_FROM_DATABASE	EMPTY
phosphoenolpyruvate carboxykinase 1 (sol	BG401803
chloride channel 7	BE276854
CD79B antigen (immunoglobulin-associated	M80461
phosphoglycerate mutase 1 (brain)	AL546245
Unknown - Human Control Plate Well H8	EMPTY
alpha-2-macroglobulin	AU119825
sine oculis homeobox (Drosophila) homolo	AI031679
fusion, derived from t(12;16) malignant	D53304
Junk, low PCR	EMPTY
EphB2	BE796053
Junk, low PCR	EMPTY
heterogeneous nuclear ribonucleoprotein	AL134884
KIAA0542 gene product	BE043980
Homo sapiens cDNA: FLJ21042 fis, clone C	AK024695
hypothetical protein FLJ13441	AI090843
No ID Incyte EST	EMPTY
XAGE-1 protein	AW975988
peptidase D	AL547859
KIAA0449 protein	AB007918
ESTs, Moderately similar to GLK5_HUMAN G	BG151527
KIAA0095 gene product	XM_012552
integrin, alpha 9	NM_002207
Unknown - Human Control Plate Well H12	EMPTY
COX15 (yeast) homolog, cytochrome c oxid	XM_005811

cryptochrome 1 (photolyase-like)	D83702
Homo sapiens colon cancer antigen NY-CO-	AA404231
thyroid hormone receptor binding protein	AK027040
prostate tumor over expressed gene 1	AW966798
ESTs	AW013911
protease, serine, 22	XM_006625
glycogenin 2	NM_003918
F-box only protein 9	AL031178
hypothetical protein FLJ10422	BF035587
Junk, low PCR	EMPTY
Homo sapiens KIAA0846 protein (KIAA0846)	AW968327
calcium/calmodulin-dependent protein kin	AI492596
non-metastatic cells 2, protein (NM23B)	BF058614
cholinergic receptor, nicotinic, alpha p	NM_000742
ATP-binding cassette, sub-family D (ALD)	BE274237
COX11 (yeast) homolog, cytochrome c oxid	XM_008627
Empty	EMPTY
O-linked N-acetylglucosamine (GlcNAc) tr	NM_003605
density-regulated protein	AW402876
hypothetical protein FLJ20073	AI826495
Junk, low PCR	EMPTY
excision repair cross-complementing rode	AI928779
ESTs, Weakly similar to AF151840 1 CGI-8	W76105
KIAA1105 protein	AW418517
sudD (suppressor of bimD6, Aspergillus n	AU137473
minichromosome maintenance deficient (S.	AU131348
hypothetical protein FLJ14054	AA999973
Junk, low PCR	EMPTY
KIAA1538 protein	AA782766
ras GTPase activating protein-like	XM_001455
RAD51-interacting protein	BE244869
trans-Golgi network protein (46, 48, 51k	AW627777
POU domain, class 2, transcription facto	AW501434
etoposide-induced mRNA	BE740116
Empty	EMPTY
KIAA0260 protein	AA682653
ESTs	AA485438
electron-transfer-flavoprotein, alpha po	N24973
KIAA0185 protein	D80007
histidyl-tRNA synthetase	AA911991
ESTs	AA115931
hypothetical protein MGC3136	AI347229
Homo sapiens clone 23771 mRNA sequence	AL134453
ubiquitin specific protease 9, X chromos	AU118783
thyroid receptor interacting protein 15	AA490664
integrin, alpha 2 (CD49B, alpha 2 subuni	BE501907
Homo sapiens CocoaCrisp mRNA, complete c	AF142573
mitogen-activated protein kinase kinase	NM_003954
regulator of G-protein signalling 20	AA242973
H2B histone family, member B	AL353759
heme oxygenase (decycling) 1	AU129800

ret finger protein 2	AL526890
Empty	EMPTY
Junk, low PCR	EMPTY
ESTs	AV653605
Homo sapiens cDNA: FLJ23068 fis, clone L	AW002018
peroxisomal biogenesis factor 6	NM_000287
SFRS protein kinase 1	AW958364
Homo sapiens cDNA FLJ12908 fis, clone NT	AU128882
duodenal cytochrome b	AL136693
KIAA0562 gene product	NM_014704
KIAA0416 protein	NM_015564
Homo sapiens mRNA; cDNA DKFZp761D221 (fr	AL136561
retinoblastoma-like 1 (p107)	XM_009643
ESTs, Weakly similar to ALU1_HUMAN ALU S	AI051697
KIAA0884 protein	AB020691
actin, alpha 2, smooth muscle, aorta	AI088671
minichromosome maintenance deficient (S.	AU124152
CD7 antigen (p41)	AW408337
KIAA0742 protein	AA524505
Empty	EMPTY
small nuclear ribonucleoprotein D2 polyp	BF382008
hypothetical protein FLJ21016	NM_025160
Homo sapiens cDNA FLJ12366 fis, clone MA	AW054922
Junk, low PCR	EMPTY
Fc fragment of IgG binding protein	NM_003890
ESTs	BE219328
hypothetical protein FLJ22174	AW440843
azurocidin 1 (cationic antimicrobial pro	X58794
heterogeneous nuclear ribonucleoprotein	BE535239
ESTs	AA494538
SON DNA binding protein	AW328025
Homo sapiens BBS2 (BBS2) mRNA, complete	N56946
BCL2-antagonist/killer 1	AW276601
breast carcinoma amplified sequence 2	BG527737
KIAA0753 gene product	XM_008537
Junk, low PCR	EMPTY
insulin-like growth factor binding prote	BE615089
Empty	EMPTY
Ric (Drosophila)-like, expressed in neur	NM_002930
ESTs	N51335
eukaryotic translation initiation factor	AI948487
Junk, low PCR	EMPTY
KIAA0266 gene product	NM_021645
Homo sapiens cDNA: FLJ21482 fis, clone C	AA535580
ESTs, Weakly similar to T12682 hypotheti	N62487
KIAA0399 protein	AB007859
15 kDa selenoprotein	AA447415
ESTs	AI207250
microtubule-associated protein 1B	BE935831
ESTs	N95414
transcriptional adaptor 3 (ADA3, yeast h	BG389122

special AT-rich sequence binding protein	BE266904
KIAA0793 gene product	NM_014808
vascular endothelial growth factor B	BF128614
KIAA0250 gene product	AL137800
Empty	EMPTY
glycogen synthase kinase 3 beta	AL521664
ESTs	AA856830
linked to Surfeit genes in Fugu rubripes	BF038648
leukemia inhibitory factor (cholinergic	NM_002309
ribosomal protein S15	BF680826
Kallmann syndrome 1 sequence	AA678961
carbamoyl-phosphate synthetase 1, mitoch	AA342271
phosphoglycerate kinase 1	BG333707
cytochrome P450, subfamily IIC (mephenyt	M17398
Junk, low PCR	EMPTY
solute carrier family 6 (neurotransmitte	XM_003051
ESTs	AA824363
calcium channel, voltage-dependent, alph	NM_000722
Deleted in split-hand/split-foot 1 regio	BE045195
sialyltransferase 8 (alpha-N-acetylneura	NM_003034
3-hydroxymethyl-3-methylglutaryl-Coenzym	AL519340
sialyltransferase 9 (CMP-NeuAc:lactosylc	BF223269
ribosomal protein S4, Y-linked	BF795455
transcription factor binding to IGHM enh	NM_006521
Homo sapiens cDNA FLJ13427 fis, clone PL	AA279392
Junk, low PCR	EMPTY
interferon (alpha, beta and omega) recep	AU137565
Homo sapiens cDNA FLJ13443 fis, clone PL	AI433385
GTP cyclohydrolase 1 (dopa-responsive dy	AV655113
primase, polypeptide 1 (49kD)	BC005266
chromodomain helicase DNA binding protei	BG109201
Junk, low PCR	EMPTY
hypothetical protein FLJ11164	AI363497
HSPC033 protein	AW510702
stomatin (EBP72)-like 1	XM_007643
homeo box A9	BE899575
CD5 antigen (p56-62)	XM_006041
Homo sapiens cDNA FLJ13540 fis, clone PL	AK023602
adenylate cyclase 8 (brain)	NM_001115
ATPase, H+ transporting, lysosomal (vacu	BE899262
retinal degeneration, slow (retinitis pi	M62958
eukaryotic translation initiation factor	BG527919
ESTs	BE549953
Junk, low PCR	EMPTY
Junk, low PCR	EMPTY
zinc finger protein 277	BF064022
mitochondrial capsule selenoprotein	NM_030663
7-dehydrocholesterol reductase	BE378962
Junk, low PCR	EMPTY
Junk, low PCR	EMPTY
ESTs	AI139145

Homo sapiens cDNA: FLJ21425 fis, clone C	AK025078
keratin, hair, acidic,1	Y16787
chromosome 12 open reading frame	AJ298133
chaperonin containing TCP1, subunit 6B (	NM_006584
Junk, low PCR	EMPTY
cullin 3	AF062537
RAN binding protein 6	AF039023
pregnancy specific beta-1-glycoprotein 1	M20881
butyrophilin, subfamily 3, member A3	NM_006994
3-hydroxyisobutyryl-Coenzyme A hydrolase	AI276059
neurofilament, heavy polypeptide (200kD)	XM_009907
serine hydroxymethyltransferase 2 (mitoc	NM_005412
ribosomal protein L3	AI823777
Homo sapiens KB07 protein mRNA, partial	AF064606
Junk, low PCR	EMPTY
Junk, low PCR	EMPTY
HSPC009 protein	BE886906
polymerase (RNA) II (DNA directed) polyp	BF436055
ESTs	R53193
KIAA0214 gene product	XM_001745
peroxisomal acyl-CoA thioesterase	AI628256
Dmx-like 1	BG431794
H1 histone family, member X	BE746713
TAP binding protein (tapasin)	BE394234
v-akt murine thymoma viral oncogene homo	AU130605
phosphoglucomutase 1	NM_002633
heterogeneous nuclear ribonucleoprotein	BE153958
hippocalcin-like protein 4	AI052684
Junk, low PCR	EMPTY
hect domain and RLD 2	AF041080
L2DTL protein	AU153372
Ste-20 related kinase	AA456870
ribosomal protein L6	BG476350
Junk, low PCR	EMPTY
Homo sapiens mRNA; cDNA DKFZp586C1817 (f	AL133574
DKFZP586G1122 protein	AI270296
ESTs	AA448003
Junk, low PCR	EMPTY
serine (or cysteine) proteinase inhibito	AA316156
cleavage and polyadenylation specific fa	AI089205
KIAA0792 gene product	AK022502
ATPase, Ca++ transporting, ubiquitous	AF068221
BTG family, member 2	NM_006763
PRO0233 protein	XM_002149
pancreatitis-associated protein	NM_002580
estrogen-responsive B box protein	AI539776
Junk, low PCR	EMPTY
vav 2 oncogene	AL045952
Homo sapiens clone 022g11 My031 protein	AA614696
lymphocyte antigen 64 (mouse) homolog, r	AL570708
protein kinase C, eta	XM_007374

hypothetical protein B	NM_014262
ESTs	BF433763
ESTs, Highly similar to A46297 beta-1,6-	AI478652
ESTs	AI150075
RNA polymerase I transcription factor RR	AA977114
transforming, acidic coiled-coil contain	AW778997
H1 histone family, member 3	AL031777
RNA polymerase II transcriptional regula	AL079635
KIAA0088 protein	BG285617
Kallmann syndrome 1 sequence	NM_000216
phosphoinositide-3-kinase, class 3	NM_002647
proliferating cell nuclear antigen	AA523378
ESTs	AA809496
hydroxysteroid (17-beta) dehydrogenase 4	AU125131
sialyltransferase 1 (beta-galactoside al	AU141656
phosphatidylinositol glycan, class L	XM_008276
conserved gene amplified in osteosarcoma	BG034382
acyl-Coenzyme A dehydrogenase, C-2 to C-	NM_000017
Junk, low PCR	EMPTY
Junk, low PCR	EMPTY
ESTs	AI370989
ESTs	AA970059
metastasis-associated 1-like 1	NM_004739
SGC32445 protein	AF070551
period (Drosophila) homolog 1	BG394132
NADH dehydrogenase (ubiquinone) Fe-S pro	AL524829
chaperonin containing TCP1, subunit 3 (g	BE731258
collagen, type IV, alpha 5 (Alport syndr	NM_000495
Human cytosolic aspartate aminotransfera	EMPTY
prostatic binding protein	BG252637
Junk, low PCR	EMPTY
Junk, low PCR	EMPTY
Junk, low PCR	EMPTY
axin 2 (conductin, axil)	AI279470
Friend leukemia virus integration 1	XM_006215
Thy-1 cell surface antigen	N94350
ESTs	AA258794
ESTs, Moderately similar to A36065 prote	AI141608
v-ski avian sarcoma viral oncogene homol	AA514329
Human DNA sequence from clone RP5-1007G1	AI191623
ESTs	N47089
serine (or cysteine) proteinase inhibito	XM_001452
a disintegrin and metalloproteinase doma	AF158644
dTDP-D-glucose 4,6-dehydratase	AI923250
scrapie responsive protein 1	AI588970
ISL1 transcription factor, LIM/homeodoma	NM_002202
Human 90 kD heat shock protein gene, com	EMPTY
CDC7 (cell division cycle 7, S. cerevisi	AF015592
complement component 1, q subcomponent,	AI879461
epithelial membrane protein 1	AW265568
guanine nucleotide binding protein 4	AW593228

likely ortholog of mouse variant polyade	AI872408
chromosome 21 open reading frame 2	NM_004928
ecotropic viral integration site 2A	XM_008466
hypothetical protein FLJ11506	AI150416
ESTs, Highly similar to unnamed protein	AA400002
Rho guanine nucleotide exchange factor (	AA452145
MUM2 protein	BG474734
hypothetical protein FLJ10704	H98082
non-histone chromosome protein 2 (S. cer	BF528185
SPARC-like 1 (mast9, hevin)	NM_004684
pleiotropic regulator 1 (PRL1, Arabidops	NM_002669
sin3-associated polypeptide, 18kD	AL570795
megakaryocyte-associated tyrosine kinase	AC005777
Human mRNA for mitochobdrial enoyl-CoA h	EMPTY
chemokine (C-C motif) receptor 7	XM_008651
UDP-Gal:betaGlcNAc beta 1,4- galactosylt	AF097159
matrix metalloproteinase 7 (matrilysin,	Z11887
DEAD/H (Asp-Glu-Ala-Asp/His) box polypep	BG546189
ESTs	BE007322
tubulin, beta polypeptide	BE729764
collagen, type I, alpha 1	AW577407
Homo sapiens chromosome 19, cosmid F2216	AI050793
ESTs	AA715324
hypothetical protein MGC2655	AA825589
ESTs	AA703048
EST	N66318
neural cell adhesion molecule 1	AW768954
A kinase (PRKA) anchor protein 4	NM_003886
adenosine deaminase	BG257096
neuroblastoma (nerve tissue) protein	NM_006334
alanyl (membrane) aminopeptidase (aminop	NM_001150
Human mRNA for eukaryotic initiation fac	EMPTY
calponin 1, basic, smooth muscle	AL046845
RNA guanylyltransferase and 5'-phosphata	AW069237
nuclear protein, marker for differentiat	AI955152
catechol-O-methyltransferase	BE799904
KIAA1077 protein	AA665820
N-ethylmaleimide-sensitive factor	AL535356
melanoma antigen, family A, 1 (directs e	NM_004988
Homo sapiens testis-specific protein TSP	AA758749
ESTs, Highly similar to BimL [H.sapiens]	AA629308
ESTs	BG054744
chondroitin sulfate proteoglycan 5 (neur	AW451747
ESTs	AI079331
calpain 6	BG427471
peptidylprolyl isomerase D (cyclophilin	NM_005038
KIAA0500 protein	AI915271
TBP-interacting protein	AI914159
Ras-GTPase activating protein SH3 domain	AU125944
Human ADP-ribosylation factor mRNA, comp	EMPTY
aldo-keto reductase family 1, member A1	AW873466

small inducible cytokine subfamily A (Cy	XM_005633
Junk, low PCR	EMPTY
leukotriene A4 hydrolase	AI636026
ESTs	AI346089
colony stimulating factor 3 (granulocyte	M17706
serologically defined colon cancer antig	AA508745
Homo sapiens clone TCCCIA00427 mRNA sequ	N93201
putative ankyrin-repeat containing prote	AI969135
KIAA0472 protein	AA401745
No ID Incyte EST	EMPTY
ESTs	AI097560
betaine-homocysteine methyltransferase	BG433459
tyrosine 3-monooxygenase/tryptophan 5-mo	AA182818
Fas-activated serine/threonine kinase	AL563739
Human transcription unit PVT gene, exons	AI498125
Junk, low PCR	EMPTY
Human mRNA (HA0643) for ORF (Canis oligo	EMPTY
dual-specificity tyrosine-(Y)-phosphoryl	AL561586
chitinase 3-like 1 (cartilage glycoprote	AL035737
dedicator of cyto-kinesis 1	NM_001380
heat shock protein 75	BE901723
Homo sapiens mRNA for KIAA1754 protein,	AA425726
inositol 1,4,5-triphosphate receptor, ty	NM_002223
mammaglobin 1	NM_002411
Homo sapiens mRNA; cDNA DKFZp564B2123 (f	R61354
ESTs	BF433962
Junk, low PCR	EMPTY
ESTs	AA676952
golgin-67	AW168774
small inducible cytokine subfamily A (Cy	NM_002988
Homo sapiens clone FLC0675 PRO2870 mRNA,	AA333314
v-kit Hardy-Zuckerman 4 feline sarcoma v	BF435112
chemokine (C-X3-C) receptor 1	U20350
cystatin A (stefin A)	AI680589
Empty	EMPTY
Junk, low PCR	EMPTY
coagulation factor XIII, B polypeptide	NM_001994
ESTs	AI695956
Junk, low PCR	EMPTY
interleukin 2	S82692
guanine nucleotide-releasing factor 2 (s	NM_005312
deleted in azoospermia	AI732428
hypothetical protein FLJ23590	N93061
zinc finger protein 35 (clone HF.10)	AI809774
WNT5b protein	BF056538
hypothetical protein	XM_003567
ESTs, Weakly similar to ALU5_HUMAN ALU S	BE274637
KIAA0768 protein	AF307080
Homo sapiens mRNA for single-chain antib	AL560682
small nuclear RNA activating complex, po	XM_005594
95 kDa retinoblastoma protein binding pr	AI868427

translocase of outer mitochondrial membr	NM_014765
Empty	EMPTY
fibroblast growth factor 1 (acidic)	X59065
KIAA0354 gene product	NM_014872
pyrroline 5-carboxylate reductase isofor	BE042497
Hin-1	XM_003554
Rho GTPase activating protein 5	NM_001173
cytochrome b-245, alpha polypeptide	BG421245
Junk, low PCR	EMPTY
ESTs	AI018205
glypican 5	NM_004466
ESTs	BF725123
Homo sapiens, Similar to hypothetical pr	AA988306
ESTs, Weakly similar to ALU1_HUMAN ALU S	AI302221
Homo sapiens mRNA for C11ORF25 gene	N90336
acid phosphatase, prostate	AA654793
cyclin-dependent kinase inhibitor 2D (p1	BE855885
5-hydroxytryptamine (serotonin) receptor	AL049576
chaperonin containing TCP1, subunit 4 (d	AU124605
Empty	EMPTY
zinc finger protein 85 (HPF4, HTF1)	NM_003429
myosin VIIA (Usher syndrome 1B (autosoma	NM_000260
ESTs	AA621029
vesicle-associated membrane protein 8 (e	AI367976
KIAA0226 gene product	AI360844
Junk, low PCR	EMPTY
ATPase, H+ transporting, lysosomal (vacu	BG470373
retinoblastoma-binding protein 1	NM_023000
Junk, low PCR	EMPTY
nucleoporin 214kD (CAIN)	BF435507
No ID Incyte EST	EMPTY
ESTs	AW085301
cystatin B (stefin B)	BE736122
sulfortranferase family 4A, member 1	AF251263
KIAA0808 gene product	BG499021
ESTs, Weakly similar to ALU6_HUMAN ALU S	N59757
PDZ and LIM domain 1 (elfin)	AI687946
Empty	EMPTY
adenylyl cyclase-associated protein	BG286995
dual specificity phosphatase 8	XM_012007
Junk, low PCR	EMPTY
postmeiotic segregation increased 2-like	D38440
flavin containing monooxygenase 3	XM_010961
RAN binding protein 2	AL136868
tumor necrosis factor receptor superfami	NM_001250
Pyruvate dehydrogenase complex, lipoyl-c	AL553066
PR domain containing 2, with ZNF domain	XM_001536
ESTs	AA398118
ESTs, Weakly similar to ALU1_HUMAN ALU S	R66525
NS1-associated protein 1	BF508615
haptoglobin	AL564563

No ID Incyte EST	EMPTY
heat shock 70kD protein 9B (mortalin-2)	BG427082
zinc finger protein 217	AA507833
chondroitin sulfate proteoglycan 4 (mela	NM_001897
Empty	EMPTY
Junk, low PCR	EMPTY
vascular endothelial growth factor C	NM_005429
DKFZP586A0522 protein	BG286360
hypothetical protein FLJ10883	AL121581
Junk, low PCR	EMPTY
KIAA0437 protein	NM_015559
homeo box HB9	AI459915
RALBP1 associated Eps domain containing	NM_004726
CD47 antigen (Rh-related antigen, integr	AW470030
KIAA0626 gene product	N62737
Homo sapiens clone 24649 mRNA sequence	AA622394
Homo sapiens PAC clone RP4-751H13 from 7	BE549732
Homo sapiens, Similar to CG2245 gene pro	BC002817
mannosyl (alpha-1,6-)-glycoprotein beta-	NM_002410
solute carrier family 19 (folate transpo	BC003068
zinc finger protein (clone 647)	X16282
Ets2 repressor factor	AA291382
Empty	EMPTY
hematopoietic cell-specific Lyn substrat	AL551154
chromosome 22 open reading frame 1	NM_001585
potassium large conductance calcium-acti	BE672452
DEME-6 protein	AF007170
guanine nucleotide binding protein (G pr	AA099654
polymerase (RNA) II (DNA directed) polyp	BE268036
aldehyde dehydrogenase 3 family, member	AU136629
glutamate decarboxylase 1 (brain, 67kD)	NM_000817
biphenyl hydrolase-like (serine hydrolas	BF448366
ESTs	AA644451
ESTs, Weakly similar to ORF YGL050w [S.c	BF000103
Homo sapiens kruppel-like zinc finger fa	AF226996
inhibitor of kappa light polypeptide gen	AF153419
protocadherin alpha 9	AF152317
No ID Incyte EST	EMPTY
pescadillo (zebrafish) homolog 1, contai	NM_014303
thymosin, beta 10	AV710912
neurogranin (protein kinase C substrate,	BF948983
GATA-binding protein 2	BC002557
Junk, low PCR	EMPTY
kaptin (actin-binding protein)	W85778
REMOVED_FROM_DATABASE	EMPTY
proteasome (prosome, macropain) subunit,	AW007084
LIM domain binding 1	AW271288
presenilin 2 (Alzheimer disease 4)	NM_012486
a disintegrin and metalloproteinase doma	AA399579
ESTs	AI056157
glucosidase I	XM_002747

TSPY-like	AL136629
NADH dehydrogenase (ubiquinone) flavopro	BF313949
actin related protein 2/3 complex, subun	BG483858
leptin receptor gene-related protein	AI762194
Homo sapiens clone 24630 mRNA sequence	AF052174
v-jun avian sarcoma virus 17 oncogene ho	AI078377
phosphoglycerate mutase 2 (muscle)	BE263723
ras-related C3 botulinum toxin substrate	BE314262
hypothetical protein FLJ10788	AL122062
hypothetical protein FLJ10261	AW241356
activating transcription factor 7	AI279868
cytochrome b-5	BG054815
Junk, low PCR	EMPTY
hypothetical protein PRO1853	NM_018607
c-mer proto-oncogene tyrosine kinase	NM_006343
phosphoinositide-3-kinase, regulatory su	M61906
cholinergic receptor, nicotinic, alpha p	AA927784
Junk, low PCR	EMPTY
ESTs	AA934379
adenylate cyclase 7	NM_001114
trefoil factor 2 (spasmolytic protein 1)	AB038162
eukaryotic translation initiation factor	AA477660
endothelial differentiation, lysophospha	AU155011
zinc finger homeobox 1B	NM_014795
Junk, low PCR	EMPTY
spectrin, beta, non-erythrocytic 1	NM_003128
protease, serine, 15	BG481103
No ID Incyte EST	EMPTY
hypothetical protein FLJ22060	AI016103
electron-transfer-flavoprotein, beta pol	BF689926
apolipoprotein C-I	AV709433
CGI-41 protein	AI378484
signal recognition particle 19kD	BG531782
Junk, low PCR	EMPTY
sirtuin (silent mating type information	AI378978
jagged 1 (Alagille syndrome)	U61276
ESTs, Weakly similar to AF151800 1 CGI-4	AI310001
adducin 2 (beta)	NM_017487
nuclear localization signal deleted in v	BG491617
Homo sapiens mRNA for KIAA1727 protein,	AB051514
ATPase, Ca++ transporting, cardiac muscl	AL046795
vav 3 oncogene	XM_010540
KIAA0172 protein	D79994
No ID Incyte EST	EMPTY
general transcription factor IIF, polype	BE779719
Homo sapiens, Similar to hepatoma-derive	BF969745
hypothetical protein	AI918185
reproduction 8	AW474066
mitogen-activated protein kinase kinase	NM_005923
ESTs	AA708609
solute carrier family 15 (H+/peptide tra	NM_021082

leukocyte immunoglobulin-like receptor,	NM_006865
ESTs, Weakly similar to 2109260A B cell	AW275347
monokine induced by gamma interferon	NM_002416
ESTs	AW050488
No ID Incyte EST	EMPTY
dCMP deaminase	AA101974
nuclear receptor coactivator 3	NM_006534
kinase insert domain receptor (a type II	AA393757
von Hippel-Lindau binding protein 1	AU159844
pleckstrin homology, Sec7 and coiled/coi	AJ005197
interleukin 15 receptor, alpha	NM_002189
caudal type homeo box transcription fact	AI738580
MUM2 protein	AI416967
ESTs	AA043900
KIAA0409 protein	NM_015324
glutamate receptor, ionotropic, AMPA 2	NM_000826
Junk, low PCR	EMPTY
phosphatidylinositol-4-phosphate 5-kinas	U78580
tumor necrosis factor receptor superfami	NM_003839
Homo sapiens mRNA; cDNA DKFZp566H2446 (f	AI150949
villin 1	NM_007127
Junk, low PCR	EMPTY
matrix metalloproteinase 23A	XM_001531
hydroxy-delta-5-steroid dehydrogenase, 3	AV707424
transforming growth factor beta 1 induce	AW235866
mitogen-activated protein kinase kinase	AI825772
translational inhibitor protein p14.5	BF126383
hypothetical protein	U79275
deoxyribonuclease I	AI802091
glucose-6-phosphate dehydrogenase	AL570001
Junk, low PCR	EMPTY
Junk, low PCR	EMPTY
mesothelin	AL578289
mitogen-activated protein kinase 3	BC000205
vacuolar protein sorting 33B (yeast homo	BF110489
interferon, gamma-inducible protein 16	BG434340
Human clone 23908 mRNA sequence	U79290
synaptopodin	NM_007286
KIAA0254 gene product	AI127103
ESTs, Weakly similar to ALU1_HUMAN ALU S	BF819920
No ID Incyte EST	EMPTY
membrane protein of cholinergic synaptic	AU141724
chromosome condensation-related SMC-asso	BG030282
forkhead box C1	AL034344
splicing factor 30, survival of motor ne	BG419824
transcription factor 6-like 1 (mitochond	NM_003201
Homo sapiens phosphatidylinositol 4-kina	EMPTY
topoisomerase (DNA) II beta (180kD)	BG287875
stanniocalcin 1	XM_011704
KIAA0677 gene product	BF339879
ATP-binding cassette, sub-family C (CFTR	BF593043

Junk, low PCR	EMPTY
bromodomain and PHD finger containing, 3	AA704529
isoleucine-tRNA synthetase	BE513909
myoglobin	NM_005368
Gene 33/Mig-6	AI546883
ESTs	AA725635
Homo sapiens cDNA FLJ12048 fis, clone HE	AU146960
hypothetical protein	AI025517
WD repeat domain 1	BG034829
glioma-associated oncogene homolog (zinc	NM_005269
N-acylsphingosine amidohydrolase (acid c	AU143425
solute carrier family 13 (sodium-depende	NM_003984
cytochrome c oxidase subunit VIIa polype	AV743565
Human Na/H antiporter (APNH1) mRNA, comp	EMPTY
T cell receptor gamma locus	AI972955
Junk, low PCR	EMPTY
hypothetical protein FLJ10783	AI582366
ribosomal protein L14	AV761980
laminin, alpha 2 (merosin, congenital mu	XM_011387
ESTs	AI138338
Junk, low PCR	EMPTY
SH3-domain binding protein 5 (BTK-associ	BG030766
Homo sapiens cDNA: FLJ21409 fis, clone C	AA503479
PRKC, apoptosis, WT1, regulator	AA527277
DKFZP566A0946 protein	AI422824
ESTs	AW411259
kinesin family member C3	AI961712
polymerase (DNA directed), epsilon 2	AF036899
fibulin 2	AL050095
low density lipoprotein receptor-related	NM_004631
golgi autoantigen, golgin subfamily a, 3	NM_005895
Human mercurial-insensitive water channe	EMPTY
Junk, low PCR	EMPTY
Junk, low PCR	EMPTY
hypothetical protein FLJ22637	AI983685
FK506-binding protein 8 (38kD)	BE908846
Gardner-Rasheed feline sarcoma viral (v-	AI479655
transcription factor	AA026356
lactase	NM_002299
cysteine-rich protein 2	AL515731
ESTs	T78374
UDP-N-acetyl-alpha-D-galactosamine:polyp	BF055258
ESTs	AI190322
Homo sapiens cDNA: FLJ21909 fis, clone H	BF433430
U6 snRNA-associated Sm-like protein LSm7	AA609823
Junk, low PCR	EMPTY
platelet-derived growth factor receptor,	NM_006206
Junk, low PCR, low PCR	EMPTY
phospholipase D2	AF038440
Human nuclear ribonucleoprotein particle	EMPTY
D site of albumin promoter (albumin D-bo	D28468

stromal interaction molecule 1	NM_003156
ESTs	AA559087
KIAA0237 gene product	NM_014747
DnaJ (Hsp40) homolog, subfamily C, membe	BF058544
cysteine sulfinic acid decarboxylase-rel	BF223281
nuclear RNA helicase, DECD variant of DE	AW248283
peroxisomal biogenesis factor 12	AW451678
sterol regulatory element binding transc	BF339929
Homo sapiens cDNA FLJ11489 fis, clone HE	AI208737
hypothetical protein FLJ22251	AA278457
sirtuin (silent mating type information	W95416
Junk, low PCR	EMPTY
KIAA0222 gene product	NM_014643
serologically defined colon cancer antig	AK000694
O-6-methylguanine-DNA methyltransferase	M29971
eukaryotic translation initiation factor	BG499049
Human sterol 27-hydroxylase (CYP27) mRNA	EMPTY
inhibitor of DNA binding 1, dominant neg	AL117381
proprotein convertase subtilisin/kexin t	XM_012963
hypothetical protein MGC2487	AL122095
hyaluronan-mediated motility receptor (R	U29343
nidogen (enactin)	XM_002042
ESTs	AA668326
Junk, low PCR	EMPTY
mannosyl (alpha-1,3-)-glycoprotein beta-	BE742106
hypothetical protein FLJ10099	AU133148
Homo sapiens mRNA; cDNA DKFZp586O2124 (f	BF057380
Junk, low PCR	EMPTY
Homo sapiens cDNA FLJ20222 fis, clone CO	AW182074
histone fold protein CHRAC17; DNA polyme	BG288261
translocase of inner mitochondrial membr	AW105195
transcription factor 7 (T-cell specific,	XM_003836
protein phosphatase 2 (formerly 2A), reg	NM_002717
serum constituent protein	BE296511
Human histone H2B.1 mRNA, 3' end	EMPTY
acid sphingomyelinase-like phosphodieste	AK000184
Junk, low PCR	EMPTY
Homo sapiens mRNA for FLJ00058 protein,	AW204873
metal-regulatory transcription factor 1	NM_005955
alcohol dehydrogenase 5 (class III), chi	AW157342
ESTs	AU151944
death-associated protein 6	BE890313
SH2 domain protein 1A, Duncan's disease	NM_002351
putative Rab5-interacting protein	T23459
Homo sapiens cDNA FLJ10641 fis, clone NT	N78064
KIAA0938 protein	XM_006792
Junk, low PCR	EMPTY
S100 calcium-binding protein P	AI148603
No ID Incyte EST	EMPTY
G protein-coupled receptor	AI375869
PHD finger protein 1	AI669107

phosphoglucomutase 5	NM_021965
Arabadopsis8-125	EMPTY
eukaryotic translation initiation factor	U26032
BH-protocadherin (brain-heart)	AW960791
malic enzyme 1, NADP(+)-dependent, cytos	AL049699
Junk, low PCR	EMPTY
solute carrier family 31 (copper transpo	AA450163
KIAA0666 protein	AI379579
ESTs	AA937212
nitric oxide synthase 2A (inducible, hep	U31511
nuclear matrix protein p84	AV713026
ESTs, Weakly similar to JC1405 6-pyruvoy	AA824511
ras homolog gene family, member G (rho G	BG338917
ESTs	AA971021
No ID Incyte EST	EMPTY
H2B histone family, member A	BE747540
sema domain, immunoglobulin domain (Ig),	AF053369
ATP synthase, H+ transporting, mitochond	AL520959
calponin 3, acidic	BG538900
Arabadopsis10-125	EMPTY
pregnancy-zone protein	NM_002864
KIAA0202 protein	AL532971
KIAA0716 gene product	AI061420
arachidonate 15-lipoxygenase	NM_001140
tousled-like kinase 1	BF447046
contactin 1	AW262854
KIAA0882 protein	AA243858
ash2 (absent, small, or homeotic, Drosop	AB022785
phosphodiesterase 6G, cGMP-specific, rod	NM_002602
hypothetical protein FLJ10773	AA044181
eyes absent (Drosophila) homolog 2	U69178
ESTs	AA983787
acyl-Coenzyme A oxidase 3, pristanoyl	NM_003501
FK506-binding protein 9 (63 kD)	AL555732
solute carrier family 30 (zinc transport	NM_013309
glucuronidase, beta	AI803571
polyadenylate binding protein-interactin	AL548163
Empty	EMPTY
growth factor independent 1	XM_010519
Finkel-Biskis-Reilly murine sarcoma viru	AA316067
Homo sapiens mRNA; cDNA DKFZp761I2123 (f	AL136572
Junk, low PCR	EMPTY
TATA box binding protein (TBP)-associate	XM_011637
ESTs	AA280957
CMP-NeuAC:(beta)-N-acetylgalactosaminide	BG282988
solute carrier family 31 (copper transpo	BG248634
epithelial membrane protein 3	AL541088
Homo sapiens cDNA FLJ11161 fis, clone PL	AK002023
Junk, low PCR	EMPTY
Homo sapiens cDNA: FLJ21409 fis, clone C	AA425619
hypothetical protein	AW006162

Sp4 transcription factor	AW502401
branched chain aminotransferase 2, mitoc	AL526940
death effector domain-containing	H82490
KIAA0781 protein	BF984909
Empty	EMPTY
KIAA0276 protein	D87466
ESTs	R85437
mannosidase, alpha, class 1A, member 2	BF966791
AHNAK nucleoprotein (desmoyokin)	BG473688
cytochrome P450, subfamily IVB, polypept	NM_000779
Junk, low PCR	EMPTY
hypothetical protein from EUROIMAGE 1703	AA583309
heat shock 60kD protein 1 (chaperonin)	BG032173
serum amyloid A4, constitutive	NM_006512
ESTs	AW001886
putative glialblastoma cell differentiat	BE892211
DKFZP586B0621 protein	AW137263
sel-1 (suppressor of lin-12, C.elegans)-	AF052059
melanoma antigen, family A, 2	BG029985
G protein-coupled receptor 30	U63917
microphthalmia-associated transcription	AW673844
AU RNA-binding protein/enoyl-Coenzyme A	XM_011750
Empty	EMPTY
heparan sulfate (glucosamine) 3-O-sulfot	NM_005114
Homo sapiens PAC clone RP5-978E18 from 7	AV757511
ESTs	AI080133
mesenchyme homeo box 1	XM_008351
galactosidase, beta 1	AL545241
Z-band alternatively spliced PDZ-motif	F20509
immunoglobulin superfamily, member 3	AI968231
receptor tyrosine kinase-like orphan rec	AI638570
KDEL (Lys-Asp-Glu-Leu) endoplasmic retic	AL571182
triggering receptor expressed on myeloid	AF213457
Junk, low PCR	EMPTY
ESTs	AI141757
interleukin 1 receptor-like 2	NM_003854
carbonic anhydrase VIII	NM_004056
zinc finger protein	XM_009369
fibulin 2	NM_001998
KIAA0261 protein	AW402845
Empty	EMPTY
myosin-binding protein C, cardiac	U91629
ESTs	AW499970
KIAA1609 protein	AL137316
lymphocyte-specific protein tyrosine kin	XM_001772
integrin, beta 7	AL581999
ESTs, Weakly similar to G01789 citrate t	AA768730
hypothetical protein FLJ23467	NM_024575
moesin	Z98946
protein C (inactivator of coagulation fa	M11228
ESTs	AI866555

early growth response 3	X63741
ESTs, Weakly similar to ALU1_HUMAN ALU S	AI825027
Human DNA sequence from clone 413H6 on c	AA426092
aquaporin 5	BF341066
chloride channel, calcium activated, fam	AI660957
modulator recognition factor I	AI962711
ubiquinol-cytochrome c reductase (6.4kD)	AI564479
E74-like factor 3 (ets domain transcript	AL555767
protein with polyglutamine repeat; calci	BE741277
ESTs, Moderately similar to ALU2_HUMAN A	AI620057
H.sapiens polyA site DNA	AW516955
Junk, low PCR	EMPTY
ESTs, Weakly similar to ALU1_HUMAN ALU S	AA258233
toll-like receptor 4	AF177765
Sjogren syndrome antigen B (autoantigen	BE856256
KIAA0344 gene product	NM_014823
Junk, low PCR	EMPTY
growth factor, erv1 (S. cerevisiae)-like	AI992142
hypothetical protein	AA662785
dipeptidylpeptidase VI	XM_004709
butyrate response factor 2 (EGF-response	AW262861
SET domain and mariner transposase fusio	AL515729
CD2 antigen (p50), sheep red blood cell	NM_001767
phosphatidylinositol-4-phosphate 5-kinas	NM_003557
FK506 binding protein 12-rapamycin assoc	NM_004958
No ID Incyte EST	EMPTY
activating transcription factor 4 (tax-r	NM_001675
ESTs	AA574098
Junk, low PCR	EMPTY
centrosomal protein 2	AU143768
claudin 1	AA169619
SH3-domain GRB2-like 3	H20194
protein kinase, lysine deficient 1	AA306663
coxsackie virus and adenovirus receptor	AI557255
Homo sapiens cDNA FLJ11632 fis, clone HE	AL043915
hypothetical protein similar to mouse Dn	AI337322
ESTs	AA767669
serine (or cysteine) proteinase inhibito	AI608986
dynamin 1	AW206374
KIAA0363 protein	AB002361
Junk, low PCR	EMPTY
glycyl-tRNA synthetase	AC004976
serine/threonine kinase 25 (Ste20, yeast	BE278206
fibromodulin	AW073728
RAB9, member RAS oncogene family	BG535930
ESTs	AA505093
Junk, low PCR	EMPTY
GDNF family receptor alpha 2	U97145
FGFR1 oncogene partner	AA292127
Junk, low PCR	EMPTY
UDP-N-acetyl-alpha-D-galactosamine:polyp	XM_002282

adaptor-related protein complex 2, beta	BE890196
ESTs	BF511290
ESTs	AA694343
ESTs	AA258217
KIAA0616 protein	AB014516
hippocalcin	BC001777
ATPase, Na+/K+ transporting, alpha 3 pol	X12910
transducin-like enhancer of split 4, hom	AB033087
triosephosphate isomerase 1	BG337175
coagulation factor II (thrombin)	AU121309
murine leukemia viral (bmi-1) oncogene h	AU133654
thrombomodulin	AL049651
SnRNP assembly defective 1 homolog	AA758748
ATPase, H+ transporting, lysosomal (vacu	AI338777
myosin-binding protein H	NM_004997
ESTs	AA872860
Junk, low PCR	EMPTY
Homo sapiens clone B18 unknown mRNA	AI338760
putative G protein coupled receptor	NM_007223
ESTs	BF108664
Homo sapiens mRNA; cDNA DKFZp434D0215 (f	AA019158
KIAA0805 protein	AK027062
breast cell glutaminase	AF038170
RAN binding protein 16	BG179617
apolipoprotein L, 3	AY014906
potassium channel, subfamily K, member 3	BF970075
progesterone receptor	NM_000926
actin filament associated protein	XM_011188
motilin	XM_004152
phosphate cytidylyltransferase 2, ethano	AL044146
ESTs	AW004683
frizzled-related protein	AL546699
U5 snRNP-specific protein (220 kD), orth	AF092565
trichorhinophalangeal syndrome I	AA579370
carcinoembryonic antigen-related cell ad	NM_001816
major histocompatibility complex, class	BF795929
necdin (mouse) homolog	XM_007686
ESTs	AI690629
hypothetical protein MGC2495	AA972572
ESTs	AW514238
hypothetical protein	AI202106
truncated calcium binding protein	BG164025
mannose-binding lectin (protein A) 1, ps	AA699400
Junk, low PCR	EMPTY
UDP-galactose transporter related	AW192554
gastrin-releasing peptide receptor	NM_005314
thymidylate synthetase	AI174883
centromere protein A (17kD)	AL555786
hypothetical protein FLJ13163	AW117454
N-acetylglucosaminidase, alpha- (Sanfili	AW271546
KIAA0418 gene product	XM_005940

G protein-coupled receptor 86	AA142914
PDZ domain protein (Drosophila inaD-like	AB044807
interleukin 1, alpha	NM_000575
caldesmon 1	BG163850
hypothetical protein FLJ10116	AI089810
Homo sapiens mRNA; cDNA DKFZp564B076 (fr	AA457718
hypothetical protein FLJ10461	AL137710
adenylate cyclase 7	AL580276
carbohydrate (chondroitin 6/keratan) sul	BF477523
ESTs	BG110974
nuclear transcription factor Y, beta	AW953596
LIS1-interacting protein NUDEL; endoolig	AL571444
a disintegrin and metalloproteinase doma	NM_021612
Human uroporphyrinogen III synthase mRNA	EMPTY
U3 snoRNP-associated 55-kDa protein	XM_003209
Junk, low PCR	EMPTY
Fc fragment of IgG, high affinity Ia, re	X14356
tumor necrosis factor (TNF superfamily,	NM_000594
proteasome (prosome, macropain) 26S subu	BG528051
Human clone 23695 mRNA sequence	U79289
dual specificity phosphatase 2	NM_004418
hypothetical protein FLJ20535	AU159187
ESTs	N56841
hypothetical protein FLJ10462	AL136843
plectin 1, intermediate filament binding	AI076717
ESTs	AI633559
Junk, low PCR	EMPTY
ESTs	AI828498
hypothetical protein FLJ23306	AI088306
heterogeneous nuclear ribonucleoprotein	AW341514
Fanconi anemia, complementation group A	BG036552
Homo sapiens adenylyl cyclase-associated	EMPTY
vasoactive intestinal peptide	XM_004381
B/K protein	BC004518
transmembrane 4 superfamily member 6	AI721194
fibrinogen-like 2	AI796353
ESTs	AI123326
KIAA0215 gene product	NM_014735
fibroblast growth factor receptor 4	Y13901
ESTs	AA707217
ESTs	AI374654
GTPase activating protein	BG393566
Homo sapiens cDNA FLJ12935 fis, clone NT	AK022997
hypothetical protein FLJ12116	AU147230
NADH dehydrogenase (ubiquinone) 1 beta s	AV724440
regulator of differentiation (in S. pomb	NM_005156
PTD008 protein	BG178791
KIAA0712 gene product	AK023715
dual specificity phosphatase 3 (vaccinia	AL555009
Homo sapiens (clone 13a) deoxyhypusine s	EMPTY
COP9 (constitutive photomorphogenic, Ara	AA287305

actin, alpha 2, smooth muscle, aorta	BF681347
ZW10 interactor anti-sense	AW409765
KDEL (Lys-Asp-Glu-Leu) endoplasmic retic	AU132234
Junk, low PCR	EMPTY
SWI/SNF related, matrix associated, acti	BF111200
homeo box B5	AI689349
Junk, low PCR	EMPTY
DNAX-activation protein 10	AF172929
crooked neck protein (crn)	AL035454
Homo sapiens cDNA FLJ12425 fis, clone MA	BF439782
Homo sapiens cDNA FLJ10023 fis, clone HE	AI361569
KIAA0916 protein	AB020723
catenin (cadherin-associated protein), a	AL535157
dystrobrevin, beta	AL562339
ryanodine receptor 3	N45123
nuclear receptor subfamily 1, group D, m	D16815
Human chromatin assembly factor-I p60 su	EMPTY
transmembrane 9 superfamily member 2	AU131084
phosphoserine phosphatase-like	NM_003832
ubiquitin specific protease 14 (tRNA-gua	AL546236
coagulation factor VIII, procoagulant co	AA808551
hypothetical protein FLJ20514	AA443274
hepatoma-derived growth factor (high-mob	BF969749
KIAA0094 protein	BE902721
regulator of G-protein signalling 9	AA915931
KIAA1554 protein	AI673099
Homo sapiens cDNA FLJ13432 fis, clone PL	AK023494
opiate receptor-like 1	AW451157
KIAA0996 protein	NM_014934
C-reactive protein, pentraxin-related	AW963048
major histocompatibility complex, class	M24364
Rab geranylgeranyltransferase, beta subu	BG532217
Homo sapiens clone 24411 mRNA sequence	AA952981
Junk, low PCR	EMPTY
Human cytoplasmic chaperonin hTRiC5 mRNA	EMPTY
PDZ domain containing 1	BG398665
nuclear receptor subfamily 1, group H, m	NM_005123
membrane component, chromosome 17, surfa	D30756
microtubule-associated protein 7	BE545516
ESTs	AI360052
TEA domain family member 1 (SV40 transcr	NM_021961
receptor (calcitonin) activity modifying	XM_004893
EST	N54321
Homo sapiens cDNA: FLJ21836 fis, clone H	H78177
ESTs, Weakly similar to cDNA EST EMBL:Z1	BE813896
candidate mediator of the p53-dependent	T09994
ESTs	AA496962
C-reactive protein, pentraxin-related	AV654338
glycoprotein, synaptic 2	BG282184
pituitary tumor-transforming 1 interacti	BE898391
Homo sapiens clone 23927 mRNA sequence	C06361

Junk, low PCR	EMPTY
Human mRNA for cytochrome c oxidase subu	EMPTY
elastase 2, neutrophil	NM_001972
transglutaminase 3 (E polypeptide, prote	AL031678
stromal cell-derived factor 1	L36033
epidermal growth factor receptor (avian	NM_005228
hypothetical protein FLJ21877	AI240523
deiodinase, iodothyronine, type III	XM_007250
Junk, low PCR	EMPTY
ESTs	AI872281
ESTs	AW291290
GLE1 (yeast homolog)-like, RNA export me	AW515128
EST	AA665085
ESTs	AW970246
transcription elongation factor A (SII),	NM_003195
phosphodiesterase 1A, calmodulin-depende	AU120157
CD27-binding (Siva) protein	AI267883
mitogen-activated protein kinase kinase	AA737502
ferredoxin reductase	NM_004110
Arabadopsis2-125	EMPTY
interferon-induced protein 75, 52kD	NM_004510
eukaryotic translation initiation factor	BE792129
ESTs	AI767324
testis specific leucine rich repeat prot	AL041128
Junk, low PCR	EMPTY
Junk, low PCR	EMPTY
CD38 antigen (p45)	D84276
ESTs	AA731688
Junk, low PCR	EMPTY
ESTs	AA702016
CGI-82 protein	AI971404
KIAA0751 gene product	AA436640
COX11 (yeast) homolog, cytochrome c oxid	AI921588
tubulin, beta polypeptide	BE727082
ESTs	AA599107
BAI1-associated protein 2	BF568661
endothelin type b receptor-like protein	NM_004767
Arabadopsis4-125	EMPTY
cytochrome P450, subfamily IIC (mephenyt	AV646130
guanine nucleotide binding protein (G pr	NM_006572
ESTs	AA824285
putative protein	AW665850
Homo sapiens TXK tyrosine kinase (TXK),	XM_003333
zinc finger protein 135 (clone pHZ-17)	XM_008835
ribosomal protein L37	AW969881
guanine nucleotide binding protein (G pr	AI955355
Junk, low PCR	EMPTY
Homo sapiens zinc finger protein mRNA, c	AF334161
SMC (mouse) homolog, X chromosome	AA488934
HMP19 protein	BC002619
ribosomal protein S3	AI499808

semenogelin I	NM_003007
doublecortex; lissencephaly, X-linked (d	AK002120
hypothetical protein FLJ10374	AI672998
Niemann-Pick disease, type C1	AI001914
Arabadopsis6-125	EMPTY
Homo sapiens, clone MGC:12401, mRNA, com	M20642
dendritic cell protein	AI184292
ESTs	AA844729
proprotein convertase subtilisin/kexin t	NM_000439
Junk, low PCR	EMPTY
protein tyrosine phosphatase, receptor t	BG397566
RNA helicase family	AY013288
ESTs	AI923137
cerebellin 1 precursor	NM_004352
ESTs, Weakly similar to T15138 hypotheti	BG260450
Homo sapiens MAIL mRNA, complete cds	AI826047
ESTs, Weakly similar to SYM_HUMAN METHIO	AA058944
KIAA0738 gene product	AW962619
nuclear receptor subfamily 1, group I, m	AF061056
TATA box binding protein (TBP)-associate	AI056692
corneodesmosin	L20815
ribosomal protein S16	NM_001020
Empty	EMPTY
peanut (Drosophila)-like 1	U69565
CD36 antigen (collagen type I receptor,	NM_005506
ESTs, Weakly similar to ALU1_HUMAN ALU S	AI760013
keratin, hair, basic, 1	BE785699
steroid-5-alpha-reductase, alpha polypep	NM_000348
Junk, low PCR	EMPTY
tyrosine aminotransferase	NM_000353
lymphocyte antigen 9	AF244129
biliverdin reductase B (flavin reductase	BG340491
ESTs, Highly similar to MGR7_HUMAN METAB	AA018686
ESTs, Weakly similar to unknown [S.cerev	AI878836
ESTs	AA989401
Homo sapiens clone 24527 mRNA sequence	AF070580
putative purinergic receptor	BF795250
RAS protein activator like 1 (GAP1 like)	NM_004658
carcinoembryonic antigen-related cell ad	C06042
bridging integrator 1	BF529230
Empty	EMPTY
ubiquitin specific protease 8	NM_005154
epidermal growth factor receptor pathway	AI692447
ESTs	BE671119
protein kinase C, delta	XM_003106
peroxisome proliferative activated recep	BC000052
sterol O-acyltransferase (acyl-Coenzyme	NM_003101
Human clone 23933 mRNA sequence	AV756187
oligodendrocyte myelin glycoprotein	M63623
solute carrier family 2 (facilitated glu	NM_000340
ESTs	AA975173

DKFZP586G1517 protein	H80062
heterogeneous nuclear ribonucleoprotein	AU144878
core-binding factor, runt domain, alpha	AI584154
sin3-associated polypeptide, 30kD	AW236579
kinesin family member 3C	AI806659
hypothetical protein FLJ22004	N25427
prefoldin 1	AL521761
Empty	EMPTY
estrogen-related receptor alpha	L38487
glia maturation factor, gamma	AA279067
DKFZP564B0769 protein	AW450697
hydroxysteroid (17-beta) dehydrogenase 3	AA442370
Junk, low PCR	EMPTY
Junk, low PCR	EMPTY
ATP citrate lyase	BE890755
Junk, low PCR	EMPTY
hypothetical protein from clone 24774	AI819077
ESTs	BE047235
hypothetical protein FLJ20277	AI584095
ESTs, Weakly similar to AF174605 1 F-box	AI004646
SRY (sex determining region Y)-box 5	NM_006940
transcription elongation factor A (SII),	N41981
RAB30, member RAS oncogene family	AA455321
ESTs	AW960145
ribosomal protein S3A	BG488776
ESTs, Weakly similar to ALU8_HUMAN ALU S	BF509171
ESTs, Moderately similar to ALU8_HUMAN A	AI668709
KIAA0438 gene product	BF529933
mevalonate kinase (mevalonic aciduria)	BG474232
Ras-related associated with diabetes	AI186786
ESTs	AI244340
Junk, low PCR	EMPTY
oxytocin, prepro- (neurophysin I)	AW242780
peroxiredoxin 1	BF978853
pleckstrin	NM_002664
myosin-binding protein C, fast-type	NM_004533
Junk, low PCR	EMPTY
melanoma-associated antigen recognised b	BG491880
vesicle-associated membrane protein 3 (c	BE272994
protein tyrosine phosphatase, receptor t	U40317
Junk, low PCR	EMPTY
linker for activation of T cells	AA309971
putative protein similar to nessy (Droso	AL532090
ESTs, Weakly similar to T08680 hypotheti	N45121
ESTs	AW769896
polycystic kidney disease 1 (autosomal d	NM_000296
natriuretic peptide precursor A	M30262
deoxyguanosine kinase	U41668
degenerative spermatocyte (homolog Droso	AA039929
Junk, low PCR	EMPTY
REMOVED_FROM_DATABASE	EMPTY

Junk, low PCR	EMPTY
pyruvate dehydrogenase kinase, isoenzyme	NM_002612
MyoD family inhibitor	NM_005586
IGF-II mRNA-binding protein 3	U76705
alpha-actinin-2-associated LIM protein	BE857659
claudin 4	AL572879
ESTs, Weakly similar to ALU7_HUMAN ALU S	AW008049
E74-like factor 5 (ets domain transcript	XM_006203
alpha-2-HS-glycoprotein	AI192852
chromosome 11 open reading frame 8	AI951765
hypothetical protein MGC5499	BE883319
ESTs	AW954306
myeloid cell nuclear differentiation ant	NM_002432
PBX/knotted 1 homeobox 1	AL522004
guanylate cyclase activator 1B (retina)	NM_002098
ESTs	AA883715
ESTs, Highly similar to SMHU1B metalloth	R99207
Junk, low PCR	EMPTY
inter-alpha (globulin) inhibitor H4 (pla	XM_003219
Junk, low PCR	EMPTY
Junk, low PCR	EMPTY
butyrophilin, subfamily 2, member A1	NM_007049
Ste20-related serine/threonine kinase	BF511206
mitochondrial ribosomal protein L3	BG034840
potassium voltage-gated channel, shaker-	T28887
amine oxidase, copper containing 3 (vasc	AF067406
tetratricopeptide repeat domain 4	AI567946
plexin A2	AI924016
tubby super-family protein	AF288480
ESTs	AI080700
nudix (nucleoside diphosphate linked moi	AW173059
lectin, galactoside-binding, soluble, 3	L13210
trefoil factor 1 (breast cancer, estroge	AB038162
Junk, low PCR	EMPTY
tumor necrosis factor (ligand) superfami	XM_005349
small inducible cytokine subfamily A (Cy	XM_008452
ARP1 (actin-related protein 1, yeast) ho	BC004374
pyridoxal (pyridoxine, vitamin B6) kinas	AL519498
Human clone 23589 mRNA sequence	U79297
protein phosphatase 1, regulatory (inhib	BE872961
prostate differentiation factor	AU123196
melanoma adhesion molecule	BE786166
origin recognition complex, subunit 4 (y	AI923588
nuclear receptor subfamily 1, group H, m	BE878950
TBP-interacting protein	BF055335
solute carrier family 20 (phosphate tran	NM_006749
ESTs	AI685487
ESTs	AI335850
TGFB1-induced anti-apoptotic factor 1	AI912485
myosin, heavy polypeptide 3, skeletal mu	NM_002470
zinc finger protein 146	BF447806

ESTs, Weakly similar to ALU7_HUMAN ALU S	AI075172
Junk, low PCR	EMPTY
intersectin 1 (SH3 domain protein)	AA582575
KIAA0591 protein	BF510716
transcription factor (p38 interacting pr	Z44096
Junk, low PCR	EMPTY
solute carrier family 25 (mitochondrial	AL527028
SRY (sex determining region Y)-box 10	BE677542
fucosyltransferase 2 (secretor status in	NM_000511
membrane-bound transcription factor prot	AI656900
CREB binding protein (Rubinstein-Taybi s	NM_004380
telomeric repeat binding factor (NIMA-in	AA467901
KIAA0544 protein	BF801696
ESTs	AW300082
KIAA0630 protein	AA885443
SHC (Src homology 2 domain-containing) t	BG108303
Junk, low PCR	EMPTY
inositol 1,3,4-triphosphate 5/6 kinase	BF338323
ESTs	AI803168
GATA-binding protein 1 (globin transcrip	NM_002049
KIAA0233 gene product	NM_014745
early endosome antigen 1, 162kD	NM_003566
heterogeneous nuclear ribonucleoprotein	AI767615
pre-B-cell leukemia transcription factor	NM_006195
Alu-binding protein with zinc finger dom	NM_014274
collagen, type XVI, alpha 1	NM_001856
plakophilin 4	BE644965
Junk, low PCR	EMPTY
DKFZp434J1813 protein	BG250586
Junk, low PCR	EMPTY
meningioma expressed antigen 5 (hyaluron	AA835470
H.sapiens mRNA for cyclin H assembly fac	EMPTY
KIAA1209 protein	N70181
ESTs	AA131854
kinesin family member 3C	AF035621
U2 small nuclear ribonucleoprotein auxil	AA936430
ESTs	BE466291
Junk, low PCR	EMPTY
cell division cycle 25C	AL545014
ESTs	AA465350
hydroxyacyl glutathione hydrolase	XM_007922
hypothetical protein	AF151076
spectrin, alpha, erythrocytic 1 (ellipto	AA703344
nectin 3; DKFZP566B0846 protein	BG026179
KIAA0747 protein	BE514479
ATP synthase, H+ transporting, mitochond	AV714814
pronapsin A	NM_004851
secretory carrier membrane protein 2	AV734956
complement component (3d/Epstein Barr vi	J03565
Human liver glutamate dehydrogenase mRNA	EMPTY
ESTs	BE550363

empty spiracles (Drosophila) homolog 2	AI242313
polymerase (RNA) II (DNA directed) polyp	F24474
coatomer protein complex, subunit epsilo	AA410871
glucose phosphate isomerase	AI682204
golgi-specific brefeldin A resistance fa	AK025330
cytochrome c oxidase subunit Va	BE538296
ESTs	H13112
procollagen-lysine, 2-oxoglutarate 5-dio	AL544817
ESTs	AA102395
protein phosphatase 1A (formerly 2C), ma	NM_021003
ESTs	BF590379
four and a half LIM domains 2	AW664260
Kruppel-type zinc finger (C2H2)	AK023025
protein arginine N-methyltransferase 3(h	AF059531
paraneoplastic antigen	L02867
KIAA0547 gene product	NM_014793
Human mitochondrial ADP/ADT translocator	EMPTY
ESTs	AA603466
polyamine-modulated factor 1	AA594517
brain-specific angiogenesis inhibitor 3	AI701805
HIV-1 rev binding protein 2	AW974533
Apg12 (autophagy 12, S. cerevisiae)-like	AV696098
v-Ha-ras Harvey rat sarcoma viral oncoge	BG419155
ribosomal protein S4, X-linked	BG503207
Homo sapiens cDNA: FLJ23155 fis, clone L	AW629859
chimerin (chimaerin) 1	BF940950
angiotensin receptor-like 2	AW410962
cholinergic receptor, nicotinic, alpha p	BC000513
ESTs, Weakly similar to leucine-rich gli	AI366706
tetraspan 3	BC004280
ESTs, Weakly similar to ALU8_HUMAN ALU S	AI744345
regulator of G-protein signalling 1	S59049
downregulated in ovarian cancer 1	NM_014890
Mouse Mammary Turmor Virus Receptor homo	AI494494
Human malate dehydrogenase (MDHA) mRNA,	EMPTY
hypothetical protein FLJ13117	AK023179
ESTs	AA927436
cytochrome c oxidase subunit VIc	AI568937
ESTs	AL535154
ESTs	AW241298
glucosaminyl (N-acetyl) transferase 1, c	AL555400
FLN29 gene product	AU131366
Homo sapiens, Similar to ribosomal prote	AL390152
KIAA0128 protein; septin 2	AW402614
tubulin-specific chaperone d	AL133562
KIAA0298 gene product	AB002296
hypothetical protein FLJ23231	AA640102
thyroid autoantigen 70kD (Ku antigen)	AU153096
glutathione-S-transferase like; glutathi	BG529826
ribosomal protein S6 kinase, 70kD, polyp	XM_008307
neural precursor cell expressed, develop	BE783166

centromere protein E (312kD)	NM_001813
Unknown - Human Control Plate Well H9	EMPTY
CGI-18 protein	AW954076
Homo sapiens cDNA: FLJ21513 fis, clone C	AA913401
Junk, low PCR	EMPTY
KIAA0483 protein	AI690717
ESTs	BF977427
tec protein tyrosine kinase	NM_003215
homeo box B2	BF432153
oxidoreductase UCPA	AI141369
cleavage and polyadenylation specific fa	NM_013291
ESTs, Weakly similar to weak similarity	AW001427
SWI/SNF related, matrix associated, acti	X72889
KIAA0788 protein	AA826397
ubiquinol-cytochrome c reductase core pr	AU148041
Junk, low PCR	EMPTY
syntaxin binding protein 3	NM_007269
S-adenosylmethionine decarboxylase 1	AI042585
mitogen-activated protein kinase kinase	NM_004721
Unknown - Human Control Plate Well H12	EMPTY
ESTs	AI990892
TRIAD3 protein	BF515713
MAD (mothers against decapentaplegic, Dr	NM_005900
beta-1,3-glucuronyltransferase 1 (glucur	BE550759
CD209 antigen-like	AA448002
caspase 1, apoptosis-related cysteine pr	NM_001223
Junk, low PCR	EMPTY
ESTs	D20171
thiosulfate sulfurtransferase (rhodanese	AA483285
transcriptional co-activator with PDZ-bi	AL050107
solute carrier family 18 (vesicular mono	NM_003053
ESTs	AA203426
catenin (cadherin-associated protein), a	BF793401
serine (or cysteine) proteinase inhibito	L13470
haptoglobin-related protein	BG545187
sorbitol dehydrogenase	AU141255
KIAA0867 protein	AW513575
Empty	EMPTY
CGI-19 protein	AL355815
CGI-47 protein	AV703724
KIAA0082 protein	BF725343
transmembrane 4 superfamily member (tetr	AW248885
hypothetical protein FLJ20185	AL355192
protein kinase, Y-linked	BG506561
Rho GTPase activating protein 4	Z68128
Homo sapiens mRNA; cDNA DKFZp667O2416 (f	AI092632
hypothetical protein FLJ11294	AA773744
REV1 (yeast homolog)- like	AW614178
transcription factor CA150	NM_006706
ATPase, Ca++ transporting, type 2C, memb	N51919
Homo sapiens clone 23736 mRNA sequence	AF007153

NADH dehydrogenase (ubiquinone) Fe-S pro	NM_004553
heparan sulfate proteoglycan 2 (perlecan	NM_005529
epithelial protein up-regulated in carci	BF689053
wee1+ (S. pombe) homolog	AU143572
Empty	EMPTY
Junk, low PCR	EMPTY
spinal cord-derived growth factor-B	BE302139
putative human HLA class II associated p	AY007110
amino acid transporter system A1	BF348345
zinc finger protein 183 (RING finger, C3	AI283184
small inducible cytokine subfamily A (Cy	AI707984
myosin, heavy polypeptide 8, skeletal mu	XM_008442
Homo sapiens, Similar to single-stranded	BC000274
Junk, low PCR	EMPTY
suppressor of K+ transport defect 1	AA653471
26S proteasome-associated pad1 homolog	BE886981
ESTs	AI912373
ribonuclease, RNase A family, k6	AA834541
casein, alpha	NM_001890
cofactor required for Sp1 transcriptiona	AA737180
T-cell leukemia/lymphoma 1A	BG397961
a disintegrin and metalloproteinase doma	NM_003816
Empty	EMPTY
ghrelin precursor	XM_003163
Homo sapiens mRNA; cDNA DKFZp434D1428 (f	W68642
myosin phosphatase, target subunit 2	AW591567
hypothetical protein FLJ20277	BF589570
TRAM-like protein	NM_012288
Neuro-d4 (rat) homolog	BF970565
Junk, low PCR	EMPTY
ESTs	N99725
Homo sapiens, Similar to RIKEN cDNA 2810	AI022747
heme-regulated initiation factor 2-alpha	BE410732
signal recognition particle 14kD (homolo	AW078956
KIAA0993 protein	AF131845
HLA class II region expressed gene KE2	BE047705
bromodomain adjacent to zinc finger doma	AW853012
phosphotidylinositol transfer protein, b	AL035843
era (E. coli G-protein homolog)-like 1	BE871890
Junk, low PCR	EMPTY
Empty	EMPTY
ESTs	AI816047
DnaJ (Hsp40) homolog, subfamily B, membe	AU153895
TAR (HIV) RNA-binding protein 1	H99890
Homo sapiens mRNA; cDNA DKFZp762M127 (fr	BE047367
tenascin XA	XM_004201
thyroid stimulating hormone receptor	AF035261
sorting nexin 15	AL514784
Homo sapiens mRNA; cDNA DKFZp761G1111 (f	AA811893
KIAA0456 protein	AI091943
ESTs, Moderately similar to AF10_HUMAN A	AW573051

Junk, low PCR	EMPTY
hypothetical protein FLJ11773	BF594230
interleukin enhancer binding factor 2, 4	BE614422
phosphodiesterase 6B, cGMP-specific, rod	NM_000283
KIAA0485 protein	AB007954
immunoglobulin (CD79A) binding protein 1	AA700637
transforming growth factor, beta recepto	NM_003242
Empty	EMPTY
ESTs	AA464273
hypothetical protein FLJ10101	BF108684
cadherin 11, type 2, OB-cadherin (osteob	NM_001797
KIAA0648 protein	BE219268
Homo sapiens, clone IMAGE:3357927, mRNA,	AA724155
adenylyl cyclase-associated protein 2	AU123318
DKFZP434J046 protein	AL133651
membrane component, chromosome 11, surfa	AW295041
sodium channel, voltage-gated, type VI,	NM_002976
cleavage and polyadenylation specific fa	AK001627
thymidine kinase 2, mitochondrial	AK026021
Homo sapiens mRNA for FLJ00029 protein,	N25077
tetratricopeptide repeat domain 1	AW954147
myotubularin related protein 7	AF073482
keratin 8	AU122202
actin related protein 2/3 complex, subun	AL560125
pre-alpha (globulin) inhibitor, H3 polyp	XM_003218
Empty	EMPTY
hypothetical protein FLJ20813	AA913500
KIAA0766 gene product	AA243141
KIAA0377 gene product	BE903417
ESTs	AL521141
Fc fragment of IgE, high affinity I, rec	AV715358
protein tyrosine phosphatase, receptor t	NM_002837
KIAA0420 gene product	AB007880
hypothetical protein FLJ21212	AA983811
fibrinogen-like 1	XM_005305
ESTs, Weakly similar to LIV-1 protein [H	AI274179
LIM domain only 4	NM_006769
ethanolamine kinase	AI357819
polymyositis/scleroderma autoantigen 1 (	AL527487
ESTs, Weakly similar to ZN43_HUMAN ZINC	AW950447
ESTs, Weakly similar to S47072 finger pr	AI700612
KIAA0752 protein	AB018295
KIAA0761 protein	AA406603
thyroid peroxidase	J02970
chloride intracellular channel 4	AI381979
Junk, low PCR	EMPTY
Junk, low PCR	EMPTY
replication protein A1 (70kD)	AU124434
collagen, type XVIII, alpha 1	AL163302
neuroblastoma RAS viral (v-ras) oncogene	XM_001317
interleukin 6 signal transducer (gp130,	NM_002184

myosin light chain 2a	W17098
JM1 protein	XM_010199
Homo sapiens OSBP-related protein 6 mRNA	AW072611
ESTs	R92545
Junk, low PCR	EMPTY
H2A histone family, member N	BG109927
CHK1 (checkpoint, S.pombe) homolog	XM_006254
Homo sapiens TTF-I interacting peptide 2	AF000560
hypothetical protein F23149_1	BE562607
Junk, low PCR	EMPTY
dihydrolipoamide dehydrogenase (E3 compo	BG429134
ESTs	AI703426
serine palmitoyltransferase, long chain	AF111168
ESTs	W07758
KIAA0171 gene product	NM_014666
EGF-containing fibulin-like extracellula	NM_018894
phospholipase A2, group IB (pancreas)	NM_000928
carbohydrate (keratan sulfate Gal-6) sul	BE503729
neural cell adhesion molecule 1	NM_000615
synovial sarcoma, translocated to X chro	NM_005637
ESTs	AI025482
ESTs	AI633250
No ID Incyte EST	EMPTY
potassium voltage-gated channel, Shab-re	AF338730
synaptojanin 1	BF677649
CDC23 (cell division cycle 23, yeast, ho	NM_004661
KIAA0731 protein	AL133034
non-POU-domain-containing, octamer-bindi	BE384419
arylacetamide deacetylase (esterase)	BG542396
ESTs	AI023436
regulatory factor X-associated ankyrin-c	AF077196
Junk, low PCR	EMPTY
KIAA0027 protein	AW044650
solute carrier family 11 (proton-coupled	AL534676
neuregulin 1	NM_013957
dishevelled 3 (homologous to Drosophila	BF527005
KIAA0576 protein	AK000983
sodium bicarbonate transporter 4	NM_021196
hypothetical protein FLJ10390	AI748813
ESTs	AA747804
glucuronidase, beta	BF032215
troponin I, skeletal, fast	F22813
ESTs	AI676236
glutathione peroxidase 4 (phospholipid h	NM_002085
KIAA0662 gene product	BG108175
centromere protein B (80kD)	X55039
interleukin 10 receptor, alpha	NM_001558
E2F transcription factor 6	NM_001952
KIAA0103 gene product	AA447703
EST	AA811728
transducin-like enhancer of split 3, hom	XM_007677

protein tyrosine phosphatase, non-recept	AF178946
leukocyte-associated Ig-like receptor 1	AU122160
zinc finger protein 238	AJ223321
mitogen-activated protein kinase 8 inter	BE797338
Incyte EST	EMPTY
hypothetical protein FLJ20446	W80653
transporter-like protein	AI479682
nucleoporin 155kD	AW673911
ribosomal protein L9	BE270861
KIAA0627 protein; Drosophila	AI583424
tumor necrosis factor (ligand) superfami	AF055872
XPA binding protein 1; putative ATP(GTP)	AI291094
matrix metalloproteinase 9 (gelatinase B	NM_004994
signal transducer and activator of trans	NM_003153
KIAA0280 protein	D87470
MHC class I polypeptide-related sequence	BE737147
ESTs	AW020808
KIAA0711 gene product	AL524701
Junk, low PCR	EMPTY
G protein-coupled receptor, family C, gr	AC002550
lethal giant larvae (Drosophila) homolog	NM_004524
KIAA1128 protein	U90912
calcineurin-binding protein calsarcin-1	AW592020
ESTs	AI057634
ESTs	AA628481
H2B histone family, member B	BF509675
immunoglobulin lambda joining 3	BF338816
peptidylglycine alpha-amidating monooxyg	AU120984
cholinergic receptor, nicotinic, beta po	AW157173
SMC4 (structural maintenance of chromoso	BF239180
serine (or cysteine) proteinase inhibito	BE883604
thyroglobulin	AU141853
pyruvate dehydrogenase kinase, isoenzyme	NM_005391
ficolin (collagen/fibrinogen domain-cont	AL583593
KIAA1557 protein	AA948090
Junk, low PCR	EMPTY
chondroitin sulfate proteoglycan 2 (vers	AU118073
collagen, type IV, alpha 6	U04845
mitogen inducible 2	Z24725
X-ray repair complementing defective rep	Y08837
KIAA0579 protein	AA523974
Snf2-related CBP activator protein	AI381586
ESTs	AA649871
polymerase (RNA) II (DNA directed) polyp	BG420417
neural polypyrimidine tract binding prot	BF843306
LIM domains containing 1	XM_003254
putative DNA binding protein	AL578391
ganglioside expression factor 2	H99630
Junk, low PCR	EMPTY
Human cytosolic aspartate aminotransfera	EMPTY
immunoglobulin heavy constant mu	AI634950

lacrimal proline rich protein	AW631140
ESTs, Moderately similar to ALU1_HUMAN A	N66117
Junk, low PCR	EMPTY
phosphatidylserine decarboxylase	AI342072
hypothetical protein	AI037850
glucosidase, alpha; acid (Pompe disease,	X55079
synaptojanin 2	BE348342
myotubularin related protein 6	AW205652
Junk, low PCR	EMPTY
U6 snRNA-associated Sm-like protein LSm7	AA121509
Homo sapiens mRNA; cDNA DKFZp434K087 (fr	AA948540
cAMP response element-binding protein CR	AW469766
src homology three (SH3) and cysteine ri	AU152575
proline arginine-rich end leucine-rich r	BE836107
enolase 1, (alpha)	BE621812
protein kinase, cAMP-dependent, catalyti	NM_002730
Human 90 kD heat shock protein gene, com	EMPTY
cystinosis, nephropathic	BG392867
POU domain, class 4, transcription facto	X71488
ESTs	AA928780
hypothetical protein DKFZp761N09121	AF038190
zinc finger protein 282	D30612
KIAA0823 protein	AI432936
high-mobility group (nonhistone chromoso	BE618313
Junk, low PCR	EMPTY
nuclear receptor subfamily 2, group C, m	U10990
major histocompatibility complex, class	BF732822
ESTs	AI240369
ESTs	AI679014
Junk, low PCR	EMPTY
Homo sapiens cDNA FLJ20586 fis, clone KA	AK000593
bladder cancer associated protein	BF568604
Junk, low PCR, low PCR	EMPTY
nucleolin	AU123684
Human liver-type 1-phosphofructokinase (	EMPTY
Sec23 (S. cerevisiae) homolog B	BC005032
integral membrane protein 2A	AI183750
serine (or cysteine) proteinase inhibito	BC002538
Junk, low PCR	EMPTY
hippocalcin-like 1	AA937144
Homo sapiens cDNA FLJ12292 fis, clone MA	AA813577
adducin 3 (gamma)	AL135243
KIAA0596 protein	AI800870
RAB11A, member RAS oncogene family	XM_007625
collagen, type XIII, alpha 1	AW105556
KIAA1085 protein	AW166110
Notch (Drosophila) homolog 2	AA830014
serine (or cysteine) proteinase inhibito	AA747840
excision repair cross-complementing rode	NM_000124
RAB27A, member RAS oncogene family	AL120794
Junk, low PCR, low PCR	EMPTY

Junk, low PCR	EMPTY
Human mRNA for heterogeneous nuclear rib	EMPTY
Human ECRP gene for eosinophil cationic	X55989
Junk, low PCR	EMPTY
ESTs, Weakly similar to T46471 hypotheti	BF062969
Junk, low PCR	EMPTY
interleukin enhancer binding factor 3, 9	AJ271746
hypothetical protein	AA837108
glucose regulated protein, 58kD	BE799456
heptacellular carcinoma novel gene-3 pro	NM_016651
ninjurin 1	BG530047
acyl-Coenzyme A dehydrogenase, long chai	NM_001608
ESTs, Highly similar to C34323 GTP-bindi	AI278619
cytidine monophosphate-N-acetylneuramini	BG231623
UDP glycosyltransferase 2 family, polype	AF135416
pancreatic lipase	AA363798
regulator of G-protein signalling 1	S59049
apolipoprotein D	NM_001647
mel transforming oncogene (derived from	BG327764
Human glyceraldehyde 3-phosphate dehydro	EMPTY
peptidylprolyl isomerase A (cyclophilin	AV713597
retinoic acid receptor responder (tazaro	AV702762
uncharacterized hypothalamus protein HT0	AI823779
methylene tetrahydrofolate dehydrogenase	AL560341
interleukin 1 receptor, type II	BG248550
hypothetical protein FLJ13187	AA886792
protein kinase C, beta 1	NM_002738
ESTs, Highly similar to ALU8_HUMAN ALU S	N93536
squamous cell carcinoma antigen recognis	BG491601
Homo sapiens, Similar to hypothetical pr	AL520990
Junk, low PCR	EMPTY
ESTs	AI423072
GRB2-associated binding protein 1	AK022142
titin-cap (telethonin)	F25003
aldehyde dehydrogenase 4 family, member	AL523157
Junk, low PCR	EMPTY
phorbol-12-myristate-13-acetate-induced	BG392214
Homo sapiens cadherin-13 mRNA, complete	EMPTY
retinoic acid receptor responder (tazaro	AI784017
KIAA0527 protein	AB011099
ESTs	AA102332
CD72 antigen	NM_001782
methionine-tRNA synthetase	AU131452
kelch (Drosophila)-like 2 (Mayven)	AI814691
integrin, alpha E (antigen CD103, human	AV762515
ESTs	AA551137
vesicle transport-related protein	AF319958
caveolin 3	AF043101
ELG protein	AJ277841
Junk, low PCR	EMPTY
glutathione S-transferase M2 (muscle)	AW239463

S100 calcium-binding protein A9 (calgran	BG331778
KIAA0251 protein	AA318390
Junk, low PCR	EMPTY
ADP-ribosyltransferase (NAD+; poly (ADP-	BE740909
Empty	EMPTY
cytochrome P450, subfamily I (aromatic c	NM_000499
cyclin-dependent kinase 9 (CDC2-related	NM_001261
oviductal glycoprotein 1, 120kD (mucin 9	XM_001932
KIAA0029 protein	AW973355
Junk, low PCR	EMPTY
hypothetical protein FLJ11939	NM_024679
PTK2 protein tyrosine kinase 2	AL119232
ESTs	AA477828
hypothetical protein F25965	BE504020
early growth response 4	NM_001965
ESTs	AI635003
KIAA1391 protein	R43918
dermatopontin	AL553464
beta-transducin repeat containing	AF129530
endothelial differentiation, G-protein-c	XM_009219
arylsulfatase E (chondrodysplasia puncta	NM_000047
developmentally regulated GTP-binding pr	BF149268
Empty	EMPTY
GTP binding protein 1	NM_004286
serologically defined colon cancer antig	NM_006643
T cell receptor delta locus	X06557
CDC28 protein kinase 2	NM_001827
erythrocyte membrane protein band 7.2 (s	BF966476
ESTs	N49233
cathepsin G	NM_001911
HT021	AI383067
solute carrier family 35 (UDP-galactose	BE885525
c-myc binding protein	AL561551
ESTs	AI609073
hypothetical protein FLJ21918	AI288958
synaptosomal-associated protein, 91 kDa	N62961
ATP/GTP-binding protein	AA188236
palmitoyl-protein thioesterase 2	BE790900
Junk, low PCR	EMPTY
natriuretic peptide receptor C/guanylate	BF224187
Empty	EMPTY
interleukin 13 receptor, alpha 2	R52795
NADH dehydrogenase (ubiquinone) 1 alpha	F20629
serine protease inhibitor, Kunitz type,	AL547950
apolipoprotein L	XM_009952
Junk, low PCR	EMPTY
Homo sapiens clone 24766 mRNA sequence	AI124007
Junk, low PCR	EMPTY
Homo sapiens brain my040 protein mRNA, c	AI971242
Homo sapiens, clone IMAGE:3954961, mRNA,	U62317
synaptojanin 2	AF039945

ESTs	AI367245
CGI-26 protein	AI707567
chromosome 6 open reading frame 32	AB002384
complement component 9	K02766
KIAA0756 protein	AB018299
Junk, low PCR	EMPTY
chloride channel 2	AL524236
Empty	EMPTY
Junk, low PCR	EMPTY
surfactant, pulmonary-associated protein	J02761
SWI/SNF related, matrix associated, acti	NM_003072
neogenin (chicken) homolog 1	XM_012444
zinc finger protein 165	NM_003447
KIAA1126 protein	AA769878
complement component 2	NM_000063
Junk, low PCR	EMPTY
peroxisome biogenesis factor 13	NM_002618
neuronal Shc adaptor homolog	AL360254
ESTs	AA937116
apolipoprotein M	AI858956
ubiquitin-conjugating enzyme E2L 3	NM_003347
dystrophia myotonica-containing WD repea	BF996358
KIAA0674 protein	AB014574
zinc finger protein 134 (clone pHZ-15)	NM_003435
KIAA0685 gene product	AI741556
Empty	EMPTY
phospholipase A2, group IVA (cytosolic,	AI627464
phosphatidylinositol glycan, class C	AI679299
major vault protein	AL540890
ornithine carbamoyltransferase	NM_000531
adaptor-related protein complex 3, beta	NM_004644
ESTs	AA354976
conserved helix-loop-helix ubiquitous ki	AF012890
ATPase, Class II, type 9B	AL096735
Homo sapiens mRNA from chromosome 5q31-3	AF010236
Ser-Thr protein kinase related to the my	NM_003607
Homo sapiens PAC clone RP4-651K2 from 7p	N62902
ancient conserved domain protein 4	AI082811
lung type-I cell membrane-associated gly	AA614141
chromosome 1 open reading frame 8	BG476131
protein phosphatase 1D magnesium-depende	NM_003620
Junk, low PCR	EMPTY
KIAA0779 protein	H80618
Empty	EMPTY
hypothetical protein LOC63931	BG254653
creatine kinase, mitochondrial 2 (sarcom	NM_001825
EH domain containing 1	AL523212
Junk, low PCR	EMPTY
KIAA0097 gene product	D43948
nucleoporin p54	AA399363
potassium inwardly-rectifying channel, s	NM_021012

ESTs	AI872254
glycerol-3-phosphate dehydrogenase 2 (mi	U36310
cut (Drosophila)-like 1 (CCAAT displacem	AL558580
Human DNA sequence from clone RP5-1046G1	AW134660
ESTs	AA484891
KIAA0701 protein	AI350647
low density lipoprotein receptor-related	BF061700
RAB6 interacting, kinesin-like (rabkines	AV714379
Junk, low PCR	EMPTY
WAS protein family, member 2	AI094497
ESTs	AI222435
Junk, low PCR	EMPTY
laminin, beta 1	AU119464
ribosomal protein L13a	BG505355
exonuclease NEF-sp	AI967994
ESTs	BE465454
neuropeptide Y	K01911
TNF receptor-associated factor 3	U19260
neuro-oncological ventral antigen 1	NM_002515
filamin B, beta (actin-binding protein-2	NM_001457
uracil-DNA glycosylase	NM_003362
major histocompatibility complex, class	BG176768
chondroitin sulfate proteoglycan 6 (bama	AI819641
deoxycytidine kinase	AA639428
unc5 (C.elegans homolog) c	NM_003728
Junk, low PCR	EMPTY
interleukin 1 receptor-like 1	AL553735
T-cell, immune regulator 1	NM_006053
hypothetical protein FLJ20254	BG029850
HSPC189 protein	AA886389
Homo sapiens mRNA for TL132	AA022783
Junk, low PCR	EMPTY
UDP-Gal:betaGlcNAc beta 1,4- galactosylt	AK001006
hypothetical protein	AV755380
chromosome 2 open reading frame 3	AC005034
secretory granule, neuroendocrine protei	BE047084
glutamate receptor, ionotropic, AMPA 1	NM_000827
SAC2 (suppressor of actin mutations 2, y	AL390171
arginine-rich, mutated in early stage tu	AA974308
ribosomal protein S29	AI133015
transcobalamin II; macrocytic anemia	L02648
N-acetylneuraminic acid phosphate syntha	BE614630
Dmx-like 1	NM_005509
interleukin 8	AV717082
KIAA1404 protein	AA046494
actin, beta	BG481840
ESTs	BE047628
Homo sapiens mRNA for HMG-box transcript	AI971964
Rho guanine nucleotide exchange factor (	NM_014629
transcription factor 7 (T-cell specific,	XM_003836
nucleosome assembly protein 1-like 3	AL120635

ESTs	AI355302
myeloperoxidase	X15377
FK506-binding protein 4 (59kD)	XM_007052
D-amino-acid oxidase	AI791352
protein phosphatase 2, regulatory subuni	AA234460
Cdc42 guanine exchange factor (GEF) 9	NM_015185
CD86 antigen (CD28 antigen ligand 2, B7-	F07984
osteomodulin	AI989443
sialyltransferase 4B (beta-galactosidase	NM_006927
acrosin	XM_010064
GRB2-related adaptor protein	AW951044
serine/threonine kinase 17b (apoptosis-i	AW172382
spondin 1, (f-spondin) extracellular mat	AA127466
calpain 7	AW471155
timeless (Drosophila) homolog	AU149253
RecQ protein-like (DNA helicase Q1-like)	AU119581
Junk, low PCR	EMPTY
DnaJ (Hsp40) homolog, subfamily C, membe	NM_003315
ESTs	AA666202
Junk, low PCR	EMPTY
dual specificity phosphatase 4	NM_001394
transgelin	BG110652
KIAA0284 protein	AK025023
fibrillin 1 (Marfan syndrome)	NM_000138
SFRS protein kinase 2	AW629710
branched chain keto acid dehydrogenase E	BE254024
acyl-Coenzyme A dehydrogenase, C-4 to C-	AA505399
KIAA0152 gene product	XM_012123
nuclear receptor subfamily 1, group H, m	BF792902
chemokine (C-C motif) receptor 3	NM_001837
vacuolar protein sorting 41 (yeast homol	XM_004830
ESTs	N48704
hypothetical protein FLJ10900	AI304874
hypothetical protein 384D8_6	U62317
embryonic ectoderm development	AI990456
RAB7, member RAS oncogene family-like 1	BE696116
ESTs	AI674937
SKI-like	NM_005414
argininosuccinate synthetase	BE393272
ZW10 (Drosophila) homolog, centromere/ki	AL528376
cathepsin C	AV717480
FERM, RhoGEF (ARHGEF) and pleckstrin dom	BF793662
Junk, low PCR	EMPTY
myosin, light polypeptide, regulatory, n	AA345289
eukaryotic translation initiation factor	BF683408
H.sapiens mRNA for 3'UTR of unknown prot	AW803141
KIAA0410 gene product	NM_014778
KIAA0225 protein	D86978
neural precursor cell expressed, develop	AV651189
EST	N52017
Homo sapiens cDNA FLJ11515 fis, clone HE	AA604935

Tat-interacting protein (30kD)	BC002439
apolipoprotein A-I	J00098
cyclin D3	AI803460
ESTs	AA169248
adenosine A1 receptor	NM_000674
Junk, low PCR	EMPTY
karyopherin (importin) beta 2	U72069
valosin-containing protein	BF685476
cyclin-dependent kinase inhibitor 1B (p2	BC001971
rcd1 (required for cell differentiation,	BF744792
osteoblast specific factor 2 (fasciclin	N71912
proteasome (prosome, macropain) inhibito	AI498500
Homo sapiens cDNA: FLJ22488 fis, clone H	AI621055
ribonuclease, RNase A family, 1 (pancrea	BG399435
Homo sapiens cDNA FLJ13641 fis, clone PL	AK023703
differentially expressed in hematopoieti	AI828567
Homo sapiens phosphatidylinositol 4-kina	EMPTY
ESTs	AL118669
guanine nucleotide binding protein (G pr	BG256994
Ts translation elongation factor, mitoch	AU143121
hypothetical protein FLJ20487	AW016445
ESTs	AW628630
transforming growth factor, beta recepto	XM_001924
ferritin, heavy polypeptide 1	BE878314
hyaluronan-mediated motility receptor (R	AU146502
KIAA0337 gene product	NM_014786
ESTs	AI168277
Junk, low PCR	EMPTY
ESTs	N49098
osteoblast specific factor 2 (fasciclin	AW608422
dysferlin, limb girdle muscular dystroph	BE179051
hepatocyte nuclear factor 4, alpha	AI631717
nicastrin	BE408912
myosin, light polypeptide 4, alkali; atr	AI038953
Human lysosomal glycosylasparaginase (AG	EMPTY
Homo sapiens mRNA full length insert cDN	AV704588
hypothetical protein FLJ20391	AA928886
proteasome (prosome, macropain) subunit,	BF205294
ESTs	AI391464
hypothetical protein FLJ22489	AI056386
proteasome (prosome, macropain) 26S subu	BG288544
No ID Incyte EST	EMPTY
ESTs	N20810
KIAA0273 gene product	NM_014759
YME1 (S.cerevisiae)-like 1	AJ295618
class I cytokine receptor	AF053004
ESTs, Weakly similar to AF257182 1 G-pro	AW264261
serine/threonine kinase 24 (Ste20, yeast	AU146392
neurotrophic tyrosine kinase, receptor,	AW024537
serologically defined colon cancer antig	NM_004713
highly charged protein	NM_005800

hepatocyte growth factor (hepapoietin A;	X16323
Human mercurial-insensitive water channe	EMPTY
DKFZP434C171 protein	AL080169
SH3-domain binding protein 1	AL157480
CDC-like kinase1	AK025306
RP42 homolog	AA587366
Sam68-like phosphotyrosine protein, T-ST	AA007604
death-associated protein kinase 1	NM_004938
KIAA0020 gene product	NM_014878
Junk, low PCR	EMPTY
TIA1 cytotoxic granule-associated RNA-bi	AL520241
Homo sapiens mRNA; cDNA DKFZp434F053 (fr	AA195829
phytanoyl-CoA hydroxylase (Refsum diseas	AL517715
Junk, low PCR	EMPTY
X-ray repair complementing defective rep	AU153705
KIAA0826 protein	AB020633
vascular endothelial growth factor B	BG167387
immunoglobulin lambda-like polypeptide 3	AW898076
calcium binding atopy-related autoantige	BF431049
Human nuclear ribonucleoprotein particle	EMPTY
ESTs	AW337854
Homo sapiens mRNA; cDNA DKFZp564P116 (fr	AA732938
transcription factor 17	AL535717
Homo sapiens cDNA FLJ11904 fis, clone HE	AI908481
ESTs	AA436947
Junk, low PCR	EMPTY
proteasome (prosome, macropain) 26S subu	AB003102
ESTs, Weakly similar to CPF1 RAT CYTOCHR	BG149215
Homo sapiens mRNA; cDNA DKFZp586F1822 (f	AW955716
ceroid-lipofuscinosis, neuronal 5	NM_006493
transducer of ERBB2, 2	AL120376
SEC24 (S. cerevisiae) related gene famil	AI421248
Homo sapiens growth factor receptor-boun	XM_012695
KIAA1719 protein	AF052177
protein tyrosine phosphatase, receptor t	F08552
UDP-glucose ceramide glucosyltransferase	AI609116
solute carrier family 1 (glial high affi	Z31713
Human glutamate receptor 2 (HBGR2) mRNA,	EMPTY
hypothetical protein GL012	AI697287
ESTs, Weakly similar to AAB47496 NG5 [H.	AA707234
Junk, low PCR	EMPTY
alpha 1,2-mannosidase	AL583481
hypothetical protein FLJ20979	R11616
thyroid hormone receptor-associated prot	AB011165
Homo sapiens clone 23938 mRNA sequence	AF007142
ESTs, Weakly similar to AF113685 1 PRO09	AW303433
Rho guanine nucleotide exchange factor (	BE397399
ESTs	AI499800
defensin, beta 1	XM_005297
KIAA0788 protein	BE541714
tetraspan 3	BF967869

No ID Incyte EST	EMPTY
KIAA0703 gene product	NM_014861
Bicaudal D (Drosophila) homolog 1	U90030
RAD50 (S. cerevisiae) homolog	AF057300
Small nuclear ribonucleoprotein polypept	EMPTY
ESTs, Moderately similar to ZN75_HUMAN Z	AI572250
adaptor-related protein complex 3, mu 1	AU126774
zinc finger protein homologous to Zfp161	AL538817
ESTs	AA057445
ESTs	AA036659
protein kinase C, theta	NM_006257
Ras-related GTP-binding protein	AU150741
ESTs	AI887807
M-phase phosphoprotein 6	BG391161
hypothetical protein from EUROIMAGE 2168	AA885779
tuberous sclerosis 2	BG431241
ESTs	AA781856
ecotropic viral integration site 2B	AW007704
Human clone 23560 mRNA sequence	AW297388
Junk, low PCR	EMPTY
Fas-activated serine/threonine kinase	NM_006712
adaptor-related protein complex 1, sigma	XM_010316
Arabadopsis8-62.5	EMPTY
CD63 antigen (melanoma 1 antigen)	BE907932
ESTs	AA455079
CD24 antigen (small cell lung carcinoma	AU141274
CGG triplet repeat binding protein 1	AV682520
extracellular matrix protein 1	U68186
putative tumor suppressor	AL576483
chromatin assembly factor 1, subunit A (	NM_005483
schwannomin interacting protein 1	AV720838
ESTs	AA768288
ectodermal dysplasia 1, anhidrotic	AI015138
UDP-Gal:betaGlcNAc beta 1,4- galactosylt	AL558425
plexin A1	AI659773
MCF.2 cell line derived transforming seq	AB002360
cytochrome P450, subfamily XXVIA, polype	AL539668
CD3D antigen, delta polypeptide (TiT3 co	AA310902
CCAAT/enhancer binding protein (C/EBP),	BF343807
testis specific protein 1 (probe H4-1 p3	AA928551
Arabadopsis10-62.5	EMPTY
fibronectin 1	AW385690
hypothetical protein MGC2752	AW732217
(clone PWHLC2-24) myosin light chain 2	NM_013292
DKFZP566H073 protein	AW272284
Junk, low PCR	EMPTY
myelin associated glycoprotein	X98405
phosphodiesterase 8A	AL109687
ESTs	BF195734
ESTs	AA927462
hypothetical protein FLJ10352	AW172759

Junk, low PCR	EMPTY
DKFZP564G092 protein	AW779168
KIAA1701 protein	AF035288
Junk, low PCR	EMPTY
nitric oxide synthase 3 (endothelial cel	AI082109
replication factor C (activator 1) 2 (40	BE295474
myosin, heavy polypeptide 7, cardiac mus	XM_012330
Empty	EMPTY
endothelial differentiation-related fact	AI797795
hypothetical protein MGC5338	AW614057
B-factor, properdin	AF019413
Janus kinase 1 (a protein tyrosine kinas	AA131737
REMOVED_FROM_DATABASE	EMPTY
Junk, low PCR	EMPTY
H2A histone family, member L	BF726950
hypothetical protein	AI150554
protein tyrosine phosphatase-like (proli	AV720189
hypothetical protein FLJ10468	BF978116
paired mesoderm homeo box 1	NM_022716
Homo sapiens mRNA; cDNA DKFZp564O1262 (f	AA922441
RaP2 interacting protein 8	NM_006695
synovial sarcoma, X breakpoint 3	NM_021014
Janus kinase 3 (a protein tyrosine kinas	NM_000215
hypoxia-inducible factor 1, alpha subuni	AU154668
matrix metalloproteinase 13 (collagenase	NM_002427
Empty	EMPTY
interferon consensus sequence binding pr	AW515838
Homo sapiens, Similar to gene rich clust	AI123342
claudin 10	AV728902
neurotrimin	AI262562
sperm specific antigen 2	XM_002507
mitogen-activated protein kinase kinase	NM_006301
endothelin 1	BE904559
purine-rich element binding protein B	BE884310
Homo sapiens cDNA FLJ13706 fis, clone PL	AI051561
ESTs, Moderately similar to ALU1_HUMAN A	AA939273
a disintegrin and metalloproteinase doma	NM_003815
Homo sapiens mRNA; cDNA DKFZp434M162 (fr	W02719
arylsulfatase B	NM_000046
adhesion glycoprotein	XM_008704
leukocyte immunoglobulin-like receptor,	NM_006866
zinc finger protein 173	AA506978
Charot-Leyden crystal protein	AA534845
Empty	EMPTY
FK506 binding protein precursor	AI312534
ESTs	AA708676
mesenchyme homeo box 2 (growth arrest-sp	AA489656
hypothetical protein FLJ10199	AW118985
ribonuclease 6 precursor	BE881018
requiem, apoptosis response zinc finger	BE384951
zinc finger protein-like 1	BF684855

nebulette	AI700281
Homo sapiens cDNA: FLJ23220 fis, clone A	AI130021
ESTs	AA195902
2',3'-cyclic nucleotide 3' phosphodieste	AK026876
hypothetical protein FLJ22501	BG232039
interleukin 8 receptor, alpha	NM_000634
growth arrest-specific 11	AL561072
ATPase, H+ transporting, lysosomal (vacu	AI678997
Junk, low PCR	EMPTY
carboxypeptidase B2 (plasma)	NM_001872
Empty	EMPTY
thiopurine S-methyltransferase	BG170081
ESTs	AW770245
TAR DNA binding protein	AL045296
nudix (nucleoside diphosphate linked moi	AW292786
four and a half LIM domains 3	BF220053
Friedreich ataxia region gene X123	L27479
Junk, low PCR	EMPTY
ESTs	AA730274
ESTs	H19155
Homo sapiens PNAS-129 mRNA, complete cds	AI808409
tumor necrosis factor receptor superfami	AF012629
ESTs	AW973487
ring finger protein 15	NM_006355
TATA box binding protein (TBP)-associate	AW167514
E74-like factor 2 (ets domain transcript	BE219087
selenophosphate synthetase 2	AW957160
Homo sapiens, Similar to RIKEN cDNA A930	H61878
Junk, low PCR	EMPTY
ESTs, Weakly similar to open reading fra	AI986451
hypothetical protein MGC10433	BE388177
KIAA1557 protein	AB046777
Junk, low PCR	EMPTY
cyclin G1	BC000196
inhibin, beta B (activin AB beta polypep	NM_002193
KIAA0741 gene product	AJ006412
very low density lipoprotein receptor	BG028841
interleukin 10 receptor, beta	BC001903
ESTs, Highly similar to dJ655K7.1 [H.sap	AW163204
DC2 protein	BG534029
retinoic acid receptor responder (tazaro	AI887421
No ID Incyte EST	EMPTY
chloride intracellular channel 2	BE876853
meningioma expressed antigen 6 (coiled-c	AA446039
hypothetical protein	AI820608
protein phosphatase 4, regulatory subuni	AW865929
interleukin 11 receptor, alpha	NM_004512
Homo sapiens cDNA FLJ11465 fis, clone HE	AA700158
angiopoietin 2	AB009865
ESTs	N64757
Junk, low PCR	EMPTY

Junk, low PCR	EMPTY
KIAA0553 protein	AB011125
chaperonin containing TCP1, subunit 2 (b	AU157811
heterogeneous nuclear ribonucleoprotein	AU131950
natriuretic peptide precursor B	AL021155
EST	N66152
Junk, low PCR	EMPTY
retinal outer segment membrane protein 1	NM_000327
plakophilin 2	NM_004572
collagen, type IX, alpha 1	BF196880
inositol polyphosphate-4-phosphatase, ty	AW950490
Homo sapiens cDNA FLJ10229 fis, clone HE	AL548337
REV3 (yeast homolog)-like, catalytic sub	AL096744
poly(A)-binding protein, cytoplasmic 1	NM_002568
hypothetical protein	AW205153
crystallin, mu	XM_012491
Junk, low PCR	EMPTY
KIAA0153 protein	BC001070
solute carrier family 7 (cationic amino	AB018009
matrilin 3	AJ001047
Junk, low PCR	EMPTY
phosphoinositide-3-kinase, regulatory su	Y08991
Junk, low PCR	EMPTY
No ID Incyte EST	EMPTY
ESTs	AI702459
ubiquitin-conjugating enzyme E2H (homolo	AW206129
postmeiotic segregation increased 2-like	D38499
wingless-type MMTV integration site fami	XM_006222
Homer, neuronal immediate early gene, 2	BE742561
BH3 interacting domain death agonist	AA480285
solute carrier family 12 (potassium/chlo	NM_005072
NADH dehydrogenase (ubiquinone) 1, alpha	BE795372
ESTs	AA970119
transcription factor 21	AA027107
ESTs	BF116042
H2.0 (Drosophila)-like homeo box 1	XM_010652
autocrine motility factor receptor	AI272698
YY1-associated factor 2	BE894451
ubiquitin-conjugating enzyme E2M (homolo	AA127782
coronin, actin-binding protein, 2A	BC000010
RAB32, member RAS oncogene family	AI826630
ESTs	AI018306
Junk, low PCR	EMPTY
programmed cell death 5	AA452724
U1-snRNP binding protein homolog (70kD)	AI285573
cytochrome P450, subfamily XXIA (steroid	AF019413
nuclear RNA export factor 1	NM_006362
protein kinase, interferon-inducible dou	AF083033
beclin 1 (coiled-coil, myosin-like BCL2-	BG025795
Rab geranylgeranyltransferase, alpha sub	XM_007311
ESTs	AA962558

KIAA0349 protein	AW976158
Junk, low PCR	EMPTY
tumor necrosis factor (ligand) superfami	BE349175
CDC-like kinase 3	BE908195
beaded filament structural protein 1, fi	Y16718
SWI/SNF related, matrix associated, acti	BE778256
tyrosine 3-monooxygenase/tryptophan 5-mo	AU142852
tumor suppressing subtransferable candid	AI815922
ESTs	AA028016
heat shock 90kD protein 1, alpha	AW958952
Junk, low PCR	EMPTY
ficolin (collagen/fibrinogen domain-cont	AI052239
heat shock 70kD protein 9B (mortalin-2)	BE893054
syntaxin 6	NM_005819
oral-facial-digital syndrome 1 gene	BF980109
emerin (Emery-Dreifuss muscular dystroph	BG281822
early growth response 2 (Krox-20 (Drosop	AF139463
eukaryotic translation initiation factor	NM_003760
far upstream element (FUSE) binding prot	AW173306
ESTs, Weakly similar to ALUC_HUMAN !!!!	AI983432
syndecan binding protein (syntenin)	AU139181
TATA box binding protein (TBP)-associate	BG164406
matrix metalloproteinase 16 (membrane-in	NM_005941
basic leucine zipper transcription facto	NM_006399
c6.1A	X64643
ortholog of rat pippin	U79280
ESTs	AI051616
Homo sapiens cDNA: FLJ23006 fis, clone L	AK026659
Kell blood group precursor (McLeod pheno	NM_021083
ATPase, Ca++ transporting, plasma membra	BF432072
growth differentiation factor 11	AF100907
peroxisomal membrane protein 3 (35kD, Ze	BG501590
KIAA0903 protein	AI022927
prion protein (p27-30) (Creutzfeld-Jakob	BG179573
H.sapiens mRNA for ribosomal protein L26	EMPTY
Junk, low PCR	EMPTY
X-ray repair complementing defective rep	BC002949
ESTs	AA516420
dopa decarboxylase (aromatic L-amino aci	NM_000790
ATP-binding cassette, sub-family B (MDR/	X87344
Homo sapiens mRNA; cDNA DKFZp586H0324 (f	AA702877
sialyltransferase 4C (beta-galactosidase	AA453813
Junk, low PCR	EMPTY
eosinophil peroxidase	X14346
RNA polymerase II transcriptional regula	BE891802
ESTs	AW293233
KIAA1145 protein	AW190829
semenogelin II	AA687962
carcinoembryonic antigen-related cell ad	D90277
protein tyrosine phosphatase, non-recept	BE906686
keratin 17	AI521362

glucagon	BF980737
Human cytochrome bc-1 complex core prote	EMPTY
centromere protein F (350/400kD, mitosin	NM_005196
receptor (calcitonin) activity modifying	AL548945
Homo sapiens brain my038 protein mRNA, c	AF063596
calmegin	XM_003352
Junk, low PCR	EMPTY
zinc finger protein 232	BE138901
v-crk avian sarcoma virus CT10 oncogene	XM_008605
ESTs	AA252029
forkhead box O1A (rhabdomyosarcoma)	NM_002015
myeloid/lymphoid or mixed-lineage leukem	AB011399
ESTs	AL518022
chromosome 12 open reading frame 4	AA886652
No ID Incyte EST	EMPTY
cathepsin B	AL135262
intercellular adhesion molecule 3	NM_002162
Junk, low PCR, low PCR	EMPTY
DnaJ (Hsp40) homolog, subfamily B, membe	AL565624
Homo sapiens (clone 13a) deoxyhypusine s	EMPTY
Junk, low PCR	EMPTY
COP9 (constitutive photomorphogenic, Ara	AI141849
RAB9-like protein	N80848
Junk, low PCR	EMPTY
phospholipase C, gamma 2 (phosphatidylin	BG391985
Homo sapiens mRNA; cDNA DKFZp564D016 (fr	AI347511
Junk, low PCR	EMPTY
hypothetical protein FLJ20163	AA398584
KIAA0176 protein	D79998
ATP synthase, H+ transporting, mitochond	AI110705
hypothetical protein FLJ10535	AI090629
group XII secreted phospholipase A2	N72014
DNA segment on chromosome X (unique) 987	AI360458
regulatory factor X, 2 (influences HLA c	AC005784
Human DNA sequence from clone 889N15 on	AL031177
Junk, low PCR, low PCR	EMPTY
adaptor-related protein complex 3, delta	BE799757
Spermidine/spermine N1-acetyltransferase	EMPTY
titin	NM_003319
signal sequence receptor, alpha (translo	AA993117
Junk, low PCR	EMPTY
Junk, low PCR	EMPTY
similar to S. cerevisiae SSM4	NM_005885
hypothetical protein FLJ22995	NM_024831
phosphatidylserine synthase 1	NM_014754
Junk, low PCR	EMPTY
adrenergic, beta, receptor kinase 2	BG476807
phosphorylase, glycogen; muscle (McArdle	XM_006535
Junk, low PCR	EMPTY
putative G protein-coupled receptor	AA769179
Junk, low PCR	EMPTY

integrin-binding sialoprotein (bone sial	XM_003371
mitogen-activated protein kinase 11	AI394426
EphB1	NM_004441
matrix metalloproteinase 1 (interstitial	NM_002421
Human cytoplasmic chaperonin hTRiC5 mRNA	EMPTY
Homo Sapiens (clone B3B3E13) chromosome	BE789464
microspherule protein 1	AL573101
DKFZP564B167 protein	AA807386
lysosomal-associated membrane protein 2	AU122484
leucine-zipper-like transcriptional regu	BF793167
Incyte EST	NO ID
ectonucleoside triphosphate diphosphohyd	AF144748
mitogen-activated protein kinase 9	AA862471
fatty acid desaturase 1	AF035284
homeodomain-interacting protein kinase 3	NM_005734
Homo sapiens cDNA FLJ14232 fis, clone NT	AK025753
Homo sapiens cDNA FLJ13996 fis, clone Y7	AI358095
HLA-G histocompatibility antigen, class	AF071019
tetraspan 1	BG536266
lymphocyte antigen 6 complex, locus H	AI929550
Junk, low PCR, low PCR	EMPTY
ornithine aminotransferase (gyrate atrop	AL549701
Human mRNA for cytochrome c oxidase subu	EMPTY
protein phosphatase 2 (formerly 2A), reg	AL536030
Human clone 23612 mRNA sequence	U90902
chromosome 11 hypothetical protein ORF3	BE296758
collagen, type XI, alpha 2	AL031228
Junk, low PCR	EMPTY
ESTs	BE552186
Junk, low PCR	EMPTY
cytochrome c oxidase subunit VIII	AV706297
potassium voltage-gated channel, shaker-	AL035406
zeta-chain (TCR) associated protein kina	L05148
ESTs	AA417138
Homo sapiens cDNA FLJ11812 fis, clone HE	AI075653
lymphocyte antigen 6 complex, locus D	BE563202
KIAA0390 gene product	NM_014717
EGF-like-domain, multiple 3	BG392803
Junk, low PCR, low PCR	EMPTY
solute carrier family 22 (organic cation	NM_003059
Arabadopsis2-62.5	EMPTY
KIAA0441 gene product	NM_014797
Junk, low PCR	EMPTY
KIAA0574 protein	AB011146
zinc finger protein 184 (Kruppel-like)	BG254958
Junk, low PCR	EMPTY
hypothetical protein FLJ22009	BG253352
bone morphogenetic protein 7 (osteogenic	BE740547
Homo sapiens mRNA for FLJ00016 protein,	AW007710
solute carrier family 1 (neutral amino a	BF206100
cysteine and glycine-rich protein 1	AU117500

ESTs	BE219380
KIAA0077 protein	AU143966
bladder cancer associated protein	AA595446
hypothetical protein dJ434O14.3	NM_025228
interferon, alpha 2	V00549
meningioma (disrupted in balanced transl	NM_002430
KIAA0678 protein	AW969397
Arabadopsis4-62.5	EMPTY
Junk, low PCR	EMPTY
protein phosphatase 1, catalytic subunit	BG335480
glutaminyl-peptide cyclotransferase (glu	AV707013
interferon-induced protein with tetratri	AL544297
neutrophil cytosolic factor 4 (40kD)	NM_013416
hypothetical protein FLJ10407	AI688930
phosphodiesterase 1C, calmodulin-depende	U40372
paired box gene 8	AI948472
cytidine deaminase	AV649252
non-metastatic cells 3, protein expresse	BC000250
methylmalonate-semialdehyde dehydrogenas	AV708459
MDS024 protein	AA580484
KIAA0645 gene product	BE646437
putative DNA binding protein	AF073293
trinucleotide repeat containing 12	U80743
Junk, low PCR	EMPTY
topoisomerase (DNA) III beta	NM_003935
Arabadopsis6-62.5	EMPTY
solute carrier family 22 (organic cation	NM_007105
glucose-6-phosphatase, transport (glucos	AL583096
TALE homeobox protein Meis2e	XM_012430
Junk, low PCR	EMPTY
myosin IC	NM_004998
KIAA0957 protein	AW103682
NIMA (never in mitosis gene a)-related k	Z29067
hypothetical protein	AI217620
cell division cycle 25A	BG424887
ESTs	BG548668
ESTs	BF060712
hypothetical protein FLJ23412	AW292227
angiogenin, ribonuclease, RNase A family	BE348689
collagen, type XV, alpha 1	L01697
No ID Incyte EST	EMPTY
Junk, low PCR	EMPTY
kinesin family member 13B	AI459565
Empty	EMPTY
carboxypeptidase A2 (pancreatic)	NM_001869
CDC28 protein kinase 1	AA765850
pyruvate dehydrogenase kinase, isoenzyme	BF182833
polymerase (DNA directed), alpha	NM_016937
Junk, low PCR	EMPTY
Homo sapiens clone FLC0675 PRO2870 mRNA,	AI472475
chloride channel 3	AF172729

LENG5 protein	AA861789
Junk, low PCR	EMPTY
Junk, low PCR	EMPTY
ESTs, Weakly similar to ALU7_HUMAN ALU S	AI435603
ESTs, Weakly similar to dJ309K20.4 [H.sa	N51505
Kruppel-like factor 4 (gut)	BF446905
leukocyte immunoglobulin-like receptor,	AF009644
proteasome (prosome, macropain) subunit,	BG485050
eukaryotic translation initiation factor	AA313584
Rap1 guanine-nucleotide-exchange factor	AA453498
Empty	EMPTY
Junk, low PCR	EMPTY
No ID Incyte EST	EMPTY
lactate dehydrogenase A	AU142452
ATP-binding cassette, sub-family B (MDR/	M14758
KIAA0008 gene product	NM_014750
hypothetical protein FLJ21801	AI698273
hepatocyte nuclear factor 4, alpha	AL132772
MAWD binding protein	AI765890
KIAA0596 protein	AB011168
myeloid/lymphoid or mixed-lineage leukem	NM_004641
zinc finger protein 226	AF246126
ESTs	AW468566
small inducible cytokine subfamily B (Cy	NM_002993
low molecular mass ubiquinone-binding pr	AW162336
putative brain nuclearly-targeted protei	AL109827
Junk, low PCR	EMPTY
ubiquitin specific protease 12	AL049221
Empty	EMPTY
protease, serine, 12 (neurotrypsin, moto	NM_003619
cAMP responsive element modulator	AL117336
ubiquitin C	AU160779
valyl-tRNA synthetase 2	BE262992
glucocorticoid receptor DNA binding fact	AB051509
hypothetical protein FLJ10349	XM_002117
reticulon 2	AF038540
Homo sapiens cDNA: FLJ22355 fis, clone H	BG427282
solute carrier family 16 (monocarboxylic	NM_004696
apolipoprotein C-III	NM_000040
No ID Incyte EST	EMPTY
Homo sapiens mRNA for KIAA1724 protein,	AI916530
delta (Drosophila)-like 1	AW118784
cryptochrome 2 (photolyase-like)	AB014558
6-phosphofructo-2-kinase/fructose-2,6-bi	NM_002625
Junk, low PCR	EMPTY
smoothened (Drosophila) homolog	BE785546
guanylate binding protein 1, interferon-	BF913224
membrane-bound transcription factor prot	BE388188
matrix Gla protein	AI298747
ESTs, Weakly similar to ACY2_HUMAN ASPAR	AI277021
COP9 homolog	BG540353

proteasome (prosome, macropain) 26S subu	BE392801
hypothetical protein FLJ13187	AI360785
Junk, low PCR	EMPTY
lipocalin 1 (protein migrating faster th	L14927
Homo sapiens cDNA FLJ12807 fis, clone NT	BF477754
Junk, low PCR	EMPTY
DKFZP434I092 protein	AA707503
neurexin 3	NM_004796
lymphocyte antigen 6 complex, locus E	AL548138
membrane protein, palmitoylated 3 (MAGUK	BE669711
No ID Incyte EST	EMPTY
sema domain, immunoglobulin domain (Ig),	NM_006379
8-oxoguanine DNA glycosylase	AB019531
CUG triplet repeat, RNA-binding protein	U69546
proteasome (prosome, macropain) 26S subu	D31889
golgin-67	AW157581
Homo sapiens, clone IMAGE:3603836, mRNA,	BE222511
tyrosinase-related protein 1	X51420
glutamate receptor, metabotropic 2	AB045011
Homo sapiens clone FLB8436 PRO2277 mRNA,	BE827697
tetraspan 2	AI924594
phosphatidylinositol glycan, class A (pa	S74936
hypothetical protein FLJ11085	AI187171
follistatin	NM_013409
map kinase phosphatase-like protein MK-S	XM_004906
interleukin 1, beta	W38319
cadherin 2, type 1, N-cadherin (neuronal	M34064
Homo sapiens DNA from chromosome 19, cos	AC004030
mitogen-activated protein kinase kinase	AL556109
cystathionase (cystathionine gamma-lyase	XM_001834
solute carrier family 12 (sodium/potassi	NM_000338
KIAA0397 gene product	AB007857
dynactin 2 (p50)	BG104697
Junk, low PCR	EMPTY
ESTs, Weakly similar to ALU1_HUMAN ALU S	BF196079
phospholipase A2, group IIA (platelets,	R80611
Junk, low PCR	EMPTY
Crm (Cramped Drosophila)-like	AW974201
prolactin receptor	AA033953
prodynorphin	NM_024411
ESTs, Weakly similar to ALU1_HUMAN ALU S	AA721124
plasminogen activator, urokinase recepto	BE386005
poly(A) polymerase alpha	N46321
ABO blood group (transferase A, alpha 1-	D82837
collagen, type VI, alpha 1	AL570594
lectin, galactoside-binding, soluble, 4	AA130458
retinoic acid receptor, gamma	NM_000966
retinitis pigmentosa 2 (X-linked recessi	AW243690
RNA binding motif protein 5	AL556879
phosphoribosyl pyrophosphate amidotransf	AU159995
glutamyl-prolyl-tRNA synthetase	AI656146

ESTs	AA557388
Homo sapiens mRNA; cDNA DKFZp586P1622 (f	BE787827
interferon regulatory factor 7	NM_004029
serine/threonine kinase 4	NM_006282
bladder cancer overexpressed protein	AI191665
ATPase, H+ transporting, lysosomal (vacu	NM_001692
potassium voltage-gated channel, KQT-lik	AF110020
ESTs	BF033855
heterogeneous nuclear ribonucleoprotein	BE742346
HSPC135 protein	AA936753
G protein-coupled receptor 51	AF095724
glutathione peroxidase 2 (gastrointestin	BE795731
No ID Incyte EST	EMPTY
butyrophilin, subfamily 3, member A2	AW474512
v-ets avian erythroblastosis virus E26 o	AL578223
Rho GDP dissociation inhibitor (GDI) bet	BG388517
T-cell acute lymphocytic leukemia 1	AJ131016
tissue specific transplantation antigen	AL548615
ESTs	AA418721
ESTs, Weakly similar to ALU1_HUMAN ALU S	T80121
MMS19 (MET18 S. cerevisiae)-like	NM_022362
plasminogen	K02922
caspase recruitment domain 4	AF113925
ATPase, Ca++ transporting, plasma membra	AI885833
NIMA (never in mitosis gene a)-related k	AA923277
hypothetical protein PRO1855	AI832815
Junk, low PCR	EMPTY
No ID Incyte EST	EMPTY
distal-less homeo box 2	BE905163
WAS protein family, member 1	NM_003931
Human clone CE29 7.2 (CAC)n/(GTG)n repea	AW197081
signal peptidase complex (18kD)	AL555167
synuclein, beta	S69965
telomerase reverse transcriptase	NM_003219
transketolase-like 1	NM_012253
KIAA0310 gene product	BC001404
hypothetical protein DKFZp434G0522	AI186307
hypothetical protein FLJ20758	AA121270
guanine nucleotide binding protein (G pr	BF976696
KIAA0304 gene product	BE410539
ESTs, Moderately similar to PC7084 GTP-b	AW593261
phosphorylase kinase, gamma 1 (muscle)	BE410974
Junk, low PCR	EMPTY
lipoma HMGIC fusion partner	N67270
melanoma inhibitory activity	NM_006533
ESTs	AA654772
dead ringer (Drosophila)-like 1	AI394608
iduronidase, alpha-L-	W25580
caspase 8, apoptosis-related cysteine pr	BG529842
vasoactive intestinal peptide receptor 2	NM_003382
GCIP-interacting protein p29	AI934146

glyceronephosphate O-acyltransferase	AF043937
H.sapiens mRNA for cyclin H assembly fac	EMPTY
splicing factor 3b, subunit 3, 130kD	BC003146
caspase 6, apoptosis-related cysteine pr	BG494677
ESTs, Weakly similar to KIAA1435 protein	AW673842
Junk, low PCR	EMPTY
phosphorylase kinase, alpha 2 (liver)	BF941396
ESTs	AW090060
peroxisomal biogenesis factor 7	NM_000288
podocalyxin-like	BE395330
Junk, low PCR	EMPTY
Junk, low PCR	EMPTY
ESTs	AI078015
Junk, low PCR	EMPTY
recoverin	NM_002903
zinc finger protein 205	W93354
tyrosine kinase, non-receptor, 1	BF871607
KIAA0515 protein	AB011087
CD37 antigen	AW406508
Human liver glutamate dehydrogenase mRNA	EMPTY
hypothetical protein	NM_020314
ring finger protein	XM_004547
nuclear receptor binding factor-2	AA960998
TNF receptor-associated factor 1	NM_005658
proteasome (prosome, macropain) subunit,	BG178069
ESTs, Weakly similar to A35659 krueppel-	AW245384
Junk, low PCR	EMPTY
PAK-interacting exchange factor beta	NM_003899
dermatopontin	AW016451
hypothetical protein FLJ20693	AW418886
ESTs	H80397
ESTs	AW614008
breast cancer anti-estrogen resistance 3	AW968506
lunatic fringe (Drosophila) homolog	AF193612
TTK protein kinase	NM_003318
colony stimulating factor 2 receptor, be	AW959214
v-myc avian myelocytomatosis viral relat	BF313082
Human mitochondrial ADP/ADT translocator	EMPTY
kinesin family member 5C	AA625394
interleukin 7 receptor	XM_004013
ESTs	AI910984
Junk, low PCR	EMPTY
v-yes-1 Yamaguchi sarcoma viral related	BG108304
cyclin-dependent kinase 5, regulatory su	H97961
microfibrillar-associated protein 3	NM_005927
putative glycine-N-acyltransferase	AB013093
integral membrane protein 3	AA781598
KIAA0373 gene product	NM_014684
programmed cell death 6	BE327923
hypothetical protein FLJ22471	BE906338
hypothetical protein FLJ20154	NM_017690

C-terminal binding protein 1	BF792323
serine/threonine kinase 24 (Ste20, yeast	NM_003576
myeloid/lymphoid or mixed-lineage leukem	BF195699
selectin P (granule membrane protein 140	NM_003005
Human malate dehydrogenase (MDHA) mRNA,	EMPTY
RAB interacting factor	U74324
KIAA0586 gene product	NM_014749
RBP1-like protein	N67246
selenoprotein P, plasma, 1	AU148217
tyrosine kinase 2	AU159485
wingless-type MMTV integration site fami	AW872887
solute carrier family 16 (monocarboxylic	AK000416
cytoplasmic linker 2	AB006629
FERM, RhoGEF (ARHGEF) and pleckstrin dom	AA203222
dual adaptor of phosphotyrosine and 3-ph	AI041585
Homo sapiens, Similar to rhotekin, clone	AI498180
EST	AA773613
chitinase 3-like 2	BF526854
KIAA0410 gene product	NM_014778
3'-phosphoadenosine 5'-phosphosulfate sy	NM_004670
GCN5 (general control of amino-acid synt	AV698100
ELKL motif kinase	XM_011990
Unknown - Human Control Plate Well H9	EMPTY
dynein, cytoplasmic, light intermediate	NM_006141
histidine-rich glycoprotein	AV652795
ESTs	T23939
hypothetical protein	AI963556
colipase, pancreatic	BE970031
Homo sapiens, Similar to germ cell-speci	AA063062
zinc finger protein, Y-linked	AF114156
ring finger protein 4	NM_002938
hypothetical protein	AI351845
hypothetical protein MGC3156	AU152300
Homo sapiens cDNA: FLJ23013 fis, clone L	AI249658
ESTs	AI148143
rho/rac guanine nucleotide exchange fact	AL512715
adipose specific 2	AI093004
retinoic acid receptor, beta	AA419238
cytochrome P450, subfamily XXVIIA (stero	AL524490
BCL2-interacting killer (apoptosis-induc	AL022237
Empty	EMPTY
neighbor of COX4	W07323
T cell receptor beta locus	X00437
DNA segment on chromosome 19 (unique) 11	BG236014
signal recognition particle 9kD	AV711053
gamma-glutamyltransferase 1	J05235
Homo sapiens cyclic AMP-regulated phosph	N40915
GTPase activating protein-like	AB011110
Rab9 effector p40	BC000503
nuclear factor of activated T-cells, cyt	AA477581
hypothetical protein FLJ13639	AA501987

Homo sapiens mRNA; cDNA DKFZp434K152 (fr	AI026721
ESTs	AA973681
eukaryotic translation initiation factor	BE294246
No ID Incyte EST	EMPTY
granzyme B (granzyme 2, cytotoxic T-lymp	NM_004131
activating transcription factor 3	N39944
Junk, low PCR	EMPTY
Empty	EMPTY
NADH dehydrogenase (ubiquinone) 1 alpha	AW006760
ESTs	AI401460
Junk, low PCR	EMPTY
KIAA0175 gene product	AW003353
Junk, low PCR	EMPTY
hypothetical protein RP4-622L5	AI138733
ESTs	AI763336
KIAA0435 gene product	NM_014801
interferon regulatory factor 6	NM_006147
hypothetical protein FLJ20699	AW027924
indoleamine-pyrrole 2,3 dioxygenase	AU138239
ESTs	AA670368
membrane metallo-endopeptidase (neutral	Y00811
Human Chromosome 16 BAC clone CIT987SK-A	AA186546
H.sapiens mRNA for TRE5	X78262
Junk, low PCR	EMPTY
TAR (HIV) RNA-binding protein 2	AL523706
Empty	EMPTY
arginase, liver	BG542163
ESTs	AA431300
KIAA0836 protein	AA843933
Junk, low PCR	EMPTY
cytochrome P450, subfamily I (dioxin-ind	NM_000104
hypothetical protein FLJ23516	AA595575
HIRIP5 protein; HIRA-interacting protein	AA206911
sterol-C5-desaturase (fungal ERG3, delta	AI394121
Junk, low PCR	EMPTY
DKFZP434B0335 protein	AW272594
hepatocyte nuclear factor 3, alpha	BG287384
clone HQ0477 PRO0477p	BF056917
thyroid hormone receptor interactor 8	Z39133
No ID Incyte EST	EMPTY
ATP-binding cassette, sub-family B (MDR/	AU132766
CD4 antigen (p55)	NM_000616
dual-specificity tyrosine-(Y)-phosphoryl	AF186774
Empty	EMPTY
antigen identified by monoclonal antibod	AF063591
ESTs	AA670369
Wiskott-Aldrich syndrome protein interac	AW086445
Junk, low PCR	EMPTY
gamma-glutamyl hydrolase (conjugase, fol	AL522691
ESTs	AI821999
Homo sapiens mRNA; cDNA DKFZp434E033 (fr	AI919212

enoyl Coenzyme A hydratase, short chain,	AL549816
excision repair cross-complementing rode	NM_005236
chromosome 11 open reading frame 24	AI499213
nucleoporin 98kD	U41815
mRNA for FLJ00023 protein	BF769921
sperm associated antigen 6	NM_012443
neuronal thread protein	NM_014486
CCR4-NOT transcription complex, subunit	NM_013316
nuclear RNA export factor 1	AW025508
KIAA0690 protein	AI188138
Empty	EMPTY
sortilin-related receptor, L(DLR class)	NM_003105
KIAA0996 protein	AW770225
RAB38, member RAS oncogene family	AI367782
Junk, low PCR	EMPTY
attractin	AL132773
gonadotropin-regulated testicular RNA he	AI680671
AT-hook transcription factor AKNA	AW276108
NADH dehydrogenase (ubiquinone) Fe-S pro	BF245775
POM (POM121 rat homolog) and ZP3 fusion	AA383329
hypothetical protein	AA903507
Junk, low PCR	EMPTY
Homo sapiens mRNA; cDNA DKFZp434D0428 (f	AW015987
transmembrane protein 1	AB001517
surface glycoprotein, Ig superfamily mem	NM_016952
molybdopterin synthase sulfurylase	XM_009466
Junk, low PCR	EMPTY
sprouty (Drosophila) homolog 2	NM_005842
Empty	EMPTY
nucleosome assembly protein 1-like 4	BG282426
ESTs	AA610122
hypothetical protein FLJ21439	AA700175
Junk, low PCR	EMPTY
tuftelin-interacting protein	AA046677
ESTs	AA704966
ESTs	AA493491
endocytic receptor (macrophage mannose r	AF134838
glutamate decarboxylase 2 (pancreatic is	AI497983
hypothetical protein FLJ22116	BE327849
G protein-coupled receptor 4	U21051
ESTs	N39074
Homo sapiens, Similar to Bicaudal D (Dro	AI698095
interferon, beta 1, fibroblast	V00546
dimethylarginine dimethylaminohydrolase	AL078459
butyrate response factor 1 (EGF-response	NM_004926
poly (ADP-ribose) glycohydrolase	NM_003631
Empty	EMPTY
glutamate-cysteine ligase, modifier subu	NM_002061
ESTs, Moderately similar to ALU7_HUMAN A	AA631812
hypothetical protein FLJ10420	AL573766
B-cell CLL/lymphoma 2	XM_008738

solute carrier family 7 (cationic amino	NM_003983
connector enhancer of KSR-like (Drosophi	BE221409
ESTs	AA251570
transgelin 2	AU126823
retinitis pigmentosa GTPase regulator	NM_000328
WD repeat domain 10	AK024435
Junk, low PCR	EMPTY
Homo sapiens cDNA FLJ14236 fis, clone NT	AI334647
nescient helix loop helix 1	M96739
arachidonate 12-lipoxygenase, 12R type	NM_001139
coproporphyrinogen oxidase (coproporphyr	XM_002831
claudin 4	BF036516
f-box and leucine-rich repeat protein 7	AW614058
Junk, low PCR	EMPTY
sortilin 1	NM_002959
ESTs	AA215320
Down syndrome critical region gene 1	AU124628
KIAA0322 protein	AB048365
hypothetical protein	BF475450
Homo sapiens, clone MGC:12257, mRNA, com	BC005233
ribosomal protein L18	AA323244
Junk, low PCR	EMPTY
ESTs, Weakly similar to zinc finger prot	AA946752
hypothetical protein FLJ11838	AI281162
ESTs	AI479330
serine/threonine kinase 29	AF020089
CDC10 (cell division cycle 10, S. cerevi	AU135444
TEK tyrosine kinase, endothelial (venous	AL047086
STIP1 homology and U-Box containing prot	BF526386
interleukin 6 receptor	NM_000565
tumor necrosis factor (ligand) superfami	BG170786
acetyl-Coenzyme A acyltransferase 1 (per	XM_002882
regenerating islet-derived 1 alpha (panc	BC005350
hypothetical protein DKFZp586H0623	AA461425
phosphodiesterase 4B, cAMP-specific (dun	L12686
proteasome (prosome, macropain) activato	AA310524
ESTs	AA938980
SMC (mouse) homolog, Y chromosome	AF273841
ribosomal protein L3	AV756387
orosomucoid 1	XM_011748
Junk, low PCR	EMPTY
Homo sapiens cDNA FLJ11903 fis, clone HE	AA464096
hypothetical protein FLJ20345	AW129735
loss of heterozygosity, 11, chromosomal	BC001234
cullin 5	NM_003478
basic leucine zipper nuclear factor 1 (J	AW969737
POU domain, class 2, transcription facto	BG106848
carcinoembryonic antigen-related cell ad	AI696953
KIAA0406 gene product	AL109823
replication factor C (activator 1) 4 (37	BF062845
docking protein 1, 62kD (downstream of t	NM_001381

vesicle-associated membrane protein 5 (m	AF077197
RAD23 (S. cerevisiae) homolog B	BF795639
adipose most abundant gene transcript 1	NM_004797
tripartite motif protein TRIM8	AF220034
tumor necrosis factor (ligand) superfami	AF053712
transmembrane 7 superfamily member 1 (up	NM_003272
G2 protein	U10991
bone morphogenetic protein receptor, typ	BF593960
hypothetical protein FLJ14281	AA261857
ESTs	N64455
Treacher Collins-Franceschetti syndrome	AI765758
translocase of inner mitochondrial membr	BG502292
protein tyrosine kinase 2 beta	BF796371
tumor necrosis factor receptor superfami	AA769631
exportin 1 (CRM1, yeast, homolog)	D89729
eukaryotic translation initiation factor	BG503248
a disintegrin and metalloproteinase doma	BF515855
Fanconi anemia, complementation group G	AW952789
ESTs, Weakly similar to T00331 hypotheti	R17434
abl-interactor 12 (SH3-containing protei	XM_010847
microphthalmia-associated transcription	AV717752
potassium channel modulatory factor	BG109888
synapsin II	AF077671
Junk, low PCR	EMPTY
mature T-cell proliferation 1	AL536910
Human DNA sequence from clone RP11-127L2	AI749754
ESTs	AI373233
ESTs	AI052511
hypothetical protein	BF528327
cytochrome P450, subfamily XIX (aromatiz	NM_000103
protein kinase C, nu	XM_002659
Junk, low PCR	EMPTY
accessory proteins BAP31/BAP29	BF978554
Junk, low PCR	EMPTY
transcription factor CP2	BC003634
calmodulin 2 (phosphorylase kinase, delt	AL533404
ESTs	N64198
splicing factor, arginine/serine-rich 7	AW949533
Junk, low PCR	EMPTY
solute carrier family 17 (anion/sugar tr	AF244577
small nuclear ribonucleoprotein polypept	AA737229
calcium channel, voltage-dependent, L ty	M92269
interleukin 10	NM_000572
ESTs	AI143430
ESTs, Highly similar to unnamed protein	BF940604
ESTs	AI363134
bromodomain, testis-specific	AA884041
KIAA0769 gene product	AW021430
Junk, low PCR	EMPTY
aquaporin 8	XM_007993
protective protein for beta-galactosidas	AL562902

PCTAIRE protein kinase 2	AL119276
microtubule-associated protein tau	BF343227
Junk, low PCR	EMPTY
reversion-inducing-cysteine-rich protein	AW192740
WAS protein family, member 3	S69790
elastase 1, pancreatic	BG542116
hypothetical protein FLJ22316	AA236726
WD repeat domain 7	AB011113
zyxin	NM_003461
Junk, low PCR	EMPTY
ESTs	N67037
myosin IE	AW276523
ESTs	W46422
KIAA0375 gene product	AB002373
calcium channel, voltage-dependent, beta	L06112
No ID Incyte EST	EMPTY
meningioma expressed antigen 6 (coiled-c	BF980666
KIAA0561 protein	AW071776
aldehyde dehydrogenase 3 family, member	XM_008515
Homo sapiens catechol-O-methyltransferas	EMPTY
suppressor of Ty (S.cerevisiae) 5 homolo	AL532087
DAZ associated protein 2	AL036958
translocase of outer mitochondrial membr	AL544642
protein phosphatase 3 (formerly 2B), cat	NM_005605
ancient conserved domain protein 4	BE906066
RAB3 GTPase-ACTIVATING PROTEIN	D31886
CDC5 (cell division cycle 5, S. pombe, h	AA219672
Junk, low PCR	EMPTY
Homo sapiens cDNA: FLJ23454 fis, clone H	AA705184
TIA1 cytotoxic granule-associated RNA-bi	BF198030
leptin receptor	AA808323
ESTs	AV724325
transforming growth factor beta-stimulat	BF852901
Homo sapiens mRNA; cDNA DKFZp434N2116 (f	AV648824
protein C receptor, endothelial (EPCR)	AF106202
decidual protein induced by progesterone	XM_005817
KIAA0244 protein	AK026492
H.sapiens mRNA for elongations factor Tu	EMPTY
cerebral cavernous malformations 1	BG163551
zinc finger protein 185 (LIM domain)	U82671
dolichyl-phosphate (UDP-N-acetylglucosam	AL560150
thiosulfate sulfurtransferase (rhodanese	BF972766
hypothetical protein FLJ10569	AI274857
neuralized (Drosophila)-like	AI739022
guanine nucleotide binding protein (G pr	BG251149
ESTs	AA812741
Junk, low PCR	EMPTY
KIAA0720 protein	BF033726
ESTs	AW628326
ESTs	AI028242
carboxypeptidase N, polypeptide 2, 83kD	J05158

ATP-binding cassette, sub-family B (MDR/	M23234
v-myb avian myeloblastosis viral oncogen	AW976979
prostaglandin I2 (prostacyclin) receptor	XM_009032
matrix metalloproteinase 15 (membrane-in	BG327005
Human liver-type 1-phosphofructokinase (	EMPTY
hypothetical protein, expressed in osteo	F12860
keratin 6A	L42611
TATA box binding protein (TBP)-associate	AU151990
Wilms tumor 1	NM_024425
Human DNA sequence from clone GS1-115M3	N30155
ceroid-lipofuscinosis, neuronal 2, late	BF337318
prefoldin 4	BG255394
No ID Incyte EST	EMPTY
ESTs, Weakly similar to ALU1_HUMAN ALU S	AI002517
Junk, low PCR	EMPTY
alpha integrin binding protein 63	AL042831
high-mobility group 20A	BF196736
estrogen receptor binding site associate	NM_004215
aldo-keto reductase family 1, member C4	AV694764
BCL2/adenovirus E1B 19kD-interacting pro	AA129678
kinesin-like 4	NM_007317
aldo-keto reductase family 1, member B1	BF213317
Human mRNA for heterogeneous nuclear rib	EMPTY
clusterin (complement lysis inhibitor, S	AL533269
zinc finger protein 38 (KOX 25)	X07290
3-oxoacid CoA transferase	NM_000436
KIAA0161 gene product	AW172840
ESTs	AW022870
glutamyl aminopeptidase (aminopeptidase	NM_001977
keratin 14 (epidermolysis bullosa simple	BE614947
Homo sapiens mRNA; cDNA DKFZp434I0535 (f	AA406607
ESTs, Weakly similar to I54338 zinc fing	BE893508
Junk, low PCR	EMPTY
KIAA0764 gene product	BE463934
ESTs	AA954969
macrophage myristoylated alanine-rich C	AU145579
potassium intermediate/small conductance	NM_002250
fatty acid binding protein 4, adipocyte	BG287253
biglycan	BG111541
SNARE protein	AI432607
Human glyceraldehyde 3-phosphate dehydro	EMPTY
secretory leukocyte protease inhibitor (	BG533465
bagpipe homeobox (Drosophila) homolog 1	NM_001189
KIAA0211 gene product	BF508616
cytochrome P450 isoform 4F12	AY008841
hypothetical protein FLJ13611	Z99387
Rho-associated, coiled-coil containing p	NM_005406
early development regulator 2 (homolog o	BE892356
ESTs	AV744404
ESTs	AI681269
hypothetical protein FLJ20366	AA890056

Junk, low PCR	EMPTY
ESTs	AI699361
protein kinase, AMP-activated, gamma 1 n	AL577774
contactin 1	NM_001843
spastic ataxia of Charlevoix-Saguenay (s	AB018273
bladder cancer overexpressed protein	AA748850
coagulation factor II (thrombin) recepto	NM_005242
Homo sapiens cadherin-13 mRNA, complete	EMPTY
Rho GTPase activating protein 1	BG468434
DNA2 (DNA replication helicase, yeast, h	BE085640
serine hydroxymethyltransferase 1 (solub	BE856375
similar to phosphatidylcholine transfer	AI827995
ESTs	AA464699
peptidyl prolyl isomerase H (cyclophilin	BF794821
ESTs	AI086307
ESTs	AA343510
hypothetical protein FLJ10474	AI820014
ESTs	BF798058
hypothetical protein FLJ20643	XM_012785
Homo sapiens mRNA for FLJ00116 protein,	AI989598
pituitary tumor-transforming 1 interacti	BE795643
acetylcholinesterase (YT blood group)	AI831696
putative acyltransferase	AL359403
Junk, low PCR	EMPTY
6-pyruvoyltetrahydropterin synthase	NM_000317
Empty	EMPTY
ceruloplasmin (ferroxidase)	AW950668
asialoglycoprotein receptor 1	NM_001671
ESTs, Moderately similar to AT2A_HUMAN P	AI018742
neuromedin U	BE932728
Junk, low PCR	EMPTY
histone acetyltransferase 1	BG178769
integrin cytoplasmic domain-associated p	BG032225
ESTs	AI273285
KIAA0318 protein	AB002316
KIAA0904 protein	AW305119
associated molecule with the SH3 domain	AI991435
ESTs	AA757616
keratin 7	BE736996
recombination activating gene 2	AW058148
polymerase (DNA directed), delta 3	D26018
ubiquilin 2	BF433926
solute carrier family 12 (sodium/potassi	NM_001046
Empty	EMPTY
MAX-interacting protein 1	NM_005962
guanylate cyclase 2D, membrane (retina-s	NM_000180
Homo sapiens, RIKEN cDNA 0610043B10 gene	AI340212
CUG triplet repeat, RNA-binding protein	BG029685
KIAA0227 protein	D86980
msh (Drosophila) homeo box homolog 2	D14970
enhancer of zeste (Drosophila) homolog 2	AA053521

ESTs	H02833
cyclin T1	NM_001240
ring finger protein 7	AA521231
protein kinase NYD-SP15	AI142574
KIAA1145 protein	AA515537
cyclin-dependent kinase 8	BE467537
alpha2,8-sialyltransferase	XM_008705
KIAA0625 protein	AI458143
ESTs	AA219230
major histocompatibility complex, class	AI494316
Empty	EMPTY
H2A histone family, member O	AI885852
glutamate receptor, ionotrophic, AMPA 3	NM_000828
serine protease inhibitor, Kazal type, 5	AW445207
general transcription factor IIH, polype	NM_001516
metallothionein 1L	BF031192
carnitine palmitoyltransferase I, liver	BC000185
MHC class I region ORF	NM_006674
annexin A13	NM_004306
ribonuclease H1	AL554334
ESTs	AA862426
ESTs	AA805635
DKFZP564I1922 protein	AA464691
kinesin 2 (60-70kD)	AW963252
HLA-G histocompatibility antigen, class	AI123699
Homo sapiens clone 23583 mRNA sequence	AI767486
KIAA0125 gene product	NM_014792
synaptosomal-associated protein, 23kD	AW956343
Empty	EMPTY
cytochrome c oxidase subunit VIII	AW009404
ribosomal protein S24	AV709655
Junk, low PCR	EMPTY
immunoglobulin mu binding protein 2	L24544
coagulation factor C (Limulus polyphemus	NM_004086
protein kinase, DNA-activated, catalytic	BE018389
POU domain, class 1, transcription facto	NM_000306
serine/threonine kinase 18	NM_014264
Junk, low PCR	EMPTY
ESTs, Highly similar to B56409 zinc fing	AW162204
ESTs	AA156906
protein kinase, cAMP-dependent, regulato	AY007115
No ID Incyte EST	EMPTY
ATPase, H+ transporting, lysosomal (vacu	AA715129
hypothetical protein MGC11271	NM_024323
KIAA0618 gene product	AI439625
dedicator of cyto-kinesis 2	D86964
Empty	EMPTY
multifunctional polypeptide similar to S	AI524157
pleiomorphic adenoma gene 1	NM_002655
EST	W92315
lymphocyte antigen 117	Y14768

nephrosis 1, congenital, Finnish type (n	NM_004646
ribosomal protein L13	BG489603
periplakin	NM_002705
brain-derived neurotrophic factor	X60201
Junk, low PCR	EMPTY
ESTs, Moderately similar to ALU1_HUMAN A	AA490232
Homo sapiens, Similar to RIKEN cDNA 2010	AA704113
KIAA0700 protein	AI018400
Junk, low PCR	EMPTY
KIAA0042 gene product	BE644769
PET112 (yeast homolog)-like	BF195982
transmembrane protease, serine 2	AF123453
protease, serine, 8 (prostasin)	NM_002773
Empty	EMPTY
calcium/calmodulin-dependent protein kin	AI052299
3-hydroxy-3-methylglutaryl-Coenzyme A re	AA602498
ESTs, Weakly similar to ALU6_HUMAN ALU S	AA071159
Sec23 (S. cerevisiae) homolog A	AV701894
arachidonate 12-lipoxygenase	AF143883
glycoprotein A repetitions predominant	NM_005512
small nuclear ribonucleoprotein polypept	F20803
Junk, low PCR	EMPTY
Junk, low PCR	EMPTY
KIAA0736 gene product	BE465640
solute carrier family 4, sodium bicarbon	AI453320
Junk, low PCR	EMPTY
FK506-binding protein 5	NM_004117
H2A histone family, member Z	AA948729
doublecortex; lissencephaly, X-linked (d	NM_000555
Junk, low PCR	EMPTY
ATP-binding cassette, sub-family C (CFTR	NM_000392
homeo box B6	AA610066
Junk, low PCR	EMPTY
hypothetical protein FLJ11342	NM_018394
hypothetical protein FLJ22569	BF594226
cell division cycle 2-like 5 (cholineste	NM_003718
ribosomal protein L7a	AV742224
ESTs, Weakly similar to O4HUD1 debrisoqu	AI582291
breast cancer 1, early onset	AU125312
major histocompatibility complex, class	M83664
KIAA0993 protein	AW338128
KIAA0532 protein	AB011104
Junk, low PCR	EMPTY
cocaine- and amphetamine-regulated trans	NM_004291
ATPase, H+ transporting, lysosomal (vacu	AI880007
protein kinase, AMP-activated, alpha 1 c	AW243792
hexosaminidase B (beta polypeptide)	BG478334
Not56 (D. melanogaster)-like protein	BF979931
inositol polyphosphate-4-phosphatase, ty	XM_003530
polymerase (DNA directed), beta	AL547658
heterogeneous nuclear protein similar to	BE537399

ESTs, Weakly similar to unnamed protein	AI816983
Junk, low PCR	EMPTY
Junk, low PCR	EMPTY
non-metastatic cells 1, protein (NM23A)	AL530970
KIAA0790 protein	AK025495
Junk, low PCR	EMPTY
KIAA1232 protein	AA477567
ESTs, Weakly similar to LKHU proteoglyca	AA632415
cullin 2	AF126404
Homo sapiens, Similar to RIKEN cDNA 2610	BF588577
cannabinoid receptor 2 (macrophage)	NM_001841
gamma-tubulin complex protein 2	AW835358
soc-2 (suppressor of clear, C.elegans) h	AF068920
sodium-dependent high-affinity dicarboxy	AL442082
oxytocin receptor	AI095591
matrix metalloproteinase 16 (membrane-in	AI192539
nucleoporin 153kD	NM_005124
HLA class II region expressed gene KE4	AL031228
HIV-1 Tat interactive protein, 60 kDa	AW162761
GTP-binding protein Rho7	AI864474
outer dense fibre of sperm tails 2	XM_011756
cathepsin E	AI598121
DKFZP434C212 protein	AW977288
zinc finger protein 261	NM_005096
ubiquinol-cytochrome c reductase hinge p	BE621639
ESTs, Weakly similar to unnamed protein	BF341082
Junk, low PCR	EMPTY
Junk, low PCR	EMPTY
hepatitis A virus cellular receptor 1	XM_011327
destrin (actin depolymerizing factor)	AL522332
coronin, actin-binding protein, 1A	BE252062
glutathione S-transferase M2 (muscle)	AW386100
chromosome 14 open reading frame 2	AV711844
S-phase kinase-associated protein 2 (p45	BF311153
Junk, low PCR	EMPTY
cyclin-dependent kinase 7 (homolog of Xe	BE887969
ESTs, Moderately similar to unknown [H.s	AI624501
pregnancy-induced growth inhibitor	AA581497
aldehyde dehydrogenase 3 family, member	BF679509
casein kinase 2, alpha 1 polypeptide	BE408670
hypothetical protein FLJ20208	NM_017712
ELK4, ETS-domain protein (SRF accessory	XM_010736
hematopoietic protein 1	BG429014
ferritin, heavy polypeptide 1	BF346319
glucosamine-6-phosphate isomerase	BE886578
Junk, low PCR	EMPTY
cadherin 6, type 2, K-cadherin (fetal ki	AU149929
prefoldin 5	AB055805
cadherin 1, type 1, E-cadherin (epitheli	AA569764
Junk, low PCR	EMPTY
ATPase, H+ transporting, lysosomal (vacu	BE252259

fusion, derived from t(12;16) malignant	AL549027
RD RNA-binding protein	NM_002904
gamma-aminobutyric acid (GABA) A recepto	NM_014211
hypothetical protein dJ310O13.3	AA453838
ESTs	N70665
electron-transfer-flavoprotein, alpha po	BE779126
guanine nucleotide exchange factor for R	W56891
solute carrier family 7 (cationic amino	AL553162
WW domain-containing oxidoreductase	AF211943
chromosome X open reading frame 12	AW162159
Homo sapiens cDNA: FLJ23546 fis, clone L	BE856915
myxovirus (influenza) resistance 2, homo	XM_012984
Junk, low PCR	EMPTY
clathrin, light polypeptide (Lcb)	BE791502
minichromosome maintenance deficient (S.	AU124962
DEAD/H (Asp-Glu-Ala-Asp/His) box polypep	AK001467
islet amyloid polypeptide	X68830
inhibitor of growth family, member 3	NM_019071
pre-B-cell leukemia transcription factor	NM_002585
aryl hydrocarbon receptor nuclear transl	NM_001668
ATPase, Class II, type 9B	AL096735
adaptor-related protein complex 1, mu 2	AA781244
No ID Incyte EST	EMPTY
peroxisome biogenesis factor 1	AL046043
epithelial V-like antigen 1	AF275945
Junk, low PCR	EMPTY
IKK-related kinase epsilon; inducible Ik	NM_014002
MAX dimerization protein	BF894861
hypothetical protein FLJ20551	AI378789
cyclin A1	NM_003914
KIAA0680 gene product	AA243866
ATP-binding cassette, sub-family C (CFTR	AB005659
GAP-associated tyrosine phosphoprotein p	BG032520
EST	AI273534
LIM domain kinase 2	AW292015
KIAA0663 gene product	W68201
sushi-repeat protein	NM_014467
Human AMP deaminase (AMPD2) mRNA	EMPTY
chemokine (C-C motif) receptor 1	XM_003248
leukocyte immunoglobulin-like receptor,	AF009007
hypothetical protein FLJ20604	AA934684
cyclin B1	AI972071
retinoid X receptor, beta	XM_011378
Junk, low PCR	EMPTY
trophinin associated protein (tastin)	NM_005480
hypothetical protein R32184_1	BE559812
hypothetical protein FLJ23293 similar to	AA481883
KIAA0450 gene product	AA757847
hypothetical protein FLJ14299	BF435432
ESTs, Weakly similar to ALU7_HUMAN ALU S	AA453747
Homo sapiens (clone 115392) mRNA	AL562009

Junk, low PCR	EMPTY
vasoactive intestinal peptide receptor 2	NM_003382
interferon-related developmental regulat	AU143090
H2B histone family, member Q	BF794197
Human lysosomal glycosylasparaginase (AG	EMPTY
signal recognition particle receptor ('d	BF037652
GTP-binding protein ragB	XM_010416
hypothetical protein FLJ22167	NM_024533
Junk, low PCR	EMPTY
low density lipoprotein-related protein-	BE730977
ESTs	AW593062
fyn-related kinase	NM_002031
dickkopf (Xenopus laevis) homolog 3	AB033421
vacuolar sorting protein 4	AL572725
pre-B-cell leukemia transcription factor	AW263437
ESTs, Weakly similar to ALU1_HUMAN ALU S	F32275
No ID Incyte EST	EMPTY
hypothetical protein FLJ20375	BF446957
KIAA0735 gene product; synaptic vesicle	NM_014848
sodium channel, nonvoltage-gated 1, beta	AI683977
caspase 7, apoptosis-related cysteine pr	U37449
hypothetical protein MGC4701	BC003648
Human ionizing radiation resistance conf	EMPTY
MpV17 transgene, murine homolog, glomeru	BG494133
Junk, low PCR	EMPTY
ESTs	W15284
cytochrome P450, 51 (lanosterol 14-alpha	NM_000786
salivary proline-rich protein	XM_003536
Homo sapiens mRNA; cDNA DKFZp434B195 (fr	BE018185
uridine phosphorylase	BG492119
sterol-C4-methyl oxidase-like	NM_006745
testis enhanced gene transcript (BAX inh	AW188221
HSPC135 protein	AA679577
ESTs	BF222926
ESTs	AW241505
ariadne (Drosophila) homolog, ubiquitin-	AL040708
eukaryotic translation elongation factor	BF688911
cyclin-dependent kinase 2	BF793700
Junk, low PCR, low PCR	EMPTY
adenosine monophosphate deaminase 1 (iso	NM_000036
Human mRNA for eukaryotic initiation fac	EMPTY
protein tyrosine phosphatase, receptor-t	AW950896
Junk, low PCR	EMPTY
ESTs	AA613805
collagen, type X, alpha 1 (Schmid metaph	X72579
proteasome (prosome, macropain) subunit,	AI262446
Homo sapiens complement-c1q tumor necros	AI916779
integrin beta 3 binding protein (beta3-e	AV743950
E2F transcription factor 3	AU153511
Junk, low PCR	EMPTY
NICE-5 protein	AI699906

pyruvate dehydrogenase phosphatase	AI265979
ESTs	AI806828
KIAA0157 protein	AK025991
amiloride-sensitive cation channel 1, ne	NM_001094
No ID Incyte EST	EMPTY
IMP (inosine monophosphate) dehydrogenas	XM_004627
muscle RAS oncogene homolog	NM_012219
Human glutamate receptor 2 (HBGR2) mRNA,	EMPTY
branched chain alpha-ketoacid dehydrogen	AL521642
mutS (E. coli) homolog 3	AI888396
ESTs, Weakly similar to T00375 hypotheti	AI284080
interferon-related developmental regulat	AL560304
voltage-dependent anion channel 2	BG178874
ESTs	BE858390
Junk, low PCR	EMPTY
KIAA0604 gene product	NM_014693
Homo sapiens mRNA for KIAA1741 protein,	W73588
tweety (Drosophila) homolog 1	AI337174
ESTs	BF439036
Junk, low PCR	EMPTY
methyl-CpG binding domain protein 4	AI634868
Homo sapiens clone 24425 mRNA sequence	Z44446
macrophage myristoylated alanine-rich C	NM_023009
protein kinase, cAMP-dependent, regulato	BG106229
tumor-associated calcium signal transduc	BE674290
Small nuclear ribonucleoprotein polypept	EMPTY
KIAA0381 protein	AW028055
hypothetical protein similar to beta-tra	AI186992
ESTs	BF433571
Junk, low PCR	EMPTY
protein kinase C, iota	AA827196
ESTs, Weakly similar to /prediction	AA970091
Junk, low PCR	EMPTY
retinol-binding protein 1, cellular	BE550120
Homo sapiens clone 25085 mRNA sequence	AF131754
hypothetical protein FLJ20628	AI168370
KIAA1377 protein	AB037798
6.2 kd protein	AF150733
short-chain dehydrogenase/reductase 1	AI858402
membrane protein of cholinergic synaptic	BF848246
RYK receptor-like tyrosine kinase	BG260940
KIAA0295 protein	AB002293
PWP2 (periodic tryptophan protein, yeast	AB001517
Arabadopsis8-31.25	EMPTY
Homo sapiens ryanodine receptor 2 (cardi	XM_001778
cell division cycle 42 (GTP-binding prot	AU146354
hypothetical protein FLJ20831	AK000838
zinc finger protein 142 (clone pHZ-49)	NM_005081
core-binding factor, beta subunit	NM_001755
REMOVED_FROM_DATABASE	EMPTY
Homo sapiens PNAS-13 mRNA, complete cds	AI492167

leiomodin 1 (smooth muscle)	NM_012134
microtubule-associated protein, RP/EB fa	AI885178
hypothetical protein FLJ10633	AI002410
leptin receptor	NM_002303
Homo sapiens mRNA for KIAA1758 protein,	AB051545
serologically defined colon cancer antig	NM_004713
sarcoglycan, alpha (50kD dystrophin-asso	AU139735
5-hydroxytryptamine (serotonin) receptor	Y12507
intracellular antigen detected by monocl	AK025144
Junk, low PCR	EMPTY
Arabadopsis10-31.25	EMPTY
Junk, low PCR	EMPTY
endothelin receptor type B	BE837728
cullin 4B	AW474599
chromobox homolog 1 (Drosophila HP1 beta	AL046741
synaptosomal-associated protein, 91 kDa	AF054993
Homo sapiens mRNA for KIAA1263 protein,	AW023860
hypothetical protein	AA828501
SH3-domain GRB2-like 2	XM_005469
solute carrier family 1 (high affinity a	NM_005071
ESTs	AW956016
phosphatidylinositol (4,5) bisphosphate	AC005005
KIAA1223 protein	AI139985
similar to calcium/calmodulin dependent	AL023754
cholinergic receptor, nicotinic, delta p	BF306695
KH-type splicing regulatory protein (FUS	BE251540
lamin A/C	H22169
plastin 1 (I isoform)	XM_011023
Empty	EMPTY
acyl-Coenzyme A oxidase 2, branched chai	BG545036
CD53 antigen	AW575081
NS1-binding protein	BE858805
Junk, low PCR	EMPTY
translin	AL533554
BCL2-associated athanogene 4	AI640615
inhibitor of kappa light polypeptide gen	N71526
CD68 antigen	BG252230
Homo sapiens similar to KIAA0166 gene pr	XM_012185
ESTs, Weakly similar to A46010 X-linked	AI952727
Junk, low PCR	EMPTY
activated RNA polymerase II transcriptio	AA477295
serine/threonine kinase with Dbl- and pl	XM_003027
proteasome (prosome, macropain) subunit,	AU126666
thymosin, beta, identified in neuroblast	BG531641
Rac/Cdc42 guanine exchange factor (GEF)	AW515372
progestin induced protein	AW968976
Empty	EMPTY
protein kinase C, alpha	NM_002737
ESTs	H48352
Homo sapiens mRNA; cDNA DKFZp434P116 (fr	AI003568
thyroid hormone receptor, beta (avian er	XM_002986

formyl peptide receptor 1	L10820
hypothetical protein FLJ20003	AI089360
hypothetical protein FLJ12949	AW192919
fatty-acid-Coenzyme A ligase, very long-	AL552895
REMOVED_FROM_DATABASE	Removed
vascular endothelial junction-associated	AI366088
phosphodiesterase 4D, cAMP-specific (dun	U02882
KIAA1552 protein	AA401452
copine VI (neuronal)	XM_007296
beta-2-microglobulin	AV710740
ATP synthase, H+ transporting, mitochond	BG030146
Junk, low PCR	EMPTY
v-rel avian reticuloendotheliosis viral	AL531207
Empty	EMPTY
KIAA0196 gene product	BE867293
ESTs	N24233
glucocorticoid receptor DNA binding fact	AL040592
protein tyrosine phosphatase, non-recept	NM_002830
activin A receptor, type I	NM_001105
ESTs	AA887547
Homo sapiens, clone IMAGE:3449838, mRNA,	T30778
KIAA0195 gene product	AF070545
homeo box A7	XM_011610
ESTs	AW449258
islet cell autoantigen 1 (69kD)	NM_004968
chromosome 21 open reading frame 18	AA984919
ClpX (caseinolytic protease X, E. coli)	AA678577
KIAA0614 protein	AB014514
Huntingtin interacting protein E	AI628601
F-box only protein 21	AV703995
ubiquitin specific protease 15	AF153604
Empty	EMPTY
zinc finger protein, subfamily 1A, 1 (Ik	NM_006060
Homo sapiens cDNA FLJ13618 fis, clone PL	BG502046
hypothetical protein IMPACT	AW167014
A kinase (PRKA) anchor protein 10	AA452319
tryptophan rich basic protein	NM_004627
eukaryotic translation initiation factor	AL031668
ESTs	AI041055
polymerase (RNA) II (DNA directed) polyp	BG105054
major histocompatibility complex, class	U83582
Junk, low PCR	EMPTY
Junk, low PCR	EMPTY
Junk, low PCR	EMPTY
Junk, low PCR	EMPTY
zeta-chain (TCR) associated protein kina	L05148
vinexin beta (SH3-containing adaptor mol	BG252405
inositol 1,4,5-trisphosphate 3-kinase B	NM_002221
KIAA0807 protein	AA808202
Human clone 23801 mRNA sequence	U79282
quinone oxidoreductase homolog	NM_004881

enhancer of filamentation 1 (cas-like do	R61386
platelet-activating factor acetylhydrola	L25107
Junk, low PCR	EMPTY
ESTs	AA707491
Junk, low PCR	EMPTY
platelet factor 4 variant 1	NM_002620
Junk, low PCR	EMPTY
ESTs, Weakly similar to ALUB_HUMAN !!!!	N42848
KIAA1183 protein	AI867302
ESTs	AA115553
KIAA0775 gene product	BE300065
ribosomal protein S3	AA593872
ATPase, H+/K+ exchanging, beta polypepti	NM_000705
oxygen regulated protein (150kD)	AI969119
PCTAIRE protein kinase 3	BC000281
tumor necrosis factor receptor superfami	XM_001743
spermidine/spermine N1-acetyltransferase	BF573292
Bcl-2 binding component 3	BG258126
MIL1 protein	AI144469
Junk, low PCR	EMPTY
KIAA1240 protein	AB033066
ESTs	AA041552
protamine 1	AL036195
proteasome (prosome, macropain) 26S subu	AA604188
epimorphin	NM_001980
Junk, low PCR	EMPTY
G5b protein	R69648
ESTs, Weakly similar to S28942 protein k	AW263657
KIAA0860 protein	AU148620
KIAA0789 gene product	XM_006610
KIAA0746 protein	AW503827
sorting nexin 3	BF082466
ATP synthase, H+ transporting, mitochond	NM_007100
gamma-aminobutyric acid (GABA) A recepto	XM_003519
calumenin	AI139684
methionine aminopeptidase; eIF-2-associa	AI184621
hypothetical protein MGC4614	AW969567
Junk, low PCR	EMPTY
major histocompatibility complex, class	BF732381
period (Drosophila) homolog 3	BF941635
thyrotropin-releasing hormone	NM_007117
retinoblastoma-binding protein 4	XM_001765
prospero-related homeobox 1	XM_001994
Homo sapiens mRNA; cDNA DKFZp762O1615 (f	W24346
HSPCO34 protein	AI280264
ESTs	AA399472
FOS-like antigen 2	NM_005253
splicing factor, arginine/serine-rich 1	AU130109
ESTs, Moderately similar to ALU1_HUMAN A	AW474244
ring finger protein (C3HC4 type) 8	BE894381
Homo sapiens clone TUA8 Cri-du-chat regi	AL544713

ATP synthase, H+ transporting, mitochond	BE739100
CD14 antigen	AL549182
nuclear receptor subfamily 1, group I, m	NM_005122
receptor (TNFRSF)-interacting serine-thr	BF436924
angiotensin I converting enzyme (peptidy	NM_000789
KIAA0033 protein	AA374991
hypothetical protein	R11669
transaldolase 1	BE538166
serine (or cysteine) proteinase inhibito	BF339409
solute carrier family 28 (sodium-coupled	NM_004212
ESTs, Weakly similar to RFXK_HUMAN DNA-B	AW024833
hypothetical protein MGC2601	BE796254
DKFZP586N1922 protein	AI379891
solute carrier family 1 (neuronal/epithe	AA243675
cold inducible RNA-binding protein	AI879285
testican 3	BC000460
No ID Incyte EST	EMPTY
lymphotoxin beta receptor (TNFR superfam	AI193089
cytochrome b5 outer mitochondrial membra	BF982660
solute carrier family 26, member 3	NM_000111
DnaJ (Hsp40) homolog, subfamily B, membe	NM_007034
DKFZP564B147 protein	AA872359
Homo sapiens clone 23664 and 23905 mRNA	AF035315
cytochrome P450, subfamily XIB (steroid	NM_000497
hypothetical protein FLJ10191	AW204828
KIAA0102 gene product	BG495280
casein kinase 2, beta polypeptide	BF338390
Junk, low PCR	EMPTY
ESTs	AA946869
hypothetical protein MGC4365	AI302370
ESTs	AA688021
estrogen-related receptor gamma	AB020639
Homo sapiens clone 24407 mRNA sequence	AF070575
ATPase, Class V, type 10B	AK025290
potassium channel, subfamily K, member 5	AK001897
diazepam binding inhibitor (GABA recepto	AA830695
carbonyl reductase 1	BE276411
bone morphogenetic protein 6	AA426586
glial fibrillary acidic protein	AA059335
Junk, low PCR	EMPTY
U5 snRNP-specific protein, 116 kD	BG419967
Cbp/p300-interacting transactivator, wit	BF110899
ring finger protein 14	AV717404
exostoses (multiple)-like 1	NM_004455
Junk, low PCR	EMPTY
ankyrin 1, erythrocytic	NM_000037
KIAA0451 gene product	NM_014826
EST	N47785
DKFZP434N093 protein	BG392388
protein geranylgeranyltransferase type I	AA481712
small inducible cytokine A4 (homologous	AV758471

No ID Incyte EST	EMPTY
Human DNA sequence from clone 1170K4 on	AL022314
interleukin 1 receptor, type I	NM_000877
hypothetical protein MGC10471	X13956
H.sapiens mRNA for ribosomal protein L26	EMPTY
translocase of inner mitochondrial membr	AV702166
major histocompatibility complex, class	M20503
transmembrane 4 superfamily member 4	AI982840
guanidinoacetate N-methyltransferase	NM_000156
ESTs	AW264687
activin A receptor, type II	NM_001616
hexokinase 1	BF311786
ESTs	H73202
ESTs	BF508832
hypothetical protein SP192	NM_021639
Junk, low PCR	EMPTY
ESTs	AA555042
chitinase 3-like 2	NM_004000
growth hormone receptor	NM_000163
protein kinase domains containing protei	BG387820
retinoic acid receptor responder (tazaro	AI027581
death associated protein 3	AA207194
Human cytochrome bc-1 complex core prote	EMPTY
calpain 3, (p94)	BE302598
potassium voltage-gated channel, subfami	NM_002236
laminin, alpha 3 (nicein (150kD), kalini	NM_000227
ATP-binding cassette, sub-family G (WHIT	AF038175
hypothetical protein MGC2217	BE876967
integrin, alpha 8	L36531
small inducible cytokine subfamily C, me	AL031736
ESTs, Moderately similar to ALU7_HUMAN A	AI565168
haemopoietic progenitor homeobox	XM_005768
centaurin-alpha 2 protein	AI167962
ESTs, Weakly similar to T46916 hypotheti	AW051955
ESTs	AA975489
cytochrome P450, subfamily IIE (ethanol-	AL532220
glutamate receptor, metabotropic 4	NM_000841
expressed in activated T/LAK lymphocytes	XM_008619
M-phase phosphoprotein 10 (U3 small nucl	X98494
complement component 1, r subcomponent	M14058
Human liver mRNA fragment DNA binding pr	EMPTY
NS1-associated protein 1	BE408656
HNK-1 sulfotransferase	AW956276
ladinin 1	XM_001914
killer cell lectin-like receptor subfami	AF078550
hypothetical protein FLJ11196	AW102946
solute carrier family 16 (monocarboxylic	NM_004694
fatty acid binding protein 1, liver	M10050
ESTs	AI028661
ESTs	AA625304
Junk, low PCR	EMPTY

hypothetical protein EDAG-1	AI133032
ESTs	AA146968
interferon, gamma-inducible protein 30	AL561631
endoplasmic reticulum glycoprotein	NM_006816
KIAA0907 protein	NM_014949
tetraspan 5	AF065389
actinin, alpha 1	AU118989
Spermidine/spermine N1-acetyltransferase	EMPTY
speckle-type POZ protein	NM_003563
integrin, alpha X (antigen CD11C (p150),	NM_000887
Homo sapiens clone FBD3 Cri-du-chat crit	AL080234
benzodiazapine receptor (peripheral)	BG289525
flotillin 1	AA293676
KIAA0329 gene product	NM_014844
ADP-ribosylation factor-like 2	NM_001667
ESTs	AA179167
hypothetical protein FLJ13902	AU159482
sorting nexin 6	BG035178
KIAA1272 protein	BE349987
Homo sapiens cDNA FLJ20796 fis, clone CO	AK000803
ATP/GTP-binding protein	AA188236
phosphoinositide-3-kinase, class 2, gamm	AJ000008
zinc finger protein	BC002580
G protein-coupled receptor 56	BF593277
activating transcription factor 2	NM_001880
Homo sapiens protein tyrosine kinase (Sy	EMPTY
general transcription factor II, i	BG259774
RNA binding motif protein 6	AL036332
singed (Drosophila)-like (sea urchin fas	BE298451
ligase I, DNA, ATP-dependent	XM_009118
ubiquinol-cytochrome c reductase complex	AV737698
ubiquitin-like 4	AL522146
serine dehydratase	R42895
anaphase-promoting complex subunit 10	AA262889
ESTs	AW084810
chromosome 21 open reading frame 18	AB004848
ESTs	AW973337
ESTs	BG537484
transcription elongation factor A (SII),	AU139477
dynein, axonemal, heavy polypeptide 9	AF257737
signal regulatory protein, beta, 1	AL049634
retinal G protein coupled receptor	NM_002921
nuclear receptor subfamily 3, group C, m	NM_000901
Human mRNA for histidyl-tRNA synthetase	EMPTY
KIAA0135 protein	U79240
hemoglobin, delta	NM_000519
Conserved gene telomeric to alpha globin	AI745503
retinal pigment epithelium-specific prot	NM_000329
Homo sapiens cDNA FLJ13681 fis, clone PL	AA922376
Epstein-Barr virus induced gene 3	AL545121
insulin-like growth factor binding prote	AA451821

ESTs	AI831847
matrix metalloproteinase 14 (membrane-in	AI052526
staufen (Drosophila, RNA-binding protein	AI147913
putative RNA binding protein	AW975823
Homo sapiens mRNA; cDNA DKFZp564H1916 (f	W80824
calcium/calmodulin-dependent protein kin	L41816
solute carrier family 10 (sodium/bile ac	NM_003049
KIAA0637 gene product	AW162147
adenosine A2a receptor	NM_000675
progestagen-associated endometrial prote	M61886
Arabadopsis2-31.25	EMPTY
origin recognition complex, subunit 3 (y	AF125507
deleted in bladder cancer chromosome reg	NM_014618
ESTs	AA993566
thyroid stimulating hormone, beta	AV752295
mannosidase, alpha, class 1A, member 2	NM_006699
TRAF family member-associated NFKB activ	BF439429
reelin	NM_005045
ESTs	AA928141
adrenergic, beta-3-, receptor	AI276134
hypothetical protein FLJ10300	AW468854
Junk, low PCR	EMPTY
TAK1-binding protein 2; KIAA0733 protein	BG110734
KIAA0774 protein	AB018317
No ID Incyte EST	EMPTY
inhibitor of growth 1 family, member 1	AF181850
EGF-containing fibulin-like extracellula	AW138268
homeo box (expressed in ES cells) 1	BF001626
Arabadopsis4-31.25	EMPTY
inhibitor of DNA binding 2, dominant neg	AI798557
glycoprotein Ib (platelet), alpha polype	NM_000173
ESTs	AW592246
E2F transcription factor 1	AL121906
Junk, low PCR	EMPTY
histidine ammonia-lyase	NM_002108
CD3-epsilon-associated protein; antisens	NM_012099
ESTs	AW468728
KIAA0007 protein	AK000836
ESTs, Weakly similar to hiwi [H.sapiens]	AA503085
tRNA isopentenylpyrophosphate transferas	AI347522
ESTs	BE891602
KIAA0913 protein	AW273142
Human (clone CTG-A4) mRNA sequence	AL530945
arfaptin 1	AU129957
Nedd-4-like ubiquitin-protein ligase	U96114
mitogen-activated protein kinase 14	NM_001315
Arabadopsis6-31.25	EMPTY
nuclear receptor coactivator 4	AW467396
Junk, low PCR	EMPTY
ESTs	AW665447
low density lipoprotein receptor (famili	AW006976

Junk, low PCR	EMPTY
KIAA0514 gene product	NM_014696
cerebellar degeneration-related protein	NM_004065
ESTs	N26569
myosin VA (heavy polypeptide 12, myoxin)	NM_000259
fetal Alzheimer antigen	AA169449
KIAA0615 gene product	BF436126
ESTs	AA827805
Homer, neuronal immediate early gene, 1B	AW376142
KIAA0802 protein	AB018345
high-mobility group protein 2-like 1	AL079310
chimerin (chimaerin) 2	NM_004067
chymotrypsin-like	NM_001907
Empty	EMPTY
granulin	BG253285
RAD52 (S. cerevisiae) homolog	U12134
KIAA0495	BE350299
predicted osteoblast protein	AI491952
seladin-1	BE253839
Junk, low PCR	EMPTY
gamma-aminobutyric acid (GABA) A recepto	AF165124
myeloid/lymphoid or mixed-lineage leukem	XM_006779
Junk, low PCR	EMPTY
ESTs	AA157506
hypothetical protein FLJ22457	AI767412
Junk, low PCR	EMPTY
glucosaminyl (N-acetyl) transferase 2, I	NM_001491
KIAA0624 protein	AI193238
growth arrest-specific 2	BE550832
profilin 2	AV724105
CDC6 (cell division cycle 6, S. cerevisi	BE907412
Empty	EMPTY
mitogen-activated protein kinase 6	BE883563
karyopherin alpha 4 (importin alpha 3)	BE393532
ESTs, Highly similar to S66292 actin-cro	AI017174
GRB2-associated binding protein 2	NM_012296
amyloid beta (A4) precursor-like protein	AU125698
Wilms tumor associated protein	BC002734
REMOVED_FROM_DATABASE	EMPTY
glutamate receptor, metabotropic 1	AL035698
Junk, low PCR	EMPTY
RAN binding protein 17	BE501432
frequenin (Drosophila) homolog	AI589810
Homo sapiens cDNA: FLJ22042 fis, clone H	AA496402
RNA helicase-related protein	BG165945
proline-rich protein with nuclear target	BG435213
LIM domain only 6	AI017508
tumor necrosis factor receptor superfami	NM_002546
SREBP CLEAVAGE-ACTIVATING PROTEIN	AL040564
Empty	EMPTY
CD69 antigen (p60, early T-cell activati	NM_001781

lymphocyte-specific protein 1	AI131342
stromal antigen 1	AI139660
Junk, low PCR	EMPTY
interleukin 9 receptor	NM_002186
deleted in lymphocytic leukemia, 1	AA479324
Junk, low PCR	EMPTY
mab-21 (C. elegans)-like 1	NM_005584
Junk, low PCR	EMPTY
hypothetical protein FLJ12612 similar to	BF939657
ESTs	W37319
hypothetical protein FLJ11457	AI091098
polymerase (DNA directed), delta 3	D26018
tubby (mouse) homolog	NM_003320
hypothetical protein DKFZp564F013	AA215791
Junk, low PCR	EMPTY
phosphofructokinase, platelet	BE378739
