# Supplementary material for: Early indicators of exposure to biological threat agents using host gene profiles in peripheral blood mononuclear cells
Source: BMC Infect Dis. 2008 Jul 30;8:104. doi: 10.1186/1471-2334-8-104 (PMC2542375; doi:10.1186/1471-2334-8-104)
Supplement: Additional file 3 — List of the first 50 genes that were selected using the Grow/Shrink method. This table lists the genes that passed the statistical analysis using the Grow/Shrink method. [file 1471-2334-8-104-S3.pdf]

Gene Name	Gene ID
interferon gamma precursor (IFN-gamma; IFNG); immune inte	X01992; M29383
myeloblastin precursor (MBN); leukocyte proteinase 3 (PRTN3	M29142
cell surface glycoprotein MUC18; melanoma-associated antige	M28882
kidney glomeruli chloride channel; ClC-5	X91906
ephrin type-A receptor 2 precursor; epithelial cell kinase (ECK)	M59371 M36395
selenium-binding protein	U29091
complement component 5 (C5)	M65134
inhibin alpha subunit precursor (INHA)	M13981
14-3-3 protein beta/alpha; protein kinase C inhibitor protein-1 (	X57346
YL-1 protein	D43642
apolipoprotein E precursor (APOE)	M12529
Rad50	U63139
NADH-ubiquinone oxidoreductase B18 subunit; complex I-B18	M33374
tristetraproline (TTP); TIS11; ZFP36; growth factor-inducible n	M92843
matrix metalloproteinase 11 (MMP11); stromelysin 3	X57766
TRF1-interacting ankyrin-related ADP-ribose polymerase tanky	AF082556
neuronal acetylcholine receptor protein beta 4 subunit precurs	U62439
voltage-gated potassium channel	Y15065
photolyase/blue-light receptor homolog	D84657
angiotensin-converting enzyme (ACE)	A00914
erythropoietin receptor (EPOR)	M60459
acidic fibroblast growth factor (AFGF) + heparin-binding growth	X65778 + X51943 + M13361
macMARCKS; MARCKS-related protein (MRP); MLP	X70326
cyclic-AMP-dependent transcription factor atr-1; TREB36 prote	X55544
transcription factor ZFM1	D26120
PCAF-associated factor 65 alpha	AF069735
defensin 6 precursor	M98331
CXC chemokine precursor	AJ002211
fasL receptor; apoptosis-mediating surface antigen fas; APO-1	M67454
pancreatitis-associated protein 1 precursor	D13510
cyclin-dependent kinase 4 inhibitor B (CDKN2B); p14-INK4B;	U17075; L36844
vascular endothelial growth factor C precursor (VEGF-C); vasc	U43142
sodium- & chloride-dependent taurine transporter	Z18956
paraneoplastic encephalomyelitis antigen HUD; HU-antigen D	M62843
glutathione S-transferase mu1 (GSTM1; GST1); HB subunit 4;	X68676; S01719
homeobox protein HOX-D3; HOX-4A	D11117
interleukin-3 precursor (IL-3); multipotential colony-stimulating	M14743; M17115
melanotransferrin precursor; melanoma-associated antigen p9	M12154
epithelial discoidin domain receptor 1 precursor (EDDR1; DDR	X74979
cadherin 8 (CDH8)	L34060
lnk adaptor protein	AF055581
lactotransferrin precursor; lactoferrin	X53961
thiosulfate sulfurtransferase; rhodanese	D87292
endothelin 2 (ET2)	M65199
protein-tyrosine phosphatase 1E	L34583
dimethylaniline monooxygenase (N-oxide forming) 1 (EC 1.14.	M64082
tissue inhibitor of metalloproteinases 2 (TIMP2); metalloprotein	J05593
LYL-1 protein	M22637
myelin-associated glycoprotein precursor (MAG)	M29273
