# Supplementary material for: Early indicators of exposure to biological threat agents using host gene profiles in peripheral blood mononuclear cells
Source: BMC Infect Dis. 2008 Jul 30;8:104. doi: 10.1186/1471-2334-8-104 (PMC2542375; doi:10.1186/1471-2334-8-104)
Supplement: Additional file 4 — List of the accession numbers and sequences of the primer sets used in this study. [file 1471-2334-8-104-S4.pdf]

Gene Name	Full Name	GenBank	Sequence
GBP	guanine-nucleotide binding protein	M36429	F-GACTCAAGGCTGCTGGTCA
			R-AAGCGGCAACACGACAGGTA
HIF1	hypoxia-inducible factor-1 alpha	U22431	F-AATGATGTAATGCTCCCC
			R-TTCTATGACTCCTTTTCCTG
18s	18s ribosomal protein	M10098	F-AATTGACGGAAGGGCACCAC
			R-CGGACATCTAAGGGCATCACAG
C5AR	C5 anaphylatoxin receptor	M62505	F-CCACGCGGTCCACCAAGACACTCAAGG
			R-GTGGCCCATGAGGCTGTCGCCTAC

Product Size
257
741
316
373
