# Supplementary material for: Early indicators of exposure to biological threat agents using host gene profiles in peripheral blood mononuclear cells
Source: BMC Infect Dis. 2008 Jul 30;8:104. doi: 10.1186/1471-2334-8-104 (PMC2542375; doi:10.1186/1471-2334-8-104)
Supplement: Additional file 5 — Lists the genes of the Clontech 1.2 human array. [file 1471-2334-8-104-S5.pdf]

Protein/gene	Accession number
Von Hippel-Lindau tumor suppressor protein (VHL)	L15409
cadherin1 (CDH1); epithelial cadherin precursor (E-cadher	Z13009
LUCA2; lysosomal hyaluronidase 2 (HYAL2) ; PH-20 homo	U09577
N-myc proto-oncogene	M13228
B-raf proto-oncogene (RAFB1)	M95712
vascular endothelial growth factor receptor 1 (VEGFR1); ty	X51602 + U01134
transforming protein rhoA H12 (RHO12; ARH12; ARHA)	L25080
G2/mitotic-specific cyclin A (CCNA; CCN1)	X51688
BUBR1 protein kinase	AF046079
wee1Hu CDK tyrosine 15-kinase; wee-1-like protein kinase	U10564
aurora-related kinase 1 (ARK1)	D84212
CDC25B; CDC25HU2; M-phase inducer phosphatase 2	M81934; S78187
transmembrane 4 superfamily protein; SAS	U01160
calcium-activated potassium channel beta subunit; maxi K	U25138
mothers against dpp homolog 4 (SMAD4); MADR4; pancre	U44378
ras-related protein RAP-1A; C21KG; KREV-1 protein; GTF	M22995
LUCA15 putative tumor suppressor	U23946
erythroblastosis virus oncogene homolog 1 (ETS-1); p54	J04101
pim-1 proto-oncogene	M54915
tyrosine-protein kinase receptor tyro3 precursor; rse; sky; c	D17517
transforming protein p21/K-ras 2B	M54968
G2/mitotic-specific cyclin B1 (CCNB1)	M25753
cell division control protein 2 homolog (CDC2); p34 protein	X05360
serine/threonine-protein kinase PLK1 (STPK13)	U01038
aurora- & IPL1-like midbody-associated protein kinase 1 (A	AF008552
CDC25C; M-phase inducer phosphatase 3	M34065
C-1	U41816
G protein-activated inward rectifier potassium channel 1 (G	U39196
adenomatous polyposis coli protein (APC protein); DP2.5	M74088; M73548
EB1 protein	U24166
neogenin	U61262
MAD protein; MAX dimerizer	L06895
c-raf proto-oncogene	X03484
c-ros-1 tyrosine-protein kinase proto-oncogene	M34353
N-ras; transforming p21 protein	X02751
G1/S-specific cyclin D1 (CCND1); cyclin PRAD1; bcl-1 onc	X59798; M64349
cyclin-dependent protein kinase 2 (CDK2); p33 protein kina	M68520
cell division protein kinase 9 (CDK9); serine/threonine prot	L25676
cyclin-dependent kinase 4 inhibitor B (CDKN2B); p14-INK4	U17075; L36844
prothymosin alpha (ProT-alpha; PTMA)	M26708
cyclin-D binding Myb-like protein (hDMP1)	AF084530
G protein-activated inward rectifier potassium channel 2 (G	U24660
breast cancer type 2 susceptibility protein (BRCA2)	U43746
ezrin; cytovillin 2; villin 2 (VIL2)	X51521
transforming growth factor-beta signaling protein 1 (BSP1)	U57456
jun-D	X56681
A-raf proto-oncogene serine/threonine-protein kinase; PKS	L24038
proto-oncogene tyrosine-protein kinase abl; p150; c-abl	M14752; M14753; M14754
C-cbl proto-oncogene	X57110

G1/S-specific cyclin D2 (CCND2) + KIAK0002	M90813 + D13639
cell division protein kinase 4; cyclin-dependent kinase 4 (C	M14505
stem cell tyrosine kinase 1 (STK1); FL cytokine receptor pı	U02687
cyclin-dependent kinase 4 inhibitor (CDK4I; CDKN2); p16-	L27211
DNA-binding protein inhibitor ID-1; Id-1H	D13889
water channel aquaporin 3 (AQP3)	AB001325
ASIC3 proton gated cation channel	AB010575
tumor suppressor protein DCC precursor; colorectal cance	X76132
transforming growth factor-beta 3 (TGF-beta3)	J03241
p78 putative serine/threonine-protein kinase	M80359
B-myb	X13293
tyrosine-protein kinase receptor UFO precursor; axl oncog	M76125
tyrosine-protein kinase ABL2; tyrosine kinase ARG (ABLL)	M35296
INT-2 proto-oncogene protein precursor (fibroblast growth	M17446
G1/S-specific cyclin D3 (CCND3)	M92287
cell division protein kinase 6 (CDK6); serine/threonine prot	X66365
serine/threonine-protein kinase KKIALRE	X66358
cyclin-dependent kinase 4 inhibitor D (CDKN2D); p19-INK4	U40343; U20498
transcription factor DP2 (Humdp2); E2F dimerization partn	U18422
sulfate transporter; diastrophic dysplasia protein	U14528
G protein-activated inward rectifier potassium channel 3 (G	U52152
p53-associated mdm2 protein	Z12020; M92424
transforming growth factor beta receptor III precursor (TGF	L07594
C-maf transcription factor	AF055377
fos-related antigen 2 (FRA2)	X16706
macrophage colony stimulating factor I receptor precursor	X03663
C-src proto-oncogene (SRC1)	K03214; X03996
mas proto-oncogene	M13150
G1/S-specific cyclin E (CCNE)	M73812
cell division protein kinase 5 (CDK5); tau protein kinase II (	X66364
CDC2-related protein kinase CHED	M80629
cyclin-dependent kinase inhibitor 1C (CDKN1C); p57-KIP2	U22398
helix-loop-helix protein HLH 1R21; DNA-binding protein inl	X69111
erythrocyte glucose transporter 1 (GLUT1)	K03195
ATP-sensitive inward rectifier potassium channel 8 ; UKAT	D50312
neurofibromatosis protein type I (NF1); neurofibromin	M60915
prohibitin (PHB)	S85655; U17179
elk-1; ets-related proto-oncogene	M25269
fos-related antigen (FRA1)	X16707
c-kit proto-oncogene; mast/stem cell growth factor recepto	X06182
C-yes proto-oncogene (YES1)	M15990
thrombopoietin receptor precursor (TPOR); myeloproliferat	U68162
G2/mitotic-specific cyclin G1 (CCNG1; CYCG1)	U47413
protein serine/threonine kinase STK1; cell division protein	L20320
p35 cyclin-like CAK1-associated protein	X92669
cyclin-dependent kinase inhibitor 1 (CDKN1A); melanoma	U09579; L25610
40S ribosomal protein S19 (RPS19)	M81757
liver glucose transporter 2	J03810
calcium-activated potassium channel HSK1	U69883
moesin-ezrin-radixin-like protein (MERLIN); schwannomin	L11353; Z22664; X72657; L27133
tight junction protein zonula occludens (ZO-1); tight junctio	L14837

A-myb proto-oncogene; myb-related protein A	X66087
v-erbA related protein (EAR2)	X12794
met proto-oncogene; hepatocyte growth factor receptor pre	J02958
C-fes proto-oncogene	X52192
cell surface glycoprotein MUC18; melanoma-associated ar	M28882
cyclin H (CCNH); MO15-associated protein	U11791; U12685
extracellular signal-regulated kinase 1 (ERK1; p44-ERK1);	X60188
cyclin G-associated kinase (GAK)	D88435
cyclin-dependent kinase inhibitor 3 (CDKN3); CDK2-assoc	L25876
bullous pemphigoid antigen 1 (BPAG1; BPA); hemidesmos	M63618
brain glucose transporter 3 (GTR3)	M20681
chloride conductance regulatory protein ICLN; nucleotide-s	X91788
retinoblastoma-like protein 2 (RBL2; RB2); 130-kDa retinol	X74594
c-myc purine-binding transcription factor puf; nucleoside di	L16785 + M36981
c-fos proto-oncogene; G0S7 protein	K00650
ets-related protein tel; ets translocation variant 6 (ETV6)	U11732
papillary thyroid carcinoma-encoded protein + ret proto-on	M31213 + M57464
C-fgr proto-oncogene (p55-FGR); SRC2	M19722
insulin-like growth factor binding protein 2 (IGFBP2)	M35410
fte-1; yeast mitochondrial protein import homolog; 40S ribo	M77234
extracellular signal-regulated kinase 2 (ERK2); mitogen-ac	M84489
serine/threonine-protein kinase NEK3; NIMA-related protei	Z29067
ubiquitin-conjugating enzyme E2 H10; ubiquitin-protein liga	U73379
proliferating cell nucleolar antigen P120; NOL1	X55504
E16 amino acid transporter	AF077866
voltage-gated potassium channel protein KV12; HUKIV; HI	L02752
p53 cellular tumor antigen	M14694; M14695
nucleoside diphosphate kinase A (NDKA); NDP kinase A; t	X17620
c-jun proto-oncogene; transcription factor AP-1	J04111
triiodothyronine receptor; thyroid hormone receptor (THRA	M24898
epidermal growth factor receptor (EGFR)	X00588; K03193; X00663; U48722
shb proto-oncogene	X75342
T-lymphoma invasion and metastasis inducing TIAM1	U16296
cation-independent mannose-6-phosphate receptor precur	Y00285; J03528
extracellular signal-regulated kinase 3 (ERK3); MAP kinas	X80692
CDC-like kinase 2 (CLK2)	L29216
geminin	AF067855
NuMA	Z11583
aquaporin 4; WCH4; mercurial-insensitive water channel (I	U34846
voltage-gated potassium channel protein KV11; HUKI; HBI	L02750
retinoblastoma-associated protein (RB1); PP110; P105-RE	M15400
TSG101 tumor susceptibility protein	U82130
myb proto-oncogene; c-myb	M15024
v-erbA related protein (EAR3); COUP transcription factor (	X12795; X16155; X58241
ERBB2 receptor protein-tyrosine kinase; neu proto-oncoge	M95667 + M11730
ski oncogene	X15218
matrix metalloproteinase 11 (MMP11); stromelysin 3	X57766
cyclin A1 (CCNA1)	U66838
extracellular signal-regulated kinase 4 (ERK4); MAP kinas	X59727
CDC-like kinase 3 (CLK3)	L29220
katanin p80 subunit	AF052432

myeloid cell nuclear differentiation antigen (MNDA)	M81750
aquaporin 9	AB008775
voltage-gated potassium channel protein KV14; HUKII; HB	M55514
Wilms' tumor protein (WT33; WT1)	X51630
maguk p55 subfamily member 2; MPP2 protein; discs large	X82895
c-myc oncogene	V00568
ETS oncogene (PEP1)	L16464
ERBB-3 receptor protein-tyrosine kinase precursor; epider	M29366; M34309
snoN oncogene	X15219
cyclin T CDK9-associated	AF045161
cyclin G2 (CCNG2)	U47414; L49506
extracellular signal-regulated kinase 5 (ERK5); BMK1 kina	U25278
serum-inducible kinase (SNK)	AF059617
diaphanous 1 (HDIA1)	AF051782
transducer of erbB2 (TOB)	D38305
cationic amino acid transporter 3	AJ000730
N-type calcium channel alpha-1B subunit; omega-conotoxi	M94172
putative protein-tyrosine phosphatase PTEN; mutated in m	U92436
tumor suppressor maspin; protease inhibitor 5 (PI5)	U04313
c-rel proto-oncogene protein	X75042
cot proto-oncogene	D14497
ERBB4 receptor protein-tyrosine kinase; Her4 tyrosine kina	L07868
CBL-B	U26710
cyclin K	AF060515
bub1 mitotic checkpoint kinase	AF053305
cdc2-related protein kinase PISSLRE	L33264
cyclin-dependent kinase regulatory subunit 1 (CKS1)	X54941
sprouty 2 (SPRY2)	AF039843
p55CDC	U05340
putative renal organic anion transporter 1	AF057039
dihydropyridine-sensitive L-type calcium channel beta-3 su	U07139
colorectal mutant cancer protein (MCC)	M62397
tumor suppressor LUCA1; hyaluronoglucosaminidase (HYA	U03056
L-myc proto-oncogene (MYCL1)	M19720
C-mos proto-oncogene serine/threonine-protein kinase	J00119
platelet-derived growth factor receptor alpha subunit (PDG	M21574
H-ras proto-oncogene; transforming G protein	V00574
cyclin E2	AF091433
serine/threonine-protein kinase NEK2; NIMA-related protei	U11050
CDC-like kinase 1 (CLK1)	L29222
cyclin-dependent kinase regulatory subunit (CKS2)	X54942
cell division cycle protein 25A (CDC25A); M-phase inducer	M81933
RCL growth-related c-myc-responsive gene	AF040105
erythrocyte urea transporter (UTE; UT1); SLC14A1; HUT1	U35735
neuronal low-voltage-activated T-type calcium channel alp	AF029228; AF029229
kidney glomeruli chloride channel; ClC-5	X91906
monocarboxylate transporter 1 (MCT1)	L31801
zinc transporter 4	AF025409
macrophage-stimulating protein receptor precursor (MSP r	X70040
urokinase-type plasminogen activator receptor GPI-anchor	U08839; M83246; X51675
related to receptor tyrosine kinase (RYK)	S59184

proto-oncogene tyrosine-protein kinase lck; p56-lck; lymph	U07236
glycogen synthase kinase 3 beta (GSK3 beta); tau kinase	L33801
dual-specificity mitogen-activated protein kinase kinase 6 (	U39657
cAMP-dependent protein kinase type II alpha regulatory su	X14968
lipid-activated protein kinase PRK1;PKN cell morphology-r	U33053
serine/threonin-protein kinase PAK-beta; p21-activated kin	AF068864
phospholipase C-delta-1 (PLC-delta-1; PLCD1); 1-phospha	U09117
ADP-ribosylation factor 1	M36340
cardiac muscle sodium channel alpha subunit; HH1	M77235
sodium/hydrogen exchanger 1 (Na+/H+ exchanger 1; NHE	M81768
Golgi 4-transmembrane spanning transporter; MTP	D14696
autocrine motility factor receptor (AMF receptor; AMFR)	L35233
vascular endothelial growth factor receptor 2 precursor (VE	L04947; X61656
protein-tyrosine kinase transmembrane receptor ror1	M97675
tyrosine-protein kinase lyn	M16038
pyruvate dehydrogenase kinase kinase precursor	L42450
MAPK/ERK kinase kinase 3 (MEK kinase 3; MEKK3)	U78876
Janus kinase 1 (JAK1)	M35203
serum- & glucocorticoid-regulated serine/threonine protein	AJ000512
myotonic dystrophy protein kinase-like protein	Y12337
phosphatidylinositol 3-kinase catalytic subunit delta isoform	U86453
ras-related protein RAP-1B; GTP-binding protein SMG p21	X08004
KCNQ3 potassium channel	AF033347
sodium/hydrogen exchanger 3 (Na+/H+ exchanger 3; NHE	U28043
organic cation transporter 1	U77086
colon carcinoma kinase 4 precursor (CCK4) + transmembr	U33635 + U40271
angiopoietin 1 receptor precursor; tyrosine-protein kinase r	L06139
neurotrophic tyrosine kinase receptor-related 3; TKT precu	X74764
integrin-linked kinase (ILK)	U40282
ribosomal protein kinase B (RSKB)	AJ010119
protein kinase C alpha polypeptide (PKC-alpha; PKCA)	M22199
janus kinase 3 (JAK3); leukocyte janus kinase (L-JAK)	U09607
serine/threonine-protein kinase NRK2; serine/threonine kir	L20321
ribosomal protein S6 kinase II alpha 1 (S6KII-alpha 1); ribo	L07597
phosphatidylinositol 3-kinase regulatory beta subunit (PI3-I	X80907
ras-related protein RAB2	M28213
voltage-gated potassium channel	Y15065
small intestine oligopeptide transporter; peptide transporte	U13173
apolipoprotein E precursor (APOE)	M12529
T-lymphocyte activation CD86 antigen precursor; activation	L25259
high-affinity nerve growth factor receptor precursor; trk-1 tr	X03541
tyrosine kinase receptor tie-1 precursor	X60957; S89716
epidermal growth factor receptor substrate 15 (EPS15); AF	U07707; Z29064
tyrosine-protein kinase ack	L13738
protein kinase C beta I (PKC-beta-1)	M27545; X06318
c-jun N-terminal kinase 1 (JNK1); JNK46	L26318
protein kinase MLK-3; sprk	L32976
ribosomal protein S6 kinase II alpha 2 (S6KII-alpha 2); ribo	X85106
68-kDa type I phosphatidylinositol-4-phosphate 5-kinase a	U78576
ras-related protein RAB3B	M28214
sodium- & chloride-dependent glycine transporter 1 (GLYT	S70609

high-affinity glutamate transporter; excitatory amino acid tr	U03506
cholesteryl ester transfer protein precursor (CETP); lipid tra	M30185
CC chemokine receptor type 1 (CC CKR1; CCR 1); macro	D10925
brain-derived neurotrophic factor (BDNF)/NT-3 growth fact	U12140
epithelial discoidin domain receptor 1 precursor (EDDR1; [	X74979
cell division cycle protein 25 nucleotide exchange factor (C	M91815; L26584
tyk2 non-receptor protein tyrosine kinase	X54637
protein kinase C delta (NPKC-delta)	D10495
c-jun N-terminal kinase 2 (JNK2); JNK55	L31951
tyrosine kinase tnk1	U43408
ribosomal protein S6 kinase II alpha 3 (S6KII-alpha 3); ribo	U08316
phospholipase C (PLCL)	D42108
ras-related protein RAB4A	M28211
sodium-dependent dopamine transporter; DA transporter (	M95167
plasma membrane calcium-transporting ATPase isoform 2	L20977
lecithin-cholesterol acyltransferase (LCAT); phosphatidylch	M12625
thrombin receptor (TR); F2R; PAR1	M62424
NT-3 growth factor receptor precursor (NTRK3); C-trk tyro	U05012
leukocyte tyrosine kinase receptor precursor (LTK)	X60702
NCK melanoma cytoplasmic src homolog (HSNCK)	X17576
MAPKAP kinase (3pK)	U09578
protein kinase C epsilon type (NPKC-epsilon)	X65293
C-jun N-terminal kinase 3 alpha2 (JNK3A2); PRKM10 + M.	U34819 + U07620
serine kinase	U09564
kinase suppressor of ras-1 (KSR1)	U43586
Gem; induced immediate early protein; ras family member	U10550
ras-related protein RAB5A	M28215
sodium- & chloride-dependent GABA transporter 3	S75989
copper-transporting ATPase 2; copper pump 2; Wilson dis	U11700
vesicular acetylcholine transporter (VAChT)	U10554
ephrin type-B receptor 2 precursor; tyrosine-protein kinase	L41939
G protein-coupled receptor kinase GRK5	L15388
G-protein-coupled receptor HM74	D10923
lnk adaptor protein	AF055581
mitogen-activated protein kinase p38 (MAP kinase p38); cy	L35253; L35263
protein kinase C eta type (NPKC-eta); PKC-L	M55284
focal adhesion kinase (FADK); proline-rich tyrosine kinase	L13616
calcium/calmodulin-dependent protein kinase I (CAMKI)	L41816
ephrin A3 precursor (EFNA3); EPH-related receptor tyrosir	U14187
Ral A; GTP-binding protein	X15014
ras-related protein RAB6	M28212
sodium-dependent serotonin transporter; 5HT transporter (	X70697
sodium/potassium-transporting ATPase beta 3 subunit (AT	U51478
thyroxine-binding globulin precursor; T4-binding globulin	M14091
tyrosine kinase receptor HEK; ephrin type-A receptor 3 pre	M83941
transferrin receptor (TFRC); CD71 antigen	X01060
protein-tyrosine phosphatase gamma precursor (R-PTP-ga	L09247
putative src-like adapter protein (SLAP)	U30473
LIM domain kinase 1 (LIMK-1)	D26309
protein kinase C gamma type (PKC-gamma)	Z15114
phosphatidylinositol 3-kinase regulatory alpha subunit (PTI	M61906

phosphorylase B kinase gamma catalytic chain testis isofo	M31606
phosphatidylinositol-4-phosphate 5-kinase II beta; 1-phos|	U85245
transforming protein rhoB; ARHB; ARH6	X06820
neuro epithelioma transforming gene 1 (NEP1; NET1); gua	U02081
sodium-dependent noradrenaline transporter; norepinephri	M65105
synaptic vesicle amine transporter (SVAT); monoamine tra	L09118
transthyretin precursor (TTR); prealbumin; TBPA	K02091
frizzled	L37882
vascular endothelial growth factor receptor 3 precursor (VE	X68203; X69878; U43143
guanine nucleotide release/exchange factor (GNRP); ras-(	L13857
epidermal growth factor receptor kinase substrate EPS8	U12535
MAP kinase-activated protein kinase 2 (MAPKAP kinase 2	U12779
protein kinase C zeta type (NPKC-zeta)	Z15108
p21-activated kinase alpha (PAK-alpha; PAK1)	U24152
casein kinase I gamma 2 (CKI-gamma 2)	U89896
phospholipase C beta 3 (PLC beta 3); 1-phosphatidylinosit	Z16411
ras-related protein RAB3A	M28210
guanine nucleotide regulatory protein tim1	U02082
sodium- & chloride-dependent taurine transporter	Z18956
sodium/potassium-transporting ATPase alpha 1 subunit (N	D00099
alpha-fetoprotein precursor; alpha-fetoglobulin	V01514
ephrin type-B receptor 3 precursor; tyrosine-protein kinase	X75208
ephrin A receptor 4 precursor; tyrosine-protein kinase rece	L36645
c-src kinase (CSK); protein-tyrosine kinase cyl	X59932
T-lymphocyte maturation-associated protein MAL	M15800
dual-specificity mitogen-activated protein kinase kinase 1 (	L05624
protein kinase C theta (PKC-theta)	L07032
serine/threonine-protein kinase PCTAIRE 1 (PCTK1)	X66363
cAMP-dependent protein kinase beta-catalytic subunit (PK	M34181
phosphatidylinositol 3-kinase catalytic subunit alpha isofor	Z29090
ras-related protein RAB-7	X93499
guanine nucleotide-binding protein G-i/G-s/G-t beta subuni	M36429
sodium/glucose cotransporter 2; (Na+/glucose cotransport	M95549
adrenoleukodystrophy protein (ALDP); X-linked ALD	Z21876
serotransferrin precursor; siderophilin; beta-1-metal bindin	M12530
interferon-gamma (IFN-gamma) receptor beta subunit prec	U05875
ephrin type-A receptor 2 precursor; epithelial cell kinase (E	M59371 M36395
tyrosine-protein kinase HCK; P59-HCK & P60-HCK; hemo	M16591
mitogen-activated protein kinase kinase kinase 5 (MAP/EF	D84476
dual specificity mitogen-activated protein kinase kinase 2 (	L11285
calcium/calmodulin-dependent protein kinase type II beta s	U50358
5'-AMP-activated protein kinase catalytic alpha-1 subunit; /	U22456
cAMP-dependent protein kinase gamma-catalytic subunit (	M34182
phosphatidylinositol 4-kinase alpha (PI4-kinase; PTDINS-4	L36151
guanine nucleotide regulatory protein alpha-13 subunit; G1	L22075
transducin beta 5 subunit; GTP-binding protein G(i)/G(s)/G	AF017656
kidney oligopeptide transporter; kidney H+/peptide cotrans	S78203
ATP-binding casette 8 (ABC8); Drosophila white homolog	X91249
lactotransferrin precursor; lactoferrin	X53961
interleukin-6 receptor beta subunit precursor (IL-6R-beta; i	M57230
serine/threonine-protein kinase receptor R4 precursor (SKI	L11695

70-kDa zeta-associated protein (ZAP70)	L05148
myosin light chain kinase (MLCK) smooth muscle & non-m	U48959
dual specificity mitogen-activated protein kinase kinase 3 (	L36719
calcium/calmodulin-dependent protein kinase type IV catal	L24959
tyrosine-protein kinase tec	D29767
cAMP-dependent protein kinase type I beta regulatory sub	M65066
phospholipase C beta 2 (PLC-beta 2; PLCB2); 1-phosphat	M95678
guanine nucleotide-binding protein G(I)/G(S)/G(T) beta sul	M36430
GTP-binding protein ras associated with diabetes (RAD1)	L24564
sodium-dependent proline transporter	S80071
canalicular multispecific organic anion transporter; multidru	U63970
melanotransferrin precursor; melanoma-associated antigei	M12154
stromal cell derived factor 1 receptor (SDF1 receptor); fusi	D10924
anaplastic lymphoma kinase GN (ALK)	U62540
c-fer proto-oncogene	J03358
titin	X69490
c-jun N-terminal kinase kinase 1 (JNKK); JNK activating ki	L36870
casein kinase II alpha subunit (CK II); CSNK2A1	J02853
Bruton's tyrosine kinase (BTK); agammaglobulinaemia tyrc	U10087; X58957
cAMP-dependent protein kinase type II beta regulatory sul	M31158
phospholipase C gamma 1 (PLC-gamma 1; PLCG1); 1-ph	M34667
ras-related C3 botulinum toxin substrate 1; p21-rac1; ras-li	M29870; M31467
RalB GTP-binding protein	M35416
neutral amino acid transporter A (SATT); alanine/serine/cy	L14595
cystic fibrosis transmembrane conductance regulator (CFT	M28668
Insulin receptor-related protein precursor (IR-related recep	J05046
ephrin type-A receptor 1 precursor; tyrosine-protein kinase	M18391
fibroblast growth factor receptor 3 precursor (FGFR3); JTK	M58051 + X58255
growth factor receptor-bound protein 2 (GRB2) isoform; GI	L29511; M96995
cAMP-dependent protein kinase I alpha regulatory subunit	M33336
dual specificity mitogen-activated protein kinase kinase 5 (	U25265
cAMP-dependent protein kinase alpha-catalytic subunit (PI	X07767
Janus kinase 2 (JAK2); receptor-associated tyrosine kinas	AF005216
focal adhesion kinase 2 (FADK2; FAK2); cell adhesion kin	L49207 + U43522 + U33284
phospholipase C-gamma-2 (PLC-gamma-2; PLCG2); 1-ph	X14034
vav oncogene	X16316
dual-specificity protein phosphatase 9; mitogen-activated p	Y08302
protein-tyrosine phosphatase 1E	L34583
PTPCAAX1 nuclear tyrosine phosphatase (PRL-1)	U48296
adenylyl cyclase IX	AF036927
signal transducer and activator of transcription 3 (STAT3);	L29277
14-3-3 protein beta/alpha; protein kinase C inhibitor proteir	X57346
FKBP-rapamycin associated protein (FRAP); rapamycin ta	L34075
retinoic acid receptor beta (RXR-beta; RXRB)	M84820; X63522; S54072
caspase & rip adaptator with death domain (CRADD); rip-a	U84388
calpain p94 large (catalytic) subunit; calcium-activated neu	X85030
granzyme A precursor; cytotoxic T-lymphocyte proteinase	M18737
poly(ADP-ribose) polymerase (PARP; PPOL); ADPRT; NA	M18112; J03473
DNA polymerase beta subunit (DPOB)	D29013
MCM2 DNA replication licensing factor; nuclear protein BM	D21063
DNA excision repair protein ERCC1	M13194

leukocyte common antigen precursor (L-CA); CD45 antige	Y00638
cGMP-inhibited 3',5'-cyclic phosphodiesterase B (CGI-PDE	U38178
retinal guanylyl cyclase 1 precursor (RETGC-1); retinal gua	M92432
signal transducer and activator of transcription 6 (STAT6);	U16031
protein kinase C substrate 80-kDa protein heavy chain (PK	J03075
SH3P17 SH3 domain-containing protein	U61166
WSL protein + TRAMP + Apo-3 + death domain receptor 3	Y09392 + U75380 + U74611 + U83597
CD40 receptor-associated factor 1 (CRAF1)	U21092
calcium-dependent protease small (regulatory) subunit; ca	X04106
CAD; DNA fragmentation factor 40-kDa subunit (DFF40)	AF064019
inducible nitric oxide synthase (INOS); type II NOS; hepatc	L09210
DNA polymerase gamma (POLG); mitochondrial DNA poly	X98093
MCM4 DNA replication licensing factor; CDC21 homolog	X74794
xeroderma pigmentosum group D complementing protein (	X52221
protein-tyrosine phosphatase 1B (PTP-1B)	M31724
cGMP-inhibited 3',5'-cyclic phosphodiesterase A (CGI-PDE	M91667
retinal guanylyl cyclase 2 precursor (RETGC-2); retinal gua	L37378
cAMP-response element binding protein (CREB)	M34356
linker for activation of T-cells (LAT)	AF036906; AF036905
SH3P18 SH3 domain-containing protein	U61167
CD27L antigen receptor precursor; T-cell activation CD27	M63928
FAN protein	X96586
BAD protein; bcl-2 binding component 6 (BBC6); bcl-2L8	U66879
DNA fragmentation factor 45 (DFF45)	U91985
defender against cell death 1 (DAD1)	D15057
DNA polymerase delta catalytic subunit	M80397
MCM5 DNA replication licensing factor; CDC46 homolog	X74795
xeroderma pigmentosum group B complementing protein (	M31899
protein-tyrosine phosphatase 2C (PTP-2C); SH-PTP2	L08807
3'5'-cAMP phosphodiesterase HPDE4A6	U18087
soluble guanylyl cyclase beta 2 subunit; guanylate cyclase	AF038499
RalGDSB; GTP/GDP dissociation stimulator for a ras-relat	U14417
hint protein; protein kinase C inhibitor 1 (PKCI1)	U51004
FRAP-related protein; protein kinase ATR	U49844
lymphocyte activation CD30 antigen; KI-1	M83554
caspase-2 precursor (CASP2); ICH-1L protease + ICH-1S	U13021 + U13022
BCL-2 binding athanogene-1 (BAG-1); glucocorticoid rece	S83171; Z35491
rac-alpha serine/threonine kinase (rac-PK-alpha); protein	M63167
inhibitor of apoptosis protein 3 (API3; IAP3); X-linked inhib	U45880; U32974
DNA topoisomerase I (TOP1)	J03250
MCM6 DNA replication licensing factor ; p105MCM	D84557
excision repair protein ERCC6; Cockayne syndrome protei	L04791
leukocyte antigen-related protein precursor (LAR) ; PTPRF	Y00815 + X69398
adenylate cyclase type I; ATP pyrophosphate-lyase; Ca2+/	L05500
cGMP-dependent 3',5'-cyclic phosphodiesterase (CGS-PD	U67733
oligophrenin 1	AJ001189
macMARCKS; MARCKS-related protein (MRP); MLP	X70326
connector enhancer of KSR-like protein (CNK)	AF100153
fasL receptor; apoptosis-mediating surface antigen fas; AF	M67454
caspase-3 (CASP3); apopain precursor; cysteine protease	U13737
bcl2 homologous antagonist/killer (BAK)	U23765; U16811; X84213

death-associated protein kinase 1 (DAP kinase 1; DAPK1)	X76104
cytoplasmic dynein light chain 1 (HDLC1); protein inihibitor	U32944
DNA topoisomerase II alpha (TOP2A)	J04088
MCM7 DNA replication licensing factor; CDC47 homolog; p	D55716
6-O-methylguanine-DNA methyltransferase (MGMT); meth	M29971
serine/threonine protein phosphatase PP2A-alpha catalytic	X12646
adenylate cyclase type II; ATP pyrophosphate-lyase; aden	X74210
neurogranin (NRGN); RC3	Y09689
ran GTPase activating protein 1 (RANGAP1)	X82260
14-3-3 protein sigma; stratifin; epithelial cell marker protein	AF029082
CD40 ligand (CD40-L); tumor necrosis factor (TNF)-related	L07414
tumor necrosis factor receptor 1 (TNFR1); tumor necrosis f	M33294
caspase-4 precursor (CASP4); ICH-2 protease; TX proteas	U28014 + U28015
apoptosis regulator bax	L22474
interferon-inducible RNA-dependent protein kinase (P68 ki	M35663; U50648
cytochrome P450 reductase	S90469
proliferating cyclic nuclear antigen (PCNA); cyclin	M15796; J04718
photolyase/blue-light receptor homolog	D84657
mutL protein homolog; DNA mismatch repair protein MLH1	U07418
protein phosphatase PP2A 55-kDa regulatory subunit neur	M64930
guanylate cyclase soluble alpha 2 subunit	X63282; Z50053
recoverin; cancer-associated retinopathy protein (CAR pro	S43855
rap1 GTPase activating protein 1 (RAP1GAP)	M64788
GAP-associated protein	U17032
fas antigen ligand (FASL); apoptosis antigen ligand (APTL	D38122; U08137
tumor necrosis factor receptor (TNFR) + tumor necrosis f	M32315 + M55994
caspase-6 precursor (CASP6); cysteine protease MCH2 is	U20536 + U20537
apoptosis regulator bcl-2	M14745
Fas-activated serine/threonine (FAST) kinase	X86779
cytoplasmic antiproteinase 3 (CAP3); protease inhibitor 19	U71364
replication protein A 70-kDa subunit (RPA70; REPA1; RF-A	M63488
nibrin (NBS1)	AF051334
xeroderma pigmentosum group G complementing protein (	L20046; X69978
protein phosphatase PP2A 55-kDa regulatory subunit alph	M64929
guanylate cyclase soluble beta-1 subunit; guanylate cyclas	X66533
S100 calcium-binding protein A7; psoriasin	M86757
rap1 GTPase-GDP dissociation stimulator 1; SMG p21 stim	X63465
tuberin; tuberous sclerosis 2 protein (TSC2)	X75621
tumor necrosis factor precursor (TNF-alpha; TNFA); cache	X01394
protein-tyrosine phosphatase zeta precursor (R-PTP-zeta)	M93426
cysteine protease ICE-LAP3	U39613
apoptosis regulator bclw; KIAA0271; BCL2L2	U59747
apoptotic protease activating factor 1 (APAF1)	AF013263
ionizing radiation resistance-conferring protein + death-ass	U18321 + X83544
replication protein A 14-kDa subunit (RP-A) (RF-A); replica	L07493
DNA-(apurinic or apyrimidinic site) lyase; AP endonuclease	X59764; X66133
xeroderma pigmentosum group C repair complementing pr	D21090
protein phosphatase 2B regulatory subunit; calcineurin B s	M30773
bone marrow stromal antigen 1 (BST-1); ADP-ribosyl cycla	D21878
S100 calcium-binding protein A1; S-100 protein alpha chai	X58079
rho GDP dissociation inihibitor 1 (RHO-GDI 1); RHO-GDI a	X69550

TRRAP protein	AF076974
lymphotoxin-alpha precursor (LT-alpha); tumor necrosis fa	D12614
adenosine A1 receptor (ADORA1)	S56143
caspase-8 precursor (CASP8); ICE-like apoptotic protease	U60520; U58143; X98172; AF00962
apoptosis regulator bcl-x	Z23115; L20121; L20122
IEX-1L anti-death protein; PRG-1; DIF-2	AF039067; AF071596
inhibitor of apoptosis protein1 (HIAP1; API1) + IAP homol	U45878 + U37546
activator 1 140-kDa subunit (A1 140-kDa subunit); replicati	L14922
ataxia telangiectasia (ATM)	U33841
DNA mismatch repair protein PMS1 (PMS1 protein homolo	U13695
serine/threonine protein phosphatase 2B catalytic subunit	L14778
calcium/calmodulin-dependent 3',5'-cyclic nucleotide phos	U40370
interferon regulatory factor 1 (IRF1)	X14454
GTPase-activating protein (GAP); ras p21 protein activator	M23379
leucine-rich repeat protein SHOC-2; ras-binding protein SU	AF068920
lymphotoxin-beta (LT-beta; LTB); tumor necrosis factor C (	L11015
adenosine A2A receptor (ADORA2A)	X68486
caspase-9 precursor (CASP9); ICE-like apoptotic protease	U56390; U60521
induced myeloid leukemia cell differentiation protein MCL-	L08246
SL cytokine precursor; FLT3 ligand (FLT3LG)	U04806; U03858
inhibitor of apoptosis protein 2 (HIAP2; IAP2) + IAP homol	U45879 + U37547
activator 1 40-kDa subunit; replication factor C 40-kDa sub	M87338
Ku 70-kDa subunit; ATP-dependent DNA helicase II 70-kD	M32865; S38729
DNA mismatch repair protein PMS2 (PMS1 protein homolo	U13696
protein phosphatase 2C alpha isoform (PP2C-alpha)	S87759
calcium/calmodulin-dependent 3',5'-cyclic nucleotide phos	U56976
cAMP response element binding protein (CRE-BP1); trans	M31630
58-kDa inhibitor of the RNA-activated protein kinase	U28424
IkappaB kinase complex-associated protein (IKAP)	AF044195
TNF-related apoptosis inducing ligand (TRAIL); APO-2 liga	U57059
adenosine A3 receptor (ADORA3)	X76981
caspase-10 precursor (CASP10); ICE-LIKE apoptotic prote	U60519
BCL-2-related protein A1 (BCL2A1); BFL1 protein; hemop	U29680; Y09397
cellular apoptosis susceptibility protein (CAS); chromosom	U33286
ALG-2 calcium-binding protein	AF035606
DNA polymerase epsilon subunit B; DNA polymerase II su	AF025840
Ku (p70/p80) subunit; ATP-dependent DNA helicase II 86-	M30938
Rad50	U63139
serine/threonine protein phosphatase PP1-alpha 1 catalyti	M63960
ephrin A4 precursor (EFNA4); EPH-related receptor tyrosi	U14188
NF-kappaB transcription factor p65 subunit; RELA; NFKB3	L19067
cortactin; amplaxin; ems-1 oncogene	M98343
zyxin + zyxin-2	X94991; X95735
CD27 ligand (CD27LG); CD70 antigen	L08096; S69339
receptor interacting protein; serine/threonine protein kinas	U25994; U50062
interleukin-1 beta convertase precursor (IL-1BC); IL-1 beta	U13699; M87507; X65019
bcl-2 interacting killer (BIK); NBK apoptotic inducer protein	X89986; U34584
growth arrest & DNA-damage-inducible protein 153 (GADI	S40706; S62138
DNA polymerase epsilon catalytic subunit A; DNA polymer	L09561
replication factor C 36-kDa subunit (RFC36); activator 1 36	L07540
DNA ligase I; polydeoxyribonucleotide synthase (ATP) (DN	M36067

DNA-repair protein complementing XP-A cells; xeroderma	D14533
dual-specificity protein phosphatase 2; PAC-1	L11329
cAMP-dependent 3',5'-cyclic phosphodiesterase 4D (PDE4	U02882
signal transducer and activator of transcription 1 alpha/beta	M97935
muscle/brain cAMP-dependent protein kinase inhibitor (PK	S76965
leukemia inhibitory factor receptor precursor (LIF-R)	X61615
insulin-like growth factor I receptor (IGF1R)	X04434; M24599
DAXX	AF015956
calpain 1 large (catalytic) subunit; mu-type calcium-activate	X04366
NIP1 (NIP1)	U15172
clusterin precursor (CLU); complement-associated protein	M74816
MCM3 DNA replication licensing factor; DNA polymerase a	D38073
replication factor C 38-kDa subunit (RFC38); activator 1 38	L07541
DNA ligase III (LIG3); polydeoxyribonucleotide synthase	X84740
DNA-repair protein complementing XP-C cells; xeroderma	D21089
myotubularin	U46024
adenylate cyclase VII; ATP pyrophosphate-lyase; adenylyl	D25538
signal transducer and activator of transcription 2 (STAT2);	U18671; M97934
14-3-3n protein eta; protein AS1; YWHAH; YWHA1	L20422
junction plakoglobin (JUP); desmoplakin III (DP3)	M23410; Z68228
retinoic acid receptor epsilon (RAR-epsilon); retinoic acid r	X07282; Y00291
tumor necrosis factor receptor 1-associated death domain	L41690
calpain 2 large (catalytic) subunit; M-type calcium-activate	M23254
NIP3 (NIP3)	U15174
early response protein NAK1; TR3 orphan receptor	L13740
DNA polymerase alpha catalytic subunit (POLA)	X06745
activator 1 37-kDa subunit; replication factor C 37-kDa sub	M87339
DNA ligase IV (LIG4); polydeoxyribonucleotide synthase	X83441
uracil-DNA glycosylase precursor (UNG1)	X15653
DNA-repair protein XRCC1	M36089
growth arrest & DNA-damage-inducible protein (GADD45);	M60974
galanin receptor type 1 (GALNR1; GALR1)	L34339
neuronal acetylcholine receptor protein beta-2 subunit prec	X53179
low-affinity nerve growth factor receptor (NGF receptor; NC	M14764
dopamine beta-hydroxylase (DBH); dopamine-beta-monoo	X13255
neuroendocrine protein 7B2 precursor; secretory granule e	Y00757
achaete-scute homolog 1 (ASH1)	L08424
myelin proteolipid protein (PLP); lipophilin	M17085
ataxia-telangiectasia group D-associated protein	L24203
transcription intermediary factor 1 beta (TIF1B); KRAB-ass	U78773
CCAAT-BINDING FACTOR (CBF).	M37197
E2F-3	Y10479
CACCC-box DNA-binding protein	L04282
DNA-dependent protein kinase (DNA-PK) + DNA-PK catal	U35835 + U47077
muscle-specific DNase I-like precursor (DNase1L1; DNL1L	X90392; L40817; U06846
somatostatin receptor type 2 (SS2R); SRIF-1	M81830
5-hydroxytryptamine 3 receptor precursor (5-HT-3); seroto	D49394
aromatic-L-amino-acid decarboxylase; DOPA decarboxyla	M76180
phenylethanolamine N-methyltransferase (PNMTase); nora	J03727
glial growth factor 2 precursor (GGFHPP2); neuregulin; he	L12260; L12261 + U02326 + M94165
brain-specific polypeptide PEP-19; brain-specific antigen P	U52969

peripheral myelin protein 22 (PMP22); CD25 protein; SR13	D11428
cyclic-AMP-dependent transcription factor atr-1; TREB36 p	X55544
transcription intermediary factor 1 (TIF1)	AF009353
hepatic leukemia factor (HLF)	M95585
E2F dimerization partner 1; DRTF1-polypeptide 1 (DP1)	L23959
60S ribosomal protein L6 (RPL6); TAX-responsive enhanc	X69391
DNA damage repair & recombination protein 52 (RAD52)	U12134
melatonin receptor type 1A (MEL-1A-R)	U14108
prostaglandin E2 (PGE) receptor EP4 subtype (PTGER4; I	L25124; D28472
gamma-aminobutyric-acid receptor epsilon subunit precurs	U66661
acetylcholinesterase precursor (ACHE)	M55040
secretogranin II precursor (SGII); chromogranin C	M25756
nociceptin precursor; orphanin FQ; PPNOC	X97370
neuronatin; brain-specific mammalian developmental gene	U25033
myelin-oligodendrocyte glycoprotein precursor (MOG)	U18840
CCAAT transcription binding factor gamma subunit	Z74792
YL-1 protein	D43642
early growth response protein 3 (EGR3); zinc finger protein	S40832; X63741
interferon regulatory factor 2 (IRF2)	X15949
cellular nucleic acid binding protein (CNBP); sterol regulato	M28372
HHR23A; UV excision repair protein protein RAD23A	D21235
5-hydroxytryptamine 1A receptor (5HT1A); serotonin recep	S64045
metabotropic glutamate receptor 5 precursor (GRM5; MGL	D28538
gamma-aminobutyric-acid receptor pi subunit precursor (G	U95367
choline O-acetyltransferase (CHAT); choactase; choline ac	S45018
neurotensin/neuromedin N precursor (NT/NMN)	U91618
leptin precursor; obesity factor; obese protein	U43415
roundabout 2 (ROBO2)	AF040991
myelin basic protein (MBP)	M13577
CCAAT/enhancer binding protein alpha (C/EBP alpha)	U34070
metal-regulatory transcription factor	X78710
human immunodeficiency virus type I enhancer-binding pro	M60119
LYL-1 protein	M22637
basic transcription factor 2 44-kDa subunit (BTF2p44)	Z30094
ubiquitin-conjugating enzyme E2 17-kDa (UBE2A); ubiquiti	M74524
5-hydroxytryptamine 2A receptor (5HT2A); serotonin recep	M86841
orexin receptor 2	AF041245
GABA-B receptor 1A subunit (GABA-BR1A)	Y11044
glutamate decarboxylase 67-kDa isoform; 67-kDa glutamic	M81883
neuromedin B precursor	M21551
neuronal pentraxin II precursor (NP2)	U26662
veli-1	AF087693
neuroglycan C precursor	AF059274
hepatocyte nuclear factor 4 (HNF4); transcription factor 14	X76930
microphthalmia-associated transcription factor (MITF)	Z29678
ets-related gene transforming protein (ERG1)	M21535
nuclear factor NF-kappa-B p100 subunit; nuclear factor NF	X61498
estrogen receptor hSNF2b; global transcription activator S	D26156
translin; recombination hotspot binding protein	X78627
mu-type opioid receptor (MOR-1)	L25119
P2X purinoceptor 1; ATP receptor P2X1	X83688

GABA-B receptor 2 subunit (GABA-BR2)	AF056085
glutamate decarboxylase 65-kDa isoform; 65-kDa glutamic	M81882
preprotachykinin beta (beta-PPT); substance K; neuromed	X54469; M28019
survival of motor neuron (hSMN)	U18423
43-kDa postsynaptic protein; acetylcholine receptor-associ	Z33905
parkin	AB009973
TIS11B protein; EGF response factor 1 (ERF1)	X79067
transcription repressor protein PRDI-BF1; beta-interferon g	AF084199
transcription factor GATA-4; GATA binding factor-4	L34357
octamer-binding transcription factor 1 (oct-1; OTF1); octan	X13403
transcriptional repressor NF-X1	U15306
recA-like protein HsRad51; DNA repair protein RAD51 hon	D13804
nociceptin receptor; orphanin FQ receptor; kappa-type 3 o	X77130
P2X purinoceptor 3; ATP receptor P2X3	Y07683
glutamate receptor 5 precursor (GLUR5); ionotropic glutan	L19058
neuroendocrine convertase 1 precursor (NEC 1); prohorm	X64810
proenkephalin A precursor	J00123
lissencephalin X; doublecortin (DCX)	AF040255
synaptosomal-associated protein 25 (SNAP-25); super pro	L19761
huntingtin; Huntington's disease protein (HD protein)	L12392
HIV-1 TATA element modulatory factor	L01042
PCAF-associated factor 65 beta	AF069736
glucocorticoid receptor repression factor 1	M73077
pre-B-cell leukemia transcription factor-1; homeobox prote	M86546
cAMP-responsive element-binding protein (CREB1)	L05515
V(D)J recombination activating protein 1 (RAG1)	M29474
prostaglandin E2 receptor EP3 subtype (PGE receptor EP	S69200
gamma-aminobutyric-acid receptor beta-1 subunit precurs	X14767
neuronal acetylcholine receptor protein alpha 6 subunit pre	U62435
neuroendocrine convertase 2 precursor (NEC 2); prohorm	J05252
beta-neoendorphin-dynorphin precursor; proenkephalin B	K02268
roundabout 1 (ROBO1)	AF040990
synaptophysin (SYP); major synaptic vesicle protein p38	X06389
major prion protein precursor (PRP); PRP27-30; PRP33-3	M13667
hypoxia-inducible factor 1 alpha (HIF1 alpha); ARNT-intera	U22431
PCAF-associated factor 65 alpha	AF069735
homeobox protein HOX-A5; HOX-1C	M26679
endothelial transcription factor GATA2	M68891
GA-binding protein alpha subunit (GABP-alpha); transcript	D13316
V(D)J recombination activating protein 2 (RAG2)	M94633
substance-P receptor (SPR); NK-1 receptor (NK-1R)	S62045
gamma-aminobutyric-acid receptor gamma-2 subunit prec	X15376
neuronal acetylcholine receptor protein beta 4 subunit pre	U62439
membrane-bound & soluble catechol-O-methyltransferase	M65212
acyl-CoA-binding protein (ACBP); diazepam binding inhibi	M14200
CASK	AF032119
presynaptic density protein 95 (PSD95); DLG4	U83192
Alzheimer's disease amyloid A4 protein precursor; proteas	Y00264
jun activation domain binding protein	U65928
SPT3-like protein	AF069734
interferon regulatory factor 7 (IRF-7)	U73036

basic transcription element-binding protein 2 (BTEB2); GC	D14520
GA-binding protein beta-2 subunit (GABP-beta2); transcrip	D13318
telomerase reverse transcriptase (hTRT)	AF015950
substance-K receptor (SKR); neurokinin A receptor; NK-2 r	M57414
glutamate receptor 1 precursor (GLUR-1); GLUR-A; GluH1	M64752
glutamate (NMDA) receptor subunit epsilon 2 precursor; N	U90278
tryptophan 5-hydroxylase (TRPH); tryptophan 5-monooxyg	X52836
neurotrophin-3 precursor (NT-3); neurotrophic factor (HDN	X53655; M37763
neuromodulin; axonal membrane protein GAP-43; PP46; p	M25667
synapsin IIIA	AF046873
atrophin-1; dentatorubral-pallidoluysian atrophy protein (DI	D31840
ets domain protein elk-3; NET; SRF accessory protein 2 (S	Z36715
ADA3-like protein	AF069733
interleukin enhancer-binding factor (ILF) ILF + interleukin e	U58198 + U58197 + U58196
basic transcription factor 62-kDa subunit (BTF2)	M95809
transcription factor AREB6	D15050
TRF1-interacting ankyrin-related ADP-ribose polymerase t	AF082556
neuromedin K receptor (NKR); neurokinin B receptor; NK-3	M89473
glutamate receptor 2 precursor (GLUR2); GLUR-B; GLUR-	L20814
glutamate receptor subunit epsilon 3 precursor (GRIN2C);	L76224
flavin-containing amine oxidase A; monoamine oxidase (M	M68840
neurotrophin-4 (NT-4)	M86528
axonin-1 precursor; transient axonal glycoprotein 1 (TAG-1	X68274
channel associated protein of synapse 110 (CHAPSYN 11	U32376
paraneoplastic encephalomyelitis antigen HUD; HU-antige	M62843
histone acetyltransferase B subunit 2; retinoblastoma-bind	U35143
ADA2-like protein	AF069732
RBP2 retinoblastoma binding protein	S66431
helix-loop-helix protein; DNA-binding protein inhibitor Id-2	M97796
transcription factor ZFM1	D26120
delta lactoferrin	U84119
neuropeptide Y receptor type 1 (NPY1R)	M88461; M84755
glycine receptor alpha-1 subunit precursor (GLRA1); strych	X52009
P2X purinoceptor 5 (P2X5)	AF016709
histidine decarboxylase (HDC)	X54297
neuropeptide Y precursor (NPY)	K01911
glia maturation factor beta (GMF-beta)	M86492
amphiphysin (AMPH)	U07616
Machado-Joseph disease protein 1 (MJD1)	S75313
BRCA1-associated ring domain protein	X82200
B-cell lymphoma 3-encoded protein (bcl-3)	M31732
BRCA1-associated ring domain protein (BARD1)	U76638
DNA-binding protein SMBP-2; glial factor-1 (GF-1)	L14754
ZFM1 protein alternatively spliced product	D26121
deoxyribonuclease I (DNase I)	M55983
metabotropic glutamate receptor 1 precursor (GRM1; MGL	U31215
glycine receptor beta subunit precursor (GLRB)	U33267
P2X purinoceptor 6 (P2X6); P2XM	AB002058
phenylalanine-4-hydroxylase (PAH); phe-4-monooxygenas	K03020
5-hydroxytryptamine 1D receptor (5-HT-1D; HTR1D); sero	M89955
C-jun N-terminal kinase kinase 2 (JNKK2); mitogen-activat	AF022805

neurexin III alpha	AF099810
Kallmann syndrome protein precursor (KAL); adhesion mo	M97252
serum response factor (SRF)	J03161
B-cell lymphoma 6 protein (bcl-6); zinc finger protein 51 (Z	U00115
transcriptional repressor CTCF	U25435
global transcription activator SNF2L1	M88163
transcription factor RZR-alpha (RZRA); RAR-related orpha	U04897 + L14611
deoxyribonuclease II (DNase II); acid DNase; lysosomal D	AF060222
D2 dopamine receptor (DRD2)	M29066
neuronal acetylcholine receptor protein alpha-3 subunit pre	M37981
leptin receptor precursor; obese receptor (OB receptor; OE	U43168
tyrosine 3-hydroxylase (TYH); tyrosine 3-monooxygenase	Y00414
glial cell line-derived neurotropic factor precursor (GDNF)	L19063
myelin-associated glycoprotein precursor (MAG)	M29273
synapse-associated protein 97 (SAP97); homolog of Drosc	U13897
FCMD; fukutin	AB008226
activated RNA polymerase II transcriptional coactivator p1	U12979
CYCLIC-AMP-DEPENDENT TRANSCRIPTION FACTOR	L19871
PRB-binding protein E2F1; retinoblastoma-binding protein	M96577
interferon consensus sequence-binding protein (ICSBP)	M91196
paired box homeotic protein (PAX8) isoforms 8A/8B + isofo	L19606
brain-specific homeobox/POU domain protein 3A (brn-3A);	L20433
transcription factor TFIIB; GTF2B	M76766
transcription factor NF-ATc	U08015
ets transcription factor; NERF2	U43188
CCAAT displacement protein; CUTL1; CASP	L12579
Ini1	U04847
osteoblast specific factor 2 (OSF2); OSF-2P1	D13666
leukosialin precursor; leukocyte sialoglycoprotein; sialopho	J04536
integrin beta 5 subunit precursor (ITGB5)	J05633
beta catenin (CTNNB)	X87838; Z19054
granulocyte colony stimulating factor receptor precursor (G	M59818
interleukin-2 receptor alpha subunit precursor (IL-2 recepto	X01057; X01058; X01402
interleukin-12 receptor precursor (IL-12R)	U03187
growth arrest & DNA-damage-inducible protein 45 gamma	AF078078
nuclear factor I (NFI); NFI-X	L31881
transcription factor HTF4; transcription factor 12 (TCF12);	M80627
R kappa B DNA-binding protein	U08191
TRAF-interacting protein (I-TRAF) + TRAF family member-	U59863 + U63830
cAMP-dependent transcription factor ATF-4; DNA-binding	D90209
DNA cytosine-5-methyltransferase (DNA metase; MCMT)	X63692
cadherin 3 (CDH3); placental cadherin precursor (P-cadhe	X63629
corneodesmosin precursor (CDSN); S protein	L20815
integrin alpha 4 precursor (ITGA4); VLA4; CD49D antigen	L12002; X16983; X15356
semaphorin; CD100	U60800
C5a anaphylatoxin receptor (C5AR); CD88 antigen	M62505
interleukin-6 receptor alpha subunit precursor (IL-6R-alpha	M20566; X12830
interleukin-1 receptor type II precursor (IL-1R2); IL-1R-beta	X59770
growth arrest & DNA-damage-inducible protein 45 beta (G.	AF078077
RNA polymerase II elongation factor SIII p15 subunit	L34587
transcription elongation factor SII	M81601

transcription factor 11 (TCF11); HBZ17; locus control regic	U08853
transcription initiation factor TFIID 31-kDa subunit; TAFII31	U30504
heat shock factor protein 1 (HSF1); heat shock transcriptio	M64673
DNA-binding protein A	M24069
cadherin 5 (CDH5); vascular endothelial cadherin precursc	X79981; X59796
vascular cell adhesion protein 1 precursor (V-CAM 1); CD1	M30257
cell surface adhesion glycoproteins LFA-1/CR3/p150,95 be	M15395
T-cell surface glycoprotein T4/leu-3; CD4 antigen	M12807
neuromedin B receptor (NMBR); neuromedin-B-preferring	M73482
interferon-alpha/beta receptor alpha subunit precursor (IFN	J03171
high-affinity interleukin-8 receptor A (IL-8R A); IL-8 recepto	M68932
14.5-kDa translational inhibitor protein (p14.5); UK114 anti	X95384
homeobox protein HOXB7; HOX2C; HHO.c1	M16937
nuclease-sensitive element DNA-binding protein (NSEP)	M83234
nuclear factor NF45	U10323
AP4 basic helix-loop-helix DNA-binding protein	S73885
transcriptional activator hSNF2-alpha	D26155
telomeric repeat binding factor 1 (TRF1)	U40705
cadherin 11 precursor (CDH11); osteoblast-cadherin (OB-c	L34056
E-selectin precursor; endothelial leukocyte adhesion molec	M30640
platelet membrane glycoprotein IIB precursor (GP2B); inte	M34480; J02764
B-lymphocyte CD19 antigen precursor; B-lymphocyte surfa	M21097
Duffy blood group antigen; FY glycoprotein (GPFY); glycop	U01839
interleukin-2 receptor beta subunit precursor (IL-2 receptor	M26062
androgen receptor (AR)	M20132; J03180
beta-defensin 2 precursor (hBD2); skin-antimicrobial peptic	Z71389
transcription factor E2-alpha (E2A); immunoglobulin enhar	M31523
transcriptional regulator interferon-stimulated gene factor 3	M87503
nuclear factor NF90	U10324
C-ets-2	J04102
putative transcription activator DB1	D28118
TTAGGG repeat binding factor 2 (hTRF2); telomeric DNA-	AF002999
muscle cadherin precursor (M-cadherin); cadherin 14 or15	D83542
NADH-ubiquinone oxidoreductase B18 subunit; complex I-	M33374
integrin beta 6 precursor (ITGB6)	M35198; J05522
myeloid cell surface CD33 antigen precursor	M23197
monocyte chemoattractant protein 1 receptor (MCP-1RA);	U03882
interleukin-3 receptor alpha subunit precursor (IL-3R-alpha	M74782
angiotensin II type 1A receptor (AT1AR)	M87290
defensin 6 precursor	M98331
transcription initiation factor IID; TATA-box factor; TATA se	M34960
homeobox 2.1 protein (HOX2A); HOXB5; HU1; HHO.C10	M92299
homeobox A1 protein (HOXA1); HOX1F	U10421
raf-responsive zinc finger protein	AF072825
DNA-binding protein TAXREB302; albumin D box-binding	D28468
polyadenylate binding protein-interacting protein 1 (PAIP1)	AF013758
cadherin 8 (CDH8)	L34060
neural-cadherin precursor (N-cadherin; NCAD); cadherin 2	M34064; X57548; X54315; S42303
integrin alpha 3 (ITGA3); galactoprotein B3 (GAPB3); VLA	M59911
polycystin precursor; autosomal dominant polycystic kidne	U24497
keratinocyte growth factor receptor (KGFR); fibroblast grov	M80634 + U11814 + X52832; M35718 + M87

interleukin-4 receptor alpha subunit precursor (IL-4R-alpha	X52425
follicle stimulating hormone receptor (FSHR); follitropin rec	M95489
cytochrome P450 IA2 (P450-P3) (P450-4)	Z00036
octamer-binding transcription factor 2 (oct-2; OTF2); lymph	M36542
fli-1 oncogene; ergB transcription factor	M93255
homeobox protein hLim1; LHX1	U14755
orphan hormone nuclear receptor	Z30425
zinc finger protein 91 (ZNF92); HPF7; HTF10	L11672
RPD3 protein; histone deacetylase 1 (HD1)	D50405
intercellular adhesion molecule 2 precursor (ICAM2); CD1(	X15606
B-cell differentiation CD72 antigen; Lyb-2	M54992
leukocyte adhesion glycoprotein p150, 95 alpha subunit pr	M81695; Y00093
ciliary neurotropic factor receptor (CNTFR)	M73238
activin type I receptor; serine/threonine-protein kinase rece	U14722
interleukin-5 receptor alpha subunit precursor (IL-5R-alpha	M75914
calcitonin receptor (CTR; CALCR)	L00587
cytochrome P450 IVB1 (EC 1.14.14.1) (P450-HP)	J02871
transcription factor AP-2 (TFAP2; AP2TF)	M36711
paired box protein PAX-5; B-cell specific transcription facto	M96944
trans-acting T-cell specific transcription factor GATA3	X55122; X58072
nuclear factor kappa-B DNA binding subunit (NF-kappaB; I	M58603
guanine nucleotide-binding protein G-s alpha subunit (GNA	M14631
high mobility group protein (HMG-I)	M23619
integrin alpha E precursor (ITGAE); mucosal lymphocyte-1	L25851
CD44 antigen hematopoietic form precursor (CD44H); pha	M59040
fibronectin receptor alpha subunit (FNRA); integrin alpha 5	X06256
erythropoietin receptor (EPOR)	M60459
granulocyte-macrophage colony-stimulating factor recepto	X17648
interferon-alpha/beta receptor beta subunit precursor (IFN-	X77722
beta-2 adrenergic receptor (ADRB2; ADRB2R; B2AR)	M15169
soluble epoxide hydrolase (SEH); epoxide hydratase; cyto:	L05779
mitochondrial transcription factor 1 (MTTF1); TCF6L1	M62810
special AT-rich sequence binding protein 1 (SATB1); MAR	M97287
transcription factor Sp1 (TSFP1)	J03133
zinc-finger DNA-binding protein	D45132
stem cell protein (SCL); T-cell leukemia/lymphoma-5 prote	M29038
procollagen alpha 2(IV) subunit precursor	X05562
integrin beta 8 precursor (ITGB8)	M73780
neural cell adhesion molecule L1 precursor (N-CAM L1); M	M74387
fibronectin receptor beta subunit (FNRB); integrin beta 1 (I	X07979
platelet-activating factor receptor (PAFR)	D10202
CDW40 antigen; CD40L receptor precursor; nerve growth	X60592
interleukin-2 receptor gamma subunit (IL-2R gamma; IL2R	D11086
alpha 1A adrenergic receptor (ADRA1A); alpha 1D adrene	M76446
dimethylaniline monooxygenase (N-oxide forming) 1 (EC 1	M64082
early growth response protein 1 (hEGR1); transcription fac	X52541; M62829
MSX-1 homeobox protein; HOX7	M97676
I-rel (RELB)	M83221
26S protease regulatory subunit 6A; TAT-binding protein 1	M34079
neural retina-specific leucine zipper protein (NRL)	M81840
bystin	L36720

thrombospondin 2 precursor (THBS2; TSP2)	L12350
contactin precursor (CNTN1); glycoprotein gp135	U07819
integrin alpha 6 precursor (ITGA6); VLA6; CD49F antigen	X53586; X59512
endothelin receptor type A (EDNRA; ETA)	L06622
corticotropin releasing factor receptor 1 precursor (CRF-R;	X72304
interferon-gamma receptor (IFNR-gamma; IFNGR1); CDW	J03143
ferrochelatase precursor; protoheme ferro-lyase; heme syr	D00726
glutathione reductase (GRase; GSR; GR)	X15722
transcription factor ETR101	M62831
PAX3/forkhead transcription factor fusion	U02368
Sp2 protein	M97190
purine-rich single-stranded DNA-binding protein alpha (PU	M96684
tristetraproline (TTP); TIS11; ZFP36; growth factor-inducib	M92843
autosomal dominant polycystic kidney disease II (PKD2)	U50928
bone proteoglycan II precursor (PGS2); decorin (DCN)	M14219
neural cell adhesion molecule phosphatidylinositol-linked is	X16841; S71824
integrin beta 4 (ITGB4); CD104 antigen	X53587; X52186; X51841
endothelin receptor type B (EDNRB; ETB); endothelin rece	L06623
cytokine receptor EBI3	L08187
interleukin-9 receptor precursor (IL-9R)	M84747
microsomal glutathione S-transferase II (microsomal GSTI	U77604
microsomal glutathione S-transferase 12 (GST12; MGST1	J03746; B28083
transcriptional enhancer factor (TEF1); protein GT-IIC; trar	M63896
transcription factor IIIC box B-binding subunit	U02619
Sp3 protein	M97191
transcription initiation factor 250-kDa subunit (TAFII250); T	X07024
nucleobindin precursor (NUC)	M96824
tastin	U04810
vitronectin receptor alpha subunit (VNRA); integrin alpha 5	M14648; J02826; M18365
desmoglein 2 precursor (DSG2); HDGC	Z26317; S64273
integrin alpha 1 (ITGA1); laminin & collagen receptor; VLA	X68742
insulin receptor precursor (INSR)	M10051; X02160
CC chemokine receptor type 2 (CC CKR2; CCR2); monoc	U03905
interleukin 10 receptor (IL-10R)	U00672
selenium-binding protein	U29091
glutathione S-transferase pi (GSTP1; GST3)	X08058; M24485
homeobox protein HOX-11; tcl-3 proto-oncogene	M75952
nuclear respiratory factor 1 (NRF1); alpha palindromic binc	U02683
homeobox protein HOX-D3; HOX-4A	D11117
CCAAT-binding transcription factor subunit B (CBF-B); NF-	M59079
neu differentiation factor	U02326
trophinin	U04811
alpha1 catenin (CTNNA1); cadherin-associated protein; al	D13866; D14705; L23805; L22080
platelet membrane glycoprotein IIIA precursor (GP3A); inte	J02703; M25108
integrin alpha 7B precursor (IGA7B)	X74295
platelet-derived growth factor receptor beta subunit (PDGF	M21616
N-sam; fibroblast growth factor receptor1 precursor (FGFR	X66945; M34641; M34186; M37722 + M6388
interferon gamma receptor (IFNGR)	A09781
microsomal stress 70 protein ATPase core precursor	U04735
glutathione S-transferase theta 1 (GSTT1)	X79389
transcriptional repressor protein yin & yang 1 (YY1); delta	M76541

FUSE binding protein	U05040
transcription factor TFIIIB 90 kDa subunit (HTFIIIB90)	U28838
DNA-binding protein HIP116; ATPase; SNF2/SWI2-relatec	L34673
transcription factor LSF	U03494
Herpes virus entry protein C (HVEC)	AF060231
intercellular adhesion molecule-1 precursor (ICAM1); majo	J03132
cell surface glycoprotein mac-1 alpha subunit precursor; C	J04145
leukocyte adhesion glycoprotein LFA-1 alpha subunit prec	Y00796
tumor necrosis factor-inducible protein TSG-6; hyaluronate	M31165
interleukin-7 receptor alpha subunit precursor (IL-7R-alpha	M29696
interleukin-1 receptor type I precursor (IL-1R1); IL-1R-alph	M27492
thiosulfate sulfurtransferase; rhodanese	D87292
heme oxygenase 1 (HO1); HSOXYGR	X06985
heme oxygenase 2 (HO2)	D21243; S34389
quinone oxidoreductase; NADPH:quinone reductase; zeta-	L13278; S58039
MPV17 protein	X76538
bone morphogenetic protein 4 (BMP4) + bone morphogen	D30751 + M22490
thrombomodulin precursor (THBD; THRM); fetomodulin; C	M16552
insulin-like growth factor binding protein 1 (IGFBP1); place	M31145
teratocarcinoma-derived growth factor 1 (TDGF1); epiderm	M96955 + M96956
insulin-like growth factor IA precursor (IGF1A); IGFBP1; sc	M27544 + M37484
glycoprotein hormone alpha subunit precursor	V00518
interferon-beta (IFN-beta; IFNB); fibroblast interferon	M28622
follistatin-related protein precursor	U06863
proteasome component C2; macropain subunit C2; protea	D00759
25-kDa trypsin inhibitor	D45027
plasminogen precursor (PLG)	X05199
heat shock cognate 71-kDa protein	Y00371
heat-shock protein 40 (HSP40)	D49547
alpha-1-acid glycoprotein 1 precursor (AGP1); orosomucoi	X02544
bone morphogenetic protein 5 precursor (BMP5)	M60314
FMLP-related receptor I (FMLPRII); RMLP-related recepto	M76673
vascular endothelial growth factor precursor (VEGF); vasc	M32977; M27281
endothelial-monocyte activating polypeptide II (EMAP II)	U10117
delta-like protein precursor (DLK)	U15979; Z12172
alpha calcitonin precursor	J00109 X00356
interferon-alpha2 precursor (IFN-alpha; IFNA); leukocyte ir	J00209; J00207
complement component 5 (C5)	M65134
proteasome component C3; macropain subunit C3; multica	D00760
matrix metalloproteinase 1 (MMP1); interstitial collagenase	X05231
protein C inhibitor (PROCI; PCI); plasma serine protease ir	M68516; J02639
heat shock 70-kDa protein 6 (heat shock 70-kDa protein B	X51757; M11236
mitochondrial matrix protein P1 precursor; p60 lymphocyte	M34664
alpha-1-antichymotrypsin precursor (ACT)	K01500
bone morphogenetic protein 6 precursor (BMP6)	M60315
macrophage-specific colony-stimulating factor (CSF-1; MC	M37435
pleiotrophin precursor (PTN) + osteoblast specific factor 1	X52946 + D90226 + M57399
vascular endothelial growth factor C precursor (VEGF-C); v	U43142
macrophage inflammatory protein 1 beta precursor (MIP1-	J04130
parathyroid hormone-related protein precursor (PTH-RP)	M17183
interleukin-10 precursor (IL-10); cytokine synthesis inhibito	M57627

puromycin-sensitive aminopeptidase (PSA)	Y07701
proteasome component C5; macropain subunit C5; protea	D00761
matrix metalloproteinase 2 (MMP2); 72-kDa gelatinase A;	J03210; J05471
endothelial plasminogen activator inhibitor-1 precursor (PA	X04429; M14083
heat shock-related 70-kDa protein 2	L26336
heat shock 90-kDa protein A (HSP90A; HSPCA); HSP86	X07270
B94 protein	M92357
bone morphogenetic protein 8 (BMP8; osteogenic protein :	M97016
hepatocyte growth factor activator (HGF activator)	D14012
T-cell-secreted protein I-309 precursor; small inducible cyt	M57502
interferon gamma-induced protein precursor (gamma-IP10	X02530
ribonuclease/angiogenin inhibitor (RAI); placental ribonucle	M36717
interleukin-16 (IL-16); lymphocyte chemoattractant factor (I	M90391
interleukin-13 precursor (IL-13); NC30	L06801
heregulin-beta3; glial growth factor; neuregulin; GGFHPP1	L12261
proteasome component C8; macropain subunit C8; multica	D00762
matrix metalloproteinase 3 (MMP3); stromelysin 1 precurs	X05232
placental plasminogen activator inhibitor 2 (PAI-2; PLANH:	M18082; J02685
glutathione peroxidase (GSHPX1; GPX1)	Y00483; M21304
27-kDa heat-shock protein (HSP27); stress-responsive pro	X54079
C-reactive protein precursor	X56692
bone-derived growth factor 1 (BPGF1)	L42379
hepatoma-derived growth factor (HDGF)	D16431
stem cell factor precursor (SCF); mast cell growth factor (M	M59964
migration inhibitory factor-related protein 14 (MRP14); calg	X06233
erythroid differentiation protein (EDF); inhibin beta A subu	J03634
interleukin-18 precursor (IL-18); interferon-gamma-inducin	D49950
interleukin-14 precursor (IL-14); high molecular weight B-c	L15344
alpha-1-antitrypsin precursor; alpha-1 protease inhibitor; a	X02920
proteasome component C9; macropain subunit C9; multica	D00763
matrix metalloproteinase 7 (MMP7); matrilysin	X07819
metalloproteinase inhibitor 1 precursor (TIMP1); erythroid p	X03124
glutathione peroxidase-gastrointestinal (GSHPX-GI); gluta	X53463
70-kDa heat shock protein 1 (HSP70.1; HSPA1)	M11717
eosinophil granule major basic protein precursor (MBP); pr	Y00809
insulin-like growth factor II (IGF2); somatomedin A	M29645
endothelin 3 (EDN3; ET3)	J05081
heparin-binding EGF-like growth factor (HBEGF); diphtheri	M60278
migration inhibitory factor-related protein 8 (MRP8); calgra	X06234
angiotensin-converting enzyme (ACE)	A00914
interferon gamma precursor (IFN-gamma; IFNG); immune	X01992; M29383
interleukin-11 (IL-11); adipogenesis inhibitory factor (AGIF	M57765
carboxypeptidase H precursor (CPH); carboxypeptidase E	X51405
acrosin precursor	Y00970
matrix metalloproteinase 8 (MMP8); neutrophil collagenase	J05556
tissue inhibitor of metalloproteinases 2 (TIMP2); metallopro	J05593
natural killer cell enhancing factor (NKEFB) + thiol-specific	L19185 + Z22548; X82321
cytosolic superoxide dismutase 1 (SOD1)	K00065; X02317
monocyte chemotactic protein 4 precursor (MCP4); monoc	U46767
platelet-derived growth factor B subunit precursor (PDGFB	X02811; X02744; M12783; M16288
neuroleukin (NLK); glucose-6-phosphate isomerase (GPI);	K03515

hepatocyte growth factor (HGF); scatter factor (SF); hepat	M60718
platelet-derived growth factor A subunit precursor (PDGFA	X06374
prorelaxin H2 precursor (RLN2)	A06925
interleukin-7 (IL-7)	J04156
interleukin-12 beta subunit precursor (IL-12B); cytotoxic ly	M65290
dipeptidyl-peptidase I precursor (DPP-I); cathepsin C; cath	X87212
acrosin-trypsin inhibtor II precursor; HUSI II	M91438
matrix metalloproteinase 9 (MMP9); gelatinase B; 92-kDa	J05070; D10051
tissue inhibtor of mettaloproteinase 4 (TIMP4)	U76456
thioredoxin peroxidase 2 (TDPX2); thioredoxin-dependent	X67951
glutaredoxin	X76648
pancreatitis-associated protein 1 precursor	D13510
granulocyte-macrophage colony stimulating factor (GM-CS	M11220
thrombopoietin precursor (THPO); megakaryocyte colony	L36052; L36051; U11025
keratinocyte growth factor (KGF); fibroblast growth factor 7	M60828
leukemia inhibitory factor precursor (LIF); differentiation-sti	X13967; M63420
renin-binding protein (RENBP; RNBP)	D10232
interleukin-2 precursor (IL-2); T-cell growth factor (TCGF)	A14844
interleukin-12 alpha subunit precursor (IL-12A); cytotoxic ly	M65291
cathepsin H precursor	X07549
leukocyte elastase inhibitor (LEI); monocyte/neutrophil ela	M93056
matrix metalloproteinase 12 (MMP12); metalloelastase	L23808
matrix metalloproteinase 17 (MMP17); membrane-type ma	X89576
cytochrome P450 IIF1 (CYP2F1)	J02906
thioredoxin reductase	X91247
osteoclast stimulating factor	U63717
transforming growth factor-alpha (TGF-alpha; TGFA); EGF	K03222
uromodulin; Tamm-Horsfall urinary glycoprotein (THP)	M17778
brain-derived neurotrophic factor (BDNF)	M61176
acidic fibroblast growth factor (AFGF) + heparin-binding gr	X65778 + X51943 + M13361
glucagon precursor (GCG)	J04040
interleukin-1 alpha precursor (IL-1 alpha; IL1A); hematopoi	X02851
interleukin-15 (IL-15)	U14407
cystatin-related epididymal spermatogenic protein	AF059244
inter-alpha-trypsin inhibitor heavy chain H2 precursor (ITI	X07173
matrix metalloproteinase 14 precursor (MMP14); membrar	D26512; X83535
tripeptidyl-peptidase I precursor; tripeptidyl aminopeptidas	AF017456
dioxin-inducible cytochrome P450 1B1 (CYP1B1)	U03688
NAD(P)H dehydrogenase; quinone reductase; DT-diaphor	J03934
CXC chemokine precursor	AJ002211
transforming growth factor-beta (TGF-beta; TGFB)	X02812; J05114
T-cell-specific rantes protein precursor; sis delta; small ind	M21121
embryonic growth/differentiation factor 1 (GDF1) + UOG-1	M62302
macrophage inflammatory protein 2 alpha (MIP2-alpha); g	X53799
inhibin alpha subunit precursor (INHA)	M13981
interleukin-1 beta precursor (IL-1 ; IL1B); catabolin	K02770
interleukin-9 precursor (IL-9); T-cell growth factor p40	X17543; M30134
major epididymis-specific protein E4 precursor (hE4); epid	X63187
inter-alpha-trypsin inhibitor heavy chain H3 precursor (ITI	X67055
matrix metalloproteinase 15 (MMP15); membrane-type ma	Z48482
dipeptidyl peptidase IV (DPP IV; DPP4); T-cell activation	M74777

S-mephenytoin 4 hydroxylase; cytochrome P450 IIC9 (CYI	M21940 + M15331; M21939 + M61858 + M61
25-hydroxy vitamin D3 1-alpha hydroxylase mitochondrial	AF020192
bone morphogenetic protein 3B precursor (BMP3B); growt	D49493
granulocyte colony-stimulating factor precursor (G-CSF); p	X03438
macrophage inflammatory protein 1 alpha precursor (MIP1	M23452
endothelin 2 (ET2)	M65199
placenta growth factors 1 + 2 (PLGF1 + PLGF2)	X54936
estrogen sulfotransferase (STE; EST1)	U08098
interleukin-3 precursor (IL-3); multipotential colony-stimula	M14743; M17115
interleukin-17 precursor (IL-17); cytotoxic T-lymphocyte-as	U32659
insulin-degrading enzyme; insulysin; insulinase; insulin pro	M21188
inter-alpha-trypsin inhibitor heavy chain H4 precursor (ITI h	D38595
matrix metalloproteinase 16 precursor (MMP16); membran	D50477
myeloblastin precursor (MBN); leukocyte proteinase 3 (PR	M29142
mitochondrial cytochrome P450 XIA1 precursor; P450(SC(	M14565
glutathione synthetase (GSH synthetase; GSH-S); glutathi	U34683
bone morphogenetic protein 1 (BMP1) + procollagen C-pro	M22488 + U50330
transforming growth factor beta2 precursor (TGF-beta2; T(	M19154; M22045; M22046; Y00083
monocyte chemotactic protein 1 precursor (MCP1); monoc	M24545
hepatocyte growth factor-like protein; macrophage-stimula	M74178
granulocyte chemotactic protein 2 (GCP 2); neutrophil-acti	X78686
insulin-like growth factor-binding protein 3 precursor (IGF-b	M31159; M35878
interleukin-4 precursor (IL-4); B-cell stimulatory factor 1 (B	M13982
parathymosin	M24398
methionine aminopeptidase 2 (METAP2); peptidase M2; in	U29607
neuroserpin precursor; protease inhibitor 12	Z81326
matrix metalloproteinase 13 (MMP13); collagenase 3 prec	X75308
cathepsin L precursor; major excreted protein (MEP)	X12451
polymorphic arylamine N-acetyltransferase (PNAT) + mon	X14672; X17059
glutathione S-transferase mu1 (GSTM1; GST1); HB subun	X68676; S01719
bone morphogenetic protein 2A (BMP2A)	M22489
kidney epidermal growth factor (EGF); urogastrone	X04571
oncostatin M (OSM)	M27288
thymosin beta-10 (TMSB10; THYB10); PTMB10	M92381
OX40 ligand (OX40L); GP34; tax-transcriptionally activate	X79929
cellular retinoic acid-binding protein II (CRABP2)	M68867
interleukin-6 precursor (IL-6); B-cell stimulatory factor 2 (B	X04602; M14584
thymosin beta 4; FX	M17733
proteasome activator HPA28 subunit beta	D45248
cytoplasmic antiproteinase 2 (CAP2); protease inhibitor 8	L40377
cathepsin D precursor (CTSD)	M11233
activator of RNA decay (ARD-1)	U14575
serum paraoxonase/arylesterase 1 (PON1); serum aryldial	M63012
glutathione S-transferase A1 (GTH1; GSTA1); HA subunit	M25627
bone morphogenetic protein 3 (BMP3); osteogenin	M22491
cytokine humig; interferon-gamma-induced monokine (MIG	X72755
amphiregulin (AR); colorectum cell-derived growth factor (	M30704
connective tissue growth factor precursor (CTGF)	M92934
interleukin-8 precursor (IL-8); monocyte-derived neutrophil	Y00787
corticotropin-releasing factor-binding protein	X58022
interleukin-5 precursor (IL-5); T-cell replacing factor (TRF);	X04688; J03478

Wnt-13	Z71621
proteasome inhibitor HPI31 subunit	D88378
bikunin; hepatocyte growth factor activator inhibitor 2	U78095
metalloprotease/disintegrin/cysteine-rich protein precursor	U41766
zinc finger X-chromosomal protein (ZFX)	X59738
ubiquitin	M26880
phospholipase A2	M86400
hypoxanthine-guanine phosphoribosyltransferase (HPRT)	V00530
liver glyceraldehyde 3-phosphate dehydrogenase (GAPDH	X01677
brain-specific tubulin alpha 1 subunit (TUBA1)	K00558
HLA class I histocompatibility antigen C-4 alpha subunit (H	M11886
cytoplasmic beta-actin (ACTB)	X00351
23-kDa highly basic protein; 60S ribosomal protein L13A (I	X56932
40S ribosomal protein S9	U14971

771 + M87772

7 + M63888 + M63889

1854
